# Hematopoietic stem-cell senescence and myocardial repair - Coronary artery disease genotype/phenotype analysis of post-MI myocardial regeneration response induced by CABG/CD133+ bone marrow hematopoietic stem cell treatment in RCT PERFECT Phase 3

**DOI:** 10.1016/j.ebiom.2020.102862

**Published:** 2020-07-04

**Authors:** Markus Wolfien, Denise Klatt, Amankeldi A. Salybekov, Masaaki Ii, Miki Komatsu-Horii, Ralf Gaebel, Julia Philippou-Massier, Eric Schrinner, Hiroshi Akimaru, Erika Akimaru, Robert David, Jens Garbade, Jan Gummert, Axel Haverich, Holger Hennig, Hiroto Iwasaki, Alexander Kaminski, Atsuhiko Kawamoto, Christian Klopsch, Johannes T. Kowallick, Stefan Krebs, Julia Nesteruk, Hermann Reichenspurner, Christian Ritter, Christof Stamm, Ayumi Tani-Yokoyama, Helmut Blum, Olaf Wolkenhauer, Axel Schambach, Takayuki Asahara, Gustav Steinhoff

**Affiliations:** a Department of Systems Biology and Bioinformatics, University Rostock, Institute of Computer Science, Ulmenstrasse 69, 18057 Rostock, Germany; b Hannover Medical School, Institute of Experimental Hematology, Carl-Neuberg-Strasse 1, 30625 Hannover, Germany; c Department of Advanced Medicine Science, Tokai University School of Medicine, Shimokasuya 143, Isehara, Kanagawa 259-1143, Japan; d Nanobridge, LLC. 1-3-5-202, Sawaragi-Nishi Ibaraki Osaka 567-0868, Japan; e Institute of Biomedical Research and Innovation, 2-2 Minatojima-minamimachi, Chuo-ku, Kobe 650-0047, Japan; f Reference and Translation Center for Cardiac Stem Cell Therapy, Department Life, Light and Matter and Department of cardiac surgery, University Medicine Rostock, Schillingallee 35, 18055 Rostock, Germany; g Ludwig-Maximilians-Universität München, LAFUGA Genomics, Gene Center, Feodor-Lynen-Strasse 25, 81377 Muenchen, Germany; h University Medical Center Goettingen, Institute for Diagnostic and Interventional Radiology, Robert-Koch-Strasse 40, 37075 Göttingen, Germany; i Department of Cardiac Surgery, Heart Center University Medicine Leipzig, Strümpellstrasse 39, 04289 Leipzig, Germany; j Heart and diabetes center North Rhine Westfalia, University hospital of the Ruhr university Bochum, Georgstraße 11, 32545 Bad Oeynhausen, Germany; k Medical school Hannover, Department of heart-, thoracic- and vascular surgery, Carl Neuberg Strasse 1, 30625 Hannover, Germany; l Department of cardiothoracic surgery, Osaka city university, 1-4-3, Asahimachi, Abeno. Osaka, 545-8585. Japan; m Department of Cardiac and Vascular Surgery, University heart center Hamburg, Martinistraße. 52, 20246 Hamburg, Germany; n German Heart Center Berlin, Department of Heart-, Thoracic- and Vascular Surgery, Augustenburger Platz 1, 13353 Berlin, Germany

**Keywords:** Clonal hematopoiesis of indeterminate pathology, CHIP, SH2B3, Myocardial regeneration, Cardiac stem cell therapy, Angiogenesis induction, Post myocardial infarction heart failure, Coronary bypass surgery, CABG, Machine learning, ANCOVA, Analysis of covariance, Ang-1, Angiopoeitin 1, AUR, Arch user repository, AUC, Area under curve, BM, Bone marrow, BMMNC, Bone marrow mononuclear cell, BMSC, Bone marrow stem cells, BrdU, Brome deoxyuridine, CABG, Coronary Artery Bypass Graft, CAP-EPC, Concentrated Ambient Particles – Endothelial Progenitor Cells, CD, Cluster of Differentiation, CEC, Circulating endothelial cells, CEC panel, CDs measured in PB, CFU, Colony-forming unit, CHIP, Clonal hematopoiesis of indeterminate potential, CI, Confidence interval, c-KIT/CD117, Stem Cell Factor Receptor c-KIT, CD117, CLARA, Clustering for Large Applications, CPC, cardiac progenitor cell, CSC, cardiac stem cell, DE, Differential gene expression, DNAseq, desoxyribonucleid acid sequencing, EGF, Epidermal growth factor, EGFR, Epidermal growth factor receptor, ELISA, Enzyme-Linked Immunosorbent Assay, EPC, Endothelial Progenitor Cells, EPC panel, CDs measured in PB, EPO, Erythropoietin, EPOR, Erythropoietin receptor, FACS, Fluorescence activated cell sorter, FDR, False discovery rate, GADPH, Glyceraldehyde 3 phosphate dehydrogenase, GATA4, Transcriptional activator that binds to the consensus sequence 5′-AGATAG-3’, GFP, Green fluorescent protein, GMP, Good Manufacturing Practice, GWAS, Genome wide association study, HR, Hazard ratio, HIF, Hypoxia-Inducible Factor, transcription factor, HSC, Hemopoeitic stem cell, hu, human, ICH GCP, Tripartite Guidelines Guideline for Good Clinical Practice, IGF-1, Insuline-like Growth Factor 1, IGFBP, Insuline-like growth factor binding proteine, IHG, Analysis performed in accordance with *ISHAGE guidelines*, IL, Interleukin, InDel, mutation insertion or deletion variant, KSL, mouse bone marrow stem cell subpopulation c-KIT+ Sca-I+ lin-, LAD, Left anterior descending coronary artery, RIVA, LAS, Longitudinal axis strain, LCRC, Loss of cardiac regeneration capacity, LNK, SH2B adapter protein 3 (lymphocyte adapter protein), LVEDV, Left Ventricular End Diastolic Volume, LVEF, Left Ventricular Ejection Fraction, LVESD, Left Ventricular End Systolic Dimension, m, mouse, MI, myocardial infarction, ML, Machine learning, MNC, Mononuclear cells, MRI, Magnetic resonance imaging, 6MWT, 6-Minute Walk Test, NGS, Next Generation Sequencing, NR, non-responder, PB, Peripheral blood, PBMNC, mononuclear cells isolated from peripheral blood, PCR, Polymerase chain reaction, PDGF, Platelet derived growth factor, PDFR, Platelet derived growth factor receptor, PEI, Paul-Ehrlich Institute, PI3K, Phosphoinositide-3-Kinase, PBMNC, Peripheral blood mononuclear cell, PPMC, Pearson Product Moment Correlation, qPCR, Quantitative polymerase chain reaction, R, responder, RFI, Reactome functional interaction, RNASeq, Ribonucleid acid sequencing, ROC, Receiver operating characteristics, RT-PCR, Reverse transcriptase polymerase chain reaction, RWMS, Regional wall motion score, SDF-1, Stromal Cell-derived Factor 1, SH2B3, LNK [Src homology 2-B3 (SH2B3)] belongs to a family of SH2-containing proteins with important adaptor functions, SCF, Stem Cell Factor, SNP, Single nucleotide polymorphism, variant, STEMI, ST- segment Elevation Infarction, SUSAR, Suspected Unexpected Serious Adverse Reaction, TiCoNE, Time course network enrichment, TNF, Tumor Necrosis Factor, t-SNE, t-distributed stochastic neighbour embedding, VCA, Virus-Capsid-Antigen, VEGF, Vascular Endothelial Growth Factor, VEGFR, Vascular Endothelial Growth Factor Receptor, WT, wild type, WGCNA, Weighted gene coexpression network analysis

## Abstract

**Background:**

Bone marrow stem cell clonal dysfunction by somatic mutation is suspected to affect post-infarction myocardial regeneration after coronary bypass surgery (CABG).

**Methods:**

Transcriptome and variant expression analysis was studied in the phase 3 PERFECT trial post myocardial infarction CABG and CD133^+^ bone marrow derived hematopoetic stem cells showing difference in left ventricular ejection fraction (∆LVEF) myocardial regeneration Responders (*n=*14; ∆LVEF +16% day 180/0) and Non-responders (*n=*9; ∆LVEF -1.1% day 180/0). Subsequently, the findings have been validated in an independent patient cohort (*n=*14) as well as in two preclinical mouse models investigating *SH2B3*/LNK antisense or knockout deficient conditions.

**Findings:**

1. Clinical: R differed from NR in a total of 161 genes in differential expression (*n=*23, *q*<0•05) and 872 genes in coexpression analysis (*n=*23, q<0•05). Machine Learning clustering analysis revealed distinct R*vs*NR preoperative gene-expression signatures in peripheral blood acorrelated to *SH2B3* (*p<*0.05). Mutation analysis revealed increased specific variants in R*vsN*R. (R: 48 genes; NR: 224 genes). **2. Preclinical:***SH2B3*/LNK-silenced hematopoietic stem cell (HSC) clones displayed significant overgrowth of myeloid and immune cells in bone marrow, peripheral blood, and tissue at day 160 after competitive bone-marrow transplantation into mice. *SH2B3*/LNK^−/−^ mice demonstrated enhanced cardiac repair through augmenting the kinetics of bone marrow-derived endothelial progenitor cells, increased capillary density in ischemic myocardium, and reduced left ventricular fibrosis with preserved cardiac function. **3. Validation:** Evaluation analysis in 14 additional patients revealed 85% R*vs*NR (12/14 patients) prediction accuracy for the identified biomarker signature.

**Interpretation:**

Myocardial repair is affected by HSC gene response and somatic mutation. Machine Learning can be utilized to identify and predict pathological HSC response.

**Funding:**

German Ministry of Research and Education (BMBF): Reference and Translation Center for Cardiac Stem Cell Therapy - FKZ0312138A and FKZ031L0106C, German Ministry of Research and Education (BMBF): Collaborative research center - DFG:SFB738 and Center of Excellence - DFG:EC-REBIRTH), European Social Fonds: ESF/IV-WM-B34-0011/08, ESF/IV-WM-B34-0030/10, and Miltenyi Biotec GmbH, Bergisch-Gladbach, Germany. Japanese Ministry of Health : Health and Labour Sciences Research Grant (H14-trans-001, H17-trans-002)

**Trial registration:**

ClinicalTrials.gov NCT00950274

## Graphical abstract

**Figure:**
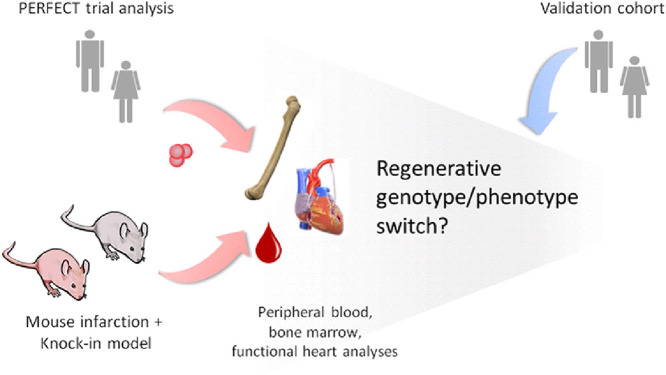

**Research in context**

**Evidence before this study**

The basis for this current work is the randomized double-blinded placebo controlled multicenter Phase 3 PERFECT-trial in which post myocardial infarction (MI) patients after coronary artery bypass graft (CABG) surgery have been treated with intramyocardial CD133^+^ bone marrow derived hematopoetic stem cells (BM-HSC) or Placebo. At the time we identified the correlation of myocardial regeneration with systemic bone marrow response characterized by a preoperative biomarker signature in peripheral blood (PB) of 20 angiogenesis and stem cell related factors [17]. An additional outcome prediction obtained by Machine Learning (ML) received an accuracy rate of 85% for responder (R) and 80% for non-responder (NR). Here, genetic dysregulation of BM-HSC was suspected and now followed up by gene expression and mutational dysregulation analysis. LNK is an adaptor protein coded by the gene *SH2B3* and negatively regulates multiple essential signals in hematopoietic stem cells (HSC). Its regulatory role for BM-HSC in cardiovascular repair remains shallow and will be investigated throughout this underlying manuscript.

**Added value of this study**

In the present series of experiments, we clarified that HSC signaling adaptor gene mutations in *SH2B3* contribute to a polygenic gene expression circuit switch including the genes *PLCG1, LPCAT2, GRB2, AFAP1, AP1B1, KLF8, MARK3* favorable for the cardiac healing process in MI-patients undergoing cardiac recovery after CABG surgery. An integrative ML analysis of preoperative PB enables highly sensitive clinical diagnosis and prediction of cardiac regeneration response after CABG. It may be used for treatment monitoring for cardiac regeneration and give rise to a patient specific ML supported therapy in the future. Our findings in PERFECT about R*vs*NR and in *SH2B3*/LNK^−/−^ mice suggest that the significantly reduced ischemic myocardial damage with preserved cardiac function following MI is mainly due to enhanced angiogenesis in ischemic myocardium.

**Implications of all the available evidence**

This novel approach of disease genotype/phenotype analysis combining gene expression, coexpression, and transcript variant calling in a randomized clinical trial led to the discovery of a polygenic circuit involved in HSC response associated to cardiac regeneration capacity. In the following, the findings were verified by animal studies and assisted by correlation analysis of human and mice. This comparison enabled new insights into adaptor proteins, proliferation signaling, and immune checkpoint regulation controlling for vasculogenesis/angiogenesis and cardiac tissue repair. Recovery of expedient cardiac function was observed through up-regulation of HSC/EPCs circulation and stimulation of immune progenitor cell (PC) proliferation. Our findings show that mutational changes in gene expression transcripts have important implications for formulations of new therapeutic strategies to diagnose and enhance cardiac repair by stem cells.

Alt-text: Unlabelled box

## 1 Introduction

**Fig. 1:**
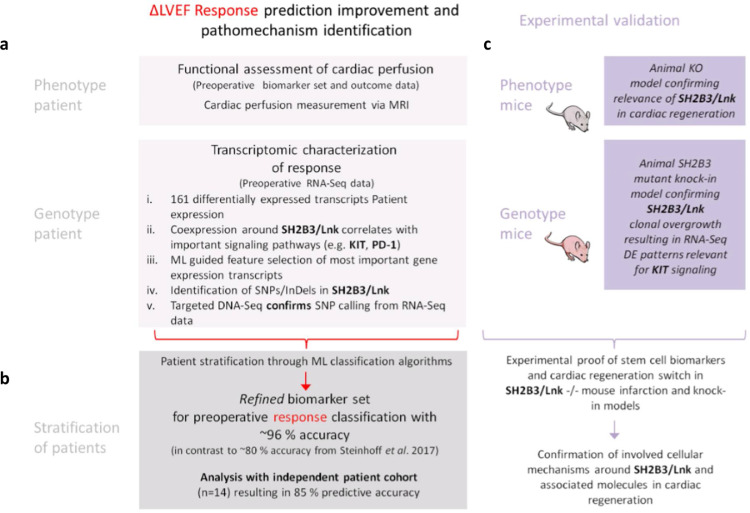
Overview of utilized integrative analysis approach integrating clinical patient data with murine pre-clinical models: Genotype/phenotype analysis in randomised clinical trial PERFECT cardiac regeneration outcome and knock-out animal disease model verification of regulatory genes.

The hematopoietic system has traditionally been considered as an organized, hierarchical system with multipotent, self-renewing stem cells at the top, lineage-committed progenitor cells in the middle, and lineage-restricted precursor cells, which give rise to terminally differentiated cells, at the bottom [[1], [2], [3]]. However, disorders of clonal hematopoiesis of indeterminate pathology (CHIP) has been described in hematological and cardiovascular disease patients and associated to congenital or somatic DNA mutations [4,5]. The question arises, which mutations in stem congenital or somatic cell regulatory genes cause hematopoietic clonal advantage and impact cardiovascular pathology [6,7]. *SH2B3*, which codes for the LNK adaptor protein, is one of the major mutated genes associated with hematopoietic stem cell (HSC) proliferation disorders, such as myelodysplasia, erythrocytosis or leukemia [8,9]. In genome wide association studies (GWAS) of cardiovascular patients, the *SH2B3* phosphorylation related missense variant rs3184504 was found to be associated with increased platelet count, monocyte proliferation, hypertension, peripheral/coronary artery disease, autoimmune disease, and longevity [9], [10], [11], [12], [13], [14], [15]. *SH2B3*/LNK expression regulation is largely unknown, but expected to impact cardiovascular regeneration through *c-KIT*/CD117 expressing hematopoietic, myeloid, lymphocytic, endothelial, and mesenchymal progenitor cells in blood [9,10]. In contrast to this, intracardiac *SH2B3*/LNK expression was found to be associated with pressure overload cardiac hypertrophy regulation [16]. At present, the regulatory role of *SH2B3* in stem cell proliferation and inflammation response remains unclear in patients with coronary artery disease, especially in post-myocardial infarction repair leading either to regeneration or inflammatory fibrosis of the myocardium [9,13]. Furthermore, it is unclear, if a monogenic switch of *SH2B3* gene expression or SNP altered LNK protein function in bone marrow stem cells is able to control cardiac regeneration by altering bone marrow response [9]. Moreover, frequency and type of *SH2B3* clonal mutations of HSC of patients with cardiac disease is unknown and may have impact on variable pathology. In the recent outcome analysis of the phase 3 clinical PERFECT trial we are investigating intramyocardial transplantation of c-KIT/CD117^+^/CD133^+,^/CD34^+^ bone marrow derived hematopoeitic stem cells (BM-HSC) in post-myocardial infarction (MI) coronary artery bypass graft (CABG) patients. We found striking differences in induction of cardiac regeneration in 60% of BM-HSC treated and placebo groups characterized by a preoperative Machine Learning (ML) signature in peripheral blood (PB) [17]. Responders (R) *vs*. non-responders (NR) were significantly different preoperatively, with R characterized by increased peripheral blood c-KIT/CD117^+^/CD133^+^/CD34^+^ circulating stem cells (EPC), increased thrombocytes, while NR had increased Erythropoeitin (EPO), Vascular endothelial growth factor (VEGF) and N-terminal pro b-type natriuretic peptide (NTproBNP) in preoperative serum [17]. Induced bone marrow stem cell proliferation responses in R was suspected to be due to adaptor protein *SH2B3*/LNK activity [17]. Based on this, we first performed variant and gene expression analyses in PERFECT responders vs. non-responders and compared diagnostic R*vs*NR signatures (Fig. 1A). Then we validated the effect on R/NR signature switch in *SH2B3*/LNK deficient mouse models to investigate the role of HSC dysfunction in cardiac repair . Final evaluation of the signatures was performed in an independent patient cohort and by mouse/man correlation analysis (Fig. 1B,C).

## 2 Methods

### 2.1 Study Design

Peripheral blood bone marrow response was studied by whole transcriptome analysis in the randomized phase 3 PERFECT trial biomarker subgroup (total *n=*39, Rostock *n=*23, Hannover *n=*14, Leipzig *n=*2) [17]. Primary analysis was performed at the Rostock trial site with available biobank, clinical (per protocol), and biomarker data (*n=*23). CD133^+^ BMSC treated (*n=*13) and placebo controls (*n=*10) were equally distributed. In the biomarker patient cohort (*n=*23; Placebo/CD133^+^9/14), we investigated systemic bone marrow stem cell response in peripheral blood in Responders (R) classified by the difference in left ventricular ejection fraction (∆LVEF) ≥5 % after 180d (*n=*14; Placebo/CD133^+^ 7/7) and Non-responders (NR) classified by ∆LVEF <5 % after 180d (*n=*9; Placebo/CD133^+^ 5/4) in both treatment groups (intramyocardial Placebo *vs*. CD133+). For validation variant expression analysis, MRI, and clinical outcome was tested in 14 patients from the Hannover trial center with available clinical data.

### 2.2 Ethical approval and trial setting

RNA sequencing (RNA-Seq) analysis and mRNA RT-PCR in PB: Samples were taken from informed study patients who gave their written consent according to the Declaration of Helsinki (Approval by the Ethical committee, Rostock University Medical Center 2009; No. HV-2009-0012). Analyses and examinations were performed before unblinding of the trial and under careful adherence to the protection of data privacy (pseudonyms).

### 2.3 Transcriptome Analysis of EDTA blood samples using NGS

RNA of frozen EDTA blood samples was isolated in a three step procedure: First, the GeneJET Stabilized and Fresh Whole Blood RNA KIT (Thermo Scientific) was used following manufacturer's instructions. Second, isolated RNA was precipitated with 2.5 volumes ethanol under high salt conditions (10 % of 3 M sodium acetate, pH 5•2). After DNase digest (Thermo Scientific), the RNA was finally purified using Agencourt RNAClean XP beads (Beckman Coulter). Purified RNA was analyzed on a Bionalyzer (Agilent) using RNA 6000 Nano Chips (Agilent). Quality controlled RNA was used to construct sequencing libraries using the Universal Plus mRNA–Seq Technology (Nugen) according to manufacturer's instructions. Briefly, mRNA was selected by oligo d(T) beads, reverse transcribed and cDNA from Globin messengers was removed by the Globin depletion module (Nugen). Quality controlled and quantified libraries were sequenced on a HiSeq1500 system (Illumina) in single-end mode (100 nt read length). For RNA-Seq data analysis, adapter clipping and quality trimming procedures for data pre-processing were performed and aligned the reads to the hg19 genome (for patient data) and mm10 genome (for murine data) with the aid of kallisto, respectively. Differential expression analysis was performed using the likelihood ratio test of the DESeq2 package (genes with >2-fold change and a *q*-value < 0•05 are considered as significantly differentially expressed). The gene set enrichment analysis (GSEA), annotation, including functional annotation clustering and functional classification, was performed according to Enrichr [18].

### 2.4 Variant calling from transcriptomic data

The previously preprocessed human RNA-Seq datasets were realigned to the hg19 reference (Ensembl version 94) with Star (2-pass mode). The variant calling was applied by the Gatk toolkit [19] with specialized filters (e.g. variants are only considered, if they are confirmed with five independent reads - a comprehensive workflow is shown in Supplement Figure S1.

### 2.5 Experimental CRISPR-Cas9 induced SH2B3/LNK antisense silencing mouse model

#### 2.5.1 Lentiviral vector production

The lentiviral vectors pRRL.U6.Lnk-sgRNA.EFS.dTomato.pre or pRRL.U6.NT-sgRNA.EFS.eBFP2.pre were packaged into viral particles by transfection of 10 µg vector, 12 µg pcDNA3.GP.4xCTE, 6 µg pRSV-Rev and 2 µg pMD2.G into HEK-293T cells in 10 cm plates using the calcium-phosphate method. Medium change was performed 6-8 h later and viral supernatants were harvested 30 h and 54 h post-transfection. The lentiviral supernatants were pooled and concentrated by ultracentrifugation. Vector titers were determined on lineage-negative mouse bone marrow cells.

#### 2.5.2 Competitive bone marrow transplantation

Lineage-negative mouse bone marrow cells were isolated by flushing femurs, tibias and pelves of GFP^+^Cas9 mice (B6J.129(Cg)-Gt(ROSA)26Sortm1.1(CAG-cas9*,-EGFP)Fezh/J) (IMSR Cat# JAX:026179, RRID:IMSR_JAX:026179) followed by lineage depletion using the MojoSort Mouse Hematopoietic Progenitor Cell Isolation Kit (BioLegend). Cells were prestimulated for 24 hin StemSpan (Stem Cell Technologies) supplemented with 100 U/mL penicillin and 100 µg/mL streptomycin (PAA), 2 mM L-glutamine (Biochrom), 20 ng/mL mTPO, 20 ng/mL mIGF-2, 10 ng/mL mSCF, 10 ng/mL hFGF-1 (all cytokine: Peprotech), 20 µg/mL meropenem (Hexal) and 10 µg/mL heparin (Ratiopharm). Cells were transduced at a density of 1•5 × 10^6^ cells/mL at an MOI of 30. For competitive transplantation, equal cell numbers of cells transduced with lentiviral vector pRRL.U6.Lnk-sgRNA.EFS.dTomato.pre or pRRL.U6.NT-sgRNA.EFS.eBFP2.pre were mixed and about 5 × 10^5^ cells per mouse were transplanted into irradiated (2 × 4•5 Gy) GFP^−^ CD45.2 B6 (C57BL/6) recipients. Cell mixtures were analyzed by flow cytometry 4-5 d after transduction to confirm equal distribution of both cell fractions. At week 4, 8, 12, and 18 after transplantation, blood counts were performed and peripheral blood was analyzed for donor cell engraftment and lineage distribution by flow cytometry. All experimental procedures were conducted in accordance with the German Animal Protection Law Guidelines for the Care and Use of Laboratory Animals and the study protocol was approved by the Ethics Committee of the LAVES (Lower Saxony State Department for Consumer and Food Safety Protection), Germany.

#### 2.5.3 Experimental SH2B3/LNK knockout model

*T*he *SH2B3/LNK*^–/–^ mouse strain was generated as described previously [10]. C57BL/6 mice (CLEA Japan, Tokyo, Japan) were used as WT control mice. GFP transgenic mice (GFP-Tg mice; C57BL/ 6TgN [act EGFP] Osb Y01) were mated with WT mice or *SH2B3/LNK^–/–^* mice and generated *WT/GFP* mice or *SH2B3/LNK^–/–^/GFP* mice, respectively, for BM transplantation (BMT) studies. All experimental procedures were conducted in accordance with the Japanese Physiological Society Guidelines for the Care and Use of Laboratory Animals and the study protocol was approved by the Ethics Committee in RIKEN Center for Developmental Biology.

#### 2.5.4 Statistical analysis

The results were statistically analyzed using a software package (Statview 5.0, Abacus Concepts Inc, Berkeley, CA). All values were expressed as mean±standard deviation (mean±SD). The comparisons among more than three groups were made using the one-way analysis of variances (ANOVA) in Prism 4 (GraphPad Software, San Diego, CA). Post hoc analysis was performed by Tukey's multiple comparison test, Mann-Whitney comparison test or Bonferroni post-hoc test. Differences of *p<*0•05 were considered to denote statistical significance.

#### 2.5.5 Data analysis with machine learning

Identifying key features and classification of the comprehensive patient data was obtained by employing supervised and unsupervised Machine Learning (ML) algorithms . We preprocessed the data, while removing features with low variance and high correlation for dimension reduction, following best practices recommendations. We compared the following supervised algorithms: AdaBoost (AB), Gradient Boosting (GB), Support Vector Machines (SVM), and Random Forest (RF) [20]. We employed classifiers that are suitable for training on small data sets for a comparison of features given little training and chose the most appropriate algorithm according to accuracy and robustness towards overfitting [21]. Supervised ML models were 10-fold cross-validated and 100 times repeated. We then applied feature selection for the AB, GB, and RF classifiers to further reduce the number of features to <20. We employed principial component analysis (PCA), t-distributed Stochastic Neighbor Embedding (tSNE), and Uniform Manifold Approximation and Projection (UMAP, https://arxiv.org/abs/1802.03426) for unsupervised machine learning classification and nonlinear dimensionality reduction.

#### 2.5.6 WGCN analysis

Weighted gene coexpression network analysis (WGCNA) was performed by applying the R package “WGCNA” to the human RNA-Seq count data. We first constructed the topological overlap matrix (TOM) of all investigated transcripts (~160,000) using the soft thresholding method. We calculated the eigenvalues of the transcripts and evaluated the adjacency based on distance. We subjected transcripts to hierarchical clustering (average linkage) and assigned transcripts with the dynamic hybrid method into groups. We computed the connectivity based on the interaction partners (*k*) and evaluated the gene significance, which represents the resulting module membership.

## 3 Results

In our analysis we integrated clinical genotype and phenotype data as well as experimental gene knockout animal modeling in which we aimed to unravel and validate diagnostic associations of blood, bone marrow, and heart tissue (Fig. 1). At the phenotypic level, left ventricular function measured in magnetic resonance imaging (MRI) showed recovery with a mean difference in primary endpoint outcome ∆LVEF (d.180/0) in Responders (R) +16% *vs*. Non-responders (NR) -1•1% (*p<*0•01, t-test; Mann-Whitney Rank Sum test) (Table 1). Significant difference was found in R for myocardial capillary perfusion measured in MRI with increased epicardial (*p=*0•038, t-test; Mann-Whitney Rank Sum test) and endocardial (*p=*0•024, t-test; Mann-Whitney Rank Sum test) maximal upslope velocity after 180 days (Table 1).

**Table 1:** Left ventricular function and myocardial perfusion outcome analysis. MRI evaluation biomarker .subgroup (*n=*23) for primary endpoint (delta LVEF 180/0), myocardial function by long-axis-strain analysis, and myocardial perfusion by semiquantitative analysis (mean value of 16 segments). Responders (*n=*14) were classified according to primary endpoint outcome by delta LVEF >5% d. 180/0, non-responders (*n=*9) by delta LVEF <5% d.180/0. Long-axis-strain measurement was performed according to Giesdal O et al [22], myocardial perfusion was measured according to Mordini FE et al [23].

	Baseline (day 0)	SD	Primary endpoint (day 180)	SD	Delta (180/0)	P-value (t-test; Mann-Whitney Rank Sum test)
**LVEF (%)**						
Responder (n evaluable=14)	33,3	5,0	49,3	6,7	16,0	**P≤0.001**
Non-Responder (n evaluable = 9)	33,3	7,5	32,2	9,1	-1,1	*P=*0.781
Responder – Non-responder	0		17,1		17,1	**P≤0.001**
**Long axis strain (LAS global)**						
Responder (n evaluable=14)	-7,6	2,2	-9,4	2,2	-1,8	***P=*0.032**
Non-Responder (n evaluable = 9)	-8,4	2,7	-9,5	2,7	-1,1	*P=*0.402
Responder – Non-responder	+0,8		+0,1		-0,7	*P=*0.416
**Maximal upslope epicardial**						
Responder (n evaluable=13)	27,0	10,4	37,4	17,3	10,4	***P=*0.018**
Non-Responder (n evaluable = 9)	29,6	11,5	28,7	9,1	-0,9	*P=*0.895
Responder – Non-responder	-2,6		+8,7		11,3	***P=*0.038**
**Maximal upslope endocardial**						
Responder (n evaluable=13)	29,7	12,6	42,4	19,5	12,7	***P=*0.014**
Non-Responder (n evaluable = 9)	33,6	11,5	33,8	9,7	0,2	*P=*0.967
Responder – Non-responder	-3,9		8,6		12,5	***P=*0.024**

### 3.1 A: Clinical phenotype and genotype of cardiac regeneration response

#### 3.1.1 Gene expression analysis

In addition to previously identified correlating angiogenesis biomarkers and *SH2B3*/LNK RT-PCR analysis of PB [17], we performed an in-depth gene expression analysis. In order to study transcriptome profile patterns of R and NR signatures, the capture of polyadenylated RNA was conducted by high throughput sequencing. The experimental procedure included a depletion of cDNA derived from Globin messengers transcriptome to enable high resolution RNA-Seq in preoperative PB samples from 23 patients (14 R, 9 NR).

**Fig. 2:**
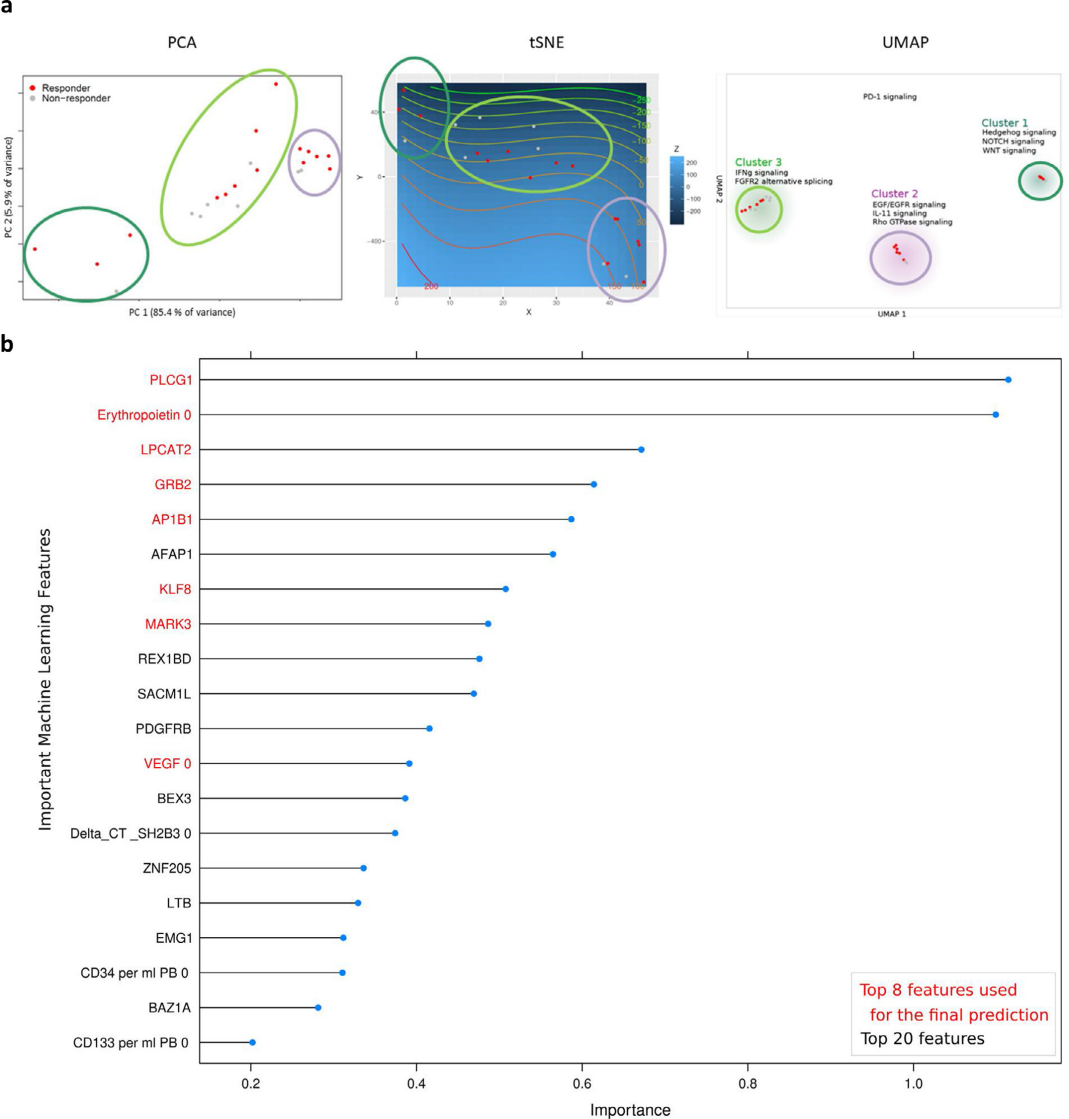
**a**: ML subgroup clusters of cohort study (Responder, *n*=14, red points; Non-responder, *n*=9, grey points). **b**: Machine learning feature selection on clinical trial research data and RNA-Seq data. Accuracy comparison for the supervised prediction of the patient responsiveness using only preoperative data. Results are obtained after feature selection and subsequent prediction with two independent classifiers. The graph shows the true positive prediction weights of the ML model (RF for feature selection and SVM for final prediction). Combinations and subsets of these features have been subsequently used to train the final model. The importance indicates a hierarchy of the most relevant features needed for a classification.

Differential gene expression analysis revealed distinct R/NR patterns consisting of 161 significant genes (q<0•05) out of ~160,000 transcripts. The highest significance was found for 122 unique genes (R/NR: q=0•02) (Supplementary Data SD1a). Clustering for all used methods examined potentially occuring patient subgroups. Three independent clustering analyses (PCA, tSNE, and UMAP) on all gene expression read counts showed a clear distinction between patients (Fig. 2a, Supplementary Figure 1c). All methods clustered the patients into the same defined subgroups, which did not change. Pathway analysis of differing genes was subsequently conducted on each of the three clusters to investigate the specific differences towards the gene signaling among these subgroups (Fig. 2a, Supplementary Data SD1b). Then, we performed the coexpression analysis by WGCNA, an so-called guilt-by-association approach, to be able to interconnect *SH2B3*/*LNK* with similarly regulated transcripts. *SH2B3*/*LNK* was identified to be coexpressed within a cluster of 872 genes (Supplementary Data SD1c). The corresponding pathways of the coexpressed genes were c-KIT receptor signaling pathway, as well as EGF, PDGF, TCR, IL6, and Interferon 1 signaling (Table 2).

**Table 2:** Gene set enrichment pathway analysis utilized by Enrichr for differential gene expression, coexpression, and transcriptomic variants is based on preoperative RNA-Seq data . The 161 significantly differentially expressed transcripts identified by DESeq2 and 872 WGCNA transcripts have been applied to the pathway enrichment analysis of Enrichr for the WikiPathways and BioCarta database. The obtained pathways are significantly enriched according to the adjusted p-value < 0.05

Type of data analysis	Database	Pathway Term	p-value	**Adjusted p-value**
**Differential expression**	**BioCarta**	Ras Signaling Pathway_Homo sapiens_h_rasPathway	0,0004127	0,0235251
		AKT Signaling Pathway_Homo sapiens_h_aktPathway	0,0076997	0,1097214
		Cyclin E Destruction Pathway_Homo sapiens_h_fbw7Pathway	0,0018925	0,0447290
		E2F1 Destruction Pathway_Homo sapiens_h_skp2e2fPathway	0,0023542	0,0447290
		Control of Gene Expression by Vitamin D Receptor_Homo sapiens_h_vdrPathway	0,0169139	0,1722122
		Beta-arrestins in GPCR Desensitization_Homo sapiens_h_bArrestinPathway	0,0181276	0,1722122
	**WikiPathways**	Hematopoietic Stem Cell Differentiation_Homo sapiens_WP2849	0,0050742	0,2572584
		Translation Factors_Homo sapiens_WP107	0,0063783	0,2572584
		AMPK Signaling_Homo sapiens_WP1403	0,0145744	0,4408766
		RalA downstream regulated genes_Homo sapiens_WP2290	0,0034194	0,2572584
		EGFR1 Signaling Pathway_Mus musculus_WP572	0,0384020	0,4723207
		IL-6 signaling Pathway_Mus musculus_WP387	0,0353539	0,4723207
		Androgen receptor signaling pathway_Homo sapiens_WP138	0,0284065	0,4723207
		Striated Muscle Contraction_Mus musculus_WP216	0,0369516	0,4723207
		Striated Muscle Contraction_Homo sapiens_WP383	0,0321361	0,4723207
**Coexpression**	**BioCarta**	EGF Signaling Pathway_Homo sapiens_h_egfPathway	0,0001867	0,0148901
		PDGF Signaling Pathway_Homo sapiens_h_pdgfPathway	0,0004153	0,0148901
		Control of Gene Expression by Vitamin D Receptor_Homo sapiens_h_vdrPathway	0,0004653	0,0148901
		IL 6 signaling pathway_Homo sapiens_h_il6Pathway	0,0023696	0,0440702
		Cell to Cell Adhesion Signaling_Homo sapiens_h_cell2cellPathway	0,0020125	0,0440702
		Eukaryotic protein translation_Homo sapiens_h_eifPathway	0,0027544	0,0440702
		T Cell Receptor Signaling Pathway_Homo sapiens_h_tcrPathway	0,0037212	0,0486744
		Map Kinase Inactivation of SMRT Corepressor_Homo sapiens_h_egfr_smrtePathway	0,0040712	0,0486744
		Internal Ribosome entry pathway_Homo sapiens_h_iresPathway	0,0045632	0,0486744
		TPO Signaling Pathway_Homo sapiens_h_TPOPathway	0,0080521	0,0515331
		Inhibition of Cellular Proliferation by Gleevec_Homo sapiens_h_gleevecpathway	0,0067889	0,0515331
		Erk1/Erk2 Mapk Signaling pathway_Homo sapiens_h_erkPathway	0,0067889	0,0515331
		Sprouty regulation of tyrosine kinase signals_Homo sapiens_h_spryPathway	0,0056252	0,0515331
		How Progesterone Initiates the Oocyte Maturation_Homo sapiens_h_mPRPathway	0,0074082	0,0515331
		mTOR Signaling Pathway_Homo sapiens_h_mTORPathway	0,0080521	0,0515331
	**WikiPathways**	Kit Receptor Signaling Pathway_Mus musculus_WP407	0,0000385	0,0056566
		mRNA processing_Mus musculus_WP310	0,0000874	0,0064241
		Interferon type I signaling pathways_Homo sapiens_WP585	0,0002457	0,0101753
		EGF/EGFR Signaling Pathway_Homo sapiens_WP437	0,0003291	0,0101753
		EPO Receptor Signaling_Homo sapiens_WP581	0,0004153	0,0101753
		EPO Receptor Signaling_Mus musculus_WP1249	0,0004153	0,0101753
		mRNA Processing_Homo sapiens_WP411	0,0007745	0,0162654
		PDGF Pathway_Homo sapiens_WP2526	0,0013840	0,0254306
		IL-6 signaling pathway_Homo sapiens_WP364	0,0018385	0,0270260
		IL-7 Signaling Pathway_Mus musculus_WP297	0,0018385	0,0270260
		Translation Factors_Mus musculus_WP307	0,0022338	0,0298523
		IL-3 Signaling Pathway_Homo sapiens_WP286	0,0026782	0,0325360
		EGFR1 Signaling Pathway_Mus musculus_WP572	0,0028773	0,0325360
**SNP-Responder**	**BioCarta**	Calcium Signaling by HBx of Hepatitis B virus_Homo sapiens_h_HBxPathway	0,0025401	0,0379514
		T Cell Receptor Signaling Pathway_Homo sapiens_h_tcrPathway	0,0027108	0,0379514
		IL 4 signaling pathway_Homo sapiens_h_il4Pathway	0,0025401	0,0379514
		Repression of Pain Sensation by the Transcriptional Regulator DREAM_Homo sapiens_h_dreampathway	0,0025401	0,0379514
		Nuclear receptors coordinate the activities of chromatin remodeling complexes and coactivators to facilitate initiation of transcription in carcinoma cells_Homo sapiens_h_rarrxrPathway	0,0025401	0,0379514
	**WikiPathways**	mRNA Processing_Homo sapiens_WP411	0,0000037	0,0005582
		Diurnally Regulated Genes with Circadian Orthologs_Homo sapiens_WP410	0,0001005	0,0037941
		Diurnally Regulated Genes with Circadian Orthologs_Mus musculus_WP1268	0,0001005	0,0037941
		Exercise-induced Circadian Regulation_Mus musculus_WP544	0,0001005	0,0037941
		mRNA processing_Mus musculus_WP310	0,0001861	0,0056192
		IL-2 Signaling Pathway_Homo sapiens_WP49	0,0012438	0,0268307
		Cytoplasmic Ribosomal Proteins_Homo sapiens_WP477	0,0010767	0,0268307
		IL-4 Signaling Pathway_Homo sapiens_WP395	0,0025723	0,0409333
		RANKL/RANK Signaling Pathway_Homo sapiens_WP2018	0,0027108	0,0409333
		Apoptosis-related network due to altered Notch3 in ovarian cancer_Homo sapiens_WP2864	0,0024383	0,0409333
**SNP-Non-responder**	**BioCarta**	Mechanism of Protein Import into the Nucleus_Homo sapiens_h_npcPathway	0,0017807	0,1887530
		Thrombin signaling and protease-activated receptors_Homo sapiens_h_Par1Pathway	0,0187440	0,2862846
		Role of MEF2D in T-cell Apoptosis_Homo sapiens_h_mef2dPathway	0,0248418	0,2862846
		ADP-Ribosylation Factor_Homo sapiens_h_arapPathway	0,0227045	0,2862846
		Spliceosomal Assembly_Homo sapiens_h_smPathway	0,0114323	0,2862846
		Cycling of Ran in nucleocytoplasmic transport_Homo sapiens_h_ranPathway	0,0144950	0,2862846
		Role of Ran in mitotic spindle regulation_Homo sapiens_h_ranMSpathway	0,0215374	0,2862846
		Erythropoietin mediated neuroprotection through NF-kB_Homo sapiens_h_eponfkbPathway	0,0297088	0,2862846
	**WikiPathways**	Proteasome Degradation_Homo sapiens_WP183	0,0000001	0,0000161
		Allograft Rejection_Homo sapiens_WP2328	0,0000055	0,0007073
		Proteasome Degradation_Mus musculus_WP519	0,0000215	0,0018437
		G13 Signaling Pathway_Mus musculus_WP298	0,0007868	0,0505532

#### 3.1.2 Stratification of patients by ML selected features and correlation analysis

**Fig. 3:**
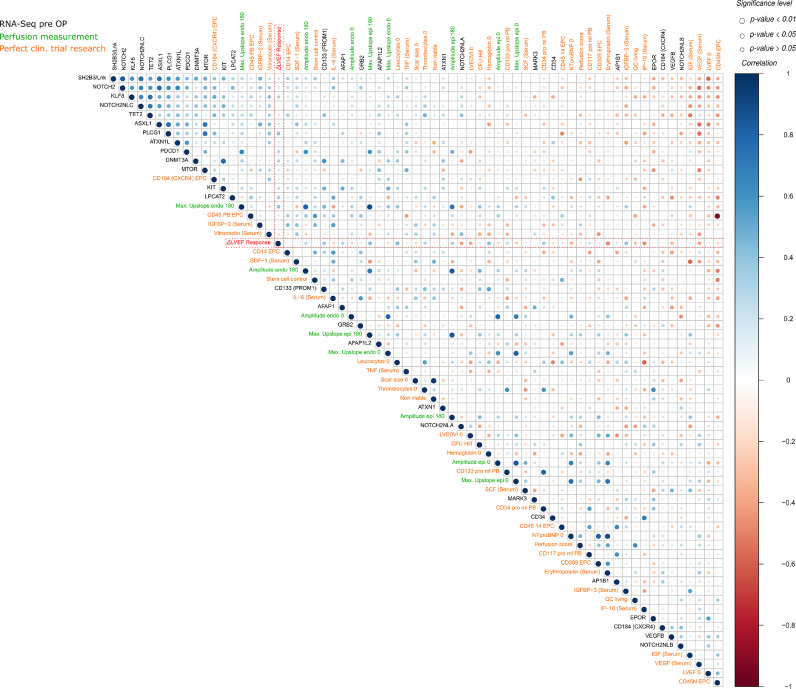
Integration of RNA-Seq, perfusion, and clinical trial research data for Pearson correlation analysis. Comparison of peripheral blood (PB) circulating cells and biomarkers (orange), MRI myocardial perfusion parameters (green), and human PB gene expression data (RNA-Seq) (black). The ΔLVEF response (red) is highlighted for an improved visual analysis of important correlations. The color scale, ranging from *1* to *-1* in the upper panel (blue to red), represents the correlation between the different factors. The size of the dots represents the significance (*p<*0,01, *p<*0,05, and p>0,05; Pearson correlation) of the respective correlation (For interpretation of the references to color in this figure legend, the reader is referred to the web version of this article.).

ML feature selection was applied as an independent method identifying the most important features among all gene expression and PERFECT trial outcome data. A first model achieved a prediction accuracy of 90% (ROC AUC 91•2%; CI: 89•4-93•0) when selecting the most important top 20 features as potential biomarkers (Fig. 2b). An integrational correlation analysis was applied to identify interrelations among the transcriptomic and phenotypic layer as well as between known angiogenesis and immune response markers. In particular, we correlated previously identified preoperative biomarkers for R*vs*NR found in the prior PERFECT trial analysis [17], ML top-selected genes (*PLCG1, LPCAT2, GRB2, KLF8, AFAP1, MARK3, AP1B1*), CHIP-related genes (*TET2, ASXL-1, DNMT3A*), previously identified adaptor protein LNK coding gene SH2B3, and related pathways (*EPOR, KIT, KIT-L, PROM1*/CD133, *NOTCH2, PDCD1*/PD-1, *ATXN1L, MTOR* genes) as well as myocardial perfusion parameters (Fig. 3). Top-listed correlations (*p<*0•05; Pearson correlation coefficient) were found for *SH2B3* to the gene expression of *NOTCH2, KLF8, NOTCH2NLC, TET2, ASXL1, PLCG1,* and *ATXN1L* (Fig. 3). ML-top listed *PLCG1, LPCAT2* were correlated to ∆LVEF response (p>0•05; Pearson correlation coefficient), *PDCD1*/PD-1 to ∆LVperfusion (*p<*0•05). Response was also correlated to increased *PROM1*/CD133 RNA, *AFAP1* RNA, myocardial perfusion (∆ maximal upslope gradient epicardial after 180 days), preoperative leukocyte count, CD34 count, IGFBP3 serum protein, and hemoglobin (p>0•05; Pearson correlation coefficient; *n=*23). NR (negative correlation to ΔLVEF Response) correlated to preoperative LVEDV Index, *VEGF-B, NOTCH2NLA* gene expression, serum levels of NT proBNP, VEGF, Erythropoietin, and IP10 (*p<*0•05; Pearson correlation coefficient; *n=*23). Examplarily, an even higher complexity of differential gene transcript correlations to different genes were demonstrated for *PROM1*/CD133 and *NOTCH2* on the isoform level(Supplemental Fig. S2).

#### 3.1.3 Gene variant analysis

**Fig. 4:**
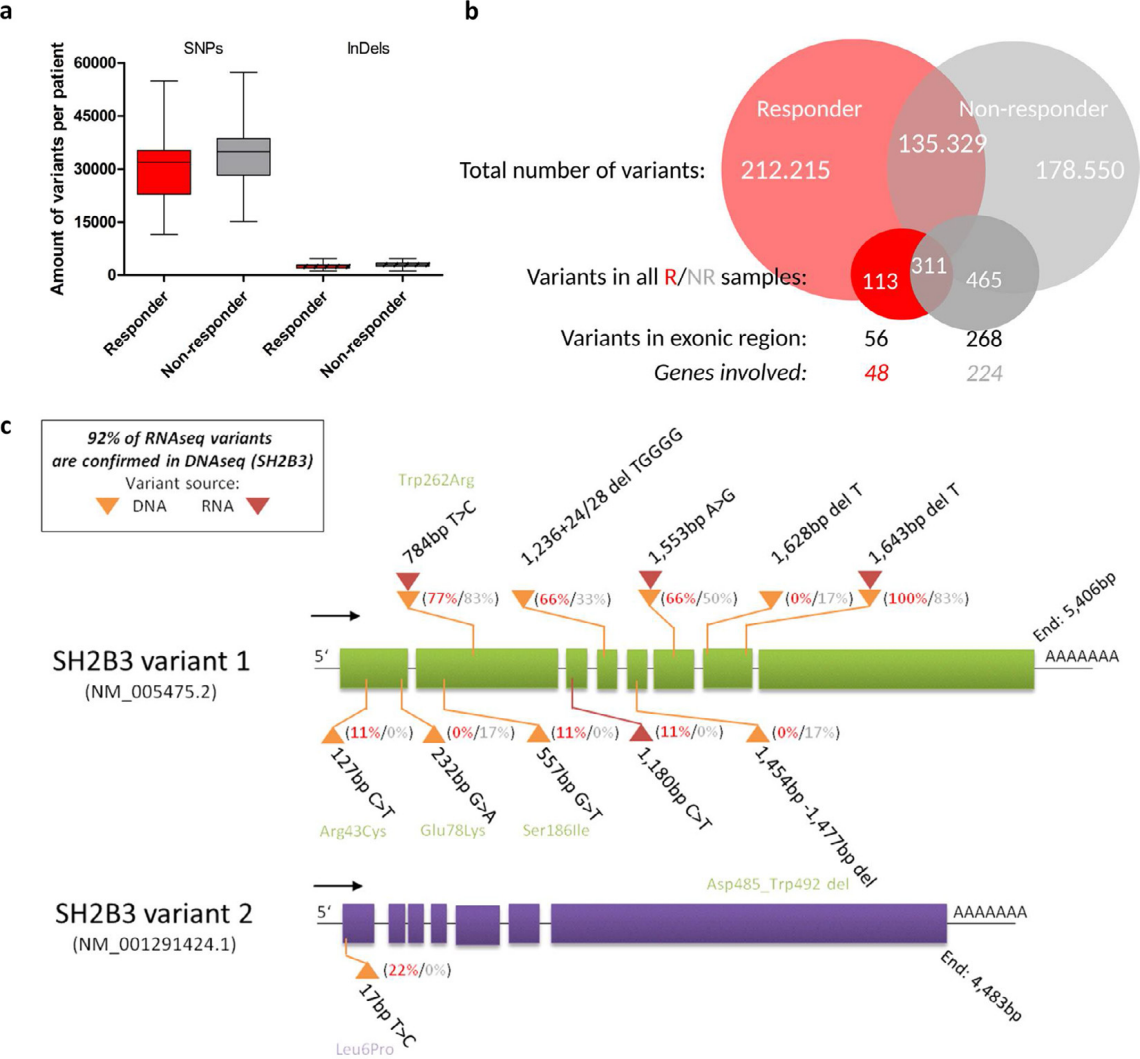
Summary of genetic mutation signature analysis in PERFECT patients via sequencing analysis. **a**: Transcriptomic variants identified through RNA-Seq data analysis. Plot shows the average number of variants (SNPs and InDels) per patient that have been identified by applying our customized transcriptomic variant calling pipeline and filtering approaches. SNPs and InDels are considered as successfully called, if at least five independent reads support the individual variant. **b**: Venn diagram for the R*vs*NR variant comparison, exonic region association, and unique gene identification. **c**: Targeted DNA-Seq (yellow triangle) and RNA-Seq (red triangle) variant summary of SH2B3. The plot shows the ratio of SNP/del sites that are identified in Responders (red) and Non-responders (grey) as well as the possible amino acid transfer from its origin to its potential replacement (For interpretation of the references to color in this figure legend, the reader is referred to the web version of this article.).

Transcriptomic mutation signature analysis performed on RNA-Seq data revealed increased specific variants in NR *vs*. R (NR: 465/178550 variants were contained in all NR, involved 224 genes with 268 exon regions; R: 113/212215 variants contained in all R, involved 48 genes with 56 exon regions (Supplemental data SD1d) (Fig. 4a, 4b). The DNA sequencing (DNA-Seq) confirmed more than 90% of the variants that were called in SH2B3 from RNA-Seq data (Fig. 4c, Supplemental Table 2). Total amount of variants, SNPs, and InDel mutations were not different in R*vs*NR (Fig. 4a, 4b). Main pathways possibly affected by variants were proteasome degradation (NR) and mRNA processing (R) (Table 3). Frequent mutations were present in all top-listed R/NR correlating genes including *SH2B3*/LNK, *NOTCH2, PDCD1*/PD-1, *VEGF-B, PLCG1, GRB2, PROM1*/CD133, *mTOR*, but also in the CHIP-related genes *DNMT3, TET2, ASXL1* that were identified by RNA-Seq SNP calling (Supplementary Data SD1d). Moreover, variants in the reference gene *GAPDH* used for RT-PCR were observed with differences in ΔΔCT calculation as compared to Pol2a (Supplementary Data SD1e). Therefore, RT-PCR gene expression previously used for *SH2B3* [17] was not used for the final outcome analysis. In addition, the Src-family adaptor protein coding gene *SH2B3*/LNK was found to be modified by SNP in DNA-Seq analysis of all patients (23/23) by deletions (100%) or nucleotide exchange (100%), with 83% of SNP resulting in amino acid substitution (Table 3). In RNA-Seq transcriptome analysis, the SNP variant rs3184504 p.Trp262Arg amino acid exchange in the pleckstrin binding domain was found in DNA-Seq (78%) and in RNA-Seq (83%) (Fig. 4c, supplementary Data SD1d, Table S2). Patients with rs3184504 SNPs in DNA-Seq (*n=*17/23) were distributed: R 12/14, NR 5/9. In order to validate the influence of single gene silencing on R/NR gene circuit and the resulting phenotype, we studied gene knockout effects of *SH2B3* in mouse models.

**Table 3:** **Variant frequency of the SH2B3 Gene by** DNA sequencing analysis. Variants, amino acid exchange, and frequency (homozygous > 50%, heterozygous > 25%). LVEF Responder (R) and non-responder (NR) are indicated. Sequencing analysis DNA was performed on peripheral blood by Centogene GmbH, Rostock. Validation was performed by mRNA sequencing performed by GeneCenter, LMU Munich.

	DNA seq SH2B3	amino acid exchange		
study ID	Results (SNP)		transcript	zygosity
				
RTC106	c.784T>C	p.Trp262Arg	NM_005475.2	heterozygous
NR	c.1236+24_1236+28delTGGGG		NM_005475.2	heterozygous
	c.*1553A>G		NM_005475.2	heterozygous
	c.*1643delT		NM_005475.2	heterozygous
				
RTC133	c.784T>C	p.Trp262Arg	NM_005475.2	heterozygous
Resp	c.1236+24_1236+28delTGGGG		NM_005475.2	heterozygous
	c.*1553A>G		NM_005475.2	heterozygous
	c.*1643delT		NM_005475.2	heterozygous
				
RTC124	c.127C>T	p.Arg43Cys	NM_005475.2	heterozygous
Resp	c.784T>C	p.Trp262Arg	NM_005475.2	heterozygous
	c.1236+24_1236+28delTGGGG		NM_005475.2	heterozygous
	c.*1553A>G		NM_005475.2	heterozygous
	c.*1643delT		NM_005475.2	heterozygous
				
RTC117	c.784T>C	p.Trp262Arg	NM_005475.2	heterozygous
Resp	c.1236+24_1236+28delTGGGG		NM_005475.2	heterozygous
	c.*1553A>G		NM_005475.2	heterozygous
	c.*1643delT		NM_005475.2	heterozygous
				
RTC146	c.*1643delT		NM_005475.2	heterozygous
NR				
RTC137	c.784T>C	p.Trp262Arg	NM_005475.2	homozygous
NR	c.*1553A>G		NM_005475.2	homozygous
	c.*1643delT		NM_005475.2	heterozygous
				
RTC140 NR	c.784T>C	p.Trp262Arg	NM_005475.2	homozygous
	c.1236+24_1236+28delTGGGG		NM_005475.2	heterozygous
	c.*1553A>G		NM_005475.2	heterozygous
	c.*1643delT		NM_005475.2	heterozygous
				
RTC099 NR	c.784T>C	p.Trp262Arg	NM_005475.2	heterozygous
	c.*1628delT		NM_005475.2	heterozygous
				
RTC139 R	c.17T>C	p.Leu6Pro	NM_001291424.1	heterozygous
	c.784T>C	p.Trp262Arg	NM_005475.2	heterozygous
	c.1236+24_1236+28delTGGGG		NM_005475.2	heterozygous
	c.*1553A>G		NM_005475.2	heterozygous
	c.*1643delT		NM_005475.2	heterozygous
				
RTC145 R	c.*1643delT		NM_005475.5	heterozygous
				
RTC116 NR	c.1454_1477del	p.Asp485_Trp492del	NM_005475.2	heterozygous
	c.*1643delT		NM_005475.2	heterozygous
				
RTC143 R	c.784T>C	p.Trp262Arg	NM_005475.2	heterozygous
	c.*1643delT		NM_005475.2	heterozygous
				
RTC127 R	c.784T>C	p.Trp262Arg	NM_005475.2	heterozygous
	c.1236+24_1236+28delTGGGG		NM_005475.2	heterozygous
	c.*1553A>G		NM_005475.2	heterozygous
	c.*1643delT		NM_005475.2	heterozygous
				
RTC115 R	c.*1643delT		NM_005475.2	heterozygous
				
RTC114 R	c.784T>C	p.Trp262Arg	NM_005475.2	homozygous
	c.1236+24_1236+28delTGGGG		NM_005475.2	heterozygous
	c.*1553A>G		NM_005475.2	heterozygous
	c.*1643delT		NM_005475.2	heterozygous
				
RTC110 R	c.784T>C	p.Trp262Arg	NM_005475.2	heterozygous
	c.*1643delT		NM_005475.2	heterozygous
				
RTC113 R	c.784T>C	p.Trp262Arg	NM_005475.2	homozygous
	c.1236+24_1236+28delTGGGG		NM_005475.2	heterozygous
	c.*1553A>G		NM_005475.2	heterozygous
	c.*1643delT		NM_005475.2	heterozygous
				
RTC119 NR	c.17T>C	p.Leu6Pro	NM_001291424.1	heterozygous
	c.*1643delT		NM_005475.2	heterozygous
				
RTC136 R	c.784T>C	p.Trp262Arg	NM_005475.2	homozygous
	c.*1643delT		NM_005475.2	heterozygous
				
RTC134 NR	c.232G>A	p.Glu78Lys	NM_005475.2	heterozygous
	c.784T>C	p.Trp262Arg	NM_005475.2	heterozygous
	c.*1643delT		NM_005475.2	heterozygous
				
RTC130 R	c.557G>T	p.Ser186Ile	NM_005475.2	heterozygous
	c.784T>C	p.Trp262Arg	NM_005475.2	homozygous
	c.1236+24_1236+28delTGGGG		NM_005475.2	heterozygous
	c.*1553A>G		NM_005475.2	heterozygous
	c.*1643delT		NM_005475.2	heterozygous
				
RTC132 NR	c.*1643delT		NM_005475.2	heterozygous
				
RTC131 R	c.784T>C	p.Trp262Arg	NM_005475.2	homozygous
	c.*1643delT		NM_005475.2	heterozygous
				
				

### 3.2 B: Experimental phenotype and genotype validation of cardiac regeneration response switch

#### 3.2.1 Clonal advantage of *SH2B3*/LNK knockout HSC in an experimental competitive transplantation assay

**Fig. 5:**
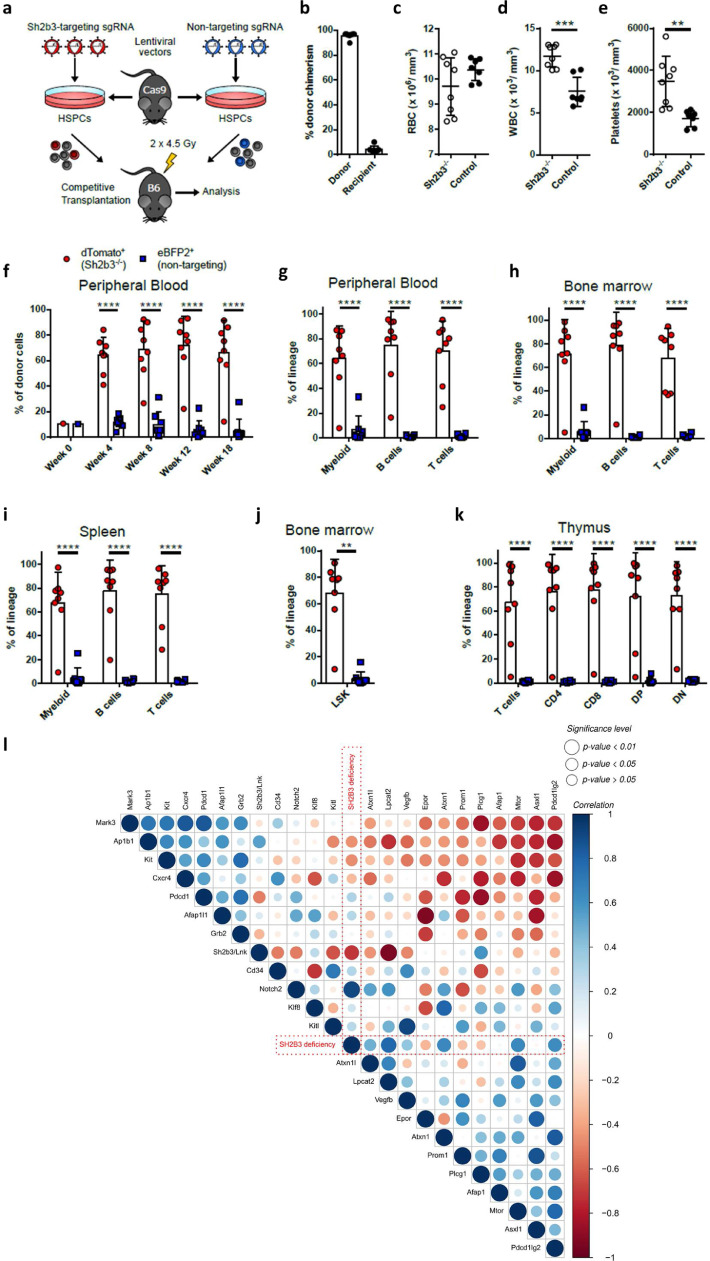
Influence of SH2B3 on HSC clonal overgrowth by using competitive bone marrow transplantation of Sh2b3^−/−^ HSPCs. **a**: Scheme of the competitive transplantation assay is shown. HSPCs, which are derived from a SpCas9 transgenic mouse model (GFP^+^), were transduced with a lentiviral vector carrying a sgRNA against Sh2b3 and a dTomato fluorescent reporter. As competitor cells, HSPCs were transduced with a non-targeting sgRNA and an eBFP2 fluorescent reporter. After transduction, the Sh2b3^−/−^ and Sh2b3-intact competitor cells were transplanted in a 1:1 mixture into irradiated C57BL/6 (B6, GFP^−^) recipient mice. Irradiation was performed using a fractionated dose of 2 × 4^.^5 Gy. **b**: Percentage of donor (GFP^+^) and recipient (GFP^−^) cells of total CD45^+^ cells in the bone marrow of mice at week 18 after transplantation. **c**: Red blood cell (RBC) count in Sh2b3^−/−^ transplanted mice and untreated control animals at week 18 after transplantation. **d**: White blood cell (WBC) count in Sh2b3^−/−^ transplanted mice and untreated control animals at week 18 after transplantation. **e**: Platelet count in Sh2b3^−/−^ transplanted mice and untreated control animals at week 18 after transplantation. **f**: Presence of Sh2b3^−/-^ (dTomato^+^) and competitor (eBFP2^+^) cells in the donor cell population in the peripheral blood at week 4, 8, 12, and 18 after transplantation. Week 0 shows the presence of dTomato^+^ and eBFP2^+^ cells in the transplanted cell population. **g**-**k**: Presence of Sh2b3^−/-^ (dTomato^+^) and Sh2b3-intact competitor (eBFP2^+^) cells in the indicated lineage of donor cells in the peripheral blood g:, in the bone marrow **h**:, in the spleen **i**:, in Lineage^−^ Sca1^+^ cKIT^+^ (LSK) HSPCs of the bone marrow **j**:, and in T cells of the thymus **k**: at week 18 after transplantation. **l**: Pearson correlation analysis of RNA-Seq data derived from murine Sh2b3 HSC clonal overgrowth model. The Sh2b3 deficiency (red) is highlighted for an improved visual analysis of important correlations. The color scale, ranging from *1* to *-1* in the upper panel (blue to red), represents the correlation between the different factors. The size of the dots represents the significance (*p<*0^.^01, *p<*0^.^05, and p>0^.^05, Pearson correlation) of the respective correlation. Transplanted mice: *n*=8. Control mice: *n*=7. All graphs represent mean ± SD. Statistics: **c**-**e**: Unpaired t-test after normality test (D'Agostino & Pearson omnibus normality test) was passed; **f**-**i**:, **k**: Two-way ANOVA. **j**: Kolmogorov-Smirnov test. Significance level: ** *p<*0^.^ 01, *** *p<*0^.^001, and **** *p<*0^.^0001 (For interpretation of the references to color in this figure legend, the reader is referred to the web version of this article.).

To show that *SH2B3*/LNK-deficient hematopoietic stem and progenitor cells (HSPCs) have an advantage to repopulate the bone marrow after partial bone marrow ablation, we performed a competitive transplantation assay. CRISPR-Cas9-mediated *SH2B3*^−/-^ knockout cells (labeled with a dTomato fluorescent reporter) competed against eBFP2-marked *SH2B3*-intact competitor cells transduced with a non-targeting sgRNA (Fig. 5A). At week 18 post-transplantation, the bone marrow of transplanted mice consisted of 95•7% ± 2•4% donor cells, which indicated that despite partial bone marrow ablation, the remaining recipient cells were displaced by donor cells (Fig. 5B). Blood counts of transplanted mice revealed a normal red blood cell count compared to untreated control mice (Fig. 5C). However, we observed a significant increase of white blood cells and platelets in *SH2B3*^−/−^ transplanted mice in comparison to control animals (Fig. 5D, E). Already four weeks after transplantation, the donor cells were dominant with 64•3% ± 13•8% by dTomato^+^ *SH2B3*^−/-^ cells, which remained stable over time (Fig. 5F). Moreover, dTomato^+^ *SH2B3*^−/−^ cells predominantly contributed to the hematopoiesis and outcompeted eBFP2^+^ competitor cells in myeloid, B-cell, and T-cell formation as quantified in the peripheral blood (Fig. 5G). Similar results were obtained for the different lineages (myeloid^CD11b+^, B^B220+^ and T^CD3+^ cells) in the bone marrow and spleen (Fig. 5H, I). Even 68•2% ± 25•5% of Lineage Sca1^+^ cKIT^+^ (LSK) HSPCs in the bone marrow were dTomato^+^ *SH2B3*^−/−^ cells, which revealed a repopulating advantage also on the stem cell level (Fig. 5J). The selective advantage of *SH2B3*^−/−^ was also present in the T cell compartment, including CD4^+^ and CD8^+^ single-positive, double-positive, and double-negative T cells in the thymus of transplanted mice (Fig. 5K). To compare gene expression profiles between WT and *SH2B3*^−/−^ cells, we performed an in-depth gene expression analysis. In order to study transcriptome profile patterns of *SH2B3*^−/-^ and WT peripheral blood signatures, the capture of polyadenylated RNA by high throughput sequencing was applied. To ensure a high comparability to the human samples, the cDNA derived from Globin messengers was also depleted. Correlation analysis was performed (Supplementary Figure 3; Supplementary Data SD1f) and showed a correlation (*p<*0•01) of *SH2B3*^−/-^ with *LPCAT2, NOTCH2, PDCD1LG2*/PD-1, *PROM1*/CD133, *ATXN1*, and *MTOR. SH2B3*^+/+^ was negatively correlated to gene expression of *LPCAT2, KITL, PDCD1*/PD-1 and positively correlated to *PLCG1* and *AP1B1* (Fig. 5L). Affected pathways detected by GSEA include the Kit receptor, EGFR1, and IL-2 signaling pathways (*p<*0•05).

To elucidate the effect of *SH2B3*/LNK-deficiency on previously observed HSC/EPC kinetics in the setting of a mouse MI model, we used a second mouse model and examined the frequency of the kit^+^/sca^+^/lin^−^ HSC (KSL) population.. Interestingly, the KSL population was significantly increased in *SH2B3*/LNK^−/−^ mice compared to WT mice, i.e. on day 1, 3, 7, 14, and 28 (day 7: WT, 12•9 ± 3•7 *vs. SH2B3*/LNK^−/−^, 22•9±3•1 %, *p<*0•01; day 14: WT, 10•1±1•0 *vs. SH2B3*/LNK^−/−^, 22•9±4•8 %, *p<*0•01 two-way ANOVA followed by Tukey's multiple comparisons test) (Fig. 6A, Supplemental Data SD2). To examine the effect of *SH2B3*/LNK-deficiency on EPC mobilization into circulation post MI, we performed FACS analysis for Sca-1^+^/ lineage^−^ cells, an EPC-enriched fraction in PB. The number of the cells pre-infarction was similar in *SH2B3*/LNK^−/−^ and WT mice, whereas on days 3 and 14 post infarction, it was significantly greater in *SH2B3*/LNK^−/−^ mice than WT mice (day 3: WT, 25•3±3.0 *vs. SH2B3*/LNK^−/−^, 37•0±9•4 × 10^4^ cells/mL, *p<*0•05; Day 14: WT, 23•5±3•7 *vs. SH2B3*/LNK^−/−^, 56.0±12•4 × 10^4^ cells/mL, *p<*0•001 two-way ANOVA followed by Tukey's multiple comparisons test) (Fig. 6B, Supplemental Data SD2). In isolated KSL cells, the mRNA expression of angiogenic factors (*VEGF-B, FGF-4, HGF* and *Ang-1*), survival factor (*IGF-1*), and stem/progenitor chemokines (*IGF-2* and *SDF-1*) was significantly up-regulated in *SH2B3*/LNK^−/−^ mice compared to WT mice (Fig. 6C; Supplemental Data SD2). Recruitment of BM-derived EPCs to infarcted myocardium was significantly increased in *SH2B3*/LNK^−/-^ mice *vs*. WT mice (WT, 87•1±74•9 *vs. SH2B3*/LNK^−/−^, 647•1±174•7/mm^2^, *p<*0•001, Mann-Whitney comparison test) (Fig. 6D, Supplemental Data SD2). Moreover, incorporation of BM-derived EPCs into capillary vessels was significantly increased in *SH2B3*/LNK^−/-^ EPCs than WT EPCs even at day 28 post MI (WT, 13•8±9•6 *vs. SH2B3*/LNK^−/−^, 92•0±27•0/mm^2^, *p<*0•001, Mann-Whitney comparison test). Frequency of c-KIT^+^ cells in whole heart analyzed by FACS was greater in *SH2B3*/LNK^−/−^ than WT mice (WT, 3•2±0•7 *vs. SH2B3*/LNK^−/−^, 5•2±0•6 %, *p<*0•05, Mann-Whitney comparison test) (Fig. 6E, Supplemental Data SD2).

**Fig. 6:**
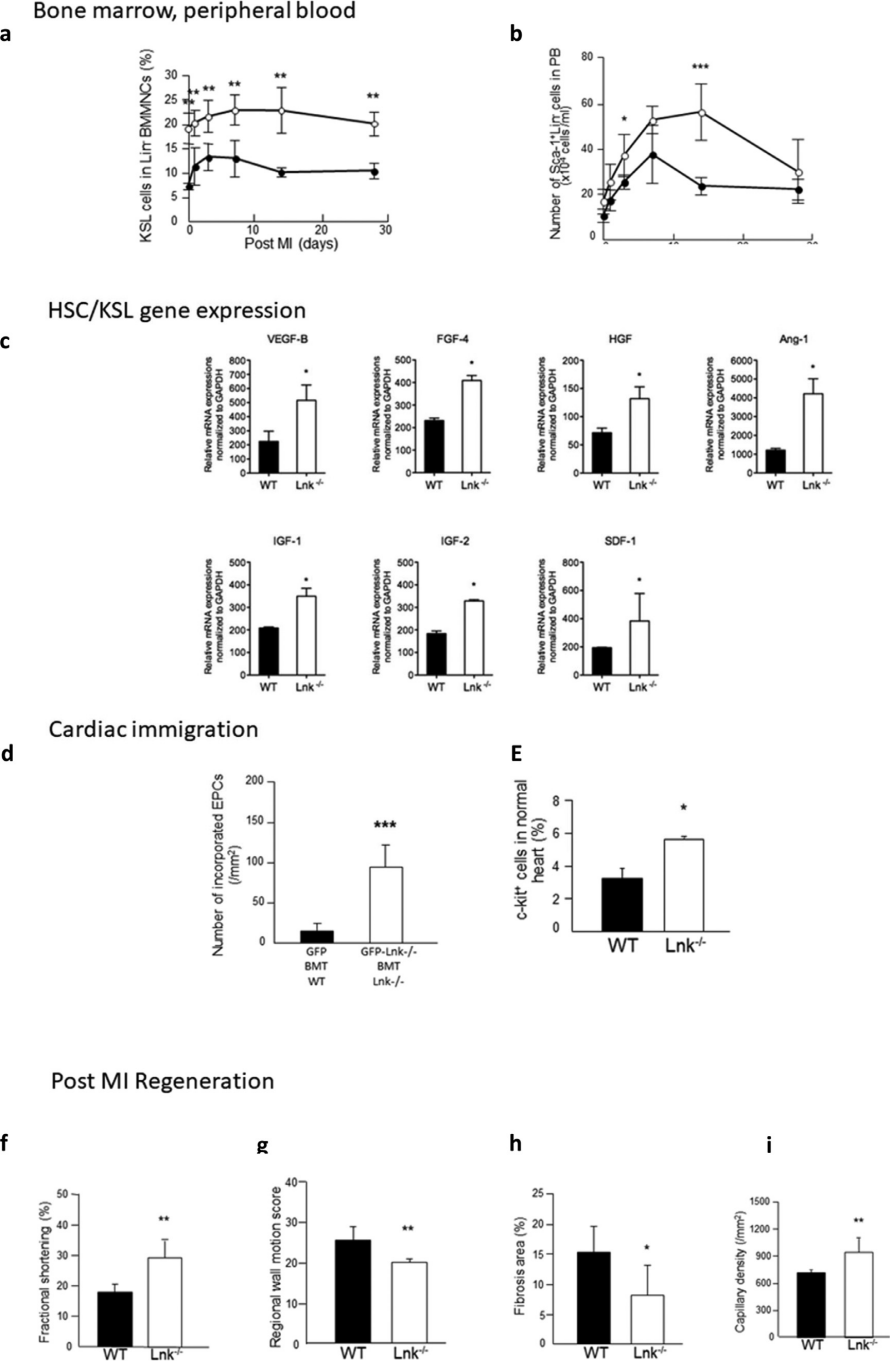
Experimental SH2B3 mouse MI-model Bone marrow, peripheral blood kinetics of KSL cells in BM and SL cells in PB in WT *vs*. SH2B3/LNK^−/-^ mice following MI. SH2B3/LNK^−/−^ leads to increased EPC in bone marrow and circulation post MI **a**: Percent of KSL cells in Lin^−^ BMMNCs before and after MI significantly increased in SH2B3/LNK^−/−^ mice (open circles) compared with WT mice (closed circles). Two-way ANOVA followed by Tukey's multiple comparisons test **, *p<*0^.^01 *vs*. WT (*n=*3-4). **b**: Number of Sca-1^+^/Lin^−^ (SL) cells in PB in SH2B3/LNK^−/−^ mice (open circles) and WT mice (closed circles) before (Pre) and one day, 3, 7, 14, and 28 days after MI. Two-way ANOVA followed by Tukey's multiple comparisons test *, *p<*0^.^05 and ***, *p<*0^.^01 *vs*. WT (*n=*3-4). **c**: HSC/KSL gene expression - Growth Factor and Chemokine mRNA expressions in WT BM-KSL cells *vs*. SH2B3/LNK^−/−^ BM-KSL cells. KSL cells were sorted from freshly isolated BMMNCs by FACS, and were analysed the expressions of VEGF-B, FGF-4, HGF, Ang-1, IGF-1, IGF-2, and SDF-1 by quantitative real-time RT-PCR. Each relative mRNA expression was normalized to GAPDH and compared between WT BM-KSL cells (solid bar) and SH2B3/LNK^−/−^ BM-KSL cells (open bar). Bonferroni post hoc test *, *p<*0^.^05. (*n=*3). **d**: Effect of SH2B3/LNK gene deficiency on recruitment of BM-derived progenitors to ischemic myocardium. d: Double fluorescent immunostaining for GFP (green) and isolectin B4 (red) in heart sections in WT mice transplanted with GFP^+^ BM and in SH2B3/LNK^−/−^ mice transplanted with GFP^+^- SH2B3/LNK^−/−^ BM 7 days following MI. Number of recruited BM-derived cells into vasculature in ischemic myocardium 28 days following MI were counted and averaged. Mann-Whitney comparison test **, *p<*0^.^01 and ***, *p<*0^.^001 *vs*. WT mice transplanted with GFP BM. (*n=*3). **e**: Assessment for proliferation activity in CSCs/CPCs and cardiomyocytes in ischemic myocardium. Double fluorescent immunostaining for BrdU (red) and c-KIT (green) in heart sections in WT mice and in SH2B3/LNK^−/−^ mice 7 days following MI. Number of BrdU^+^/c-KIT^+^ cells in ischemic myocardium 7 days following MI were counted and averaged. Mann-Whitney comparison test *, *p<*0^.^05 *vs.* WT mice (WT: *n=*4 and SH2B3/LNK^−/−^: *n=*3). **f**-**i**: Post MI regeneration: physiological and histological assessment for LV function in WT *vs*. SH2B3/LNK^−/-^ mice following MI. M-mode echocardiography in WT mice and SH2B3/LNK^−/−^ mice 28 days following MI. Fractional shortening (**f**) and regional wall motion score (**g**) were significantly great in SH2B3/LNK^−/−^ mice than that in WT mice. Hemodynamic study using a micro-tip catheter in WT mice and SH2B3/LNK^−/−^ mice 28 days following MI. +dP/dt, -dP/dt and EDP were significantly preserved in SH2B3/LNK^−/−^ mice than those in WT mice. Mann-Whitney comparison test *, *p<*0^.^05 and **, *p<*0^.^01 *vs.* WT. (*n=*11) (+dP/dt: WT, 5,942^.^1±823^.^7 *vs*. SH2B3/LNK^−/−^, 8,901^.^6±1,147^.^9 mmHg/sec, *p<*0^.^01; -dP/dt: WT, -4,675^.^9±615^.^9 *vs*. SH2B3/LNK^−/−^, -6,201^.^4±875^.^4 mmHg/sec, *p<*0^.^01; EDP: WT, 8^.^6±2^.^1 *vs*. SH2B3/LNK^−/−^, 4^.^4±1^.^2 mmHg, *p<*0^.^05) (**h**) Representative Masson's trichrome stained heart sections in WT mice and SH2B3/LNK^−/−^ mice 28 days following MI. Percent of fibrosis area in entire LV area on cross-sections. Histological analysis was performed on day 28 post MI. The percentage of fibrosis area was less in SH2B3/LNK^−/−^ mice than WT mice (WT, 15^.^2±4^.^3 *vs*. SH2B3/LNK^−/−^, 8^.^0±5^.^0 %, *p<*0^.^05). Fibrosis area was significantly reduced in SH2B3/LNK^−/−^ mice compared with WT mice. Bonferroni post hoc test * *p<*0,05 *vs*. WT. (WT: *n=*6 and SH2B3/LNK^−/−^: *n=*10) (**i**) Immunostaining for isolectin B4 (brown) in WT and SH2B3/LNK^−/−^ mice 28 days following MI. Capillary density in ischemic border zone in infarcted myocardium of WT mice and SH2B3/LNK^−/−^ mice. Bonferroni post hoc test **, *p<*0^.^01 *vs*. WT. (WT: *n=*5 and SH2B3/LNK^−/−^: *n=*9) capillary density in infarction border zone was significantly greater in SH2B3/LNK^−/−^ mice than WT mice (WT, 713±28 *vs*. SH2B3/LNK^−/−^, 937±157/mm^2^, *p<*0^.^01). On the other hand, there was no significant difference in LV function and capillary density between WT mice and SH2B3/LNK^−/−^ mice without ischemic injury. **j**: Pearson correlation analysis between the mouse infarction model (SH2B3/LNK ^-/−^*vs.* WT) and human phase 3 PERFECT trial (ΔLVEF Responder *vs.* Non-responder). The human ΔLVEF response is highlighted for an improved visual analysis of important correlations. The color scale, ranging from *1* to *-1* in the upper panel (blue to red), represents the correlation between the different factors. The size of the dots represents the significance (*p<*0^.^01, *p<*0^.^05, and p>0^.^05; Pearson correlation) of the respective correlation. Comparison of peripheral blood (PB) circulating cells and biomarkers between mice (purple) and human (serum) (black) (For interpretation of the references to color in this figure legend, the reader is referred to the web version of this article.).

**Fig. 6:**
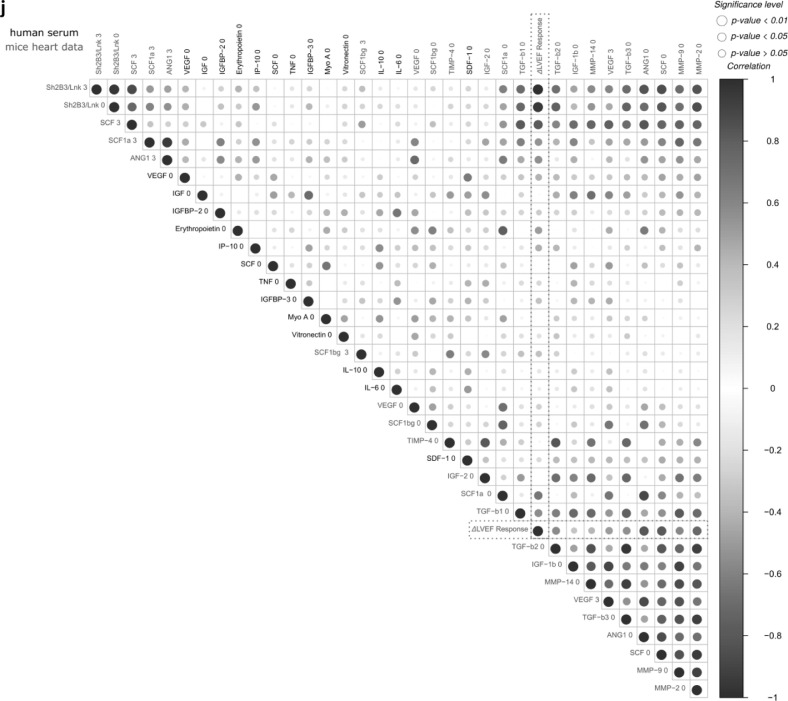
(Continued).

#### 3.2.2 Preserved left ventricular function and structural integrity in infarcted myocardium in *SH2B3*/LNK^−/−^ mice

To investigate the effect on the phenotypic level in mice, LV function of WT mice and *SH2B3*/LNK^−/−^ mice on day 28 post MI was analyzed by echocardiography. Fractional shortening (FS: WT, 17•7±2•6 *vs. SH2B3*/LNK^−/−^, 29•2±5•6 %, *p<*0,01, Mann-Whitney comparison test) (Fig. 6F), a parameter of global left ventricular contractility, and regional wall motion score (RWMS) were examined (RWMS: WT, 25•4±3•4 *vs. SH2B3*/LNK^−/−^, 19•9±0•9, *p<*0•01, Mann-Whitney comparison test) (Fig. 6G, Supplemental Data SD2). LV function was also examined using micromanometer-tipped catheters on day 28. +dP/dt, -dP/dt and endodiastric pressure (EDP), infarction borderzone fibrosis and capillary angiogenesis were significantly better preserved in *SH2B3*/LNK^−/−^ mice (Fig. 6H, I, Supplemental Data SD2). These results suggest that gene deficiency of *SH2B3*/LNK contributes to the preservation of LV function and structural integrity of infarcted myocardium post MI.

### 3.3 C: Evaluation

#### 3.3.1 Bone marrow, peripheral blood, and heart tissue data in mice and man

We compared the previously investigated datasets of mice and man using correlation analyses visualised as heatmaps with respect to comparability of *SH2B3*/LNK^−/−^ *vs* WT post MI cardiac regeneration biomarkers to post CABG/CD133^+^ cardiac regeneration (R*vs*NR) (JFig. 6J). Circulating EPCs in PB were correlated (*p<*0•05) to ∆LVEF response (R*vs*NR and *SH2B3*/LNK^−/−^ *vs*. WT) for huCD34^+^(day 0), huCD45^+^14^+^(day 0), huCD133^+^(day 0), huCD45^+^CD184(CXCR4)^+^(day 0), huCD117(c-KIT)^+^(day 0), mSca-1^+^(day 3, 7), and mCD184(CXCR4)^+^(day 0, 14, 28) (Supplementary Figure S4). KSL bone marrow SC (day 1, 7, 14) in mice were positively correlated (*p<*0•05) to human PB CD133^+^ EPC, CFU-Hill, and ∆LVEF-response (Supplementary Figure S5). ∆LVEF-response was negatively correlated (*p<*0•01) to cardiac *SH2B3*/LNK expression in mice (day 0, 3) and positively correlated (*p<*0•05) to mouse cardiac remodeling factors MMP2, SCF1, and Angiopoietin 1 on day 0 (JFig. 6J).

#### 3.3.2 ML- integrated stratification of patients

**Fig. 7:**
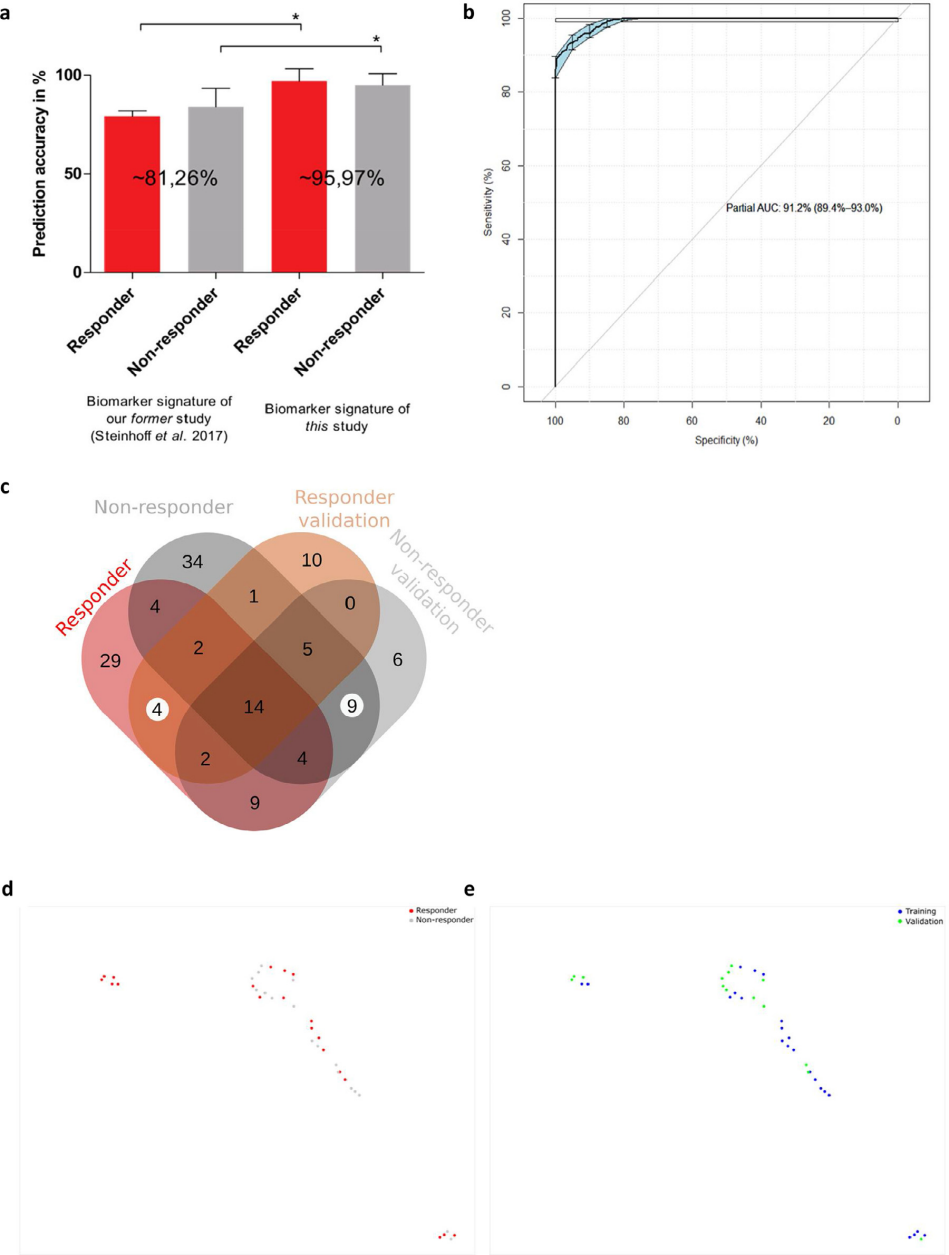
Patient stratification to responder and non-responder. Clustering and SNP signature comparison for the analysis and validation cohort. **a:** Machine learning accuracy comparison for the supervised prediction of the patient responsiveness using only preoperative data. Results are obtained after feature selection and subsequent prediction with two independent classifiers. The graph shows the true positive prediction results of two ML models (AdaBoost for feature selection and RF for final prediction for the former study and RF and SVM for the current study).The error bars indicate the respective accuracy standard deviation for the constructed models that have been obtained after 100 iterations. * indicates that the 100 model iterations are significant different according to Bonferroni post-hoc test (*p<*0.01). **b**: Receiver Operating Characteristics (ROC) curve for the random forest machine learning model. The plot represents the sensitivity (true positive rate) and the specificity (false positive rate) of the model. The area under the ROC curve (AUC) represents the entire area underneath the ROC curve and the confidence intervals (95%CI) are indicated in blue. **c**: Venn diagram summarizing the identified SNPs in R*vs*NR for the analysis and validation cohort. **d**-**e**: Validation for the clustering with primary cohort (*n=*23, blue, Rostock trial center biomarker cohort) and independent validation cohort (*n=*14, green, Hannover center). UMAP representation with k=4 and 2,000 epochs (For interpretation of the references to color in this figure legend, the reader is referred to the web version of this article.).

Combining gene expression features and biomarkers led to an improved patient signature for outcome prediction from 81% [17] to finally 96%, which was achieved by only using the top eight ML-selected features (Fig. 7A). Pathway analysis of most important ML features focusses on genes regulating hematopoietic stem cell receptor and proliferation signaling (Table 2).

R/NR selective discriminative factors were identified by combining gene expression difference, coexpression analysis, SNP/variants, and Machine Learning (Fig. 7B; Supplementary Fig. S6). Validation of an independent patient data set revealed 85% accuracy (12/14 patients) for stratification (Fig. 7C) and classification concordance to ML-clusters (Fig. 7D, E). All missclassified patients in the primary biomarker cohort analysis (1/23, male R) and the validation cohort (2/14; male/R and female/NR) exclusively showed a single SNP (rs.56313931 G/A) mutation in the X-chromosome linked target biomarker KLF-8 (3/37) (Supplementary Data SD1D).

## 4 Discussion

In the randomized PERFECT phase 3 CABG/CD133^+^ stem cell trial, we observed a preoperative signature of circulating BM-HSC/EPC and angiogenesis parameters in peripheral blood that significantly correlated to postoperative response of myocardial regeneration in contrast to no difference in surgical CABG-procedure with complete coronary revascularization [17]. Using RNA sequencing analysis in blood, we have found for the first time a corresponding signature for a decreased (NR) or enhanced (R) bone marrow stem cell response upon ischemic/inflammatory response to CABG. Gene expression patterns are characteristic for a R*vs*NR preoperative steady-state and include pathways for proliferation control (EGFR, PDFR, TCR, c-KIT), inflammation (IL2, IFN1), and platelet activating factor synthesis (LPCAT2). Identified hub genes related to BM-HSC response were mainly genes coding for signaling, adaptor, and transcription regulation proteins.

Our independent ML-based feature selection of R*vs*NR discriminating the most important factors gives evidence for a gene circuit effecting myocardial repair by modified expression of signaling and adaptor protein gene transcription. Given the complexity of stem cell response to ischemic or inflammatory stimuli as induced by CABG-surgery, we found access to circuit pattern recognition in patients only by unbiased ML methods. Learning from failed reductionistic attempts to define single control factors of stem cell reactivity, we propose this approach as an independent and unbiased start. The association of genes such as *PLCG1, LPCAT2, AP1B1, AFAP1, GRB2, KLF8, MARK3*, with circulating CD133^+^/CD34^+^ cells, and serum proteins EPO/VEGF were initially identified using ML and sequentially validated experimentally in mice and in an additional patient cohort. Further clustering analysis of the overall patient cohort revealed subgroups representing the complexity of gene expression, coexpression, and mutational variants. Their functional interplay has to be studied in additional experimental models as exemplarily shown here for the Src adaptor protein *SH2B3*/LNK that regulates crosstalk between integrin and cytokine signaling pathways in BM-HSC/megakaryocyte proliferation.

Moreover, for the first time multiple somatic mutations involving stem cell functional genes as *NOTCH2, PROM1*/CD133, *MTOR* as well as *SH2B3* gene were found in PERFECT patients with coronary arteriosclerotic disease. In this context, mutations of *SH2B3*/LNK were present in all patients with a majority of responders expressing the exon variant RS3184504 that is associated to coronary artery disease and increased thrombocyte count. Interestingly, human *SH2B3* gene expression was not linked to thrombocyte count and circulating CD133^+^/CD34^+^ BM-HSC, whereas correlation was found to LVEF response. It is conceivable, that not reduced gene expression, but mutant related altered *SH2B3*/LNK protein signaling function impairs control of stem cell proliferation. Therefore, we compared gene expression patterns in a mouse model of CRISPR-Cas9-mediated *SH2B3* knockout with human R/NR.

Similar to *SH2B3*/LNK in humans, LNK in the mouse model is an adaptor protein that negatively regulates multiple essential signals, including the SCF/c-KIT system, in stem/progenitor cells. We have previously reported that the deficiency accelerated hindlimb ischemia recovery and bone fracture healing [1], [24] is mainly achieved by restoring local blood perfusion with increased angiogenesis and osteogenesis, respectively. Following the series of *SH2B3*/LNK-related clinical observations in R/NR, we successfully demonstrated for the first time clonal dominance in hematopoiesis, lymphopoiesis, and myelopoiesis by reduced *SH2B3*/LNK signaling. To test the hypothesis of a clonal network switch found *in silico,* we subsequently performed a competitive syngeneic bone marrow transplantation model in mice transplanted with unmodified and *SH2B3*/LNK knockout HSC. *SH2B3*/LNK knockout HSC clones displayed significant overgrowth of myeloid and immune cells in bone marrow, peripheral blood, and tissue at day 160 after BMT. Moreover, the gene expression profile of peripheral blood was similar to the human R/NR signature (Table 4).

**Table 4:** Comparison of whole blood RNA-Seq gene expression in human R*vs*NR in PERFECT trial in comparison to CRISPcas SH2B3 knock-out bone marrow transplantation (BMT) mouse model. Selected correlating genes to SH2B3 in PERFECT trial R*vs*NR and SH2B3 CRISPcas HSC knock-out mouse model. Depicted are RNA-Seq analyzed gene expression levels of selected genes with baseline PB expression in PERFECT R*vs*NR as compared to mouse CRISPcas SH2B3 knock-out BMT model. Identical pattern was observed for NOTCH2, PLCG1, LPCAT2, Prom1/CD133, MTOR, whereas VEGF-B was differently regulated.

SH2B3/LNK correlating genes				RvsNR expression human PERFECT trial		SH2B3 neg. *vs* pos. mouse BMT model	
Abbreviation	Compartment	Protein function	SH2B3 GE correlation	Responder Delta LVEF	Non Responder dLVEF	KO/CRISP CAS Mouse SH2B3 deficient	WT Mouse SH2B3+
NOTCH2	Nucleus, ER, golgi, extracellular	Proliferation, differentiation, apoptosis	*P<*0.01 Pos	↑	↓	↑	↓
PLCG1 (RNA)	Cytosol, Nucleus	Signal transduction TK growth receptors	*P<*0.01 Pos	↑	↓	↑	↓
PDCD1/PD-1 (RNA)	Cytosol, extracellular	Immune checkpoint receptor ligand, apoptosis control	*P<*0.05 Pos	↑	↓	↑	↓
LPCAT2 (RNA)	ER, Golgi	Phospholipid metabolism, PAF synthesis	P>0.05 Pos	↑	↓	↑	↓
Prom1/CD133 (RNA)	ER, Membrane	Differentiation, proliferation, apoptosis signaling	*P<*0.05	↑	↓	↑	↓
MTOR (RNA)	Lysosome, Cytosol	Proliferation, metabolism signaling	*P<*0.05 Pos	↑	↓	↑	↓
VEGF-B (RNA)	extracellular	Vascular growth factor	*P<*0.05 Neg	↓	↑	↑	↓

Furthermore, in ischemic myocardium using a *SH2B3*/LNK^−/−^ mouse MI model, we demonstrated that: 1) *SH2B3*/LNK deficiency increased the number of HSC/KSL stem/progenitor cells (including EPCs) in BM and stem/progenitor cells in myocardium, 2) Angiogenic growth factor, survival factor, and stem/progenitor chemokine mRNA expressions were up-regulated in *SH2B3*/LNK deficient BM/PB HSC stem/progenitor cells, 3) Significant mobilization of BM/PB stem/progenitor cells occurred following myocardial ischemia in *SH2B3*/LNK-negative mice, 4) *SH2B3*/LNK deficiency reduced myocardial ischemic insult with increased angiogenesis to recruit BM-derived EPCs, 5) Resident stem/precursor cells in the heart proliferated and contributed to tissue regeneration in ischemic myocardium following MI.

Taken together, the obtained transcriptome data reveal that clonal HSC dysregulation led to specific disease phenotypes as reduced angiogenesis and sustained myocardial ischemia were observed to regulate cardiac recovery outcome in PERFECT trial coronary artery disease patients. Moreover, not specifically CHIP-gene mutations, but distinct regulatory angiogenesis and HSC proliferation pathway gene mutations were observed [5]. These observations underline the hypothesis of coronary artery disease (CAD) as a phenotype of hematological stem cell disease and acquired senescence by somatic mutations in the tissue repair gene circuit [4]. From this knowledge, treatment strategies for CAD with stem cells have to be reassessed [9,25]. We have to reconsider the current approach to diagnose and treat hematopoietic stem cell dysfunction as a truly cardiovascular and immune stem cell disease [9]. On this basis, temporary *SH2B3* gene downregulation or functional abrogation of LNK protein would be one of the therapeutically effective modifications in currently ongoing autologous progenitor cell transplantation therapy [26]. Moreover, modulation of *SH2B3* mutant gene expression in proliferative arteriosclerotic or hypoproliferative ischemic cardiovascular diseases may give rise to the next generation cell based therapy [27]. Temporary downregulation of adaptor gene *SH2B3* in cKIT-CD117^+^/CD133^+^/CD34^+^ HSC can be a therapeutic switch to improve downregulated stem cell response in ischemia, tissue repair, and myocardial infarction. Using the R/NR signature response described here may also guide specific immune checkpoint or interferon based drug treatment interventions.

At this stage it is speculative, but it may be assumed that the myocardial repair response or non-response to stem cell therapy of coronary artery disease patients could be predicted on the basis of the gene expression signatures found in the PERFECT trial patients. This approach may complement the current evaluation in the CardiAMP Heart Failure Trial to predict treatment response from colony forming capacity of bone marrow stem cells [28]. Next phase studies should test the validity of our integrated data analysis approach combining whole transriptome, protein expression, cell function (colony forming units/CFU or Boyden chamber migration assay) with clinical MRI-imaging, laboratory, and symptom data for ML-based clinical outcome prediction accuracy. This may enable treatment of acquired stem cell senescence in a presymptomatic state and aim for restoration of normal bone marrow function. For selection and clonal overgrowth of healthy autologous HSC using ressources like cord blood HSC banking, iPSC-technology of non-mutated cell types as cardiomyocytes for HSC generation can be a desired option. Clonal selection and expansion biotechnology or allogenic BMT can be used to treat early or advanced stages of HSC senescence. In cases of advanced HSC senescence, the positive experience of autologous/allogenic BMT in multiple myeloma patients may be followed [29].

In conclusion, the proposed ML biomarker/gene signature, resulting in 96% classification accuracy, opens a perspective for the analysis of polygenic risk and cardiovascular disease pathomechanism profiling [30]. The first use of an integrative algorithm for gene expression, hub gene coexpression, and transcribed RNA variants derived from RNA-Seq datasets allows identification of patient-specific perturbations. This leads to an individualized pathomechanism switch and targeted treatment as shown for *SH2B3*/LNK. Moreover, validation of misclassifications can be enabled by whole genome variant analysis as shown here for the X-chromosome linked target gene KLF8. ML-based diagnosis of stem cell based *cardiac regeneration capacity* in tissue ischemia, infection or vascular repair can be applied and tested for differential diagnosis in heart and degenerative organ disease.

## Role of the funding

The funding had no role in study design, in the collection, analysis, interpretation of data, in the writing of the report, and in the decision to submit the manuscript for publication. The corresponding author had full access to all the data in the study and had final responsibility for the decision to submit for publication.

## Declaration of Competing Interest

All authors declare no competing interests.

## Contributors

G Steinhoff contributed to study design, trial organization, medical controlling, enrolment and clinical follow-up of patients, research plan, analysis of clinical data, analysis of research data, data collection, data control, data analysis, and drafted the manuscript.

A Haverich, J Garbade, C Stamm, J Gummert , F M Wagner contributed to enrolment and clinical follow-up of patients, data collection and interpretation.

H Blum, S Krebs, J Philippou-Massier performed tissue processing and gene sequencing analysis, construction of cDNA libraries from polyA RNA with depletion of Globin cDNA and generation of sequencing data.

J Kowallick, CO Ritter, E Schrinner analysed MRI data.

J Nesteruk contributed to laboratory study design, follow-up analysis of patients, data collection, and statistical analyses.

R Gaebel performed laboratory analysis and examined data collection.

M Wolfien, H Hennig and O Wolkenhauer applied and investigated gene expression and machine learning data analysis.

T Asahara contributed to study design, experimental trial organization, research plan, analysis of experimental research data, data collection, data control, and drafted the manuscript.

A Kawamoto contributed to laboratory study design, data collection, and statistical analyses.

A Salybekov, M Ii, M Horii, H Iwasaki, H Akimaru, E Akimaru, A Yokoyama performed experimental animal model, laboratory analysis and examined data collection.

D Klatt and A Schambach performed experimental gene therapy, gene knock outs, competitive bone marrow transplantation model, and drafted the manuscript.

All authors contributed to final data interpretation, critically revised the manuscript, and approved the final version for submission.

## Appendix Supplementary materials

Image, application 1

Image, application 2

Image, application 3

Image, application 4

Image, application 5

Image, application 6

Image, application 7

Image, application 8

Image, application 9

Image, application 10

Image, application 11

Image, application 12

Image, application 13
