# Supplementary material for: Hematopoietic stem-cell senescence and myocardial repair - Coronary artery disease genotype/phenotype analysis of post-MI myocardial regeneration response induced by CABG/CD133+ bone marrow hematopoietic stem cell treatment in RCT PERFECT Phase 3
Source: eBioMedicine. 2020 Jul 4;57:102862. doi: 10.1016/j.ebiom.2020.102862 (PMC7339012; doi:10.1016/j.ebiom.2020.102862)
Supplement: Supplementary file 7 [file mmc7.pdf]

Supplemental data SD1: RT-PCR gene expression analysis and reference gene variation

Figure SD1a: SH2B3 relative expression (normalized to GAPDH) at time point pre-OP correlated to relative left ventricular ejection fraction (ΔLVEF) relative longitudinal axis strain (ΔLAS) and relative maximum upslope epicardial as well as endocardial at day 180/0 (normalized to pre-OP) for Non-responder and Responder; Mean±SEM; * P≤0.001, ** P=0.024 (t-test); *** P=0.015, **** P=0.038 (Mann-Whitney Rank Sum test) compared to Non-responder

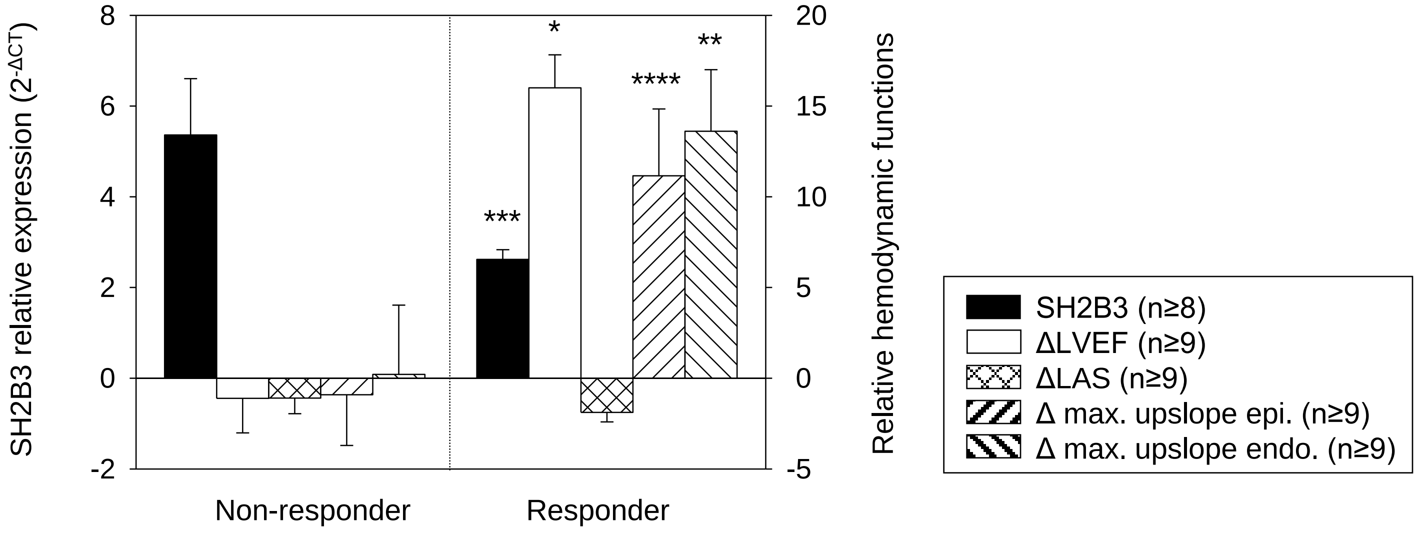

Figure SD1b: Correlation between SH2B3 relative expression (normalized to GAPDH) and circulating mononuclear cells (MNC) in peripheral blood for Non-responder and Responder at time point pre-OP; Mean±SEM; * P=0.037 (t-test); ** P=0.041, *** P=0.015, **** P=0.006 (Mann-Whitney Rank Sum test) compared to Non-responder

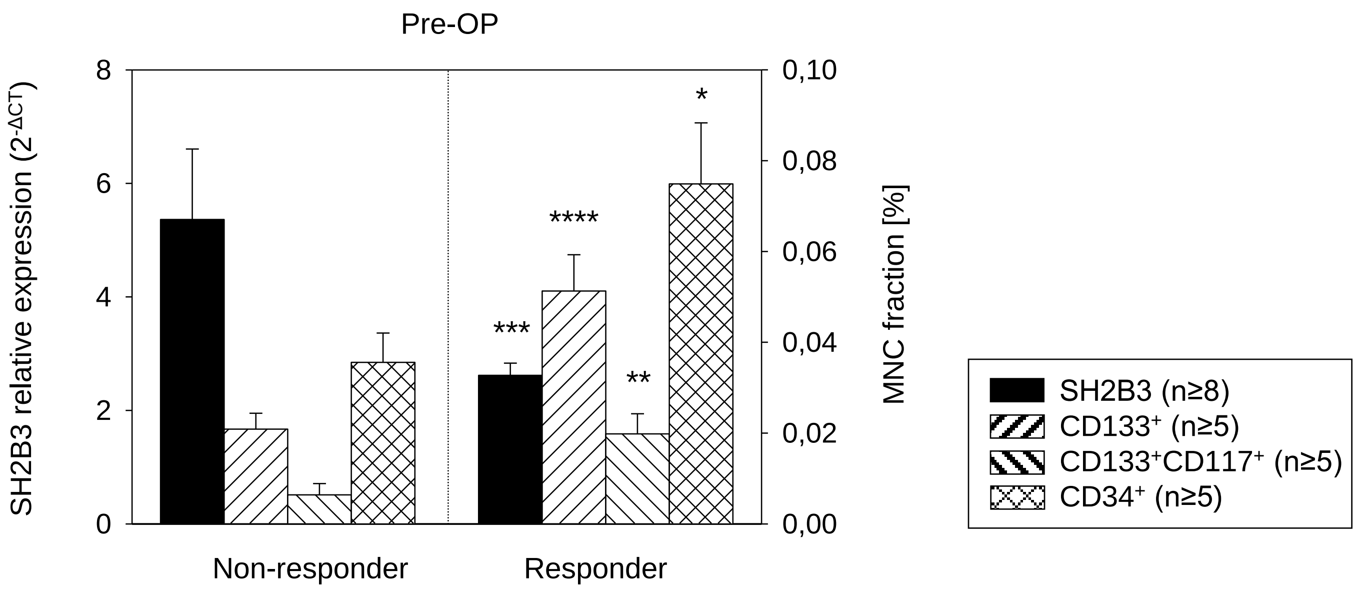

Figure SD1c: SH2B3 relative expression (normalized to POLR2A) at time point pre-OP correlated to relative left ventricular ejection fraction (ΔLVEF) relative longitudinal axis strain (ΔLAS) and relative maximum upslope epicardial as well as endocardial at day 180/0 (normalized to pre-OP) for Non-responder and Responder; Mean±SEM; * P≤0.001, ** P=0.024 (t-test); *** P=0.038 (Mann-Whitney Rank Sum test) compared to Non-responder

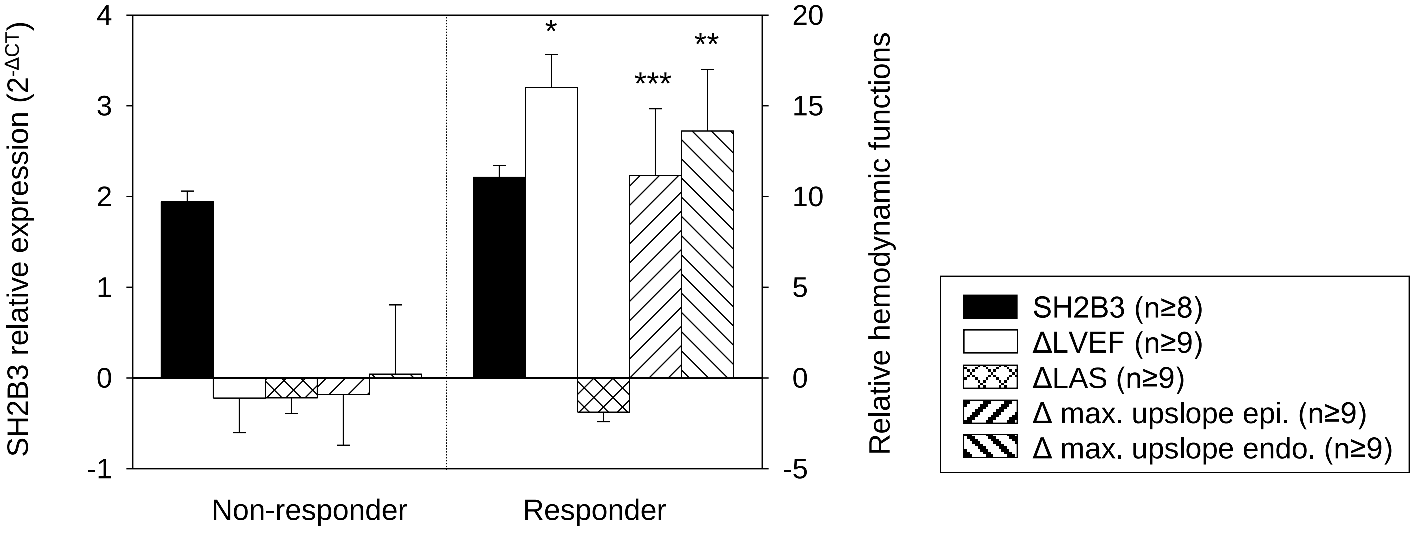

Figure SD1d: Correlation between SH2B3 relative expression (normalized to POLR2A) and circulating mononuclear cells (MNC) in peripheral blood for Non-responder and Responder at time point pre-OP; Mean±SEM; * P=0.037 (t-test); ** P=0.041, *** P=0.006 (Mann-Whitney Rank Sum test) compared to Non-responder

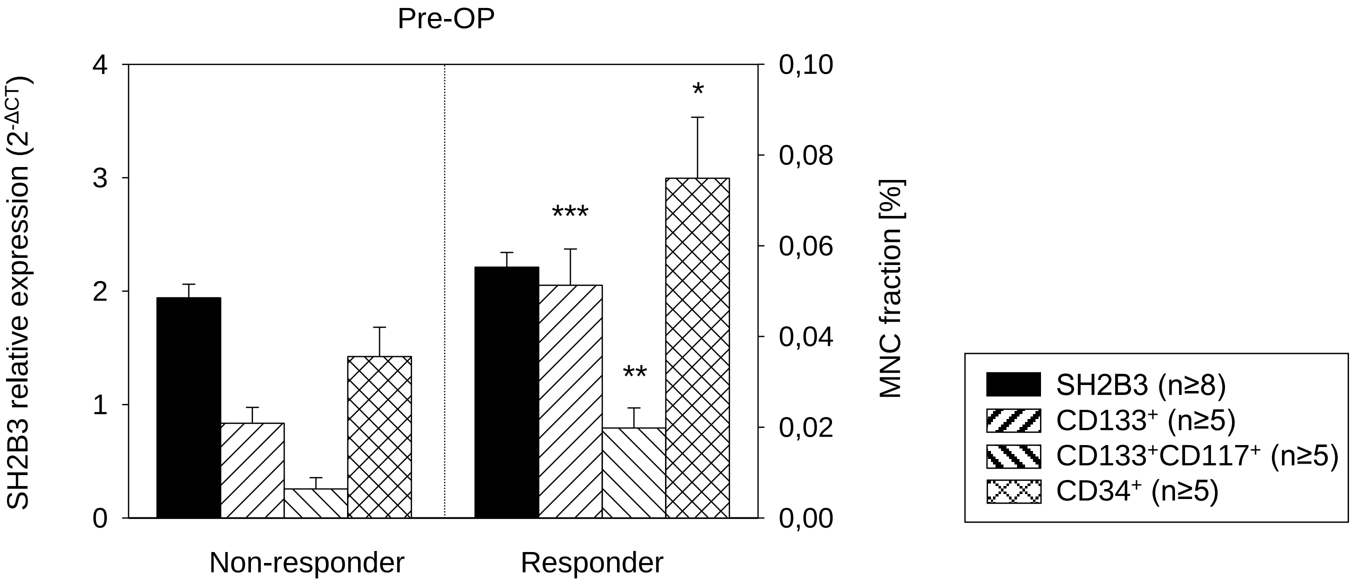

Figure SD1e: SH2B3 relative expression (normalized to GAPDH/POLR2A) at time point pre-OP correlated to relative left ventricular ejection fraction (ΔLVEF) relative longitudinal axis strain (ΔLAS) and relative maximum upslope epicardial as well as endocardial at day 180/0 (normalized to pre-OP) for Non-responder and Responder; Mean±SEM; * P≤0.001, ** P=0.024 (t-test); *** P=0.038 (Mann-Whitney Rank Sum test) compared to Non-responder

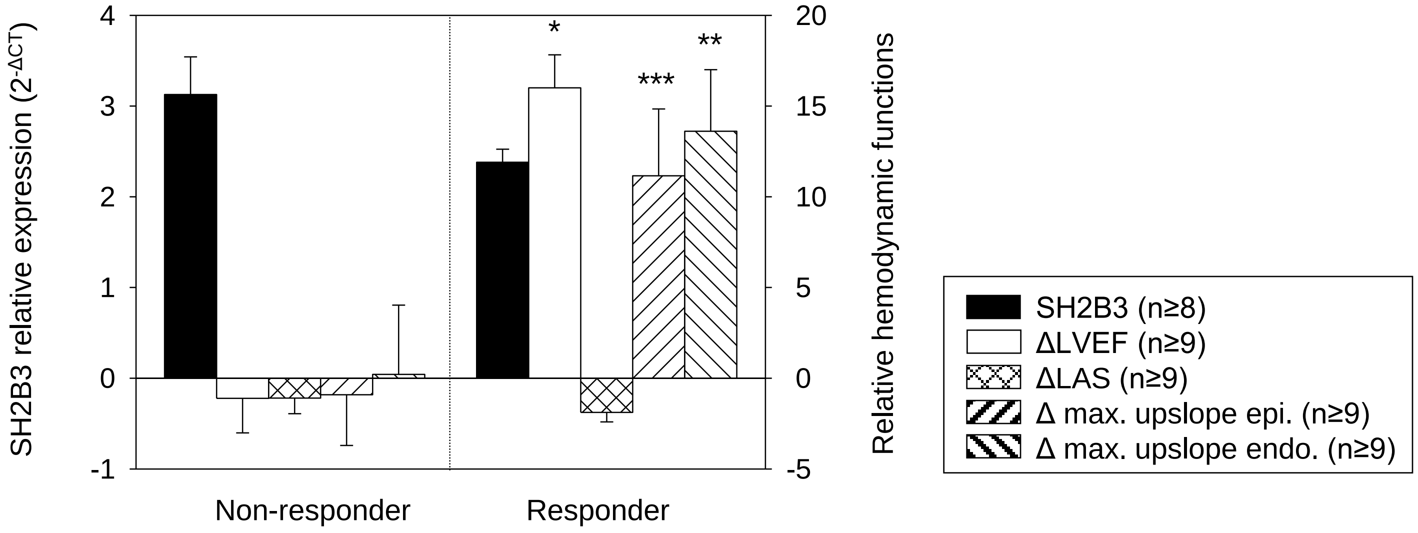

Figure SD1f: Correlation between SH2B3 relative expression (normalized to GAPDH/POLR2A) and circulating mononuclear cells (MNC) in peripheral blood for Non-responder and Responder at time point pre-OP; Mean±SEM; * P=0.037 (t-test); ** P=0.041, *** P=0.006 (Mann-Whitney Rank Sum test) compared to Non-responder

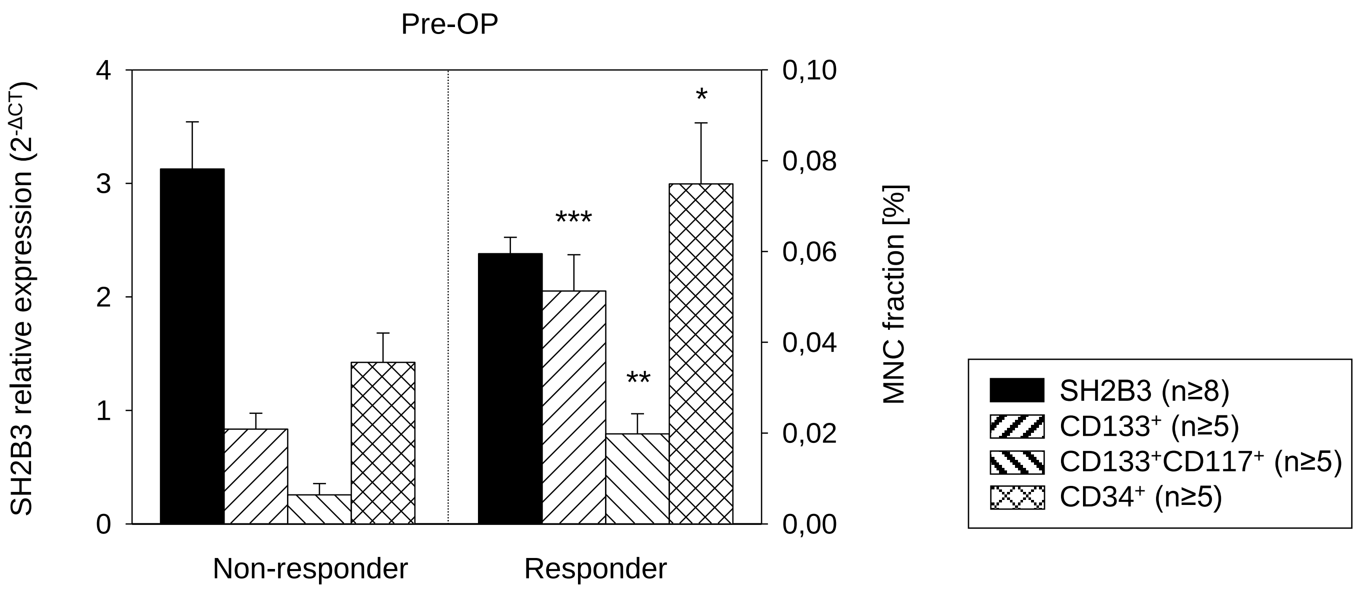

RT-PCR gene expression and RNAseq variant calling GAPDH and POLR2a/RBP1

Non-responder			
	$2^{-\Delta C_T}$ (Gapdh)	$2^{-\Delta C_T}$ (Polr2a)	$2^{-\Delta C_T}$ (both)
RTC099	3,287321565	1,38938763	2,13713919
RTC106	12,50194899	2,290361109	5,351072579
RTC116	8,488799152	2,084140723	4,206168327
RTC119	3,085144871	2,270275981	2,646531749
RTC132	2,180831893	1,891915283	2,031243261
RTC137	4,999938059	1,975344963	3,142706232
RTC140	2,95695642	1,496807647	2,103804882
RTC146	5,409452789	2,133765437	3,397425996

chr3	139240014	A	G	69,92	PASS	3	RTC099<br>RTC137<br>RTC140	intronic	RBP1
chr12	6643927	T	G	44,77	PASS	1	RTC134	intronic	GAPDH
chr12	6643991	C	G	1096,21	PASS	9	RTC099<br>RTC101_0<br>RTC106<br>RTC108_0<br>RTC125_0<br>RTC132_0<br>RTC134<br>RTC135_0<br>RTC137	exonic	GAPDH
chr12	6644256	T	A	146,77	PASS	2	RTC111_0<br>RTC125_0	intronic	GAPDH
chr12	6644696	T	G	99,27	PASS	4	RTC108_0<br>RTC111_0<br>RTC125_0<br>RTC132_0	intronic	GAPDH
chr12	6644722	A	G	93,27	PASS	5	RTC108_0<br>RTC111_0<br>RTC112_0<br>RTC125_0<br>RTC132_0	intronic	GAPDH
chr12	6644781	T	C	124,77	PASS	4	RTC108_0<br>RTC111_0<br>RTC112_0<br>RTC125_0	intronic	GAPDH
chr12	6645565	T	C	77,27	PASS	2	RTC125_0<br>RTC135_0	intronic	GAPDH
chr12	6646299	C	T	107,77	PASS	2	RTC121_0<br>RTC150_0	exonic	GAPDH
chr12	6646320	T	C	1042,27	PASS	8	RTC106<br>RTC108_0<br>RTC111_0<br>RTC125_0<br>RTC132_0<br>RTC134<br>RTC135_0<br>RTC137	exonic	GAPDH
chr12	6647109	T	C	1203,64	PASS	8	RTC106<br>RTC108_0<br>RTC111_0<br>RTC125_0<br>RTC132_0<br>RTC134<br>RTC135_0<br>RTC137	exonic	GAPDH
chr12	6647453	G	T	149,17	PASS	5	RTC111_0<br>RTC125_0<br>RTC132_0<br>RTC134<br>RTC135_0	exonic	GAPDH
chr12	6647531	T	C	29,77	PASS	1	RTC116	exonic	GAPDH

Responder
RT-PCR gene expression and RNAseq variant calling GAPDH and POLR2a/RBP1

Responder	$2^{-\Delta C_T}$ (Gapdh)	$2^{-\Delta C_T}$ (Polr2a)	$2^{-\Delta C_T}$ (both)
RTC110	2,739256391	3,024732333	2,878457464
RTC113	1,205470294	1,877958347	1,504600612
RTC114	2,813165625	2,188873566	2,481464059
RTC115	2,194274357	2,142232953	2,168097515
RTC117	1,832204194	2,71494396	2,230320988
RTC124	2,37027884	2,479223275	2,424139119
RTC127	2,010621437	1,444565713	1,704251974
RTC130	2,177702963	1,701066378	1,924686284
RTC131	2,089402796	2,069360217	2,079357358
RTC133	2,740479523	1,926853786	2,297934582
RTC139	3,534871612	2,146324648	2,754447689
RTC143	3,70166428	2,234978812	2,876306874
RTC145	3,108885923	1,844188427	2,394445957
RTC136	4,142886486	3,15525961	3,615505829

chr3	139240014	A	G	186,44	PASS	2	RTC113<br>RTC114	intronic	RBP1
chr3	139240018	C	T	31,77	PASS	1	RTC114	intronic	RBP1
chr12	6643927	T	G	37,77	PASS	1	RTC098_0	intronic	GAPDH
chr12	6643991	C	G	819,21	PASS	9	RTC095_0<br>RTC105_0<br>RTC107_0<br>RTC117_1<br>RTC127<br>RTC131_0<br>RTC139<br>RTC143_0<br>RTC148_0	exonic	GAPDH
chr12	6644256	T	A	150,36	PASS	5	RTC098_0<br>RTC105_0<br>RTC115<br>RTC117_1<br>RTC148_0	intronic	GAPDH
chr12	6644696	T	G	152,77	PASS	3	RTC098_0<br>RTC107_0<br>RTC117_1	intronic	GAPDH
chr12	6644722	A	G	158,1	PASS	3	RTC098_0<br>RTC107_0<br>RTC117_1	intronic	GAPDH
chr12	6644781	T	C	149,37	PASS	5	RTC095_0<br>RTC107_0<br>RTC117_1<br>RTC145_0<br>RTC148_0	intronic	GAPDH
chr12	6645565	T	C	77,65	PASS	4	RTC095_0<br>RTC098_0<br>RTC107_0<br>RTC117_1	intronic	GAPDH
chr12	6646299	C	T	90,27	PASS	2	RTC123_0<br>RTC148_0	exonic	GAPDH
chr12	6646320	T	C	1252,87	PASS	10	RTC095_0<br>RTC098_0<br>RTC105_0<br>RTC107_0<br>RTC113<br>RTC115<br>RTC117_1<br>RTC139<br>RTC145_0<br>RTC148_0	exonic	GAPDH
chr12	6647109	T	C	1236,77	PASS	10	RTC095_0<br>RTC098_0<br>RTC105_0<br>RTC107_0<br>RTC113<br>RTC115<br>RTC117_1<br>RTC139<br>RTC145_0<br>RTC148_0	exonic	GAPDH
chr12	6647453	G	T	126,77	PASS	8	RTC098_0<br>RTC105_0<br>RTC107_0<br>RTC113<br>RTC115<br>RTC117_1<br>RTC145_0<br>RTC148_0	exonic	GAPDH
