# Supplementary material for: Hematopoietic stem-cell senescence and myocardial repair - Coronary artery disease genotype/phenotype analysis of post-MI myocardial regeneration response induced by CABG/CD133+ bone marrow hematopoietic stem cell treatment in RCT PERFECT Phase 3
Source: eBioMedicine. 2020 Jul 4;57:102862. doi: 10.1016/j.ebiom.2020.102862 (PMC7339012; doi:10.1016/j.ebiom.2020.102862)
Supplement: Supplementary file 10 [file mmc10.docx]

**Supplementary Data SD2: Experimental SH2B3 ^-/-^ mouse myocardial infarction model**

**Experimental findings**

*Structural integrity and LV function of the infarcted myocardium is preserved*

First, we compared LV function of WT mice and LNK/SH2B3^-/-^ mice on day 28 post MI by echocardiography (Figure 1a). Fractional shortening (FS: WT, 17∙7±2∙6 vs. LNK/SH2B3^-/-^, 29∙2±5∙6 %, P<0∙01) (Figure 1b), a parameter of global left ventricular contractility and regional wall motion score (RWMS) were examined (RWMS: WT, 25∙4±3∙4 vs. LNK/SH2B3^-/-^, 19∙9±0∙9, P<0∙01) (Figure 1c). LV function was also examined using micromanometer-tipped catheter on day 28. +dP/dt, -dP/dt and end-diastolic pressure (EDP) were significantly better preserved in LNK/SH2B3^-/-^ mice (+dP/dt: WT, 5942∙1±823∙7 vs. LNK/SH2B3^-/-^, 8901∙6±1147∙9 mmHg/sec, P<0∙01; -dP/dt: WT, -4675∙9±615∙9 vs. LNK/SH2B3^-/-^, -6201∙4±875∙4 mmHg/sec, P<0∙01; EDP: WT, 8∙6±2∙1 vs. LNK/SH2B3^-/-^, 4∙4±1∙2 mmHg, P<0∙05) (Figure 1d, 1e and 1f). Histological analysis was performed on day 28 post MI (Figure 1g-j). The percentage of fibrosis area was less in LNK/SH2B3^-/-^ mice than WT mice (WT, 15∙2±4∙3 *vs*. LNK/SH2B3^-/-^, 8∙0±5∙0 %, P<0∙05) (Figure 1g and 1h). Capillary density in infarction border zone was significantly greater in LNK/SH2B3^-/-^ mice than WT mice (WT, 713±28 vs. LNK/SH2B3^-/-^, 937±157/mm^2^, P<0∙01) (Figure 1i and 1j). On the other hand, there was no significant difference in LV function and capillary density between WT mice and LNK/SH2B3^-/-^ mice without ischemic injury (Supplemental Figure 1S). These results suggest that gene deficiency of LNK/SH2B3 contributes to the preservation of LV function and structural integrity of infarcted myocardium post MI.

**
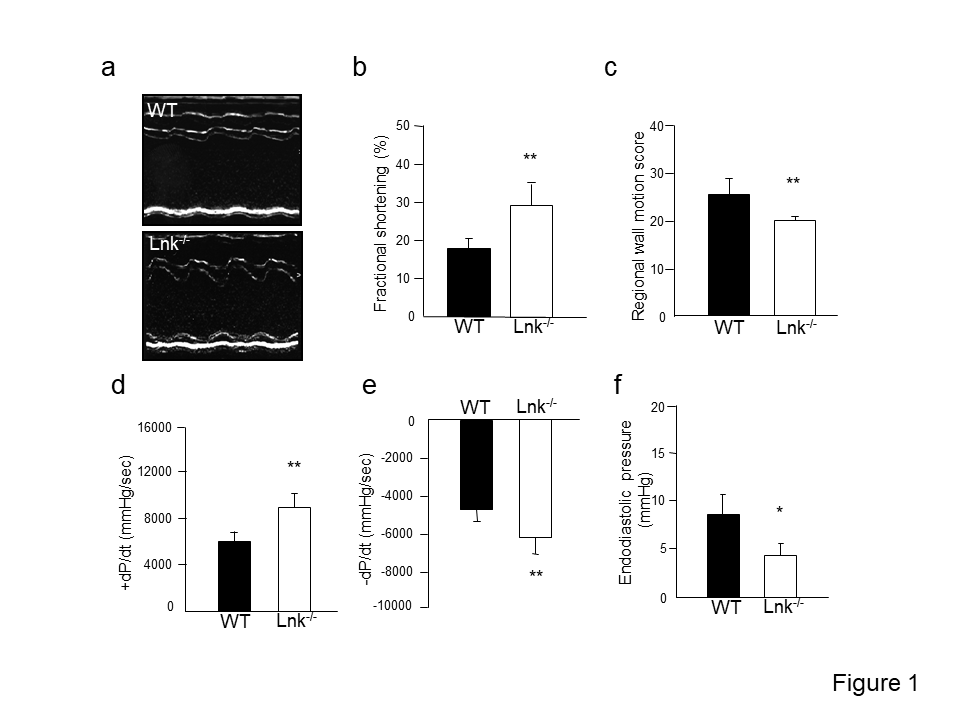
**


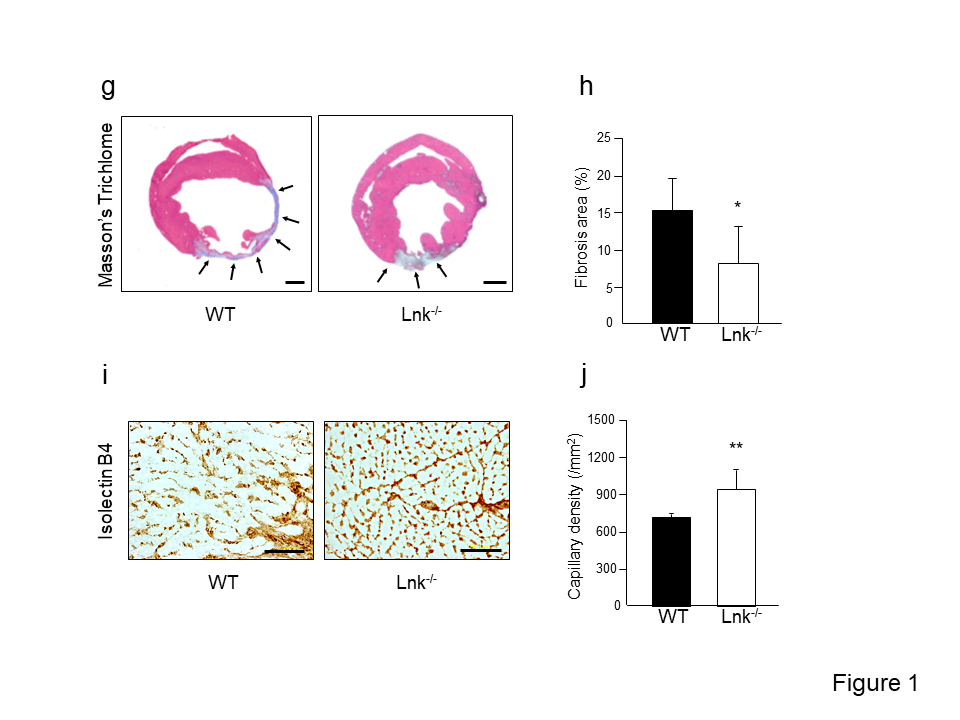


**Figure 1: Physiological and histological assessment for LV function in WT *vs*. SH2B3/LNK^-/-^ mice following MI.** a) Representative recordings of M-mode echocardiography in WT mice and SH2B3/LNK^-/-^ mice 28 days following MI. Fractional shortening (b) and regional wall motion score (c) were significantly greater in SH2B3/LNK^-/-^ mice than that in WT mice. Hemodynamic study using a micro-tip catheter in WT mice and SH2B3/LNK^-/-^ mice 28 days following MI. +dP/dt (d), -dP/dt (e) and EDP (f) were significantly preserved in SH2B3/LNK^-/-^ mice than those in WT mice. *, p<0∙05 and **, p<0∙01 *vs.* WT. (n=11) (+dP/dt: WT, 5.942∙1±823∙7 *vs*. SH2B3/LNK^-/-^, 8∙901∙6±1∙147∙9 mmHg/sec, p<0∙01; -dP/dt: WT, -4∙675∙9±615∙9 *vs*. SH2B3/LNK^-/-^, -6∙201∙4±875∙4 mmHg/sec, p<0∙01; EDP: WT, 8∙6±2∙1 *vs*. SH2B3/LNK^-/-^, 4∙4±1∙2 mmHg, p<0∙05) (Figure 4d, 4e and 4f). (Figure 4g and 4h). (g) Representative Masson’s trichrome stained heart sections in WT mice and SH2B3/LNK^-/-^ mice 28 days following MI. Arrows: fibrosis area (blue). Scale bar = 1 mm. (h) Percent of fibrosis area in entire LV area on cross-sections. Histological analysis was performed on day 28 post MI (Figure 4g-j). The percentage of fibrosis area was less in SH2B3/LNK^-/-^ mice than WT mice (WT, 15∙2±4∙3 *vs*. SH2B3/LNK^-/-^, 8∙0±5∙0 %, p<0∙05). Fibrosis area was significantly reduced in SH2B3/LNK^-/-^ mice compared with WT mice. * p<0∙05 *vs*. WT. (WT: n=6 and SH2B3/LNK^-/-^: n=10) (i) Representative images of immunostaining for isolectin B4 (brown) in WT and SH2B3/LNK^-/-^ mice 28 days following MI. Scale bar = 100 µm. (j) Capillary density in ischemic border zone in infarcted myocardium of WT mice and SH2B3/LNK^-/-^ mice. **, p<0∙01 *vs*. WT. (WT: n=5 and SH2B3/LNK^-/-^: n=9) Capillary density in infarction border zone was significantly greater in SH2B3/LNK^-/-^ mice than WT mice (WT, 713±28 *vs*. SH2B3/LNK^-/-^, 937±157/mm^2^, p<0∙01) (i and j). On the other hand, there was no significant difference in LV function and capillary density between WT mice and SH2B3/LNK^-/-^ mice without ischemic injury (Supplemental Figure S3). Graphs depict mean ± SEM. Statistical significance was determined using Mann-Whitney comparison test.


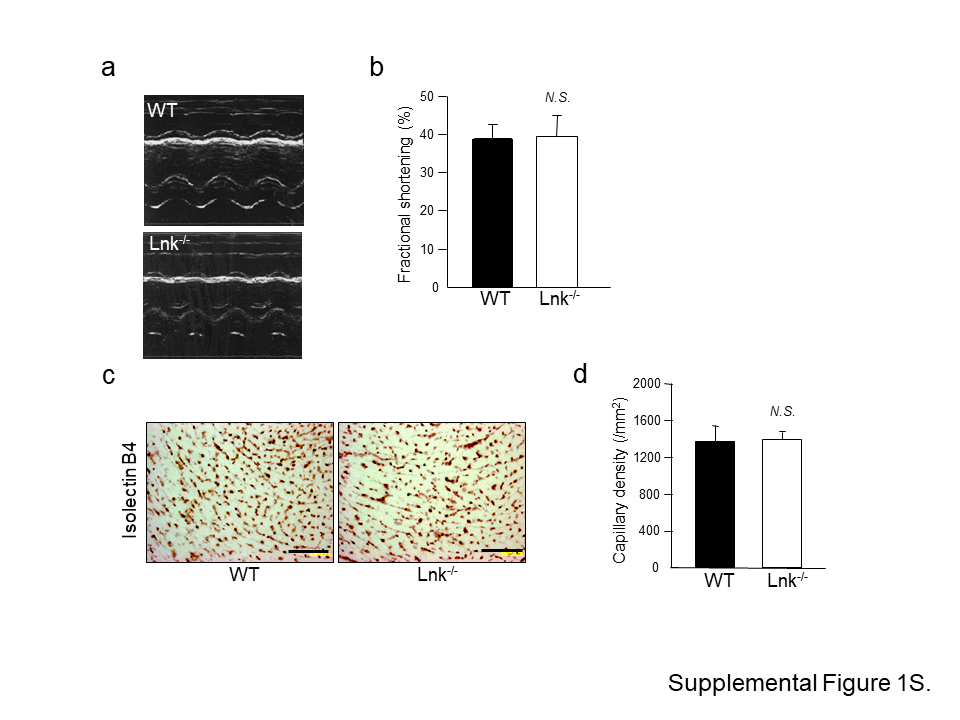


**Supplementary Figure 1S. Physiological and histological assessment for LV function in normal WT versus SH2B3/LNK^-/-^ mice.** a) Representative recordings of M-mode echocardiography in WT mice and SH2B3/LNK^-/-^ mice pre MI. Fractional shortening (b) was not significantly different between WT mice and SH2B3/LNK^-/-^ mice. (WT: n=6 and SH2B3/LNK^-/-^: n=8) (c) Representative images of immunostaining for isolectin B4 (brown) in WT and SH2B3/LNK^-/-^ mice pre MI. Scale bar =100 µm. (d) Capillary density in normal myocardium was not significantly different between WT mice and SH2B3/LNK^-/-^ mice (WT: n=4 and SH2B3/LNK^-/-^: n=3). Graphs depict mean ± SEM. Statistical significance was determined using Mann-Whitney comparison test. (*n.s*; statistical not significant)

*Echocardiography and hemodynamic measurements.* Transthoracic echocardiography (SONOS 5500, Philips Medical Systems) was performed to evaluate LV function immediately before and 28 days after MI as described previously. Under general anesthesia with ketamine and xylazine (60 mg/kg and 10 mg/kg, respectively, intraperitoneal injection), LVEDD, LVESD and FS were measured at the midpapillary muscle level. RWMS was evaluated per published criteria. Immediately after the final echocardiography on day 28, the mice underwent cardiac catheterization for more invasive and precise assessment of global LV function as described previously. A 2.0 Fr micromanometer-tipped conductance catheter (SPR 838, Millar Instruments Inc, Tx) was inserted via a right carotid artery into LV cavity. LV pressure and its derivative (dP/dt) were continuously monitored using a multi-channel recording system (Pressure-Volume Conductance System ARIA and Pressure-Volume Analysis Using P-V Analysis Software [Millar Instruments Inc] and Power Lab^®^ DAQ System [AD Instrument, Australia]). HR, EF, +dP/dt and –dP/dt were continuously recorded for 20 minutes. All data were acquired under stable hemodynamic conditions. End systolic pressure volume relation analysis (ESPVR) and preload-recruitable stroke work (PRSW) analysis are not included.

*Increased number of KSL population in BM and enhanced mobilization of BM-derived EPCs into circulation post MI*

To elucidate the effect of LNK/SH2B3-deficiency on EPC kinetics in the setting of MI, we examined the frequency of KSL population, which is known as EPC-enriched fraction in BMMNCs. Flow cytometric analysis was performed pre and on day1, 3, 7, 14 and 28 post MI (Figure 2a and 2b). It is previously reported that KSL fraction is increased in LNK/SH2B3^-/-^ mice compared to WT mice even in pre-infarction. Furthermore, KSL population was significantly increased in LNK/SH2B3^-/-^ mice compared to WT mice, as well as on day 1, 3, 7, 14 and 28 (day 7: WT, 12∙9 ± 3∙7 vs. LNK/SH2B3^-/-^, 22∙9±3∙1 %, P<0∙01; day 14: WT, 10∙1±1∙0 *vs*. LNK/SH2B3^-/-^, 22∙9±4∙8 %, P<0∙01) (Figure 2b). To examine the effect of LNK/SH2B3-deficiency on EPC mobilization into circulation post MI, we performed FACS analysis for Sca-1^+^/ lineage^-^ cells, an EPC-enriched fraction in PB. The number of the cells pre-infarction was similar in LNK/SH2B3^-/-^ mice and WT mice, whereas on days 3 and 14 post infarction, it was significantly greater in LNK/SH2B3^-/-^ mice than WT mice (day 3: WT, 25∙3±3∙0 *vs*. LNK/SH2B3^-/-^, 37∙0±9∙4×10^4^ cells/mL, P<0∙05; Day 14: WT, 23∙5±3∙7 vs. LNK/SH2B3^-/-^, 56∙0±12∙4×10^4^ cells/mL, P<0∙001) (Figure 2c).

Next, we further examined whether the increased number of EPC-enriched cell fraction in BM and PB in LNK/SH2B3^-/-^ mice is due to cell proliferation by FACS analysis for BrdU incorporation into the cells (Figure 2d). The percentage of BrdU positive KSL cells in BM was significantly high in LNK/SH2B3^-/-^ mice compared to WT mice pre and day 7 post MI (Pre: WT, 2∙6±0∙4 vs. LNK/SH2B3^-/-^, 6∙0±1∙1 %, P<0∙01; day 7: WT, 5∙9±0∙5 vs. LNK/SH2B3^-/-^, 8∙6±1∙0 %, P<0∙05) (Figure 2e). These findings suggest that LNK/SH2B3 deficiency contributes to an increase in the EPC-enriched fraction with high proliferation activity in BM and enhanced mobilization of BM derived EPCs into circulation following MI.

**
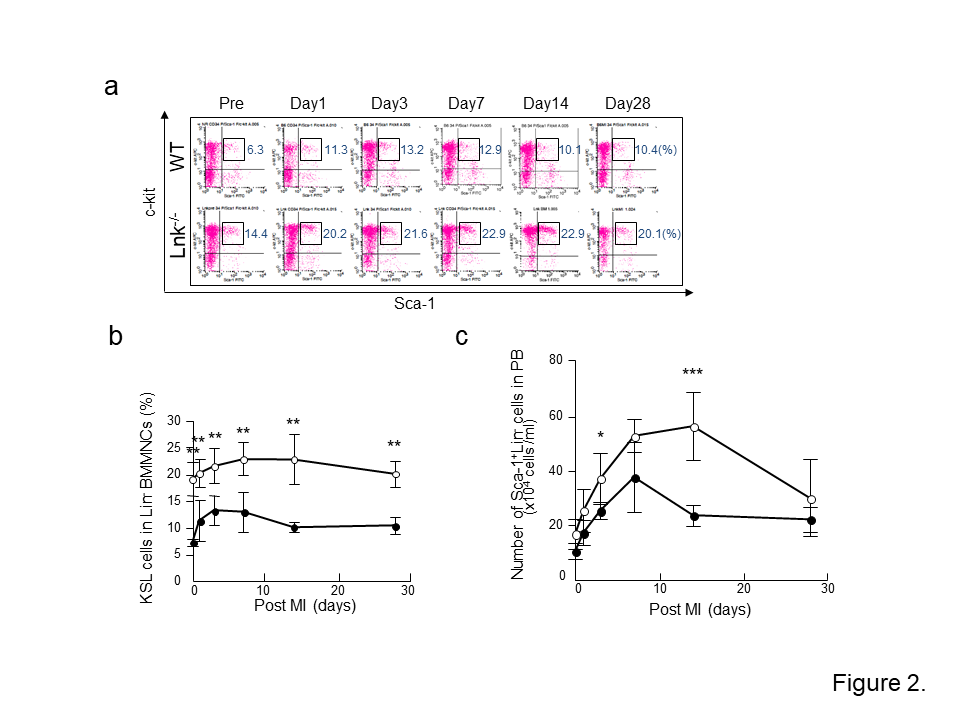
**

**
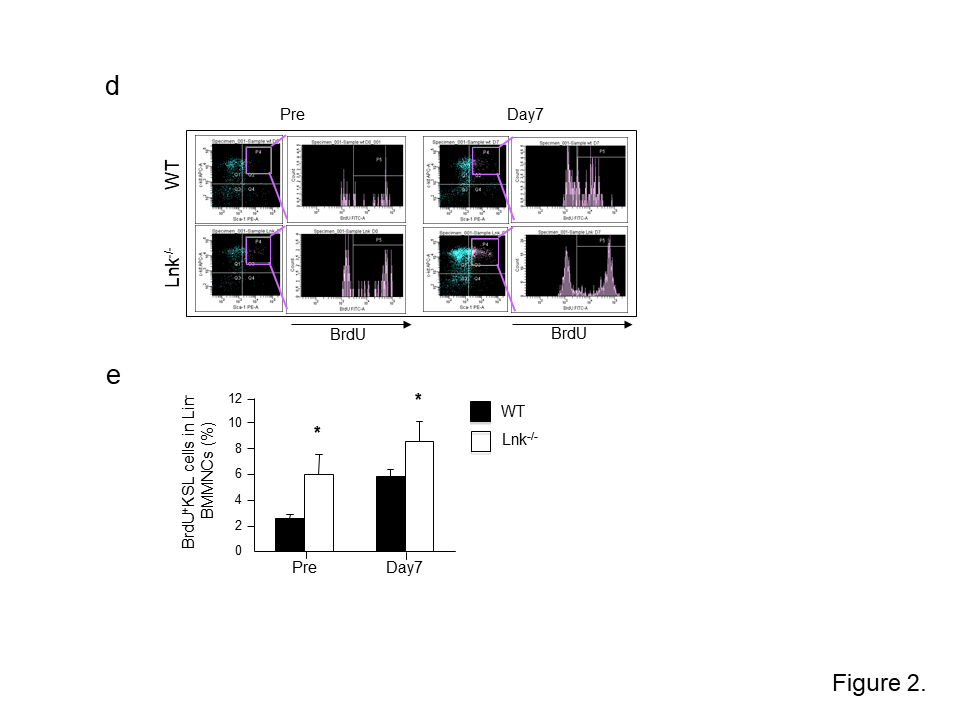
**

**Figure 2: Kinetics of KSL cells in BM and SL cells in PB in WT *vs*. SH2B3/LNK^-/-^ mice following MI. SH2B3/LNK -/- leads to increased EPC in bone marrow and circulation post MI**

a) Representative examples of serial FACS analysis of c-KIT^+^/Sca-1^+^ cells in Lin^-^ BMMNCs (KSL cells) of WT mice and SH2B3/LNK^-/-^ mice before (Pre) and one day, 3, 7, 14 and 28 days following MI. (b) Percent of KSL cells in Lin^-^ BMMNCs before and after MI significantly increased in SH2B3/LNK^-/-^ mice (open circles) compared with WT mice (closed circles). **, p<0∙01 *vs*. WT (n=3-4). (c) Number of Sca-1^+^/Lin^-^ (SL) cells in PB in SH2B3/LNK^-/-^ mice (open circles) and WT mice (closed circles) before (Pre) and one day, 3, 7, 14 and 28 days after MI. *, p<0∙05 and ***, p<0∙01 *vs*. WT (n=3-4). (d) Representative recordings of FACS analyses for BrdU^+^ KSL cells in BMMNCs before (Pre) and 7 days after MI. Left panel, KSL population and right panel, histogram of BrdU positivity in KSL population. (e) Percent of BrdU^+^ KSL cells in Lin^-^ BMMNCs before (Pre) and 7 days after MI significantly increased SH2B3/LNK^-/-^ mice compared with WT mice. *, p<0∙05 *vs*. WT (n=2). Graphs depict mean ± SEM. Statistical significance was determined using two-way ANOVA followed by Tukey’s multiple comparisons test.

*Experimental analysis in BM-KSL cells*

Since it has been shown that BM-derived cells recruited to sites of ischemia promoting angiogenesis via the paracrine effect, we compared gene expressions in BM KSL cells in WT mice and those in LNK/SH2B3^-/-^ mice by quantitative real-time RT-PCR analysis to examine the effect of LNK/SH2B3 deficiency on angiogenic factor and chemokine production in BM-derived cells. The mRNA expression of angiogenic factors (VEGF-B, FGF-4, HGF and Ang-1), survival factor (IGF-1), and stem/progenitor chemokine (IGF-2 and SDF-1) was significantly up-regulated in LNK/SH2B3^-/-^ mice than WT mice (VEGF-B: WT, 224∙6±72∙6 vs. LNK/SH2B3^-/-^, 515∙8±109∙3, P<0∙05; FGF-4: WT, 231∙1±11∙2 vs. LNK/SH2B3^-/-^, 408∙9±22∙1, P<0∙05; HGF: WT, 71∙5±8∙2 vs. LNK/SH2B3^-/-^, 131∙9±21∙1, P<0∙05; Ang-1: WT, 1210∙9±98∙5 vs. LNK/SH2B3^-/-^, 4216∙6±800∙5, P<0∙05; IGF-1: WT, 208∙7±4∙4 vs. LNK/SH2B3^-/-^, 349∙5±34∙8, P<0∙05; IGF-2: WT, 183∙7±23∙5 vs. LNK/SH2B3^-/-^, 328∙4±6∙1, P<0∙05; SDF-1: WT, 193∙4±7∙1 vs. LNK/SH2B3^-/-^, 383∙8±194∙4, P<0∙05) (Figure 3), while there was no significant difference of other angiogenesis-related gene expression between LNK/SH2B3^-/-^ mice and WT mice (Supplemental Figure 2S). These data indicate that LNK/SH2B3 deficiency enhances the recruitment of BM-derived EPCs to infarcted myocardium up-regulating the expression of favorable factors and these effects may act synergistically on angiogenesis in ischemic myocardium.


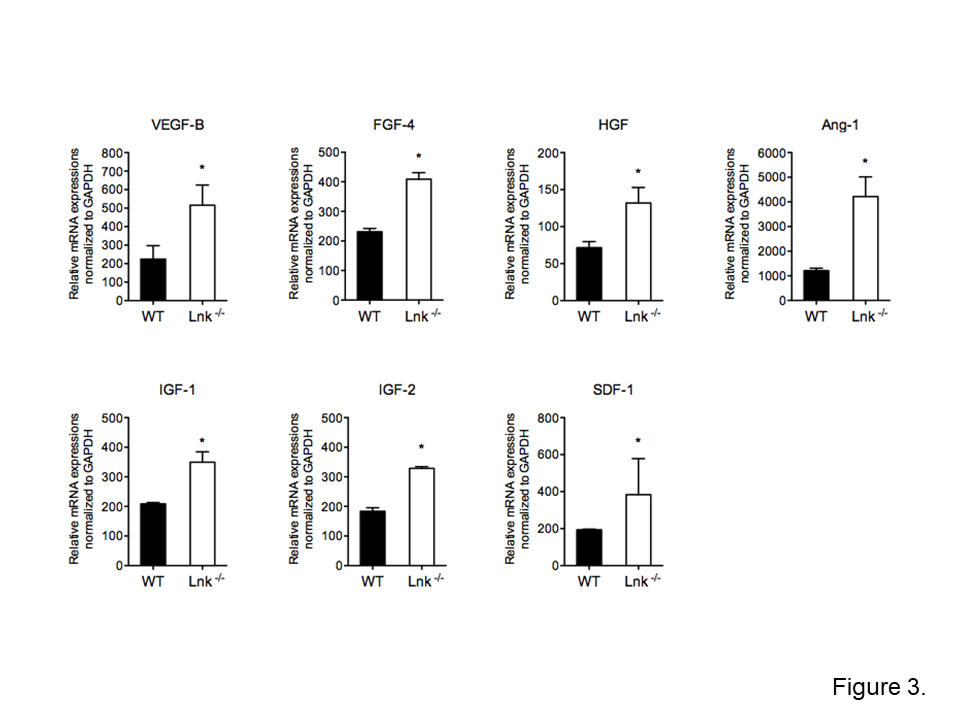


**Figure 3: Growth Factor and Chemokine mRNA expressions in WT BM-KSL cells *vs*. SH2B3/LNK^-/-^ BM-KSL cells.**

KSL cells were sorted from freshly isolated BMMNCs by FACS, and were analyzed the expressions of VEGF-B, FGF-4, HGF, Ang-1, IGF-1, IGF-2 and SDF-1 by quantitative real-time RT-PCR. Each relative mRNA expression was normalized to GAPDH and compared between WT BM-KSL cells (solid bar) and SH2B3/LNK^-/-^ BM-KSL cells (open bar). Graphs depict mean ± SEM. *, p<0∙05 as determined by Mann-Whitney comparison tests (n=3).


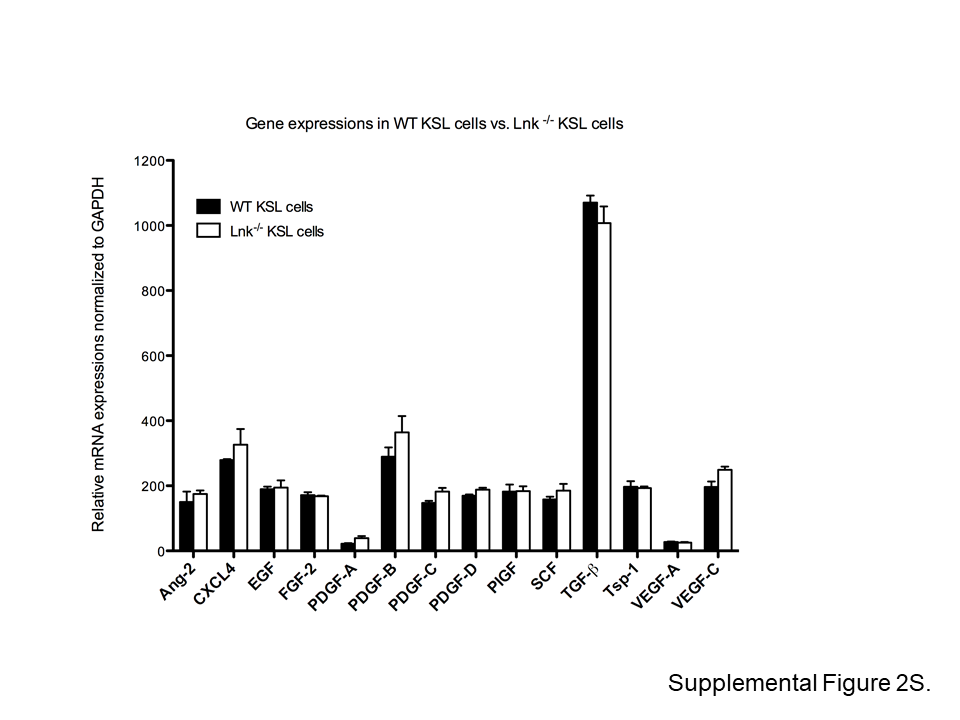


**Supplementary Figure 2S. Effect of SH2B3/LNK gene deficiency on growth factor and chemokine expressions in BM KSL cells.** KSL cells were sorted from freshly isolated BMMNCs by FACS, and were analyzed the expressions of Ang-2, CXCL4, EGF, FGF-2, PDGF-A, PDGF-B, PDGF-C, PDGF-D, PlGF, SCF, TGF-β, Tsp-1, VEGF-A, and VEGF-C by quantitative real-time RT-PCR. Each relative mRNA expression was normalized to GAPDH and compared between WT BM-KSL cells (solid bar) and SH2B3/LNK^-/-^ BM-KSL cells (open bar). Expression of these factors was not significantly different between WT mice and SH2B3/LNK^-/-^ mice (n=3). Graphs depict mean ± SEM. Statistical significance was determined using two-way ANOVA followed by Bonferroni’s or Sidak’s multiple comparisons test.

| Gene Name | GenBank Accession # | Primer Sequence |
| --- | --- | --- |
| Ang-1 | NM_009640.3 | F: ATC TTG ATA ACC GCA GCC AC |
|  |  | R: TGT CGG CAC ATA CCT CTT GT |
| Ang-2 | NM_007449.2 | F: CAA CAT CAA GGC CAT CTG TG |
|  |  | R: GGG GAG ACC TTC CTT TGT GT |
| EGF | NM_010113.3 | F: ACG GTT TGC CTC TTT TCC TT |
|  |  | R: AGT GTT CCA AGC GTT CCT GA |
| CXCL4 | NM_019932.4 | F: GCT TCT GGG CCT GTT GTT T |
|  |  | R: CCC AGA GGA GAT GGT CTT CA |
| CXCL12 (SDF-1) | NM_021704.3 | F: GCC ACG GTG TAT TTT TCC AC |
|  |  | R: CCC ACA TAC GGT AGG AGG TTT |
| FGF-2 | NM_008006.2 | F: AAG CGG CTC TAC TGC AAG AA |
|  |  | R: GGC ACA CAC TCC CTT GAT AGA |
| FGF-4 | NM_010202.5 | F: CTC CAA GAG TGT TGG GGA GA |
|  |  | R: CAC TAG GAA ATG GGG CAA GA |
| HGF | NM_010427.4 | F: TCC CTG AAA AGA CCA CTT GC |
|  |  | R: AAG TCA CCT TGC CTT GAT GG |
| IGF-1 | NM_010512.4 | F: GTG GCT GAC CCC TCT CAC TA |
|  |  | R: GAG CCT CCT GCC AAG TGT T |
| IGF-2 | NM_010514.3 | F: CCC TCA GCA AGT GCC TAA AG |
|  |  | R: TGG ATA CCA GGC CAA TTC AT |
| LNK/SH2B3 | NM_008507.3 | F: GGC ACC AGG TTC CTC CAA CA |
|  |  | R: CCT CTC TGC ACA GCT GTG AGA |
| PDGF-A | NM_008808.3 | F: GAG ATA CCC CGG GAG TTG AT |
|  |  | R: CCA TGG GCT CTC AGA CTT GT |
| PDGF-B | NM_011057.3 | F: GAG ACC TCC AAG GGA GGA AC |
|  |  | R: CTC TCC CCC ACT TCC AAT CT |
| PDGF-C | NM_019971.2 | F: GTC GCA GGG ACA GTT TGA TAA |
|  |  | R: TTT CCC ATA GAA AGG GGT AGG T |
| PDGF-D | NM_027924.2 | F: AAC TGT GGT TGC GGA ACT GT |
|  |  | R: TTG CCC CTT CTC TTG AAA TG |
| PlGF | NM_008827.2 | F: TGG AGA TGG GAC AGG ACA TT |
|  |  | R: CAC TCT GCC TGT GTT CCA GA |
| SCF | NM_013598.2 | F: TGT GGG CTT AGG AGT GAT CC |
|  |  | R: TTG GAG ATG GCA GTT GTG C |
| TGF-β | NM_011577.1 | F: GTC CTT GCC CTC TAC AAC CA |
|  |  | R: GTT GGA CAA CTG CTC CAC CT |
| TSP-1 | NM_011580.3 | F: GGC TTC CCC TTT GTT TTC TC |
|  |  | R: CTC CCT GGA AAT AGG CAC AA |
| VEGF-A | NM_001025250.3 | F: AGC ACA GCA GAT GTG AAT GC |
|  |  | R: AAT GCT TTC TCC GCT CTG AA |
| VEGF-B | NM_011697.3 | F: CAA GTC CGA ATG CAG ATC CT |
|  |  | R: TGT CTG GCT TCA CAG CAC TC |
| VEGF-C | NM_009506.2 | F: CCT GAA TCC TGG GAA ATG TG |
|  |  | R: TCC TGG ATC ACA ATG CTT CA |
| α-actin | NM_007393.3 | F: GCC TTC CTT CTT GGG TAT G |
|  |  | R: TTT CTG CGC AAG TTA GGT TT |

**Supplement Table S1: List of primer sequences used for mouse analysis.**

*LNK/SH2B3 deficiency enhances BM-derived EPC recruitment to ischemic myocardium*

Next, to examine the effect of LNK/SH2B3 gene deletion on BM-derived EPCs, MI was induced in LNK/SH2B3^-/-^ mice and WT mice, in which BM c-KIT^+^/Lin^-^/GFP^+^ cells were transplanted from LNK/SH2B3^-/-^/GFP (GFP LNK/SH2B3^-/-^ BMT LNK/SH2B3^-/-^) and LNK/SH2B3^+/+^/GFP mice (GFP BMT WT) following lethal irradiation, respectively. Double immune- and chemical-staining for GFP and isolectin B4 at day 7 post MI (Figure 4a) revealed that recruitment of BM-derived EPCs to infarcted myocardium was significantly increased in LNK/SH2B3^-/-^ mice than WT mice (WT, 87∙1±74∙9 vs. LNK/SH2B3^-/-^, 647∙1±174∙7/mm^2^, P<0∙001) (Figure 4b). Moreover, incorporation of BM-derived EPCs into capillary vessels was significantly increased in LNK/SH2B3^-/-^ EPCs than WT EPCs (WT, 13∙8±9∙6 vs. LNK/SH2B3^-/-^, 92∙0±27∙0/mm^2^, P<0∙001) (Figure 4c) even at day 28 post MI. These results indicate that LNK/SH2B3 deficiency in BM enhances not only EPC proliferation and mobilization into circulation, but also EPC recruitment to infarcted myocardium and incorporation into capillaries.

**
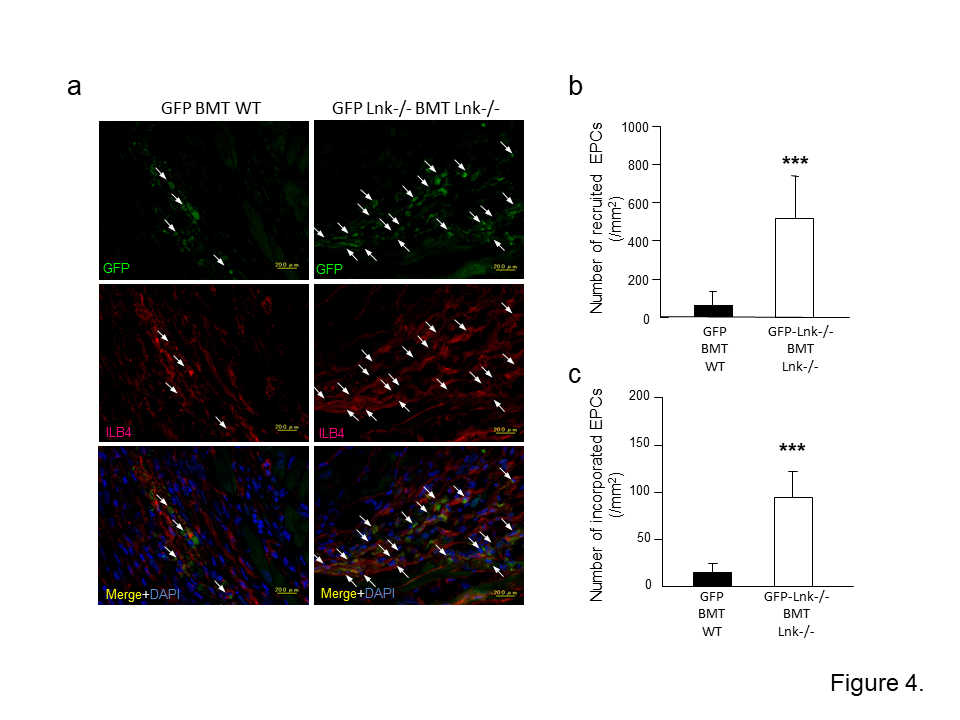
Figure 4: Effect of SH2B3/LNK gene deficiency on recruitment of BM-derived progenitors to ischemic myocardium.**

a) Representative double fluorescent immunostaining for GFP (green) and isolectin B4 (red) in heart sections in WT mice transplanted with GFP^+^ BM and in SH2B3/LNK^-/-^ mice transplanted with GFP^+^- SH2B3/LNK^-/-^ BM 7 days following MI. Arrows, recruited BM-derived cells to ischemic myocardium (GFP^+^/isolectin B4^+^ cells). Scale bar =20 µm. Number of recruited BM-derived cells in ischemic myocardium 7 days following MI (b) and incorporated BM-derived cells into vasculature in ischemic myocardium 28 days following MI (c) were counted and averaged. In the graph, **, p<0∙01 and ***, p<0∙001 as determined by Mann-Whitney comparison test *vs*. WT mice transplanted with GFP BM. (n=3).

*Cardiac stem/progenitor cells increase in LNK/SH2B3^-/-^ mice*

Recently, c-KIT^+^ cardiac stem/progenitor cells (CS/PCs) were identified in adult mouse heart. Isolated and expanded CSCs derived from adult rat hearts and human hearts support myocardial repair after MI. Together with the fact that LNK/SH2B3 was a negative regulator of self-renewality of HSC/KSL population in BMMNCs and increased in LNK/SH2B3^-/-^ mice, we hypothesized that c-KIT^+^ CS/PCs were augmented in the heart of LNK/SH2B3^-/-^ mice. First, we analyzed LNK/SH2B3 gene expression in heart tissue of WT mice and LNK/SH2B3^-/-^ mice pre and post MI. LNK/SH2B3 gene was expressed in whole heart tissue in WT mice but not in LNK/SH2B3^-/-^ mice (Supplemental Figure S3a). Moreover, in WT mice, LNK/SH2B3 gene expression was significantly higher in sorted cardiac c-KIT^+^ cells than c-KIT^-^ cells (Supplemental Figure 3Sb). Next, CS/PC kinetics in intact heart was examined. Frequency of c-KIT^+^ cells in whole heart analyzed by FACS was greater in LNK/SH2B3^-/-^ mice than WT mice (WT, 3∙2±0∙7 vs. LNK/SH2B3^-/-^, 5∙2±0∙6 %, P<0∙05) (Figure 5a and 5b). We also performed immunofluorescent staining to detect c-KIT^+^/ GATA4^-^ cells as CSCs and c-KIT^+^/ GATA4^+^ cells as CPCs. Morphological quantification revealed significant increase of CSCs and CPCs in LNK/SH2B3^-/-^ mice than WT mice in intact myocardium (CSCs: WT, 0∙8±0∙5 vs. LNK/SH2B3^-/-^, 1∙6±0∙3, P<0∙05; CPCs: WT, 0∙5±0∙2 vs. LNK/SH2B3^-/-^, 1∙3±0∙6 cells/mm^2^, P<0∙01) (Figure 5d). CSCs and CPCs were identified as well in the infarcted myocardium at day 7 post MI (Figure 5c). Number of CSCs and CPCs were also significantly greater in LNK/SH2B3^-/-^ mice (CSCs: WT, 1∙4±1∙1 vs. LNK/SH2B3^-/-^, 4∙2±1∙7, P<0∙01; CPCs: WT, 0∙9±0∙4 vs. LNK/SH2B3^-/-^, 1∙7±0∙4 cells/mm^2^, P<0∙05) (Figure 5d). These data show that gene deficiency of LNK/SH2B3 may increase CSCs and CPCs in infarcted myocardium as well as those in intact myocardium.

**
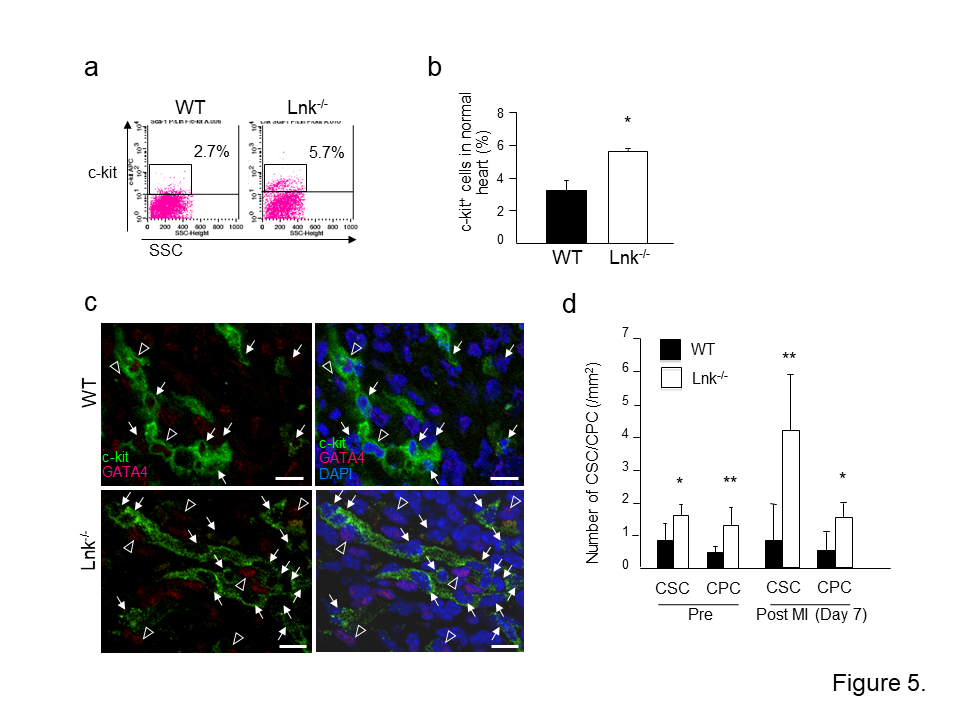
**

**Figure 5: Assessment for proliferation activity in CSCs/CPCs and cardiomyocytes in ischemic myocardium.**

(a) Representative double fluorescent immunostaining for BrdU (red) and c-KIT (green) in heart sections in WT mice and in SH2B3/LNK^-/-^ mice 7 days following MI. Arrows, proliferating CPCs (BrdU^+^/c-KIT^+^ cells). Scale bar = 10µm. (b) Number of BrdU^+^/c-KIT^+^ cells in ischemic myocardium 7 days following MI were counted and averaged. *, p<0,05 *vs.* WT mice (WT: n=4 and SH2B3/LNK^-/-^: n=3). (c) Representative double fluorescent immunostaining for BrdU (red) and cardiac troponin I (cTnI, green) in heart sections in WT mice and in SH2B3/LNK^-/-^ mice 7 days following MI. Arrows, proliferating cardiomyocytes (BrdU^+^/cTnI^+^ cells). Scale bar =100 µm. (d) Number of BrdU^+^/cTnI^+^ cells in ischemic myocardium 7 days following MI. *, p<0∙05 *vs.* WT mice (WT n=4, SH2B3/LNK^-/-^ n=3). Graphs depict mean ± SEM. Statistical significance was determined using 1-way ANOVA followed by Bonferroni’s multiple comparisons test.


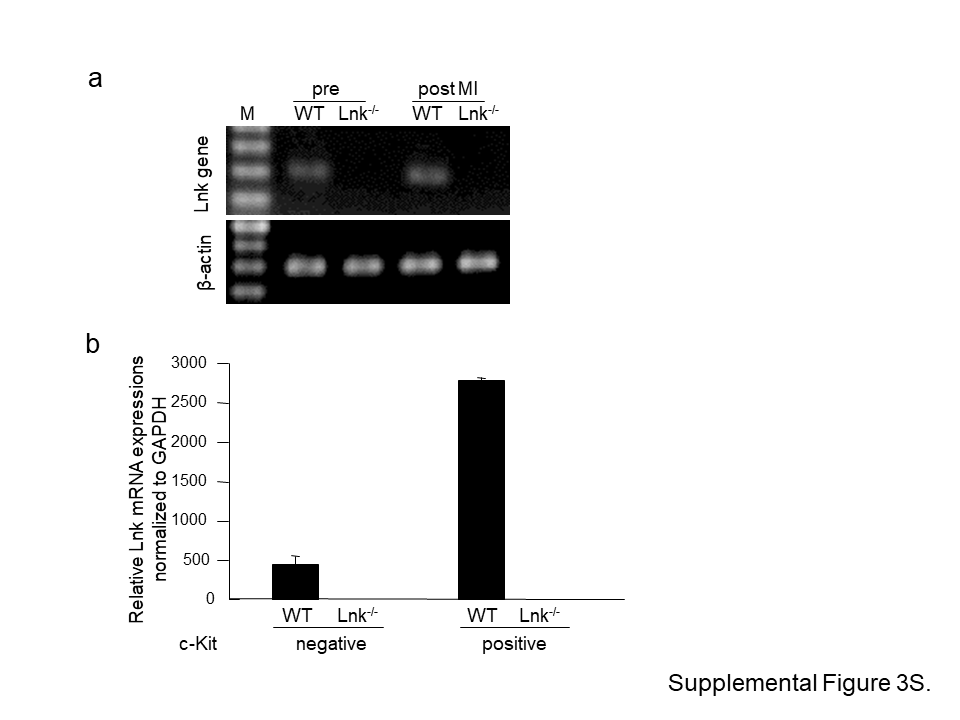


**Supplementary Figure 3S. Detection of SH2B3/LNK gene expression in myocardium.** a) The tissue samples of intact heart (pre) and post MI heart (day 7 after MI) were harvested from WT mice and SH2B3/LNK^-/-^ mice, and SH2B3/LNK and beta-actin mRNA expressions were examined by RT-PCR analysis. M, PCR loading marker. (b) SH2B3/LNK mRNA expression was examined in c-KIT^+^ cells (CS/PCs) and c-KIT ^–^ cells sorted by FACS from MNCs of intact heart sample in WT mice and SH2B3/LNK^-/-^ mice by quantitative real-time RT-PCR analysis. Relative mRNA expressions of SH2B3/LNK in c-KIT^+^ cells and c-KIT ^-^ cells were normalized to GAPDH and compared between WT mice and SH2B3/LNK^-/-^ mice. Graphs depict mean ± SEM.

*Proliferation activity in CSCs/CPCs and cardiomyocytes in ischemic myocardium is upregulated in LNK/SH2B3^-/-^ mice*

To investigate whether increased CSCs/CPCs in LNK/SH2B3^-/-^ mice is due to CSC/CPC proliferation or cardiomyogenesis post MI, we evaluated BrdU incorporation in c-KIT^+^ CSCs/CPCs or cardiomyocytes in ischemic myocardium post MI. (Figure 6) The number of BrdU^+^/c-KIT^+^ cells and BrdU^+^/cardiac troponin I (cTnI)^+^ cells, defined as proliferating CSCs/CPCs (Figure 6a) and cardiomyocytes, (Figure 6c) respectively, significantly increased in LNK/SH2B3^-/-^ mice. (BrdU^+^/c-KIT^+^: WT, 44∙1±17∙3 vs. LNK/SH2B3^-/-^, 88∙8±12∙0, P<0∙05) (Figure 6b) (BrdU^+^/cTnI^+^: WT, 6∙3±3∙9 vs. LNK/SH2B3^-/-^, 19∙5±8∙8 cells/mm^2^, P<0∙01) (Figure 6d).


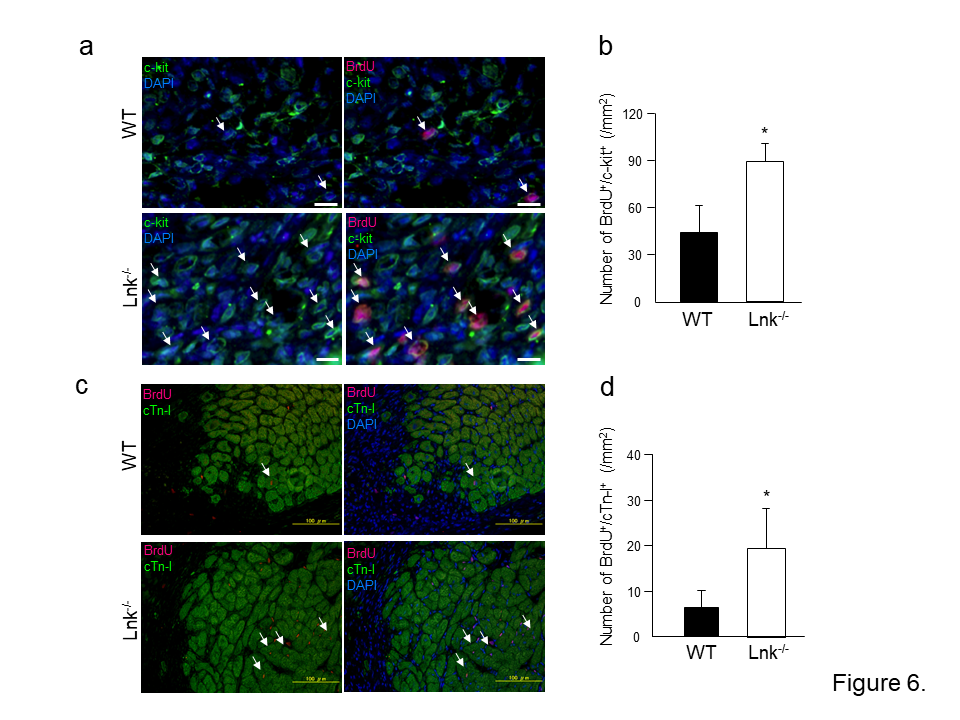


**Figure 6. Assessment for proliferation activity in CSCs/CPCs and cardiomyocytes in ischemic myocardium**

(a) Representative double fluorescent immunostaining for BrdU (red) and c-KIT (green) in heart sections in WT mice and in LNK/SH2B3^-/-^ mice 7 days following MI. Arrows, proliferating CSCs/CPCs (BrdU^+^/c-KIT^+^ cells). Scale bar = 10µm. (b) Number of BrdU^+^/c-KIT^+^ cells in ischemic myocardium 7 days following MI were counted and averaged. *, P<0∙05 versus WT mice (WT: n=4 and LNK/SH2B3^-/-^: n=3). (c) Representative double fluorescent immunostaining for BrdU (red) and cardiac troponin I (cTnI, green) in heart sections in WT mice and in LNK/SH2B3^-/-^ mice 7 days following MI. Arrows, proliferating cardiomyocytes (BrdU^+^/cTnI^+^ cells). Scale bar =100 µm. (d) Number of BrdU^+^/cTnI^+^ cells in ischemic myocardium 7 days following MI. *, P<0∙05 versus WT mice. (WT n=4, LNK/SH2B3^-/-^ n=3). Graphs depict mean ± SEM. Statistical significance was determined using either Mann-Whitney or one-way ANNOVA comparison tests.

To detect the recruitment of GFP^+^ BM-derived EPCs into infarcted myocardium, Alexa Fluor 488-conjugated rabbit anti-GFP (A-21311; in 1:200 dilution, Invitrogen) and Alexa Fluor 594-conjugated isolectin B4 (I21413; in 1:400 dilution; Invitrogen) were used to enhance the green fluorescent signal and visualize, respectively. To detect BrdU incorporating cells in CS/PCs and cardiomyocytes, sections were treated with antigen retrieval technique and incubated with mouse IgG blocking reagent (MKB-2213-1; Vector Laboratories) and immunestained with rabbit anti-c-KIT (A4502; in 1:100 dilution; Dako, Carpinteria, CA), mouse anti-cardiac troponin I (MAB3152; clone 8E10ab; Millipore), and mouse anti-BrdU (555627; BD Pharmingen). The secondary antibodies were used as follows: Alexa Fluor 488-conjugated goat anti-rat IgG (A-11006), Alexa Fluor 594-conjugated goat anti-rabbit IgG(A-11037), Alexa Fluor 594- conjugated goat anti-mouse IgG_1_ (A-21125), Alexa Fluor 488-conjugated goat anti-rabbit IgG (A-11008) and Alexa Fluor 488- conjugated goat anti-mouse IgG_2b_ (A-21141) (all from Invitrogen). The sections were counterstained with DAPI (D9542; Sigma). Images were captured with BX51 (Olympus, Tokyo, Japan) and FLUOVIEW FV1000 (Olympus). Number of immunostained cells was morphometrically quantified in 5 randomly selected fields in peri-infarcted area to fibrosis area in tissue sections.
