# Supplementary material for: Hematopoietic stem-cell senescence and myocardial repair - Coronary artery disease genotype/phenotype analysis of post-MI myocardial regeneration response induced by CABG/CD133+ bone marrow hematopoietic stem cell treatment in RCT PERFECT Phase 3
Source: eBioMedicine. 2020 Jul 4;57:102862. doi: 10.1016/j.ebiom.2020.102862 (PMC7339012; doi:10.1016/j.ebiom.2020.102862)
Supplement: Supplementary file 11 [file mmc11.pdf]

Intramyocardial transPlantation of bonE maRrow stem cells For improvEment of post-infarct myoCardial regeneraTion in addition to CABG surgery:

a controlled, prospective, randomized, double blinded multicenter trial

PERFECT

**Sponsor:**	Miltenyi Biotec GmbH, Bergisch Gladbach<br>Friedrich-Ebert-Straße 68<br>51429 Bergisch Gladbach<br>Germany<br>Phone: +49 (2204) – 8306-0<br>Fax: +49 (2204) – 8306-6699
**National Coordinating Investigator and Leiter der Klinischen Prüfung (LKP)** according to German Medical Products Act (“Arzneimittelgesetz”):	Prof. Dr. med. Gustav Steinhoff<br>UNIVERSITÄT ROSTOCK<br>Direktor Klinik und Poliklinik für Herzchirurgie, Schillingallee 35<br>D-18057 Rostock<br>Phone: +49 (381) 494-6100<br>E-mail: gustav.steinhoff@med.uni-rostock.de
**Trial Sites:**	7 sites in Germany
**Ethic Committee:**	Ethikkommission der Ärztekammer Mecklenburg-Vorpommern bei der Universität Rostock<br>St.-Georg-Str. 108, D-18055 Rostock
**EudraCT No.:**	2006-006404-11
**Sponsor Study No.:**	PERFECT 001 (M-2006-144)
**Study Drug Name:**	$CD133^+$ autologous bone marrow stem cells
**Development Phase:**	III
**Date of Protocol:**	01 Dec 2015 (Version 9.0), including Amend. 9.0 of 01 Dec 2015, Amend 1 of 19 May 09, Amend 2 of 11 NOV 09, Amend 3 of 15 NOV 10, Amend 4 of 21 DEC 10, Amend 5 of 27 APR 11, Amend 6 of 17 OCT 11, and Amend 7.1 of 02 Jul 2012, Amend 8.0 of 16 Oct 2013

The study will be conducted according to the protocol and in compliance with Good Clinical Practice (GCP), with the Declaration of Helsinki, and with other applicable regulatory requirements.

This document contains confidential information of Miltenyi Biotec GmbH
Do not copy or distribute without written permission from the Sponsor.
CONFIDENTIAL

SIGNATURE PAGE

Declaration of Sponsor

Title: INTRAMYOCARDIAL TRANSPLANTATION OF BONE MARROW STEM CELLS FOR IMPROVEMENT OF POST-INFARCT MYOCARDIAL REGENERATION IN ADDITION TO CABG SURGERY: a controlled prospective, randomized, double blinded multicenter trial

This study protocol was subjected to critical review. The information it contains is consistent with current knowledge of the risks and benefits of the investigational product, as well as with the moral, ethical and scientific principles governing clinical research as set out in the Declaration of Helsinki (2013) and the guidelines on Good Clinical Practice.

__________________________________ ____________________

Dr. med. Liane Preußner
Medical Director
Miltenyi Biotec GmbH, Bergisch Gladbach
Friedrich-Ebert-Straße 68
51429 Bergisch Gladbach
Germany

Date

Declaration of the National Coordinating Investigator and Leiter der klinischen Prüfung

Title: INTRAMYOCARDIAL TRANSPLANTATION OF BONE MARROW STEM CELLS FOR IMPROVEMENT OF POST-INFARCT MYOCARDIAL REGENERATION IN ADDITION TO CABG SURGERY: a controlled prospective, randomized, double blinded multicenter trial

This study protocol was subjected to critical review and has been approved by the Sponsor. The information it contains is consistent with the current risk/benefit evaluation of the investigational product as well as with the moral, ethical and scientific principles governing clinical research as set out in the Declaration of Helsinki (2013) and the guidelines on Good Clinical Practice.

National Coordinating Investigator and Leiter der klinischen Prüfung (according to German Medical Products Act)

______________________________	____________________
Prof. Dr. med. Gustav Steinhoff	Date

UNIVERSITÄT ROSTOCK
Direktor Klinik und Poliklinik für Herzchirurgie
Schillingallee 35
D-18057 Rostock

Phone: +49 (381) 494-6100

Fax: +49 (381) 494-6102

E-mail: gustav.steinhoff@med.uni-rostock.de

Declaration of the Trial Site Principal Investigator

Title: INTRAMYOCARDIAL TRANSPLANTATION OF BONE MARROW STEM CELLS FOR IMPROVEMENT OF POST-INFARCT MYOCARDIAL REGENERATION IN ADDITION TO CABG SURGERY: a controlled prospective, randomized, double blinded multicenter trial

All documentation for this study that is supplied to me and that has not been previously published will be kept in the strictest confidence. This documentation includes this study protocol, Investigator's Brochure, equipment for electronic data documentation, and other scientific data.

The study will not be commenced without the prior written approval of a properly constituted Independent Ethics Committee (IEC). No changes will be made to the study protocol without the prior written approval of the Sponsor and the IEC, except where necessary to eliminate an immediate hazard to the patients.

I have read and understood and agree to abide by all the conditions and instructions contained in this protocol.

Trial Site Principal Investigator

____

Name of Investigator in block letters or stamp

____________________________________ ____________________

Signature Investigator Date

PROTOCOL SYNOPSIS

**Title**	Intramyocardial TransPlantation Of BonE MaRrow Stem Cells For ImprovEment Of Post-Infarct MyoCardial RegeneraTion In Addition To CABG Surgery: a controlled prospective, randomized, double blinded multicenter trial. Short title – PERFECT
**Sponsor Study No.**	PERFECT 001 (M-2006-144)
**EudraCT No.**	2006-006404-11
**Phase**	III
**Sponsor**	Miltenyi Biotec GmbH, Bergisch Gladbach<br>Friedrich-Ebert-Straße 68<br>51429 Bergisch Gladbach<br>Germany
**National Coordinating Investigator and Leiter der klinischen Prüfung**	Prof. Dr. med. Gustav Steinhoff<br>Universität Rostock<br>Direktor, Klinik und Poliklinik für Herzchirurgie<br>Schillingallee 35<br>D-18057 Rostock<br>Phone: +49 (381) 494-6100<br>Fax : +49 (381) 494-6102<br>E-mail: gustav.steinhoff@med.uni-rostock.de
**Trial Sites**	Seven sites in Germany
**Objective(s)**	Primary objective: To determine whether injection of autologously-derived bone marrow stem cells yields a functional benefit in addition to the coronary artery bypass graft (CABG) operation as determined by left ventricular heart function (LVEF-MRI).<br>Secondary objectives: To determine the effects of an injection of autologously derived bone marrow stem cells on physical exercise capacity, cardiac function, safety and Quality of Life (QoL).
**Design**	This will be a placebo controlled, prospective, randomized, double-blind multicenter, phase III, clinical trial investigating the effects of intramyocardial injection of 5 ml $CD133^+$ bone marrow cells or placebo in 142 patients with coronary artery disease scheduled for CABG surgery. Patients will be randomized to one of the two treatment groups ($CD133^+$ or placebo) in a 1:1 ratio.<br>Patients will be required to attend 7 study assessments and 1 safety follow-up assessment after study closure:<br>1. Assessment I prior to the operation (screening)<br>2. Assessment Ia between day -2 and day of operation (day 0) (cell preparation and transfer)<br>3. Assessment II at day of operation (injection of study treatment)<br>4. Assessment IIa at day after operation (post OP/ICU)<br>5. Assessment III during the postoperative stay before or after discharge (within 72 hours of discharge)<br>6. Assessment IV (by telephone) at 3 months after the operation<br>7. Assessment V at 6-months after the operation (data closure)<br>8. Assessment VI safety up-date after study closure at 24 months after operation<br>At Assessment I the patient will be asked to sign the informed consent and baseline criteria will be assessed.<br>At Assessments I, III and V patients will undergo a 6-minute walk test (physical examinations and vital signs), Holter, 12-lead ECG, cardiac magnetic resonance imaging (MRI) and echocardiography, checking of unwanted tissue changes, laboratory including NT-proBNP and New York Heart Association (NYHA) and Canadian Cardiovascular Society (CCS) evaluations as well as listing of concomitant medications.

	Adverse events (AE) will be assessed continuously (informed consent to study end). Furthermore, major adverse cardiovascular events (MACE) will be assessed at Assessment V and VI. At Assessment IIa patients will undergo laboratory and 12-lead ECG evaluations at ICU.<br>At Assessments I, IV and V patients will be asked to complete QoL questionnaires.<br>Assessment Ia is related to randomization, cell-preparation and transfer and Assessment II is related to injections of study treatment.<br>The planned patient recruitment is 5 years, the planned duration of study, inclusive follow-up (6 months) is 5.5 years. The duration of the study for each patient will be approximately 6 months.<br>An obligatory safety follow up is planned for further 18 months after study data closure. Therefore the patients will be required to attend 24 months after the operation Assessment VI. This will be outside the study after data closure. At Assessment VI patient will undergo examination of all safety parameters and will be asked to complete QoL questionnaires.
**Treatment**	All patients will undergo bone marrow aspiration (150-200 ml) and withdrawal of 20 mL blood prior to CABG surgery.<br>Patients randomized to the active treatment group will be given 5 mL $CD133^+$ cells ($0.5\text{-}5x10^6$ cells) suspended in physiological saline + 10% autologous serum intramyocardially (divided into 15 injections) during CABG surgery.<br>Patients randomized to the placebo group will be given 5 mL physiological saline + 10% autologous serum solution intramyocardially (divided into 15 injections) during CABG surgery.
**Number of Patients**	142 patients (71 patients per group) will be enrolled to provide 60 evaluable patients in each treatment group (estimated drop-out rate 15%). When 70 patients have been included and completed the 6-month follow-up an interim analysis will be performed in a semi-blind manner by an independent statistician who is not involved in the conduct of the study to maintain the blindness of the treatment code.
**Population**	Patients will be entered into this study only if they meet all of the following inclusion criteria:<br>1. Coronary artery disease after myocardial infarction with indication for CABG surgery<br>2. Currently reduced global left ventricular ejection fraction (LVEF) assessed at site by cardiac magnetic resonance imaging (MRI) at rest (25% ≤ LVEF ≤ 50%)<br>3. Presence of a localized akinetic/hypokinetic/hypoperfused area of LV myocardium for defining the target area<br>4. Informed consent of the patient<br>5. 18 years ≤ Age < 80 years<br>6. Are not pregnant and do not plan to become pregnant during the study. Females with childbearing potential must provide a negative pregnancy test within 1-7 days before OP and must be using oral or injectable contraception (non childbearing potential is defined as post-menopausal for at least 1 year or surgical sterilization or hysterectomy at least 3 months before study start)<br>Patients will be entered into this study only if they meet none of the following exclusion criteria:<br>1. Emergency operation<br>2. Presence of any moderate-severe valvular heart disease requiring concomitant valve replacement or reconstruction<br>3. Medical History of recent resuscitation in combination with ventricular arrhythmia classified by LOWN ≥ class II<br>4. Acute myocardial infarction within last 2 weeks<br>5. Debilitating other disease: Degenerative neurologic disorders, psychiatric disease, terminal renal failure requiring dialysis, previous organ

transplantation, active malignant neoplasia, or any other serious medical condition that, in the opinion of the Investigator is likely to alter the patient's course of recovery or the evaluation of the study medication's safety

6. Impaired ability to comprehend the study information
7. Absence of written informed consent
8. Treatment with any investigational drug within the previous 30 days
9. Apparent infection (c-reactive protein [CRP] ≥ 20 mg/L, fever ≥ 38.5°C)
10. Contraindication for MRI scan
11. Immune compromise including active infection with Hepatitis B, C, HIV virus or seropositivity for Treponema pallidum
12. Pregnant or breast feeding
13. Childbearing potential with unreliable birth control methods
14. Have previously been enrolled in this study, respectively phase I and phase II
15. Known hypersensitivity or sensitization against murine products and human-anti-mouse-antibody-titer ≥ 1:1000
16. Contraindication to bone marrow aspiration
17. Known hypersensitivity against iron dextrane

Efficacy Parameters

Primary endpoint

The primary endpoint is LVEF at 6 months postoperatively, measured by MRI at rest. Cardiac MRI is established as the gold standard for determination of LV function (LVEF and LV volumes).

Secondary endpoints

1. Change in LVEF at 6 months post-OP compared with preoperatively (screening) and early postoperatively (discharge) as assessed by MRI and echocardiography.
2. Change in LV dimensions (left ventricular end systolic dimension [LVESD], left ventricular end diastolic dimension [LVEDD]) at 6 month post-OP compared with preoperatively (screening) and early postoperatively (discharge) as assessed by echocardiography.
3. Change in physical exercise capacity determined by 6 minute walk test at 6 months post-OP compared with preoperatively (screening) and early postoperatively (discharge).
4. Change in NYHA and CCS class at 6 months post-OP compared with preoperatively (screening) and early postoperatively (discharge).
5. MACE (cardiac death, myocardial infarction, secondary intervention/reoperation, ventricular arrhythmia).
6. QoL-score at 6 months post-OP compared with preoperatively (screening) and 3 months (telephone).

Safety Parameters

1. Recording of AEs
2. MACE (cardiac death, myocardial infarction, secondary intervention/reoperation, ventricular arrhythmia) and tachycardial supraventricular arrhythmia >160 bpm (Holter ECG).
3. Laboratory tests (post-operative check and specific tests for cell preparation)
4. Unwanted tissue changes (tumors) will be monitored by MRI and/or echocardiography
5. Vital signs (blood pressure and pulse)
6. Physical examination, 12-lead ECG

Safety parameters will be determined during the 7 study assessments.

An obligatory 2 years follow up will be conducted 18 months after data closure of the study (Assessment VI).

At Assessment VI (after finalization of the study) patients will undergo:

1. Recording of AEs
2. MACE (cardiac death, myocardial infarction, secondary intervention/reoperation, ventricular arrhythmia) and tachycardial supraventricular arrhythmia >160 bpm (Holter ECG).

3. Laboratory tests
4. Unwanted tissue changes (tumors) will be monitored by echocardiography
5. Vital signs (blood pressure and pulse)
6. Physical examination, 12-lead ECG
7. QoL-Score

Statistical Methods

The statistical analysis of study results will be performed according to the CPMP guidelines for “Biostatistical methodology in clinical trials in applications for marketing authorizations for medicinal products” and the ICH guideline “Statistical principles for clinical trials”.

For all data collected during the trial and reported in the case report forms describing the sample, listings of the individual raw data as well as tables of sample characteristics and/or frequencies will first be given. Continuous data will be summarized first by treatment group, secondly by certain study time points, thirdly by each study centers using descriptive statistics (mean, median, standard deviation [SD], minimum and maximum, number of available observations and number of missing observations). It will be done for absolute and percent changes from baseline too.

Categorical data will be summarized first by treatment group, secondly by certain study time points, thirdly by each study centers. The numbers and percentages of each of categories, the number of available observations and the number of missing observations will be presented in frequency tables.

The trial will be conducted at seven study sites. Therefore centers will be considered as possible prognostic factor influencing the outcome.

With regard to possible baseline and study site effects, the two-sided hypothesis for the continuous primary efficacy variable LVEF at 6 months postoperatively (comp. section 6.1.1) will be assessed using analysis of covariance (ANOVA) at adjusting for baseline LVEF and study site at a 5% level of significance.

Secondary efficacy variables (comp. section 6.1., 6.2.) will be analyzed in a strictly explorative way. If p-values are computed, no adjustment for multiple testing will be made and they will be interpreted in the exploratory sense. Similarly, confidence intervals will be computed. In order to check differences between the treatment groups for the fifth secondary endpoint (death, myocardial infarction, need for reintervention) an unadjusted survival analysis with Kaplan-Meier-estimations will be performed using the log rank test.

Adverse events (AEs) will be summarized by incidence, severity, outcome, and causal relationship to treatment and will be descriptively compared for the two treatment groups. Associated AE tables will present the total number of patients reporting at least one specific event and the maximum severity grade. Special tables will be displayed for serious AEs, MACE and for AEs leading to withdrawal. Separate summarizations of AEs by worst severity, and by relationship to treatment will also be provided.

Descriptive statistics for laboratory parameters will be presented by treatment group and time point. For continuous laboratory parameters, changes from baseline to the other time points will be presented by treatment group and descriptive statistics will be calculated. Values will also be presented according to the Common Terminology Criteria categories. Values outside corresponding normal ranges will be displayed and tabulated. Examination of the nature of the abnormalities will be performed if the rate of new or worsened abnormalities is deemed excessive.

Changes in vital signs and electrocardiograms will also be examined for treatment group differences.

Details of the statistical analyses will be documented in SAP (Statistical Analysis Plan) that is to be finalized before unblinding. An interim analysis will be performed on the first 70 patients randomized and followed-up for at least 6 month using the adaptive two-stage approach described by Bauer and Köhne 1994 The effect size of the primary efficacy parameter will be calculated in a semi-

	blinded manner. The sample size calculation for the second step will be performed using ADDPLAN (Wassmer und Eisebitt) The following secondary endpoints will be evaluated descriptively at the first stage: Change in LVEF by echo, change in physical exercise capacity determined by 6 minute walk test and change in NYHA and CCS class – all at 6 months post-OP compared with preoperatively (screening) and MACE. The trial will either be stopped for futility or continued after reassessment of the sample size.
**Schedule of Assessments**	See Table 2.

TABLE OF CONTENTS

PROTOCOL SYNOPSIS **5**

TABLE OF CONTENTS **10**

1 INTRODUCTION **16**

1.1 Background Information **16**
1.1.1 Investigational Product 16
1.1.2 Summary of Findings from Cell Research and Preclinical and Clinical Studies that are Relevant to the Trial 17
1.1.2.1 Cell Sources for Cardiac Cell Transplantation 17
1.1.2.2 Bone Marrow Cells and Angiogenesis 17
1.1.2.3 Bone Marrow Cells and Myogenesis 19
1.1.2.4 Preclinical and Clinical Trials Using Bone Marrow Mononuclear Cells 21
1.1.2.5 Previous Clinical Studies Using $CD133^+$ Bone Marrow Stem-Cells 22
1.1.2.6 Summary 27
1.2 Rationale **27**
1.3 Risk-Benefit Assessment **28**

2 STUDY OBJECTIVES **31**

2.1 Primary Objective **31**
2.2 Secondary Objectives **31**

3 OVERALL DESIGN AND PLAN OF THE STUDY **32**

3.1 Overview **32**
3.2 Endpoints **33**
3.3 Justification of the Study Design **33**

4 STUDY POPULATION **35**

4.1 Inclusion Criteria **35**
4.2 Exclusion Criteria **35**
4.3 Patient Withdrawal and Replacement **36**
4.4 Planned Sample Size and Number of Study Centers **36**
4.5 Patient Identification and Randomization **37**

5 STUDY TREATMENT **38**

5.1 Identity **38**
5.2 Administration **38**
5.3 Packaging, Labeling and Storage **39**
5.4 Blinding and Breaking the Blind **39**
5.5 Drug Accountability **41**
5.6 Compliance **41**
5.7 Concomitant Medications **41**

5.8 Bone Marrow Aspiration, Blood Collection and Transport 42
5.9 CD133+ Cell and Placebo Preparation and Treatment 42
5.9.1 CD133+ Cell Product Release criteria 43
5.9.2 Final formulation of CD133+ Cell Product and Placebo 43
5.9.3 Practices with final formulation of CD133+ Cell Product which do not fulfill the release requirements 44
5.10 Coronary Artery Bypass Graft (CABG) 44

6 PARAMETERS AND METHODS OF ASSESSMENT 45

6.1 Efficacy Parameters 45
If not stated otherwise, all examinations have to be performed at the trial site. However, routine examinations that are executed at the trial site before informed consent was obtained should not be repeated provided the respective examination has been performed according to trial protocol. In order to avoid unnecessary inconvenience and risk for the patient, this data may also be used as historic data. 45
6.1.1 Left Ventricular Ejection Fraction (LVEF) 45
6.1.2 Left Ventricular (LV) Dimensions 45
6.1.3 Classification of Heart Failure (NYHA)/Angina (CSS) 46
6.1.4 Heart Catheterization 46
6.1.5 Cardiac MRI Scan 46
6.1.6 Echocardiography 48
6.1.7 6-Minute Walk Test (6MWT) 48
6.2 Quality Of Life Questionnaires 49
6.2.1 Short Form Questionnaire (SF36) 49
6.2.2 Minnesota Living With Heart Failure Questionnaire (MLHF-Q) 49
6.2.3 Questionnaire of the EuroQoL group: EQ-5D™ 49
6.3 Safety Parameters 50
6.3.1 Adverse Events (AEs) 50
6.3.1.1 Definitions 50
6.3.1.2 Classifications 51
6.3.1.3 Monitoring, Recording and Reporting of Adverse Events 51
6.3.1.4 Follow-Up of Adverse Events 54
6.3.1.5 Adverse Events of Specific Interest 54
6.3.1.6 Major Adverse Cardiovascular Events – MACE 54
6.3.2 The Methods and Timing for Assessing and Recording Other Safety Parameters 54
6.3.2.1 Laboratory Parameters 55
6.3.2.2 Vital Signs 56
6.3.2.3 General Physical Examination 56
6.3.2.4 Holter and LOWN Classification 56
6.3.2.5 12-Lead Electrocardiogram 56
6.3.2.6 General Medical History 57
6.3.2.7 Cardiac Medical History 57
6.3.2.8 Risk Factors 58
6.3.2.9 Unwanted Tissue Changes 58

7 STUDY CONDUCT 58

7.1 Schedule of Observations 58
7.2 Observations by Visit 60
7.2.1 Screening (Assessment I; Day -7 to Day -1) 60
7.2.2 Bone Marrow Transfer /Preparation of Cell Product/Placebo (Assessment Ia; Day -2 to 0) 60
7.2.3 Intra OP: Surgical Operation (Assessment II; Day 0) 61
7.2.4 Post OP/Intesive Care Unit (Assessment II a; Day 0 to Day 4 approx.) 62
7.2.5 Hospital Discharge: (Assessment III; within 72 hrs of discharge) 62
7.2.6 Follow-Up Period 63
7.2.6.1 3-Month Follow-Up post OP (± 2 weeks, Assessment IV, per telephone) 63
7.2.6.2 6-Month Follow-Up post OP (± 2 weeks, Assessment V)/Early Termination 63
7.2.6.3 24-Month Follow-Up post OP (± 2 weeks, Assessment VI after data closure for safety reason) 64

8 STATISTICAL METHODS 65

8.1 Study Patients 65
8.1.1 Disposition of Patients 65
8.1.2 Protocol Deviations 65
8.1.3 Analysis Populations 66
8.2 General Considerations 66
8.3 Demographics, Baseline Characteristics and Concomitant Medications 67
8.4 Treatment and Study Compliance 67
8.5 Efficacy Analyses 67
8.6 Safety Analyses 67
8.7 Determination of Sample Size 68
8.8 Interim Analysis 70

9 ETHICAL, LEGAL AND ADMINISTRATIVE ASPECTS 72

9.1 Data Quality Assurance 72
9.2 Case Report Forms and Source Documentation 72
9.3 Access to Source Data 73
9.4 Data Processing 73
9.5 Archiving Study Records 73
9.6 Good Clinical Practice 73
9.7 Informed Consent 74
9.8 Protocol Approval and Amendment 74
9.9 Safety Monitoring Board 74
9.10 Duration of the Study 75
9.11 Premature Termination of the Study 75
9.12 Confidentiality 76
9.13 Other Ethical and Regulatory Issues (Optional) 76

9.14 Liability and Insurance....76
9.15 Publication Policy....76

10 REFERENCE LIST....78

11 APPENDICES....83

Appendix 1: Trial Flow Chart....84
Appendix 2: Adverse Events of Specific Interest....85
Appendix 3: Short Form Questionnaire (SF36)....86
Appendix 4: Minnesota Living with Heart Failure Questionnaire (MLHF)....90
Appendix 5: Questionnaire of the EuroQoL group: EQ-5D™....92

Tables in Text

Table 1: Laboratory Assessments....55
Table 2: Schedule of Assessments....59
Table 3: Number of Patients Necessary to Show Differences of the Primary Efficacy Parameter LVEF at 6 Months Postoperatively (Two-Sided, α = 0.05, 1 – β = 0.90)....69
Table 4: Power with N=60 Patients per Treatment Group for Several Differences of Means and Standard Deviations....69

Figures in Text

Figure 1: Current Strategies of Stem Cell Application to the Heart....16
Figure 2: Echocardiographic Data....24
Figure 3: Change in LVEF....25
Figure 4: SPECT Scans....26
Figure 5: Left ventricular segmentation....39
Figure 6: Scheme of manufacturing process....43
Figure 7: Left ventricular segmentation....47
Figure 8: Reporting of Serious Adverse Events....52

List of Abbreviations and Definitions of Terms

6MWT	6-Minute Walk Test
AMI	Acute Myocardial Infarction
AR	Adverse Reaction
AE	Adverse Event
AHA	American Heart Association
CABG	Coronary Artery Bypass Graft
CCS	Canadian Cardiovascular Society
CHMP	Committee for Medicinal Products for Human use
CK	Creatine Kinase
CK-MB	Creatine Kinase MB
CPMP	Committee for Proprietary Medicinal Products
CRF	Case Report Form
CRO	Contract Research Organization
CRP	C-Reactive Protein
CTCAE	Common Terminology Criteria for Adverse Events
DB	Data Base
DT	Deceleration Time
ECG	Electrocardiogram
EMEA	European Medicines Agency
FAS	Full Analysis Set
GCP	Good Clinical Practice
GMP	Good Manufacturing Practice
HBV	Hepatitis B virus
HCV	Hepatitis C virus
HIV	Human Immunodeficiency Virus
HSA	Human Serum Albumin
IB	Investigator Brochure
ICH	International Conference on Harmonization
ICSR	Individual Case Safety Reports
ICU	Intensive Care Unit
IEC	Independent Ethics Committee
IMP	Investigational Medicinal Product
ITT	Intent-to-treat
IVSD	Inter Ventricular Septum Diameter
LA	Left Atrium
LKP	Leiter der Klinischen Prüfung
LMCA	Left Main Coronary Artery

LV	Left Ventricular
LVEDD	Left Ventricular End Diastolic Dimension
LVEDV	Left Ventricular End Diastolic Volume
LVEF	Left Ventricular Ejection Fraction
LVESD	Left Ventricular End Systolic Dimension
LVPWD	Left Ventricular Posterior Wall Diameter
MACE	Major Adverse Cardiovascular Events
MP	Manufacturing Procedure
MRI	Magnetic Resonance Imaging
NT-pro BNP	N-Terminal pro-B-typeNatriuretic Peptide
NYHA	New York Heart Association
PBS	Phosphate Buffered Saline
PEI	Paul-Ehrlich-Institut
PPS	Per Protocol Set
QoL	Quality of Life
RA	Right Atrium
RCA	Arteria Coronaria Dextra
RCX	Ramus Circumflexus
RIVA	Ramus Interventricularis Anterior
RVEDD	Right Ventricular End Diastolic Dimension
RVOT	Right Ventricular Outflow Tract
SAE	Serious Adverse Event
SMB	Safety Monitoring Board
SAP	Statistical Analysis Plan
SAR	Serious Adverse Reaction
SOP	Standard Operating Procedure
SUSAR	Suspected Unexpected Serious Adverse Reaction
SVES	Supraventricular Extrasystoles
TPP	Treated Per Protocol
VA	Late (diastolic peak flow) Velocity
VE	Early (diastolic peak flow) Velocity
VES	Ventricular Extrasystoles

1 INTRODUCTION

1.1 Background Information

1.1.1 *Investigational Product*

In the setting of acute myocardial infarction, several studies have shown a functional benefit of intracoronary infusion of bone marrow cells compared with the standard treatment alone (Wollert and Drexler, 2005; Strauer et al, 2002; Schächinger et al, 2005 and 2006), but patients with chronic ischemic heart disease and impaired ventricular function may require a different approach. Therefore, our group developed a protocol for intramyocardial injection of purified $CD133^+$ bone marrow stem cells directly into the diseased myocardium of patients after myocardial infarction at the time of coronary artery bypass surgery. In this study, purified $CD133^+$ bone marrow stem cells will be compared to placebo (physiological saline with 10% autologous serum). Based on the encouraging results in the first 6 patients (Stamm et al, 2003), we completed a dose-escalation safety trial and then conducted a controlled study to determine efficacy compared with the standard CABG operation (Stamm et al, 2004 and 2007). Our injection pathway is demonstrated in Figure 1 as compared to alternative current strategies of bone marrow stem cell transplantation in the heart (catheter based intracoronary and endocardially).

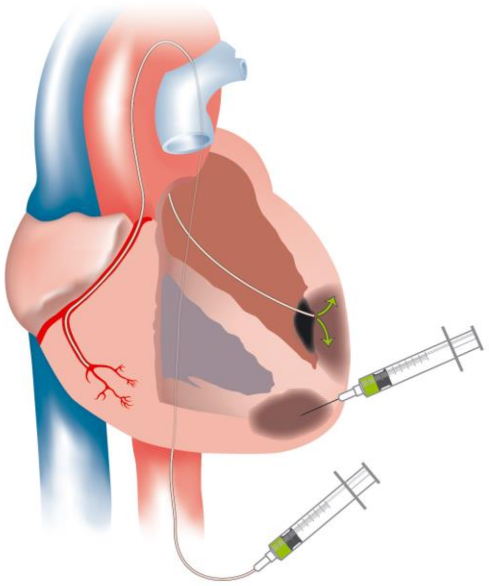

Figure 1: Current Strategies of Stem Cell Application to the Heart

Intracoronary infusion, transendocardial and epicardial intramyocardial injection

1.1.2 *Summary of Findings from Cell Research and Preclinical and Clinical Studies that are Relevant to the Trial*

1.1.2.1 Cell Sources for Cardiac Cell Transplantation

Contractile cell types such as allogenic cardiomyocytes, skeletal myoblasts, or smooth muscle cells have been shown to survive in areas of myocardial necrosis and to improve local contractile function in various small and large animal models. Skeletal muscle progenitor cells or satellite cells can be isolated from skeletal muscle and expanded in culture, and several studies have demonstrated that implantation of skeletal myoblasts in an area of infarcted myocardium improves regional contractility. In 2001 Menasche et al. reported the first clinical application of this concept in a patient who also underwent a coronary artery bypass graft (CABG) operation. The differentiation of skeletal myoblasts into cardiac cells, however, is not possible. Therefore complete myocardial regeneration cannot be achieved by skeletal myoblasts.
Another source of cells for cardiac regeneration are early precursor and adult stem cells, which can be found in the bone-marrow, heart and other organs (Leri et al, 2005). Persistent uncommitted stem cells in the adult organism have a large regeneration potential and are able to migrate and repair diseased tissue including the myocardium (Asahara et al, 1997 and 1999; Krause et al, 2001; Jiang et al., 2002). From basic research, preclinical studies and clinical trials it has become evident that bone-marrow derived stem cells contain a therapeutic efficacy for the regeneration of heart function after infarction due to angiogenesis (Leri et al, 2005).

1.1.2.2 Bone Marrow Cells and Angiogenesis

During embryonic development the primary vascular plexus is formed by hemangioblasts, stem cells capable of generating both hematopoietic progeny and endothelial cells, in a process termed vasculogenesis. Further blood vessels are generated by both sprouting and non-sprouting angiogenesis, finally leading to the complex functional adult circulatory system (Risau, 1997). Until recently only two mechanisms of postembryonic vascular remodeling have been recognized. Angiogenesis, the proliferative outgrowth of local capillaries, is one way to reinforce perfusion. Angiogenesis can be induced by various conditions, including ischemia. In case of myocardial ischemia caused by the occlusion of a coronary artery, preexisting small collateral vessels also bear the capacity to enlarge in a process termed arteriogenesis. It has been assumed for long time that both mechanisms are mainly dependent on local proliferation of resident cells. The advent of cellular therapy of ischemic organ damage has introduced neo-angiogenesis (sometimes also called vasculogenesis) by immigration of stem and progenitor cells as a third possible mechanism resulting in improved perfusion of the adult damaged heart. Accumulating evidence indicates that immigrating (stem) cells can truly differentiate along the endothelial lineage but also can provide paracrine support in these three courses of action during regenerative vascular remodeling (Kinnaird et al, 2004; Rafii and Lyden, 2003; Urbich and Dimmeler, 2004).

A link between hematopoietic stem cells and angiogenesis has been established several years ago. Takakura et al. (2000) determined the angiogenetic effects of hematopoietic stem cell transplantation in embryos with incomplete angiogenesis. Putative progenitors for therapeutic angiogenesis have been isolated from adult human peripheral blood based

on their expression of CD34, a marker molecule shared by microvascular endothelial cells and hematopoietic stem cells (Asahara et al, 1997). The same group provided the proof of concept by transplantation of genetically marked mouse bone marrow into recipient mice that were subsequently subjected to five distinct models of vascular remodeling including myocardial ischemia (Asahara et al, 1999). In this particular system, transgenic mice constitutively expressing beta-galactosidase under the transcriptional regulation of an endothelial cell-specific promoter were used as donors to replace the bone marrow in the recipient animals. The transplanted cells were detected and identified as bone marrow-derived endothelial progenitor cells in reproductive organ tissues as well as in healing cutaneous wounds one week after punch biopsy. Marrow-derived endothelial progenitor cells were found to incorporate into capillaries among skeletal myocytes in an additional test for peripheral post-ischemic regeneration after hindlimb ischemia, as well as into foci of neovascularization at the border of an infarct after permanent ligation of the anterior descending artery (Asahara et al, 1999). Most importantly, direct injection of the bone marrow mononuclear cell fraction in rat models of myocardial ischemia increased the capillary density (Tomita et al, 1999; Kobayashi et al, 2000). Analysis of the effects of blood and bone marrow derived mononuclear cell implantation into ischemic myocardium in pigs further revealed that the stem cell effects are not limited to angiogenesis and improved collateral perfusion, but also include the supply of regulatory cytokines (Kamihata et al, 2001; Kamihata et al, 2002). However, concerns exist regarding limited efficiency owing to the limited number of SC in small sample volumes of non-enriched blood and BM that are delivered intra-myocardially and the risk of foreign tissue differentiation following local stroma cell injections. Kocher et al. (2001) circumvented this problem by using positively selected $CD34^{+}/133^{+}$ cells from human donors after stem cell mobilization with G-CSF for intravenous injection after permanent ligation of the left anterior descending coronary artery in nude rats, resulting in a five fold increase in the number of capillaries compared to control. As a result of the stem cell mediated angiogenesis, which was attributed to the content of marrow-derived angioblasts, the authors also found an approximately 20% increase of left ventricular ejection fraction and cardiac index together with a reduced severity of ventricular remodeling in human CD34-treated compared to control ischemic animals (Kocher et al, 2001).

Another cell population candidate for the regeneration of ischemic cardiac muscle and vascular endothelium are $CD45^{+}$ hematopoietic $CD34^{LOW/-}$/c-kit^{+}, so called side population stem cells with a specific Hoechst 33342 DNA dye efflux pattern (Goodell et al, 1997; Jackson et al, 2001). Orlic et al. (2001) used an alternative method to enrich putative regenerative stem cells for local application by depleting unwanted cell lineages prior to enrichment for the expression of the stem cell factor receptor c-kit from murine bone marrow. Thus concentrated cells, considered to represent hematopoietic stem cells, were observed to incorporate not only into vascular structures but dominantly led to myocardial regeneration (Orlic et al, 2001). Subsequent experiments by this group employed mobilization of stem cells by G-CSF prior to experimental myocardial infarction, which also led to a significant increase in vascular density within the scar, a reduction in mortality, and a significant reduction in infarct size (Orlic et al, 2001).

Although the evidence that angiogenesis occurs in ischemic myocardium is convincing, this new therapeutic option also has a potential for serious side effects (Epstein et al,

2001). Most importantly, bone marrow-derived endothelial cells were found as part of the tumor neo-vasculature in experimental colon cancer (Asahara, 1999). This finding might suggest a risk to trigger the growth of silent tumors by systemic use of pro-angiogenic stem cell therapy.

1.1.2.3 Bone Marrow Cells and Myogenesis

While the pro-angiogenic effect of marrow-derived stem cells appears to be well established, stem cell mediated myogenesis remains a matter of debate. The traditional view implies that ischemic damage to the myocardium can only be compensated by hypertrophy, not hyperplasia, of surrounding cardiomyocytes. This dogma has recently been challenged, and intra-myocardial as well as extra-myocardial sources of regenerating contractile cells have been suggested (Mathur and Martin, 2004). Cardiomyocyte proliferation has been described, although only with minute frequency (Kaistura et al, 1998; Beldrami et al, 2001). Furthermore, the existence of cardiomyocytes of non-cardiac origin has been suggested by chimerism analyses after transplantation (Quaini et al, 2002; Laflamme et al, 2002; Muller et al, 2002), but the biologic relevance of some of these data has been questioned (Spangrude et al, 2002; Bianchi et al, 2002).

Makino et al. (1999) isolated a cardiomyogenic cell line from murine bone marrow stromal cells that were treated with 5-azacytidine and screened for spontaneous beating. Those cells connected with adjoining cells, formed myotube-like structures, and beat spontaneously and synchronously. They expressed various cardiomyocytes-specific proteins, had a cardiomyocyte-like ultrastructure, and generated sinus node-like as well as ventricular cardiomyocyte-like action potentials. Several groups implanted bone marrow stromal cells in rat and mice hearts. Some showed that they become integrated in cardiac myofibers, assume the phenotype of native cardiomyocytes, express connexins and form gap junctions with host cells (Leri et al, 2005). Others questioned this (Murry et al, 2004; Balsam et al, 2004). Toma et al. (2002) isolated human mesenchymal stem cells from bone marrow of volunteers, injected those into the left ventricle of immunodeficient mice, and found that they also assume cardiomyocyte morphology and express various cardiomyocyte-specific proteins.

The notion that bone marrow cells can regenerate infarcted myocardium led to great excitement. In their landmark paper, Orlic et al. (2001) described that injection of genetically labeled murine Lin^{NEG}/c-kit^{+} stem cells isolated from mouse bone marrow by depletion of committed cells, and further enriched for expression of c-Kit led to the formation of new myocardium, occupying two thirds of the infarct region within 9 days. This paper initiated a wave of enthusiasm, but also critical discussion. Kocher et al. (2001) used a rat model of myocardial infarction and implanted human bone marrow cells that contained cells resembling embryonic hemangioblasts. They found evidence for neoangiogenesis in the infarct tissue, associated with beneficial effects on remodeling in the peri-infarct zone and a sustained improvement of left ventricular (LV) function. The data were interpreted to indicate trans-differentiation of adult hematopoietic stem cell by crossing lineage boundaries (Korbling and Estrov, 2003). However, the fact that cells are derived from bone marrow does not necessarily prove that they are hematopoietic in origin, especially in the light of growing knowledge about mesenchymal, non-hematopoietic stem cells within the marrow. The recognition of cell fusion as a common phenomenon in some artificial transplant models for regeneration of ischemic tissue has

added to the controversy (Goodell, 2003; Camargo et al, 2004). From the clinicians point of view this was no surprise, since cell fusion is an intrinsic characteristic of contractile cells. Multinucleated skeletal myotubes are a classic example of cell fusion, and cardiomyocytes have long been known to form a large syncytial union.

More serious concerns were produced by two publications, which could not reproduce the promising in vivo trans-differentiation data. Using a modified Lin^+ depletion protocol for stem cell enrichment in an otherwise similar myocardial ischemia model, Balsam et al. (2004) found abundant GFP^+ cells in the myocardium after 10 days, which nearly disappeared until day 30. The remaining donor cells lacked cardiac tissue-specific markers, and instead adopted only hematopoietic fates as indicated by the expression of CD45. Murry et al. (2004) used both cardiomyocyte-restricted and ubiquitously expressed reporter transgenes to follow murine Lin^{NEG}/c-kit^+ stem cells after transplantation into healthy and injured mouse hearts, and could not find evidence for relevant differentiation into cardiomyocytes. In defense of the initial paper some have argued that i) the cell isolation protocols were not completely identical, and ii) both groups nevertheless observed some functional improvement in cell-treated hearts. However, it can not been denied that the evidence for myogenesis based on hematopoietic adult stem cells myogenesis is extremely controversial (Mathus and Martin, 2004; Chien, 2004; Couzin and Vogel, 2004; Honold et al, 2004). Very recently, a direct side-by-side comparison of human $CD133^+$ bone marrow cells and human skeletal myoblasts in a myocardial ischemia model in immunoincompetent rats demonstrated similar functional improvement in both groups, although only the myoblasts reached robust engraftment. Our own studies underline the angiogenic capacity of $CD133^+$ stem cells from adult human bone marrow and cord blood in a Scid-mouse myocardial infarction model (Ma et al, 2006). Moreover both cell preparations had beneficial effect on postinfarction mortality and apoptosis. Only adult bone marrow preparations contained a higher c-kit population and caused cardiac functional restoration in echocardiography. These findings underscore our limited understanding of how stem cells can elicit an improvement of heart function.

In contrast, the myogenic potential of stroma cell-derived mesenchymal stem cells is much better documented. Stroma cells are usually isolated based on their ability to adhere to plastic, not by selection for expression of certain surface markers. Their number in primary marrow aspirates is low, but they readily multiply for numerous cycles in culture, without apparent genotypic and phenotypic changes. Several years ago, Wakitani et al. (1995) reported the in vitro development of myogenic cells from rat bone marrow mesenchymal stem cells exposed to the DNA-demethylating agent 5-azacytidine. Furthermore, Makino et al. (1999) isolated a cardiomyogenic cell line from murine bone marrow stromal cells that were treated with 5-azacytidine and screened for spontaneous beating. Those cells connected with adjoining cells, formed myotube-like structures, and beat spontaneously and synchronously. They expressed various cardiomyocytes-specific proteins, had a cardiomyocyte-like ultrastructure, and generated several types of sinus node-like and ventricular cell-like action potentials. When isogenic marrow stromal cells are implanted in rat hearts, they appear to become integrated in cardiac myofibers, assume the histologic phenotype of cardiomyocytes, express connexins, and form gap junctions with native cardiomyocytes (Chedrawy et al, 2002; Wang et al, 2000). Again, epigenetic modification with 5-azacytidine is believed to facilitate differentiation towards

a cardiomyocyte phenotype in vivo (Bittira et al, 2002). Human mesenchymal stem cells derived from the marrow of volunteers have also been injected in hearts of immunodeficient mice, and again it was observed that they assume cardiomyocyte morphology and express various cardiomyocyte-specific proteins (Toma et al, 2002).
Under different cultivation conditions, mesenchymal stem cells readily assume an osteoblast, chondrocyte, or adipocyte phenotype. In fact, preclinical research on regeneration of skeletal components is much more advanced than that on cardiovascular applications. It is therefore no surprise that, when unmodified mesenchymal stem cells are implanted the heart, they may form islets resembling chondrogenic or osteogenic tissue. To date, there is very little, if any, information on stroma cell surface markers that might be helpful in identifying subpopulations with a particular potential for myogenic differentiation. It is therefore still unclear whether unmodified stroma cells that were expanded in vitro following simple isolation by plastic adherence will ultimately be useful in clinical protocols, whether a certain pro-myogenic subpopulation will be identified, or whether epigenetic re-programming prior to implantation will be necessary for functionally relevant myocardial regeneration in humans.

1.1.2.4 Preclinical and Clinical Trials Using Bone Marrow Mononuclear Cells

Probably the simplest approach to myocardial cell therapy in the clinical setting is the transfer of bone marrow mononuclear cells into the myocardium. The proponents of this approach argue that by using unmodified marrow or unselected mononuclear cells, the “ideal” cell for myocardial regeneration, which has not yet been identified, is not lost during the preparation process. Conversely, opponents argue that the vast majority of the bone marrow mononuclear cells are blood cells of all lineages and their immediate progenitors, while only few cells formally meet the stem cell criteria. Whether the local concentration of relevant stem- or progenitor cells will surpass the hypothetical threshold for induction of regeneration processes remains unclear. Indubitably, marrow mononuclear cells can be easily collected and prepared during a standard CABG operation, which is an obvious and important logistic advantage.
CABG patients were among the first to be included in clinical trials of cell therapy for myocardial regeneration. The most obvious reason is that the infarcted myocardium can be readily accessed during the operation, a unique opportunity to deliver cells in the center or the border zone of the infarcted tissue by rather simple means. The first such report came from Yamaguchi University, Japan. Hamano and colleagues described 5 patients who underwent CABG simultaneously bone marrow collection from the iliac crest (Hamano et al, 2001). The mononuclear cell fraction was prepared using a commercially available apheresis system, and between 5 and 22 injections of $5x10^7$ to $1x10^8$ cells were performed in the ischemic myocardium that was not directly revascularized by bypass grafting. In 3 of those 5 patients, improved perfusion of the cell-treated tissue was noted postoperatively. No complications such as arrhythmia or local calcification were noted, but no statement was made with respect to LV function. In a similar trial, Galinanes and colleagues from Leicester University, UK, collected marrow by sternal bone aspirate at the time of CABG surgery (Galinanes et al, 2004). This was diluted with autologous serum and injected into LV scar tissue. Postoperatively, regional contractility in LV wall segments that did or did not receive marrow cells was assessed by dobutamine stress echocardiography, and only the segmental wall motion score of the areas injected with bone marrow and receiving a bypass graft in combination

improved upon dobutamine stress. Most likely, many more patients have been subjected to similar treatment protocols elsewhere, but very little or no information as to the functional outcome is available. Most importantly, no controlled trial has so far demonstrated the superiority of CABG and mononuclear cell injection over CABG alone.

***Preclinical and clinical results using selected bone marrow stem cells*:** Our own group has focused on the intramyocardial injection of purified hematopoietic bone marrow stem cells since 2001 (Stamm et al, 2003). We chose not to simply inject an unmodified mononuclear bone marrow cell suspension, because the large number of leukocytes and their progenitors may primarily induce local inflammation, rendering the actual stem cell effects insignificant. Instead, we prepare a purified stem cell suspension using clinically approved methods. Two monoclonal antibodies are currently available for clinical selection of bone marrow stem cells, anti-CD34 and anti-CD133. Approximately 60 – 70% of the $CD34^+$ bone marrow cells co-express the CD133 antigen, and 70 - 80% of the $CD133^+$ cells are $CD34^+$ as well. The $CD133^+$ bone marrow cell population contains a small proportion of clonogenic cells, which have a very high potential to induce neoangiogenesis (Peichev M et al, 2000). Furthermore, there is accumulating evidence that the $CD133^+$/$CD34^-$-subpopulation includes multipotent stem cells with a significant potential for differentiation into mesenchymal and other non-hematopoietic lineages. Between 2001 and 2003, we conducted a formal phase-I safety and feasibility trial in 15 patients, including a dose-escalation protocol. Since 2003, an open-label controlled phase-II trial is being undertaken, that will eventually include 100 patients. Fifty patients will undergo CABG & intramyocardial stem cell delivery, and 50 patients with comparable characteristics will have CABG alone. The inclusion criteria were defined as follows: 1. documented transmural myocardial infarction more than 10 days and less than 3 months prior to admission for surgery; 2. presence of a localized area of akinetic LV wall without paradoxical systolic movement that corresponded with the infarct localization; 3. the infarct area should not be amenable to surgical or interventional revascularization; 4. elective CABG indicated to bypass stenoses or occlusions of coronary arteries other than the infarct vessel; 5. Absence of severe concomitant disease (i.e. terminal renal failure, malignoma, debilitating neurological disease). Patients who underwent emergency operation for unstable angina, reoperations, concomitant valve procedures, or had a history of significant ventricular arrhythmia are excluded. In our experience, it proved rather time-consuming to recruit patients who met the inclusion criteria (approximately 10 patients per year), probably because the modern rapid catheter interventions in acute myocardial infarction prevent the development of completely akinetic LV wall areas in many patients (Stamm et al, 2004).

1.1.2.5 Previous Clinical Studies Using $CD133^+$ Bone Marrow Stem-Cells

Since feasibility and safety of stem cell transplantation into the human heart remain to be determined, we initiated a phase I study of autologous bone marrow stem cell injection into the myocardial infarct border zone together with coronary artery bypass grafting in July 2001 (Stamm et al, 2003). Following injection of $CD133^+$ bone marrow cells in the peri-infarct zone in conjunction with CABG surgery in 11 patients, we saw significantly improved global LV function as well as improved perfusion of the infarcted myocardium (Stamm et al, 2004). Whether these beneficial effects are a consequence of the cell transplantation, the CABG surgery, or a combination of both remains unclear at this

point. However, the most important finding of our study is that there were no procedure-related complications up to 18 months postoperatively, especially no new ventricular arrhythmia or neoplasia (Stamm et al, 2007).

Clinical results of intramyocardial CD133$^+$ bone marrow stem cell transplantation with CABG surgery after myocardial infarction (Rostock trials)

***Safety trial**:* All 15 patients included in the dose-escalation safety trial tolerated the operation well and had a postoperative course without major complications. The dose escalation ranged from 0.5 to 5x10^6 CD133$^+$ bone marrow stem cells for intramyocardial transplantation. Minor complications were a rethoracotomy for bleeding from the internal mammary artery on the day of surgery and a respiratory tract infection in two patients. Otherwise, the in-hospital reconvalescence was uneventful, and all patients were referred to a cardiac rehabilitation program during the third postoperative week. Follow-up time currently ranges between 50 and 75 months and encompasses a total of 840 patient months (at August 2007). No relevant ventricular arrhythmia was recorded at any time point by online telemetric monitoring or Holter ECG, and the reported exercise tolerance improved in all patients. A 75 year old patient with cerebrovascular disease was lost to follow-up 9 months after surgery. He died of a stroke at later term (no autopsy available). All other patients were alive and well (Stamm et al, 2007). The echocardiographic data of the safety trial patients are depicted in Figure 2. Overall, the average LVEF rose from 39.0±8% to 50.1±9% at 6 months and 48±6% at 18 months (p=0.001), and LVEDV decreased from 147.9±38% to 126.4±29 and 127.2±18%, respectively (p=0.1). Myocardial perfusion was assessed by Thallium SPECT scans. The activity in the area at risk - expressed as the quotient postoperative-to-preoperative activity - demonstrated improved perfusion in the previously non- or hypoperfused infarction zone in 13 patients. The average perfusion ratio after CABG & CD133$^+$ cell injection was 1.15 ± 0.1 at 2 weeks (p=0.0001), 1.14 ± 0.2 at 6 months (p=0.02), and 1.14 ± 0.288 at 18 months p=0.07).

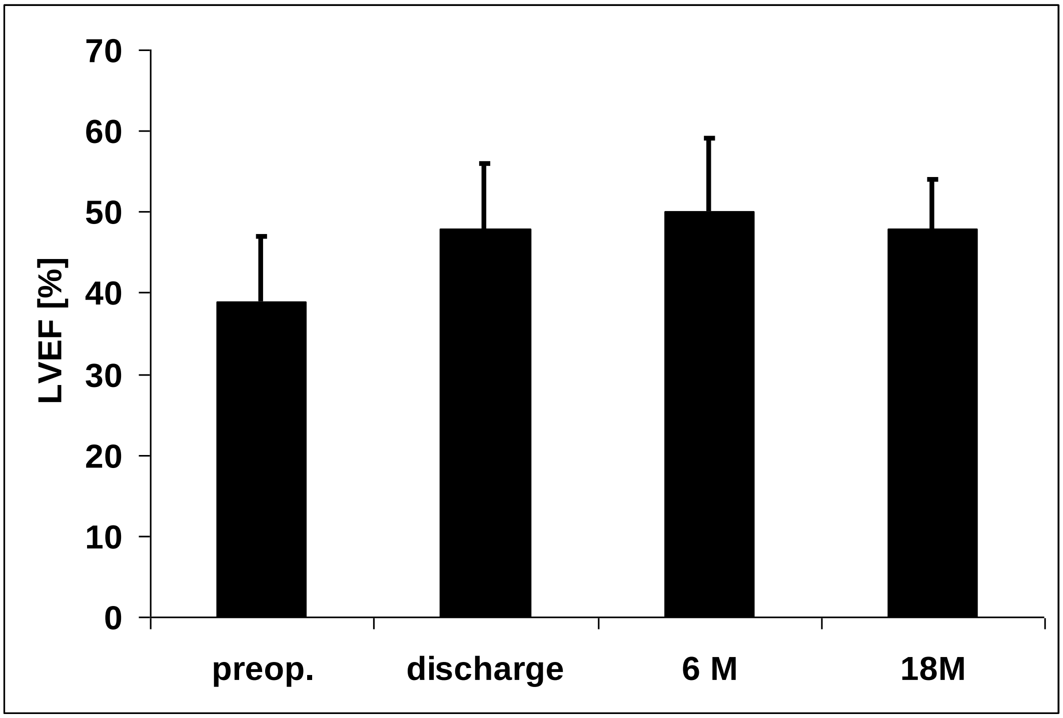

Figure 2: Echocardiographic Data

Echocardiographic data of patients included in the safety trial (n=15). Compared with the preoperative (preop) data, left ventricular ejection (LVEF) fraction rose significantly in response to CABG and $CD133^+$ cell injection ($p < 0.05$) at discharge, 6 and 18 months.

Efficacy trial*:* Forty patients were randomly assigned to undergo either CABG & cell injection or CABG only. All but one patient who remained in the study had an uneventful postoperative course. The only early postoperative complication was a low cardiac output syndrome with acute renal failure in one patient, requiring medium-dose catecholamnie treatment and temporary hemofiltration. This patient recovered completely and was transferred to the ward on postoperative day 6. During the follow-up period, no major adverse events (death, myocardial infarction, or cardiac re-intervention) were reported, and all patients were alive and well at most recent follow-up (Dec 31, 2005).

The echocardiographic data on LV function summarized in the data relevant for the primary outcome parameter, “LVEF at 6 months” are depicted in Figure 3. The average LVEF rose from 37.4±8% to 47.1±8% at 6 months in patients receiving CABG & cell injection (p=0.005), and from 37.9±10% to 41.3±8% in patients undergoing CABG only (p=0.47). As required by the study protocol, direct comparsion of the primary outcome parameter (average LVEF at 6 months) achieved a p-value of 0.04. Within the range of probablity defined by the statistical power, the null hypothesis is therefore rejected, indicating that CABG & cell injection resulted in better LVEF than CABG only. The average change in LVEF was +9.7% in CABG & cell injection patients and +3.4% in CABG only patients (p=0.0009).

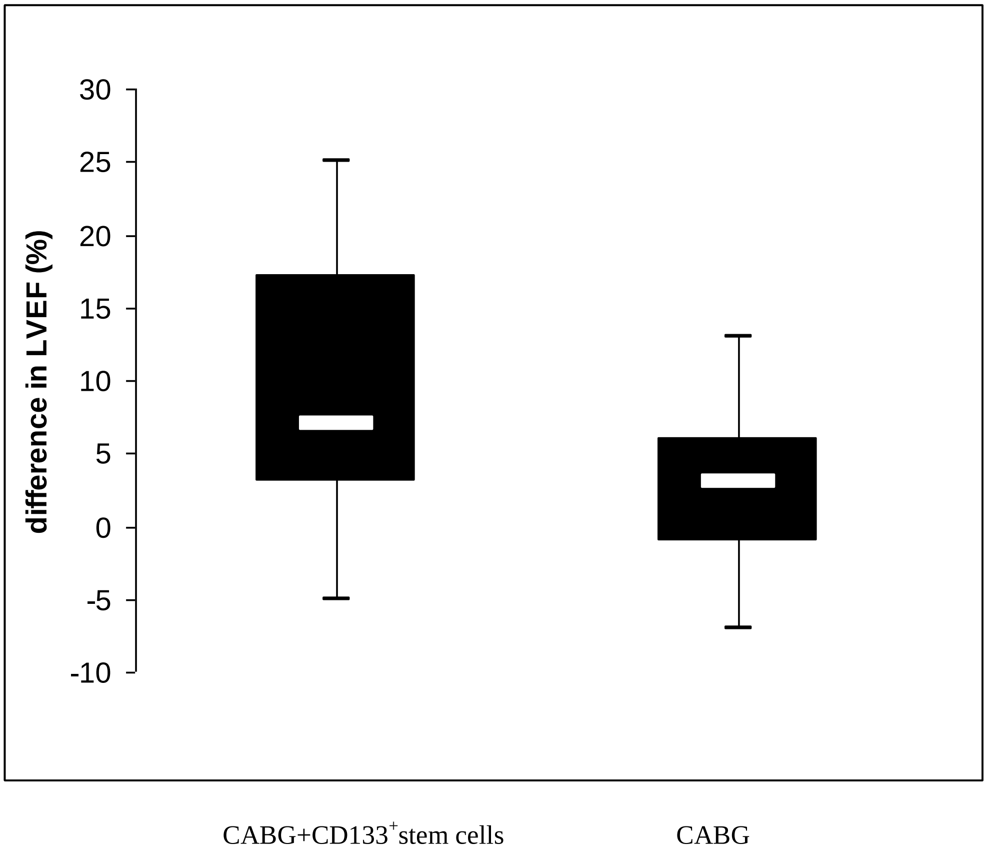

Figure 3: Change in LVEF

Change in LVEF (6 months follow up – preoperative value) in patients undergoing CABG and $CD133^+$ cell injection (CABG & cells) compared with those undergoing CABG alone (CABG-only). The average gain in LVEF was 9.7% in CABG & cell patients, and 3.4% in CABG-only patients (p=0.009). Box plots indicate 25th and 75th percentile (solid box), median (white bar), and minimum and maximum value of each dataset (whiskers).

As determined by SPECT imaging, myocardial perfusion in the area-of-interest at 6 months had improved in 4 control patients and in 11 patients who were treated with CABG & cell injection (p<0.05 by chi-square test). Overall, improvement of perfusion was greater in the CABG & cell injection group than in the CABG only group (median [25th-75th percentile] = 0.95 [0.91-1.03] for CABG only patients vs. 1.02 [0.95-1.1] for CABG & cell injection patients). Figure 4 depicts representative perfusion scans from a patient who received 5×10^6 $CD133^+$ selected cells in the border zone of a posterior transmural myocardial infarction, where no bypass graft could be placed. It is evident that at the time of discharge there was no relevant improvement, but perfusion of the ischemic tissue had virtually normalized 4 months later. This secondary gain in tissue blood supply might be attributed to the cell injection.

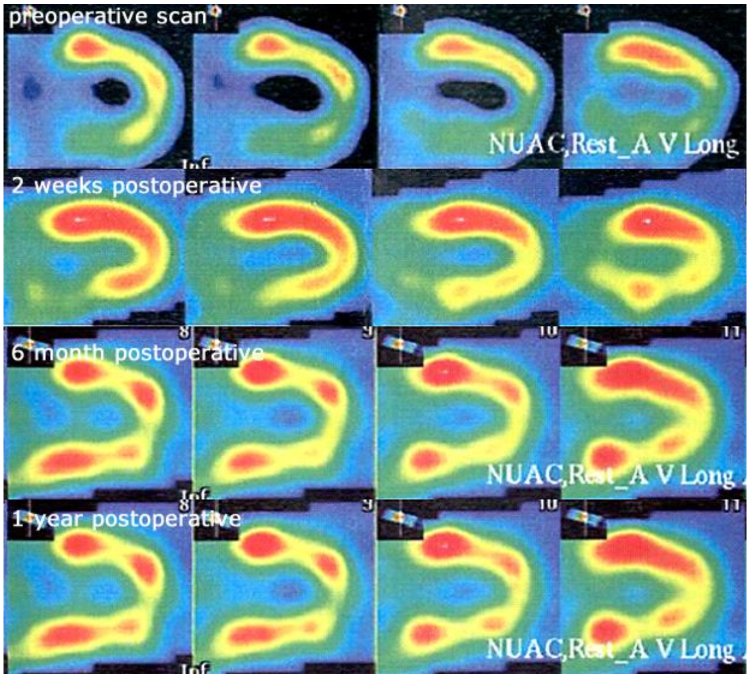

Figure 4: SPECT Scans

Representative SPECT scans of a patient in the safety trial who underwent bypass grafting to the left anterior descending coronary artery (LAD) and its branches, as well as injection of 5×10^6 $CD133^+$ selected cells in the posterior infarct area. At 6 months (3rd row) perfusion had virtually normalized.

The evaluation of the effects of $CD133^+$ bone marrow cells upon direct intramyocardial transplantation in chronically ischemic hearts of patients undergoing coronary artery bypass grafting gives primary clinical evidence of safety and efficacy of the treatment (Stamm et al, 2007). In the initial safety trial, no stem cell related complications were observed during up to six years follow-up. Left ventricular function improved, but the safety trial obviously does not allow to distinguish between the effects of cell injection and bypass grafting. In the subsequent efficacy trial in 40 patients randomly assigned to undergo CABG & cell injection or CABG controls we found that global left ventricular systolic function at 6 months was moderately but significantly better in cell-treated patients. It therefore appears that concomitant injection of $CD133^+$ bone marrow cells yields a functional benefit in addition to the bypass operation. Similar results were reported by Patel et al. in a randomized study of 20 patients with intramyocardial injection of $CD34^+$ autologous bone marrow stem cells and OP-CABG surgery (Patel et al, 2005). They found a significant improvement of ejection fraction (16.7 vs 6.5%) in the cell treated group versus a control group after 6 months. Catheter based intramyocardial injection of mononuclear bone marrow cell preparations by Perin et al (2003), Fuchs et al. (2003 and 2004) and Tse et al (2003) have also reported efficient improvement of left ventricular function in chronic ischemic heart disease.

Given the notion that autologous bone marrow stem cells can indeed improve the function of chronically ischemic myocardium in addition to the beneficial effects of traditional revascularization procedures, we believe that there is room for substantial further improvement. The cell number we have used is rather small and can be increased

by modifying the isolation process. Other cell types with a greater likelihood for true cardiomyocyte-differentiation, i.e. mesenchymal stem cell-derived cells, might ultimately prove more efficient. Strategies to precondition cells prior to implantation by pharmacologic, genetic, or physical means are also currently under evaluation. However, for the time being clinicians have to resort to clinically available cell products, and we believe that the approach we have chosen is invaluable in this respect.

1.1.2.6 Summary

Taken together, a significant amount of evidence based on a variety of small and large animal experiments indicates that neoangiogenesis in post-ischemic myocardium can be effectively induced by adult bone-marrow stem cells, while bone marrow stromal cells (i.e. mesenchymal stem cells) have primarily the potential to differentiate into cardiomyocytes. Several phase-I studies using crude bone marrow preparations are currently conducted, and initial results regarding safety and feasibility have been reported (Perin et al, 2003; Tse et al. 2003 and 2005; Beeres et al, 2006; Fuchs et al, 2003; Oakley et al, 2005). Safety and functional efficacy of highly purified human $CD133^+$ bone marrow stem cells have been shown in our own preclinical studies and clinical phase I/phase II trials in more than 60 patients undergoing CABG surgery.

1.2 Rationale

Stem cell therapy for myocardial regeneration after myocardial infarction is an exciting new field of medical research that has the potential to revolutionize cardiovascular medicine. Despite significant improvements in emergency treatment, myocardial infarction leads to a net loss of contractile tissue in many patients with coronary artery disease. Often, this is the beginning of a downward spiral towards congestive heart failure and life-threatening arrhythmia. Other than heart transplantation with its obvious limitations, current therapeutic means aim at preventing further episodes of myocardial ischemia and at enabling the organism to survive with a heart that is working only at a fraction of its original capacity. Those are far from representing a cure. In this situation, it is understandable that cardiac stem cell therapy attracts considerable attention and raises many hopes. In order to adequately judge both the potential benefits and the limitations of cardiac cell therapy, more understanding of the mechanism and the consequences of myocardial infarction and its current treatment concepts is needed.

The myocardium consists of terminally differentiated cells without a clinically relevant potential for regeneration. Hence, cardiomyocytes that were subject to necrotic or apoptotic cell death in acute myocardial infarction, ischemic cardiomyopathy, or myocarditis cannot be replaced by new contractile cells. Instead, remodeling processes ultimately lead to interstitial myocardial fibrosis or formation of a transmural fibrotic scar that further impairs systolic and diastolic ventricular function and may progress into formation of a LV aneurysm. Surgical or interventional revascularization of ischemic myocardium effectively treats angina, prevents myocardial infarction, improves function of still viable myocardium, and pharmacological therapy appears to have a beneficial impact on remodeling processes. However, viability and function of necrotic myocardium cannot be restored with current therapeutic options. Recently, transplantation of cells into infarcted myocardium has evolved a means to ultimately achieve this goal (Leri et al 2005, Steinhoff 2006). As most of ischemic heart diseases

result in chronic heart failure (Gheorghiade and Bonow, 1998) there is a high medical need for new regenerative therapies.

Based on the existing experience, it seems to be justified to conclude that transplantation of purified $CD133^+$ autologous bone marrow cells in the infarct border zone can be safely performed in patients with ischemic heart disease. Neoangiogenesis, neomyogenesis, or both have not been found in humans at this point. With this carefully designed randomized controlled study we will determine the efficacy of clinical cell transplantation for ischemic heart disease. It will be shown whether relevant myocardial regeneration can be induced by using primary isolated $CD133^+$ adult stem/progenitor cells that have not been expanded and modified ex vivo.

On the basis of the demonstration of clinical safety and efficacy in phase I and phase II studies, this study will show the therapeutic effect of $CD133^+$ isolated bone marrow stem cell injection into the myocardium of post infarct patients undergoing coronary artery bypass graft (CABG) operation. Safety and efficacy of the treatment will be clarified in a randomized placebo controlled multicenter study.

So the aim of this clinical trial is to investigate whether injection of autologously derived bone marrow stem cells yields a functional benefit in addition to the CABG operation. The following hypothesis will be tested: “Patients who undergo CABG & stem cell injection have no different left ventricular ejection fraction than patients, who undergo CABG alone, measured by Cardio MRI prior and 6 months after the operation.” This will be the primary endpoint. The changes in physical exercise capacity and myocardial perfusion will be assessed by 6 minute walk test, echocardiography, New York Heart Association (NYHA) and Canadian Cardiovascular Society (CCS) status. Patients will also be asked to complete Quality of Life (Minnesota and SF 36) questionnaires. Safety will be assessed by adverse events, the combined clinical endpoints of major adverse cardiac events (MACE: death, recurrent AMI, hospitalization with heart failure or ventricular tachycardia, target vessel revascularization including adverse events and ventricular arrhythmia), laboratory assessments and unwanted tissue changes.

1.3 Risk-Benefit Assessment

Patients with severe ischemic heart disease have a decreased Quality of Life and prognosis for survival. The conventional treatments including coronary bypass graft operation, intracoronary vascular stents and pharmacological support are not able to cure the ischemic disease. A major limitation is the recruitment of hibernating myocardium by angiogenesis and preventing of sequential loss of heart tissue. This restriction in medical treatment can be overcome by the regeneration of this diseased tissue by improved revascularization and oxygenation. Bone marrow stem cells are capable to induce angiogenesis in ischemic tissue and therefore can be used to treat this affection. The full revascularization of postinfarction myocardium can lead to a better rescue of heart tissue, prevention of progression of disease and improved left ventricular heart function. The effect of bone marrow stem cells is limited to angiogenesis of small vessels in the myocardium. Therefore, the combination of macrovascularization by CABG-surgery with microvascularization by bone-marrow stem cells is expected to give a maximal benefit to rescue ischemic heart tissue of the patient. The potential risks of the treatment (listed below) can be minimized by a number of proven measures for the selection, preparation, and application of bone-marrow stem cells of the patient.

Potential *risks* of cardiac stem cell therapy

- Transmission of infection
- Tumor formation
- Unwanted tissue formation
- Inflammation
- Immunogenicity
- Ventricular arrhythmia
- Deterioration of heart function

Potential *benefits* of autologous CD133$^+$ selected intramyocardial bone marrow stem cell therapy

- Selective isolation of early bone marrow stem cells expressing CD133 (vascular precursor cells capable to induce angiogenesis)
- Signs of efficient non-hematological differentiation of CD133 positive bone marrow stem cells into vascular cells (as compared to CD34$^+$ bone marrow stem cell selection)
- Quality assurance of cell preparation and viability
- Autologous cell preparation (no risk of immunogenicity)
- GLP/GMP-preparation (reduced risk for transmission of infection)
- Primary cell isolation, minimal cell manipulation (CD133 isolation) without in vitro modification (clearly defined unmodified stem cell population used in stem cell transplantation for hematological disease with proven safety for several years)
- Stem cell purification with discardence of inflammatory mononuclear bone marrow cells (reduced risk of tissue inflammation as compared to unselected mononuclear bone marrow cell preparations)
- No signs of tumor and unwanted tissue formation in all preclinical and clinical (phase-I and phase-II) studies
- No electrical cellular activity; no signs of ventricular arrhythmia in preclinical and clinical (reduced risk of ventricular arrhythmia) studies
- Precise local epicardial administration by intramyocardial injection in the infarct border zone (advantage as compared to intravascular intracoronary application without control of stem cell homing to the myocardium; advantage as compared to endomycardial catheter injection with less control on morphological application and bleeding complications)

Previous studies of intramyocardial bone-marrow stem cell transplantation:

In previous studies the safety, feasibility and, in part, efficacy of intramyocardial bone marrow stem cell transplantation was demonstrated in humans (Leri et al, 2005). In all trials a significant improvement of LV function and/or improved myocardial perfusion was shown. There were no procedure-related complications, especially no new ventricular arrhythmia or neoplasia. In addition, the intracoronary administration of bone-marrow stem cells after acute myocardial infarction has shown no serious side effects (Assmuss et al, 2002; Fernandez-Aviles et al, 2004; Strauer et al, 2002; Wollert et al, 2004 and 2005). Intracoronary applications, however, are not efficient in chronic ischemic heart disease and have limited functional benefit in acute myocardial infarction (Schächinger et al, 2006; Lunde et al, 2006; Assmus et al, 2006).

In previous phase I (15 patients; 2001-2003) and phase II (20+20 patients; 2003-2005) studies involving intramyocardial injection of $CD133^+$ selected bone marrow stem cells there were no adverse events (AEs) in relation to stem cell application. Long-term mortality analysis shows that until 2006 two patients died late after operation. One patient died 7 months after CABG-operation (phase I), one patient 14 months after operation (phase II, study control group) (Stamm et al, 2007). Based on this knowledge a favorable risk-benefit rate has been expected.
Data of a randomized, placebo-controlled, double-blinded phase II single center trial, recently published at the American College of Cardiology meeting in March 2012, Chicago, (Nasseri et al. 2012) failed the primary endpoint, showing no additional benefit of $CD133^+$-stem-cell-treatment in patients undergoing Bypass-grafting (only a subgroup with posterior infarcts showed improvement). Although trial design and patient population of the Cardio 133 and the PERFECT trial differ, the degree of uncertainty about the expected effect size has increased due to this new data. Therefore it has been decided to append an interim analysis to the PERFECT protocol to re-estimate the sample-size or stop the trial for futility if necessary.

CABG surgery:

In all patients of the study population, the coronary artery bypass operation is absolutely necessary in order to treat angina and to prevent the occurrence of new myocardial infarction events with possibly fatal consequences. Thus, the indication for CABG surgery is not linked with, and will not be altered by, the cell therapy protocol that is the subject of this study. CABG surgery has its innate risks, which are believed to be outweighed by the expected benefit. According to recent data published by cardiac surgery quality control bodies, the average risk of in-hospital death for patients undergoing elective CABG surgery is in the range of 2-3% (Vaccarino et al, 2002). Depending on preoperative co-morbidity, age, and LV contractile function the expected surgical risk increases (Parker et al, 2006). However, in patients who have been well examined and prepared, and who undergo scheduled surgery, the risk of death is not expected to exceed 5%. Actual data published in the SYNTAX-trial show a MACCE-rate (Major Adverse Cardiac or Cerebrovascular Event) in CABG-surgery of 9.9% in a 6 month period after surgery (Serruys et al, 2009). Due to the reduced ejection fraction of the patients included into this study the patients will be more impaired and the MACE-rate might be higher compared to the SYNTAX cohort.
In this study, patients will be randomized to receive either isolated $CD133^+$ mononuclear cells from autologous bone marrow (cells suspended in 5 mL physiological saline with 10% autologous serum) or placebo (physiological saline with 10% autologous serum) intramyocardially during the CABG operation. See Section 3.3 for justification of the design of this study.

The available information suggests that the present study has a favorable risk-benefit ratio.

2 STUDY OBJECTIVES

2.1 Primary Objective

To determine whether injection of autologously-derived bone marrow stem cells yields a functional benefit in addition to the coronary artery bypass graft (CABG) operation as determined by left ventricular heart function (LVEF-MRI).

2.2 Secondary Objectives

To determine the effects of an injection of autologously derived bone marrow stem cells on physical exercise capacity, cardiac function, safety and Quality of Life (QoL).

3 OVERALL DESIGN AND PLAN OF THE STUDY

3.1 Overview

This will be a placebo controlled, prospective, randomized, double-blind multicenter, phase III, clinical trial investigating the effects of intramyocardial injection of 5 mL $CD133^+$ bone marrow cells or placebo in 142 patients with coronary artery disease scheduled for CABG surgery. Patients will be randomized to one of the two treatment groups ($CD133^+$ or placebo) in a 1:1 ratio.

Patients will be required to attend 7 study assessments:

1. Assessment I prior to the operation (screening)
2. Assessment Ia between day -2 and day of operation (day 0; cell preparation and transfer)
3. Assessment II at day of operation (injection of study treatment)
4. Assessment IIa at day after operation (post OP/ICU)
5. Assessment III during the postoperative stay before or after discharge (within 72 hours of discharge)
6. Assessment IV (by telephone) at 3 months after the operation
7. Assessment V at 6-months after the operation (data closure)

At Assessment I the patient will be asked to sign the informed consent and baseline criteria will be assessed.

At Assessments I, III and V patients will undergo a 6-minute walk test (physical examinations and vital signs), Holter, 12-lead ECG, cardiac magnetic resonance imaging (MRI) and echocardiography, checking of unwanted tissue changes, laboratory including NT-proBNP and New York Heart Association (NYHA) and Canadian Cardiovascular Society (CCS) evaluations as well as listing of concomitant medications.

Adverse events (AE) will be assessed continuously (informed consent to study end). Furthermore, major adverse cardiovascular events (MACE) will be assessed at Assessment V. At Assessment IIa patients will undergo laboratory and 12-lead ECG evaluations at ICU.

At Assessments I, IV and V patients will be asked to complete QoL questionnaires.

Assessment Ia is related to cell-preparation and transfer and Assessment II is related to injections of study treatment.

The planned patient recruitment is 5 years, the planned duration of study, including follow-up (6 months) is 5.5 years. The duration of the study for each patient will be approximately 6 months.

An obligatory safety follow up is planned for further 18 months after study data closure. Therefore the patients will be required to attend 24 months after the operation Assessment VI. This will be outside the study after data closure. At Assessment VI patient will undergo examination of all safety parameters and will be asked to complete QoL questionnaires. AE documentation and reporting for the safety follow-up will include all (possibly) related AEs and all AEs of specific interest including MACE and death.

The trial will be conducted at seven sites in Germany. Patients will be informed by the study physician and guided additionally through the whole study by a “Study Pass”.

A Safety Monitoring Board (SMB) will be responsible for the overall safety of the patients in the trial. See Section 9.9 for further details.

Discontinuation criteria for patients, for parts of the study, and the entire study are described in Section 4.3.
See Appendix 1 for the trial flow chart and Table 2 for the schedule of trial assessments.

In all cases retention of samples of bone marrow and stem cells are stored for safety reasons in the laboratory up to the end of the study. After the end of the study (approx. 2.5 years) the patient may decide what should happen to the stored material: The patient may donate the samples for a research study (pseudonymous) or decide for a further storage or for the destruction of the material. Therefore, all patients will be unblinded at the end of the study and will decide on the use of the material.
During the preparation procedure a small fraction of the cells will be used for research-related investigations (project: “Weitere funktionelle Analysen von adulten Knochenmark-Stammzellen in vitro und in vivo”). The patient agrees to the procedure by a signature on the informed consent form.

3.2 Endpoints

Primary endpoint

The primary endpoint is LVEF at 6 months postoperatively, measured by MRI at rest. Cardiac MRI is established as the gold standard for determination of LV function (LVEF and LV volumes).

Secondary endpoints

1. Change in LVEF at 6 months post-OP compared with preoperatively (screening) and early postoperatively (discharge) as assessed by MRI and echocardiography.
2. Change in LV dimensions (left ventricular end systolic dimension [LVESD], left ventricular end diastolic dimension [LVEDD]) at 6 month post-OP compared with preoperatively (screening) and early postoperatively (discharge) as assessed by echocardiography.
3. Change in physical exercise capacity determined by 6 minute walk test at 6 months post-OP compared with preoperatively (screening) and early postoperatively (discharge).
4. Change in NYHA and CCS class at 6 months post-OP compared with preoperatively (screening) and early postoperatively (discharge).
5. MACE (cardiac death, myocardial infarction, secondary intervention/reoperation, ventricular arrhythmia).
6. QoL-score at 6 months post-OP compared with preoperatively (screening) and 3 months post-OP (telephone).

3.3 Justification of the Study Design

The study design results from our experience with phase I and phase II studies in Rostock. $CD133^+$ positive bone-marrow stem cells were isolated from the patients own bone marrow (iliac crest puncture) and prepared according to GMP standards. Small volume preparations of 5 mL were found safe for intramyocardial application using 0.2–0.3 mL volume per injection. The distribution of injection-sites was found best using one injection per square cm and 26 gauge injection needles. The injections were placed in the

borderline tissue around patients' heart infarction, so that 10-15 injections are needed to circle around the infarction area.

The main limitation of the previous controlled study was the absence of a placebo-controlled randomized grouping to exclude influence of intramyocardial injection (saline) on myocardial function. No side-effects were found in the safety and efficacy trial. No rescue medication was necessary.

We have chosen the analysis of LVEF 6 months after CABG surgery by MRI as a primary endpoint to measure the effect on regeneration of the functionaly impaired left ventricle after myocardial infarction. MRI is considered as gold standard for measurement of left ventricular function parameters. To exclude bias analysis will be done by a central MRI core-lab.

Additional secondary endpoints will underline the effects of the injection of autologously derived bone marrow stem cells with determination of physical exercise capacity, echocardiography, safety and QoL.

Former studies and experiments have shown that on average more than $5x10^6$ $CD133^+$ bone marrow stem cells can be isolated from 200 mL bone marrow aspirate (Ghodsizad, 2004). In this trial $0.5\text{-}5x10^6$ $CD133^+$ cells isolated from the harvested bone marrow will be administered. Cell yields lower than the expected $0.5x10^6$ but $\geq 0.1x10^6$ will be documented by the cell preparation team (Team A) and the information forwarded to the Statistician but not to the physician and the patient. These patients will be regarded as drop-outs but will be followed-up similar to the intend-to-treat population and evaluated separately.

Cell yields lower than $0.1x10^6$ will be documented and the product will not be released by the Team A. The patient will be dropped out of the study and the treating physician and Statistician will be informed. These patients will not be followed-up. In case the over-all drop-out rate exceeds the assumed 15% those patients that dropped-out post-hoc because of insufficient cell counts may be replaced.

4 STUDY POPULATION

The study population will consist of patients with coronary artery disease after myocardial infarction with an indication for CABG surgery. Patients must be able to provide written consent and meet all the inclusion criteria and none of the exclusion criteria.

4.1 Inclusion Criteria

Patients will be entered into this study only if they meet all of the following criteria:

1. Coronary artery disease after myocardial infarction with indication for CABG surgery
2. Currently reduced global LVEF assessed at site by cardiac MRI at rest (25% ≤ LVEF ≤ 50%)
3. Presence of a localized akinetic/hypokinetic/hypoperfused area of LV myocardium for defining the target area
4. Informed consent of the patient
5. 18 years ≤ Age < 80 years
6. Are not pregnant and do not plan to become pregnant during the study. Females with childbearing potential must provide a negative pregnancy test within 1-7 days before OP and must be using oral or injectable contraception (non-childbearing potential is defined as post-menopausal for at least 1 year or surgical sterilization or hysterectomy at least 3 months before study start).

4.2 Exclusion Criteria

Patients will be entered into this study only if they meet none of the following criteria:

1. Emergency operation
2. Presence of any moderate-severe valvular heart disease requiring concomitant valve replacement or reconstruction
3. Medical History of recent resuscitation in combination with ventricular arrhythmia classified by LOWN ≥ class II
4. Acute myocardial infarction within last 2 weeks
5. Debilitating other disease: Degenerative neurologic disorders, psychiatric disease, terminal renal failure requiring dialysis, previous organ transplantation, active malignant neoplasia, or any other serious medical condition that, in the opinion of the Investigator is likely to alter the patient’s course of recovery or the evaluation of the study medication’s safety
6. Impaired ability to comprehend the study information
7. Absence of written informed consent
8. Treatment with any investigational drug within the previous 30 days
9. Apparent infection (c-reactive protein [CRP] ≥ 20 mg/L, fever ≥ 38.5° C)
10. Contraindication for MRI scan
11. Immune compromise including Anti HIV 1/2, HBsAg, Anti-HBc-IgG, Anti HCV, Treponema pallidum
12. Pregnant or breast feeding
13. Childbearing potential with unreliable birth control methods
14. Have previously been enrolled in this study, respectively phase I and phase II

15. Known hypersensitivity or sensitization against murine products and human-anti-mouse-antibody-titer ≥ 1:1000
16. Contraindication to bone marrow aspiration
17. Known hypersensitivity against iron dextrane

4.3 Patient Withdrawal and Replacement

Patients may withdraw from the study at any time without penalty and for any reason without prejudice to his or her future medical care. All patients will be closely monitored by their individual cardiologist and general practitioner and treated as appropriate, according to the guidelines issued by the national and international cardiology organizations that relate to patients with coronary artery disease and patients with congestive heart failure. The Investigators reserve the right to conduct additional follow-up examinations.

Patients must be withdrawn under the following circumstances:

- The patient withdraws consent;
- Pregnancy. If patients are to withdraw due to pregnancy, they should be followed-up until the outcome for the mother and fetus are known.
- If serious clinical events (e.g., emergency surgery, myocardial infarction, stroke, fever ≥ 38.5^{o}C) occur between recruitment and planned surgery.

Patients may be required to withdraw after discussion with the Sponsor and/or Investigator for the following reasons:

- Adverse event(s);
- At the discretion of the Investigator;
- Violation of eligibility criteria;
- Deviation from the treatment plan specified in the protocol (e.g., incorrect administration of the study drug, failure to attend study visits).

In all cases, the reason(s) for withdrawal, and the primary reason, will be recorded on the case report form (CRF). If a patient is prematurely withdrawn from the study for any reason, the Investigator will make every effort to perform the evaluations described for the Early Termination Visit (see Section 7.2.6.2).

An excessive withdrawal of patients can render the study uninterpretable. Therefore unnecessary withdrawal of patients should be avoided. In case of withdrawal, all efforts should be made to complete and report the observation and justification of withdrawal in as much detail as possible. Withdrawn patients that dropped-out of the study because of SAE will not be replaced. However, patients who are not able to reach the primary endpoint because of their body size/weight may be replaced.

Patients from both groups, who either received Placebo or CD133+ cells may be excluded post-hoc from the study because of insufficient CD133+ cell counts (<0.5 Mio but ≥0.1 Mio). In case the drop-out rate exceeds the assumed rate of 15% these patients may be replaced. Furthermore, patients who were randomized but did terminate the study before treatment may be replaced.

4.4 Planned Sample Size and Number of Study Centers

It is planned to recruit 142 patients at seven centers in Germany for this study. Each center should enroll between 20 and 80 patients. See Section 8.7 for a discussion of sample size.

4.5 Patient Identification and Randomization

Upon enrollment, each patient will receive a four-digit **patient number** consisting of (*x-yza*). The 1st digit assigned *x*, indicates the study site (–see List of trial personnel), the 2nd, 3rd and 4th digit assigned, *yza*, indicates the individual patient. Enrolled patients who drop out of the study before randomization will retain their patient number.

Randomization to study treatment will occur at Assessment Ia (cell-preparation) after all screening procedures have been performed, eligibility for the study confirmed and after bone-marrow aspiration. The randomization will be attributed by Team A (cell processing team). Each randomized patient will receive a four-digit **randomization number** (*x-yza*). The 1st digit assigned, *x*, indicates the study site (–see Lsit of trial peronnel), the 2nd and 3rd and 4th digit assigned, *yza*, indicates the individual patient.

Randomized patients who terminate their study participation for any reason, regardless of whether study intervention was taken or not, will retain their randomization number. The randomization code will be assigned using sealed envelopes provided to the Team A for each study center.

Patients will be randomized on a 1:1 basis to receive $CD133^+$ cells or placebo.

The randomization procedure will be stratified by study site and a sequence of randomization numbers will be assigned to the bone-marrow isolation laboratory (Team A) preparing for the study centers. As randomization procedure the Permuted Block Design within strata will be used (Rosenberger and Lachin, 2002). The block size within each stratum will be randomly selected. The size of the randomization blocks as well as seed numbers will be documented but will not be disclosed to the study centers to avoid predictability of treatment. The study treatment will be randomly assigned to the randomization numbers in advance according to the randomization list held by the Biostatistician.

The randomization envelopes are numbered in an ascending order. If a patient is going to be randomized a member of Team A opens the envelope with the lowest number among all unused sealed envelopes available for this center. According to the information randomization will be carried out and $CD133^+$ cell or placebo product will be produced for the current patient.

The randomization code will be stored by the Statistician. Only the members of Team A will have access to the randomization code. All members will sign an agreement, stating that the randomization code will be kept confidential and no person outside the trial Team A will have access to the code.

At each site sealed emergency envelopes will be stored at a secure place containing the randomization code. In case of an emergency which requires knowledge of treatment the treatment code for an individual patient can be revealed to a member of Team B (treatment team) by opening the sealed envelope. See Section 5.4 for details of breaking the blind.

5 STUDY TREATMENT

5.1 Identity

All patients enrolled in the study will undergo bone marrow aspiration (150-200 mL) and withdrawal of 20 mL blood one to two days before CABG surgery at Assessment Ia.

As there is only autologous application of the stem cells each patient is his own donor. Herewith the inclusion and exclusion criteria of the study population and the assessments describe the selection and testing of the donors.

For all patients $CD133^+$ cells will be selected from the bone marrow aspirate. For patients randomized to the active treatment group the cells will be suspended in physiological saline + 10% autologous serum. For the patients of the control group, the $CD133^+$ cells will be stored at the manufacturer. See Section 5.8 for further details of bone marrow aspiration and Section 5.9 for further details of $CD133^+$ cell and placebo preparation and treatment.

5.2 Administration

Patients randomized to the active treatment group will be given 5 mL $CD133^+$ cells, saline and serum suspension intramyocardially during CABG surgery (see Section 5.10 for further details on CABG surgery).
Patients randomized to placebo treatment will be given 5 mL saline plus serum solution intramyocardially during CABG surgery
For both treatment groups, the treatments will be administered intramyocardially in the infarction border zone (penumbra) during the cardiac surgical procedure. The procedure will be performed with extracorporal circulatory support, aortic cross clamping and cardiac arrest induced by cardioplegia according to the center standards. The treatments will be administered before cross clamp release.
A series of fifteen individual injections (1 mL syringes, 26 Gauge needle) of 0.3 mL aliquots (in total 5 mL, including up to 0.5 mL rest in syringes) will be applied within 3 minutes in the region of interest (infarction border zone) at the end of bypass surgery (before the release of aortic cross clamp). No more than one injection per square centimeter should be injected. For documentation of injection sites see Figure 5.

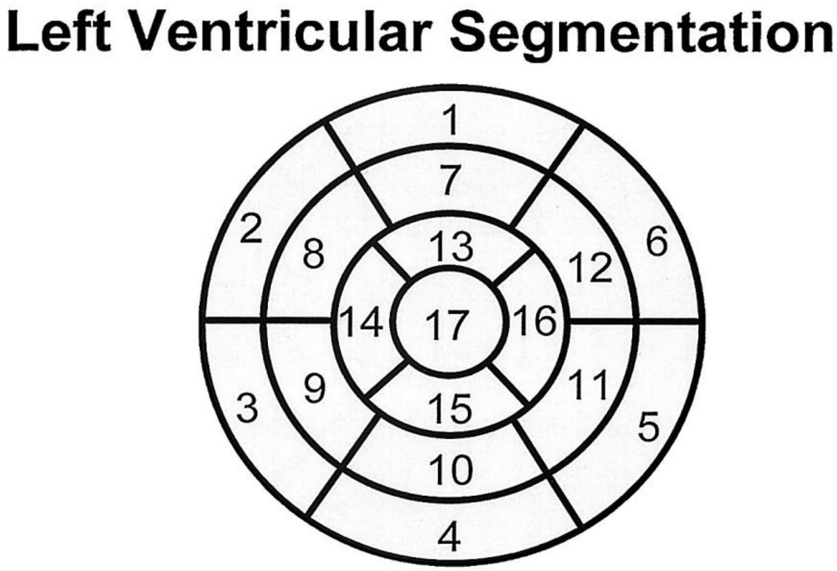

1. basal anterior	7. mid anterior	13. apical anterior
2. basal anteroseptal	8. mid anteroseptal	14. apical septal
3. basal inferoseptal	9. mid inferoseptal	15. apical inferior
4. basal inferior	10. mid inferior	16. apical lateral
5. basal inferolateral	11. mid inferolateral	17. apex
6. basal anterolateral	12. mid anterolateral	

Figure 5: Left ventricular segmentation

Patients will be treated after the standards of the centers, in accordance with the American Heart Association (AHA) guidelines for current standard of care.

5.3 Packaging, Labeling and Storage

The cell suspension or placebo product will be delivered in 1 mL opaque syringes which are packed for transport in an outer package. The syringes will be labeled in accordance with the applicable regulatory guidelines GCP-O §5 (2) 5 for labeling requirements for containers with no more than ten milliliters volume. The outer package will be labeled with the complete requirements in accordance with GCP-O §5 (2) (with name, address and telephone number of the Sponsor, name and strength of the product, with a note, that the medicinal product is intended for use in clinical trial, with precautions for the disposal of unused IMP, with trial center identification and patient identification number, and other details. As it is an autologous product and only very limited storage is allowed, additional notes will be on the outer package as: Do not irradiate! Pass on without delay! For immediate use!

See Section 5.9 for the preparation of the investigational product and placebo.

5.4 Blinding and Breaking the Blind

The study will be performed in a double-blind manner. The appearance of the final placebo and cellular product will be indistinguishable to the Investigators. Cell

concentrations of 0.2 to 2.0 x 10^6 are not detectable by pure vision in the type of syringes used for application.The patients will be blinded to the treatment they receive. The surgeons and the Investigators involved in the preoperative and postoperative assessments will be blinded regarding the treatment group assignment (Team B). Only the laboratory personnel involved in the cell isolation process will not be blinded (Team A).

Team A (cell processing team) will be responsible for the preparation of the medicinal product as well as for the preparation of the placebo control and for randomization. This team will not be involved in patient recruitment/selection, clinical assessment, data collection or the treatment/sham injection. These unblinded team members should not reveal the identity of the study medication at any time.

Team B (treatment team) will be responsible for the patient recruitment/selection, clinical assessment, data collection, bone marrow harvest, and will perform the treatment/sham injection. The members of Team B will be unaware of the randomization code and blinded to the treatment.

In case of an emergency, and necessity for breaking the code, an emergency envelope will be available 24 hours a day, 7 days a week for a member of Team B. When breaking the code, a member of Team B will sign and date on the emergency envelope which has been unblinded. The treatment code for an individual patient can be revealed to a member of Team B only in case of an emergency, which requires knowledge of treatment. At the end of the trial all emergency envelopes will be returned to the Statistician and will be checked for integrity of the seal.

The study blind should not be broken except in a medical emergency (where knowledge of the study drug received would affect the treatment of the emergency) or regulatory requirement (e.g., for serious adverse events [SAEs] or death).

The investigator should promptly document and explain to the sponsor any code breaking (e.g. accidental unblinding, unblinding due to a serious adverse event) of the investigational product.If the blind is broken, the date, time and reason must be documented in the patient’s CRF, and any associated AE report.

If an emergency unblinding becomes necessary, the Investigator should notify the Sponsor/Medical Monitor, if possible, prior to unblinding. The Investigator is responsible for opening the specified envelope, in the presence of a witness, both of whom must sign and date the envelope.

All envelopes, whether sealed or opened, must be returned to the Statistician, overviewed by the Sponsor at the end of the study.

The SMB will have access to the randomization code, and the code may be broken after appropriate discussion with the Sponsor.

If an Investigator, site personnel performing assessments, or patient, is unblinded, the patient must be withdrawn from the study and procedures accompanying withdrawal are to be performed. In cases where there are ethical reasons for the patient to remain in the study, the Investigator must obtain specific approval from LKP/Sponsor for the patient to continue in the study.

Serious unexpected suspected adverse reactions (SUSARs), which are subject to expedited reporting, should be unblinded before submission to the Regulatory Authorities.

The overall randomization code will be broken only for reporting purposes. This will occur once all final clinical data have been entered onto the database and all data queries have been resolved, and the assignment of patients to the analysis populations has been completed.

5.5 Drug Accountability

The Investigator is responsible for maintaining accurate study drug accountability records throughout the study. The dispensing of the study treatment will be documented in the CRF “intra OP” on day 0.

The Investigator is responsible for ensuring that all unused or partially used study treatment will be disposed of according to TFG § 17 and the local regulations for biological products.

Study treatment which has not abandoned the manufacturer and that is determined not to be used for patient treatment anymore or supernumerous stem cells after manufacturing will be used for research or will be destroyed according to the patient information and the informed consent. The manufacturer will ensure, that disposal will follow TFG § 17 and his regulations for biological products.

5.6 Compliance

The study treatment will be administered by the Investigator therefore patient compliance with study treatment will not be monitored.
Patients that are non-compliant with the study protocol such as non-attendance at study visits or refusal to undergo certain assessments may be candidates for patient withdrawal (see Section 4.3).

5.7 Concomitant Medications

Any medication for the patients’ treatment according to the guidelines and the standards of the centers is permitted.
Any medication the patient takes other than the study drug, including herbal and other non-traditional remedies, is considered a concomitant medication. All concomitant medications are recorded in the CRF "Concomitant Medication": generic name, indication, route of administration, dose rate with unit of measurement, date started (before trial or date), date stopped (ongoing or date) and application (continuous or as necessary. Any change in the dosage or regimen of a concomitant medication is recorded in the CRF.
At Screening, patients will be asked what medications they are currently taking. Additionally any platelet aggregation inhibitors they have taken during the last 2 weeks will be documented. At each subsequent assessment, any new concomitant medication and any changes in concomitant medications will be documented.

The following medications will be of special interest:

ACE-inhibitor	ASS
Aldosteron-Antagonist	ATII Receptor Antagonist
Beta-blocker	CSE-inhibitor
Ca-Antagonist	Diuretic
Digitalis	Marcumar
Antiarrhythmic other	Nitrate

During CABG-surgery standard medication (narcotics etc.) will not be documented in the CRF. Only medication for support of cardiovascular system during OP will be documented.

Only medication for treatment of Adverse or Serious Adverse Events will be documented.

During stay in the Intesive Care Unit only maximum dosages of catecholamines and inotropics are documented and medication for treatment of Adverse or Serious Adverse Events.

5.8 Bone Marrow Aspiration, Blood Collection and Transport

All patients enrolled in the trial will undergo bone marrow aspiration from the iliac crest. The procedure will be carried out under local anesthesia one to two days before surgery (Day -2 to -1). In total 150 to 200 mL bone marrow will be harvested. The procedure will be performed by an experienced hematologist or surgeon within a qualified operating room according to §20b German Medicinal Products Act. Furthermore, 20 mL of peripheral blood will be drawn for preparation of autologous serum. The bone marrow aspiration, blood collection, packaging, labeling and transport to the cell processing laboratory will follow SOP's developed and validated by Miltenyi Biotec GmbH.

5.9 CD133^{+} Cell and Placebo Preparation and Treatment

The manufacturing process of CD133^{+} Cell Product and placebo will be performed in a central GMP laboratory assigned by Miltenyi Biotec. The manufacturing process and quality control is performed according to validated procedures and documented in accordance with full GMP requirements.

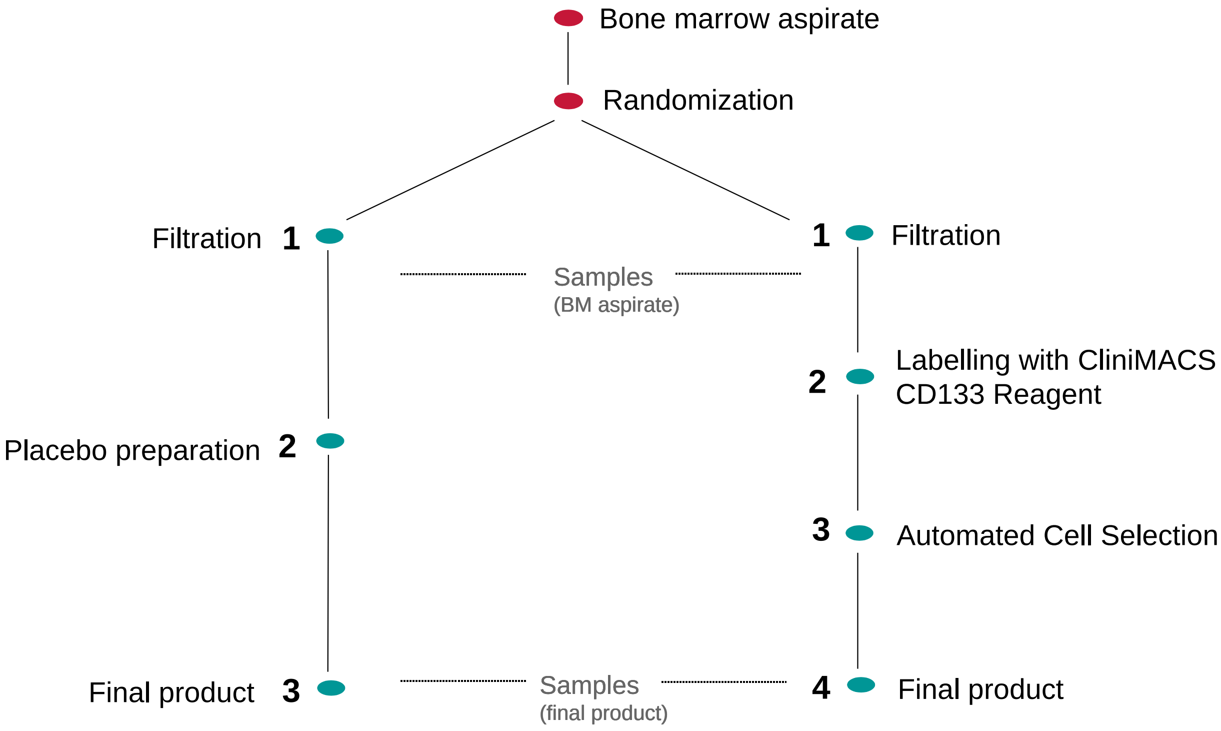

Figure 6: Scheme of manufacturing process
Patients will be randomized at the cell processing department in a 1:1 ratio to receive either cell product or placebo injection.

5.9.1 *CD133⁺ Cell Product Release criteria*

To ensure consistent quality and individual safety of the cell product, it will only be released if the following specifications regarding the validated manufacturing process have been met:

A. Minimum number of $CD133^+$ cells = $0.1x10^6$ cells
B. Maximum number of $CD133^+$ cells = $5x10^6$ cells
C. Minimum depletion of non-target cells = 2.5 log (> 99.6%)
D. Minimum percentage of viable cells = 80%
E. Manufacturing and Quality Control according to GMP

5.9.2 *Final formulation of $CD133^+$ Cell Product and Placebo*

The $CD133^+$ cells after the CliniMACS selection process will be resuspended in 5 mL of saline supplemented with 10% autologous serum and will be drawn into 5x1 mL syringes. Placebo preparations will consist of 5 mL of saline supplemented with 10% autologous serum only and will be also drawn in 5x1 mL syringes. Each 5 syringes will be packed in an outer package by the manufacturer and has to be administered within a maximum of 72 hours after aspiration.
The chosen specification for the final formulation of the IMP has been set primarily according to the results of the former phase I and II studies of the LKP (Stamm et al.

2007: range 1.08 x10^6 to 8.35 x 10^7) and further revised by additional comparative evaluation runs according to current GMP standards to the actual IMP formulation (0.1-5x10^6 $CD133^+$ cells).

5.9.3 *Practices with final formulation of $CD133^+$ Cell Product which do not fulfill the release requirements*

Final formulations which do not fulfill the release requirements will not be used in the study. The patient will be dropped out of the study and the treating physician and Statistician will be informed.

The Statistician will inform the Safety Monitoring Board, if the drop-out rate exceeds the calculated limit (see also 3.3).

In accordance with the patient's consent the cells which are not administered will either be used for research or will be destroyed.

5.10 Coronary Artery Bypass Graft (CABG)

The basic surgical technique has not fundamentally changed for several decades. The chest is opened via a median sternotomy, the pericardium is opened, and the heart is exposed. Following cannulation of the ascending aorta and the right atrium, extracorporal circulation is initiated. While the heart is still perfused and beating, the stenotic or occluded coronary arteries are exposed and prepared. Now, the aorta is clamped and a cardioplegic solution is delivered into the coronary system, arresting the heart and protecting the myocardium from ischemic injury. The coronary artery is incised and a bypass graft (i.e. saphenous vein, internal mammary artery, or others) is anastomosed to this incision. Once all bypass-to-coronary artery anastomoses have been constructed, the aortic cross clamp is released and the heart reperfused. Using a side-biting instrument, the anterior portion of the ascending aorta is partially clamped. Perforations are placed in the aortic wall, and the other end of the bypass graft is anastomosed to the aorta. Upon completion, blood flow through the bypass grafts is released and the patient is weaned from extracorporeal circulation. In a patient with impaired LV function, positive inotropic drugs may be necessary to support heart function for some time, and if this is not sufficient, intraaortic balloon counter pulsation can be used to support heart function until the myocardium has recovered.
All patients will receive the CABG operation as indicated. The decision to operate will be made in routine fashion by the referring cardiologist and the consulting surgeon. CABG surgery will be carried out on-pump and with cardioplegic arrest. All coronary arteries that should be, and can be, treated will be grafted. Upon completion of the distal anastomoses, the infarct area will be visualized and the cell suspension will be injected in the infarct border zone. From then on, the operation will be completed as per normal practice.

6 PARAMETERS AND METHODS OF ASSESSMENT

6.1 Efficacy Parameters

If not stated otherwise, all examinations have to be performed at the trial site. However, routine examinations that are executed at the trial site before informed consent was obtained should not be repeated provided the respective examination has been performed according to trial protocol. In order to avoid unnecessary inconvenience and risk for the patient, this data may also be used as historic data.

6.1.1 *Left Ventricular Ejection Fraction (LVEF)*

LVEF will be measured by cardiac MRI, established as the gold standard for determination of LV function (LVEF and LV volumes). According to the schedule cardiac MRI will be performed at Assessment I, Assessment III and Assessment V.

LVEF at 6 months postoperatively (Assessment V), measured by MRI at rest is the *primary endpoint*.

Secondary endpoints include change in LVEF pre-operatively (Assessment I) with 6 months post-operatively (Assessment V). This will be assessed by cardiac MRI scans and echocardiography (see Section 6.1.5).

Cardiac MRI scans will be performed in accordance with the Schedule of Assessments (see Table 2). The analysis and interpretation of data from the cardiac MRI scans concerning LV function parameters will be done centrally by a reviewer blinded to the treatment the patient has received.

LVEDD, LVEDV, LVESD and LVEF will be recorded in the CRF. The main area of impaired LV infarction will also be recorded in the CRF and quality will be recorded as normal, akinetic, hypokinetic or dyskinetic.

6.1.2 *Left Ventricular (LV) Dimensions*

LVESD and LVEDD will be assessed pre-operatively (Assessment I), early post-operatively (Assessment III), after 6 months (Assessment V) and after 24 months (Assessemnt VI) by echocardiography.

Echocardiography will be performed in accordance with the Schedule of Assessments (see Table 2). The review will be done centrally by a reviewer blinded to the treatment received by the patient.

6.1.3 *Classification of Heart Failure (NYHA)/Angina (CSS)*

Patients will be graded according to the NYHA classification as follows:

Class I:	Patients with no limitation of activities; they suffer no symptoms from ordinary activities.
Class II:	Patients with slight, mild limitation of activity; they are comfortable with rest or with mild exertion.
Class III:	Patients with marked limitation of activity; they are comfortable only at rest.
Class IV:	Patients who should be at complete rest, confined to bed or chair; any physical activity brings on discomfort and symptoms occur at rest

NYHA assessments will be performed in accordance with the Schedule of Assessments (see Table 2). Additionally the CCS classification will be used to classify angina.

Canadian Cardiovascular Society (CCS) classification system for Angina

CCS Class 0	Asymptomatic
CCS Class I	Ordinary physical activity such as walking, climbing stairs does not cause angina. Angina occurs with strenuous, rapid or prolonged exertion at work or recreation
CCS Class II	Slight limitation of ordinary activity. Angina occurs on walking or climbing stairs rapidly, walking uphill, walking or stair-climbing after meals, or in cold, or in wind, or under emotional stress, or only during the few hours after awakening. Walking more than two blocks on the level and climbing more than one flight of ordinary stairs at a normal pace and in normal conditions.
CCS Class III	Marked limitations of ordinary physical activity. Angina occurs on walking one to two blocks on the level and climbing one flight of stairs in normal conditions and at a normal pace.
CCS Class IV	Inability to carry on any physical activity without discomfort - anginal symptoms may be present at rest.

6.1.4 *Heart Catheterization*

Critical stenosed vessels (≥50%) will be recorded in the CRF as follows: LMCA, RIVA, RCX, RCA. The LVEF (%) and area of LV infarction (septal, posterior, anterior, lateral and other) will also be recorded in the CRF (Assessment I, Screening). Historic data (not older than 3 months) may be used.

Quality will be recorded as akinetic, hypokinetic, hypokinetic to akinetic or dyskinetic.

6.1.5 *Cardiac MRI Scan*

Cardiac MRI Scan will be performed in accordance with the Schedule of Assessments (Table 2, Assessments Screening, Hospital Discharge, 6-MonthFollow-Up). The analysis and interpretation of data from the cardio MRI concerning LV function parameters will

be performed centrally by a reviewer blinded to the treatment the patient has received. The value for LVEF for Assessment I/Screening will be determined within 12 hours by the MRI Core Lab personnel and the result will be forwarded immediately to the treating physician. The following will be recorded continuously in the CRF:

- Date of recording
- LV mass (g/m^2), body surface (m^2), weight (kg), height (cm)
- LVEDV, LVESV, LVEF (%), Scar tissue total (g), Non-viable tissue (g)
- Ventricle function and wall motion (quality) for segments 1-17 (see Figure 7; 1 = hyperkinetic, 0 = normokinetic, -1 = hypokinetic, -2 = akinetic, -3 = dyskinetic)
    - Wall motion thickening (%)
    - Regional muscle mass (g)
- Perfusion at stress and at rest for segments 1-17 (see Figure 7; 0 = normal, 1 = reduced subendokardial, 2 = reduced transmural)
- Vitality/late enhancement (LE) for segments 1-17 (see Figure 7):, LE volume (% LV mass), transmurality LE (%)
- Total assessment vitality for segments 1-17 (see Figure 7; 0 = normal, -1 = stunned, -2 = hibernating, -3 = infarct)
- Unwanted tissue changes; no/yes, if yes, describe
- Pericardial effusion (no, few, moderate, much)
- Pleural effusion (no, few, moderate, much)
- Thrombus no/yes

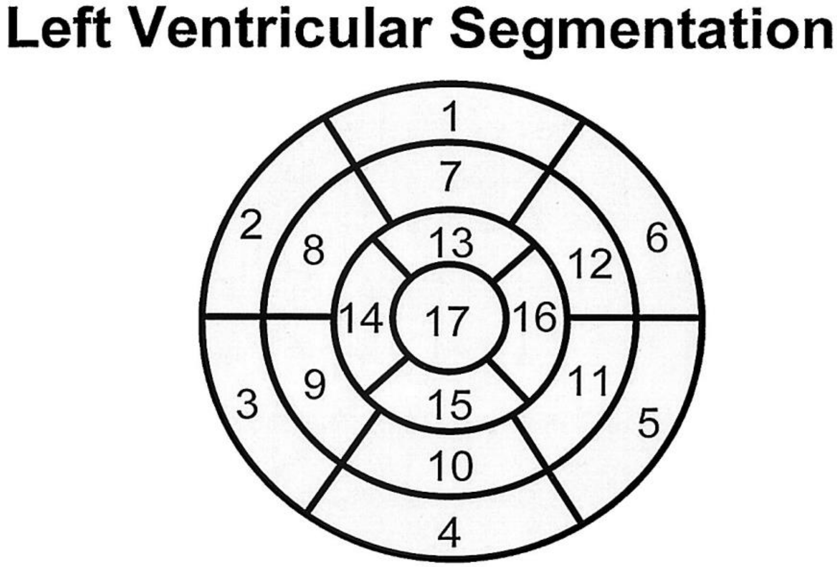

1. basal anterior	7. mid anterior	13. apical anterior
2. basal anteroseptal	8. mid anteroseptal	14. apical septal
3. basal inferoseptal	9. mid inferoseptal	15. apical inferior
4. basal inferior	10. mid inferior	16. apical lateral
5. basal inferolateral	11. mid inferolateral	17. apex
6. basal anterolateral	12. mid anterolateral	

Figure 7: Left ventricular segmentation

6.1.6 *Echocardiography*

Echocardiography will be performed as additional parameter to MRI in accordance with the Schedule of Assessments (Table 2, Assessments I, III, V, VI: Screening, Hospital Discharge, 6-Month Follow-Up (FU), 24-Month FU). The analysis and interpretation of data from the echocardiography concerning LV function parameters will be done centrally by a reviewer blinded to the treatment the patient has received. The date of performance will be recorded in the CRF. The heart rate (per min) and the quality of the images will be documented: good, middle, bad, orthograde ultrasound not measurable. LVEDD (mm), LVEDV (cm^3), LVESD (mm), LVEF (%, four chamber view), IVSD (mm), LVPWD (mm) and LVOT (mm) will be recorded in the CRF. The main area of impaired LV infarction will be recorded in the CRF. Quality assessment of wall movement will be recorded as normal, akinetic, hypokinetic or dyskinetic and the presence of an aneurysma. Pericardial effusion will be assessed: no/yes, if yes: document size (mm) and location (anterior, posterior, lateral, circular).

Furthermore the following has to be documented:

- Mitral regurgitation (none, mild, moderate)
- Tricuspid regurgitation (none, mild, moderate; if mild or moderate: Δ Pmax in mm/Hg)
- Transmital flow: VE in cm/sec, VA in cm/sec, VE/VA < 1or >1; DT (VE) in msec), VTI (LVOT) in cm
- Aortic valve regurgitation (none, mild, moderate), RVEDD (RVOT) in mm, LA (left atrium) in mm, RA (right atrium, 4 CV) in mm
- Unwanted tissue changes; no/yes; if yes, describe

6.1.7 *6-Minute Walk Test (6MWT)*

The 6MWT is a practical simple test that requires a 100-ft hallway (30 m) but no exercise equipment or advanced training for technicians. Walking is an activity performed daily by all but the most severely impaired patients. This test measures the distance that a patient can quickly walk on a flat, hard surface in a period of 6 minutes. It evaluates the global and integrated responses of all the systems involved during exercise, including the pulmonary and cardiovascular systems, systemic circulation, peripheral circulation, blood, neuromuscular units, and muscle metabolism. It does not provide specific information on the function of each of the different organs and systems involved in exercise or the mechanism of exercise limitation, as is possible with maximal cardiopulmonary exercise testing. The self-paced 6MWT assesses the submaximal level of functional capacity. Most patients do not achieve maximal exercise capacity during the 6MWT; instead, they choose their own intensity of exercise and are allowed to stop and rest during the test.

Medication taken within the last 12 hours before the test will be documented.

Blood pressure (mm/Hg), heart rate (beats/min), the saturation of peripheral oxygen ($SpO_{2;}$ %), dispnoe and fatigue assessed by Borg scale and the occurrence of any heart rhythm disturbances (if yes, slight to moderate or severe?) will be documented before

starting the test and at the end of the test. In case of severe heart rhythm disturbances patient must not perform 6MWT!

Borg scale:

0	= nothing at all	5	= severe (heavy)
0.5	= very, very slight	6	
1	= very slight	7	= very severe
2	= slight	8	
3	= moderate	9	
4	= somewhat severe	10	= very, very severe

The documentation of the test performance includes reasons for stopping or early termination of the test and the total distance walked within 6 minutes.
The 6MWT will be performed in accordance with the Schedule of Assessments (Table 2; Screening, Hospital Discharge, 6-Month Follow-Up;) and will be documented in the CRF.

6.2 Quality Of Life Questionnaires

QoL will be assessed using the Short Form Questionnaire (SF-36) and Minnesota Living with Heart Failure Questionnaire (MLHF-Q) and and additionally a standardized questionnaire for clinical and economic appraisal, the EQ-5D. These assessments will be performed in accordance with the Schedule of Assessments (Screening, 3-Month Follow-Up, 6-Month Follow-Up, 24-Month Follow-Up; Table 2).

6.2.1 *Short Form Questionnaire (SF36)*

The Short Form QoL questionnaire with 36 questions is a well-documented, self-administered scoring system measuring health status. It is used to measure subtle changes in health that follow medical interventions, such as surgery, and in allowing comparison of one technique against another (see Appendix).

6.2.2 *Minnesota Living With Heart Failure Questionnaire (MLHF-Q)*

The Minnesota Living with Heart Failure Questionnaire (MLHF-Q) is a disease specific instrument which is composed of 21 items and three scales that measure the following (see Appendix):

- The physical functioning dimension (8 items)
- The emotional functioning dimension (5 items)
- The overall score on health-related QoL (8 items).

6.2.3 *Questionnaire of the EuroQoL group: EQ-5D™*

EQ-5D is a standardized measure of health status developed by the EuroQol Group in order to provide a simple, generic measure of health for clinical and economic appraisal.(User guide v3 April 2010). The EQ-5D essentially consists of 2 pages - the EQ-5D descriptive system and the EQ visual analogue scale (EQ VAS).

The EQ-5D descriptive system comprises the following 5 dimensions: mobility, self-care, usual activities, pain/discomfort and anxiety/depression. Each dimension has 3 levels: no problems, some problems, extreme problems.

6.3 Safety Parameters

6.3.1 *Adverse Events (AEs)*

6.3.1.1 Definitions

6.3.1.1.1 Adverse Events

Any untoward medical occurrence in a patient or clinical investigation subject administered a pharmaceutical product and which does not necessarily have a causal relationship with this treatment is defined as an AE. An AE can therefore be any unfavorable and unintended sign (including an abnormal laboratory finding), symptom, or disease temporally associated with the use of an investigational medicinal product (IMP), whether or not related to the IMP.

6.3.1.1.2 Adverse Reactions (ARs)

Adverse reactions (ARs) include all untoward and unintended responses to an IMP related to any dose administered. All AEs judged by either the reporting Investigator or the Sponsor as having a causal relationship of <u>possibly, probably or definitely related</u> to the IMP qualify as ARs. An AR is defined as <u>unexpected</u> when its nature, severity or outcome is not consistent with the information that has been obtained from previous observations and investigational trials.

6.3.1.1.3 Serious Adverse Events (SAEs)

A SAE is any untoward medical occurrence that at any dose:

- results in death,
- is life-threatening (Life-threatening refers to an event in which the patient was at risk of death at the time of the event; it does not refer to an event, which, hypothetically, might have caused death if it were more serious.),
- requires inpatient hospitalization or prolongation of existing hospitalization,
- results in persistent or significant disability/incapacity,
- is associated with congenital abnormality/birth defect, or
- is another important medical event that may not be immediately life threatening or result in death or hospitalization but, based upon appropriate medical judgment are thought to jeopardize the patient or subject and/or require medical or surgical intervention to prevent one of the outcomes defining a SAE.

6.3.1.1.4 Serious Adverse Reactions (SARs) and Suspected Unexpected Serious Adverse Reactions (SUSARs)

An AR that meets seriousness criteria, defined in Section 6.3.1.1.3, is defined as a serious adverse reaction (SAR). A suspected unexpected (unlisted) serious adverse reaction (SUSAR) is a SAR, the nature or severity of which is not consistent with the applicable product information (Investigator’s brochure [IB]).

6.3.1.2 *Classifications*

6.3.1.2.1 Severity

The severity of AEs will be classified using the modified graduation of the Common Terminology Criteria for Adverse Events (CTCAE V3.0)

0 = no event
I = asymptomatic
II = symptomatic, no treatment necessary
III = symptomatic, specific treatment necessary
IV = life threatening
V = death

6.3.1.2.2 Causality of Adverse Events

The causality of AEs refers to the relationship of the AE to study treatment. When completing the CRF, the Investigator will be asked to assess the causality of the event. Causality will be categorized according to the following criteria:

- Not related:
  An adverse event which (according to the investigator's assessment) is not causally related to the use of the investigational medicinal product
- Unlikely (doubtful):
  An adverse event for which an alternative explanation is more likely, e.g. concomitant medication, concomitant diseases and/or the point at which the event occurred suggests that a causal relationship to the investigational medicinal product is unlikely
- Possible:
  An adverse event which might be caused by the use of the investigational medicinal product. An alternative explanation, e.g. concomitant medication, concomitant diseases, is inconclusive. The relationship in time is reasonable; therefore the causal relationship cannot be excluded
- Probable:
  An adverse event which might be caused by the use of the investigational medicinal product. The relationship in time suggests a causal relationship (e.g. confirmed by dechallenge). An alternative explanation (e.g. concomitant medication, concomitant diseases) is less likely
- Definite:
  An adverse event which is listed as a possible adverse reaction and cannot be reasonably explained by an alternative explanation (e.g. concomitant medication, concomitant diseases). The relationship in time (with the application of the investigational medicinal product) is very suggestive.

6.3.1.3 *Monitoring, Recording and Reporting of Adverse Events*

6.3.1.3.1 General Requirements

During the course of the study, all AEs (including SAEs), irrespective of the relatedness to the study drug, will be recorded on the AE pages of the CRF. During each monitoring visit, the Investigator and the monitor will review all AEs. The Investigator will be

responsible for ensuring that correct information concerning all AEs is included on the appropriate CRF forms.

The following data will be recorded:

- description of the AE: nature, frequency, intensity, time and date of onset and resolution, treatment, outcome, and causal relationship (in the opinion of the Investigator) to the study drug for each AE;
- whether the AE has to be considered as a SAE;
- action taken.

All AEs occurring during the study must be followed-up until the AE has completely resolved, stabilized or the sequelae can be assessed by the Investigator. In case of withdrawal the respective CRF has to be completed.

The results of additional diagnostic measures as the result of an AE, such as laboratory tests, ECG, angiogram, echocardiogram and MRI must be available at site and eventually copied on request.

A flow chart for the reporting of SAEs is shown in Figure 8.

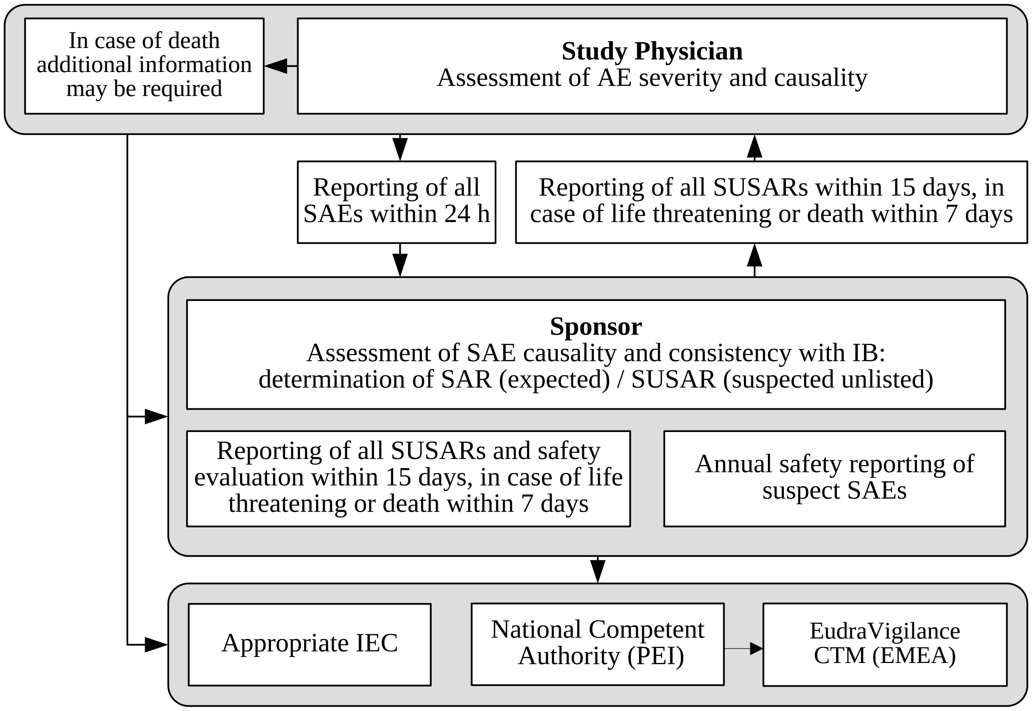

Figure 8: Reporting of Serious Adverse Events

The Investigator reports all SAEs within 24 h to the Sponsor. The Sponsor will report all serious and unexpected adverse drug reactions (SUSARs) in an expedited fashion to all concerned investigators, Independent Ethics Committees (IECs) and the Competent Authority.

6.3.1.3.2 Reporting of Serious Adverse Events

All SAEs that occur during the period of observation starting with signing of the informed consent and ending with the 6-Month Assessment, and all SAEs occurring up to 30 days after receiving the stem cell injection (study drug) in case of withdrawal, whether considered to be associated with the study drug or not, must be reported by the Investigator. A SAE report must be faxed within 24 hours following knowledge of the event by the Investigator to:

SAE Fax: +49 (2204) 8306-3333 (24-hour service)

Medical hotline: +49 (151) 171 51 878

The Investigator should not wait to receive additional information to fully document the event before notifying of a SAE, though additional information may be requested. The minimum information required for an initial report is:

- Sender of report (name, address and phone number of Investigator)
- Date of initial report
- Patient identification (patient number)
- Protocol number
- Investigational medicinal product
- Description of event with outcome, if available, and criteria for seriousness, expectedness and causality

Where applicable, information from relevant laboratory results, hospital case records and autopsy reports should be obtained. The Investigator is also required to submit follow-up reports to Adverse Event Reporting until the SAE has resolved or, in the case of permanent impairment, until the SAE stabilizes. The original SAE form must then be sent by mail to the Clinical Monitor. In addition, the event must be documented in the CRF.

As required, the Sponsor, will report the SAE to the relevant Regulatory Authority and the IECs within the required timeframe, depending on the local regulations. Regulatory agencies will be notified as soon as possible but no later than 7 days after first knowledge of fatal or life-threatening unexpected AR and no later than 15 days after knowledge of the other unexpected SAR.

6.3.1.3.3 Other Procedures for Recording and Reporting Adverse Events

Events with class I and class II severity will be analyzed at the end of the trial period. Events with class III and class IV severity will be reported to the SMB. Details of the SMB are provided in Section 9.9. Class V events must surely be reported to the SMB.

During this study, there will be standard pharmacovigilance monitoring for SARs and SUSARs to allow for annual safety reporting procedures. The Sponsor (or delegate) will be responsible for safety updates to regulatory authorities in accordance with guidelines

and guidance and the German Medicines Act (AMG) and for updating IECs (Independent Ethics Committees) in accordance with the guidelines.

6.3.1.4 Follow-Up of Adverse Events

All AEs experienced by a patient, irrespective of the suspected causality, will be monitored until the event has resolved, any abnormal laboratory values have returned to baseline or stabilized at a level acceptable to the Investigator and the Clinical Monitor, until there is a satisfactory explanation for the changes observed, or until the patient is lost to follow-up.

After class II to IV AEs, patients will remain hospitalized until complete stabilization. The institutions/physicians following the patients during and after convalescence will be informed about the patient’s study participation and advised to increase their level of attention. The study center Investigator will conduct safety-related follow-up examinations on an outpatient basis for a minimum of 6 months or until resolution of these AEs.

6.3.1.5 Adverse Events of Specific Interest

Events that are listed in Appendix 2 are considered to be of specific interest for the purpose of this study and are monitored in detail. Documentation and reporting of Adverse Events of Specific Interest will be according to the standard procedure of SAE reporting in Section 6.3.1.3.2. continuously up to study end and furthermore as safety follow up to Assessment VI after 24 months. After study termination (Assessment V, 6-Month Follow-Up) AEs not listed as AEs of specific interest will not be documented unless they are possibly, probably or definitely related to the investigational medicinal product or result in death. In case of seriousness all possibly, probably or definitely related AEs will be reported at Assessment VI, 24-Month Follow-Up.

6.3.1.6 Major Adverse Cardiovascular Events – MACE

Major adverse cardiovascular events patient alive no/yes, reoperation no/yes, secondary ICU admission no/yes, infarction post-OP no/yes, readmission no/yes, reintervention no/yes, new ventricular arrhythmia no/yes) will be separately assessed at Assessment V (6 months after OP) and 24-Month Follow-Up.

6.3.2 ***The Methods and Timing for Assessing and Recording Other Safety Parameters***

The methods for assessing safety parameters consist of clinical routine methods for observation of patients after CABG surgery (i.e. ECG, laboratory tests including human immunodeficiency virus [HIV], hepatitis B virus [HBV] and hepatitis C virus [HCV] status, Treponema pallidum), plus those that are required by the study protocol (i.e. echocardiography, Holter, ECG). They will be recorded on special AE case report forms.

An obligatory safety follow up is planned for further 18 months after study data closure. Therefore the patients will be required to attend Assessment VI 24 months after the operation. This will be outside the study after data closure. At Assessment VI patient will

undergo examination of all safety parameters and will be asked to complete QoL questionnaires.

6.3.2.1 Laboratory Parameters

Laboratory assessments will be performed locally at each center's laboratory by means of their established methods. Before starting the study, the Investigator will supply the Sponsor with a list of the normal ranges and units of measurement.
The following laboratory parameters will be determined in accordance with the Schedule of Assessments (Table 2).

Table 1: Laboratory Assessments

Hematology: (2,7 mL EDTA)	Hemoglobin (mmol/L or g/dL)	
	Leukocytes (10^9/L or /nL or tsd/µL)	
	Thrombocytes (10^9/L or /nL or tsd/µL)	
Clinical chemistry (5 mL Li-Heparin, electrolytes incl.):	C-reactive protein (CRP) (mg/L or mg/dL)	
	Creatinine (µmol/L or mg/dL)	
	Creatine Kinase (CK) (U/L)	
	Creatine Kinase MB (CK-MB) (U/L)	
	Troponin T (TNT) (ng/mL or µg/L)	
Blood lipids	Total cholesterol (mmol/L or mg/dL)	
	LDL chholesterol (mmol/L or mg/dL)	
	HDL cholesterol (mmol/L or mg/dL)	
	Triglycerides (mmol/L or mg/dL)	
Electrolytes:	Sodium (Na+) (mmol/L)	
	Potassium (K+) (mmol/L)	
NT-proBNP (2.7 mL, EDTA)	NT-proBNP (pg/mL)	
Serology (10 mL serum):	Anti HIV 1/2	positive/negative
	HBsAG	positive/negative
	Anti-HBc-IgG	positive/negative
	Anti HCV	positive/negative
	Treponema pallidum	positive/negative
	if Anti-HBc-IgG positive:	
	HBV-NAT	positive/negative
	Anti-HBs AK	quantified (≥100 IU/L)
HAMA: (2.7 mL serum)	HAMA Titer (≥ or < 1:1000, if applicable)	
Pregnancy test:	Serum pregnancy test in women of childbearing potential only	

Titer of HAMA will only be determined in case of known hypersensitivity. Laboratory abnormalities (normal ranges see **Fehler! Verweisquelle konnte nicht gefunden werden.**) should be recorded as AEs after Investigator judgment.

6.3.2.2 Vital Signs

The following vital signs examination will be assessed in accordance with the Schedule of Assessments (Table 2)

- Supine blood pressure in mm/Hg
- Classification of blood pressure according to the ESC/ESH guidelines 2007
- Resting pulse rate in beats/min
- Body temperature in °C (aural)
- Body weight in kg
- Respiratory rate in breaths/min

6.3.2.3 General Physical Examination

- Examination of head, neck, thorax, abdomen, neurological system, musculoskeletal system, skin, others (except heart) and record any pathological findings (no/yes, if yes: describe)

6.3.2.4 Holter and LOWN Classification

The electrical activity of the heart will be recorded at the Assessments Screening, Hospital Discharge, 6-Month Follow-Up and 24-Month Follow-Up (Table 2). VES in %, SVES in %, incidence of VT, maximum frequency in beats/min, and number will be documented. The occurrence of tachycardial arrhythmia will be assessed according to the LOWN classification. Historic data (not older than 4 weeks) may be used except for Assessment Hospital Discharge.

<u>LOWN Classification of Tachycardial Arrythmia</u>

Class 0	No VES
Class I	Less than 30 VES per hour
Class II	More than 30 VES per hour
Class IIIa	Polytope VES multifocal
Class IIIb	Bigeminy
Class IVa	2 consecutive
Class IVb	≥ 3 consecutive
Class V	R-T Phenomena

6.3.2.5 12-Lead Electrocardiogram

Twelve-lead ECGs will be performed in accordance with the Schedule of Assessments (assessment screening, ICU, Hospital Discharge, 6 months post-OP, 24 months post-OP). ECG measurements should be recorded as AEs or SAEs if there are increases in QT/QTc >500ms or of >60ms over baseline.

- Date of performance
- Frequency beats per min.
- Sinus rhythm yes/no
- Cardiac arrhythmia yes/no

    - Absolute arrhythmia (atrial fibrillation) yes/no, frequency per min.
    - Atrial flutter yes/no
    - SVES yes/no, number
    - Prolonged QT Interval yes/no
    - VES yes/no
    - Bigeminy yes/no
    - Couplets yes/no
    - Triplets yes/no
- AV-Block yes/no, grade I, II, III
- Right bundle branch block yes/no
- Left bundle branch block yes/no
- Isoelectric ST yes/no; if no, elevation/decrease in: V1, V2, V3, V4, V5, V6, I, II, III, aVR, aVL, aVF
- Negative T-waves: yes/no; if yes: terminal/preterminal: V1, V2, V3, V4, V5, V6, I, II, III, aVR, aVL, aVF
- Lack of R-spike: no/yes; if yes: V1, V2, V3, V4, V5, V6, I, II, III, aVR, aVL, aVF
- Pathological Q-Spike: no/yes; if yes V1, V2, V3, V4, V5, V6, I, II, III, aVR, aVL, aVF

6.3.2.6 General Medical History

General medical history of patients will be documented as follows (ascertainment not older than 24 hrs) :

- Head, no/yes, if yes: describe
- Thorax, no/yes, if yes: describe
- Abdomen, no/yes, if yes: describe
- Neurological/psychological history, no/yes, if yes: describe
- Musculoskeletal system, no/yes, if yes: describe
- Blood and hematopoietic system, no/yes, if yes: describe
- Wound infection, no/yes, if yes: describe
- Claustrophobia, yes, if yes: describe

6.3.2.7 Cardiac Medical History

Cardiac medical history will be documented with grade (0 = no event, I = asymptomatic, II symptomatic, but no specific treatment, III = symptomatic and treatment necessary, IV = life-threatening):

- AV-Block I, II, III
- Sinus bradycardia
- Supraventricular arrhythmia
- Ventricular arrythmia
- Vasovagal syncope
- Left ventricular failure
- Myocardial ischemia
- Cerebral ischemia
- Myocarditis

- Pericardial effusion, pericarditis

6.3.2.8 Risk Factors

Risk factors for coronary diseases of the patients will be documented:

- Date of last myocardial infarct
- Angina prior to myocardial infarct, if applicable: duration in months
- Prior myocardial infarcts, no/yes, if yes: how many months ago and localization (anterior, lateral, inferior)
- Prior PCI and/or stent, no/yes; if yes: vessels (LAD territory, LCX territory, RCA territory)
- Heart failure prior to myocardial infarct, no/yes
- Family disposition: arterial hypertension no/yes; Diabetes mellitus no/yes
- Smoking (never, previously, currently); number of pack-years
- EuroSCORE (logistic in %; www.euroscore.org/calcge.html) will be determined

6.3.2.9 Unwanted Tissue Changes

During cardiac MRI and echocardiography-examinations there will be a special check for unwanted tissue changes.

7 STUDY CONDUCT

7.1 Schedule of Observations

The schedule of assessments is provided in Table 2.

Table 2: Schedule of Assessments

Assessments	I<br>Screening	Ia<br>BM transfer/ Cell Prep.	II<br>Intra-OP	IIa<br>Post OP /ICU	III<br>Hospital Discharge [e]	IV<br>3-Month FU [e]	V<br>6-Month FU/ Early termin.	*VI*<br>*24-Month FU*
**Timepoint**	**Day -7 to -1**	**Day -2 to 0**	**Day 0**	**Day 1**	Within 72 hrs of discharge	**Month 3**[e]<br>(± 2 weeks)	**Month 6**<br>(±2 weeks)	*Month 24*[d]<br>*(±2 weeks)*
**Informed consent**	X							
**Patient Demographics**	X							
**Inclusion/exclusion criteria**	X							
**Medical history: general/cardiac /risk factors**	X							
**Physical examination: general/vital signs**	X				X		X	*X*
**Cardiac examination:**								
**NYHA and CCS criteria**	X				X		X	
**Heart catheterization**	X							
**Holter (VES, SVES), LOWN**	X				X		X	*X*
**12-lead ECG**	X			X	X		X	*X*
**Cardiac MRI scan**	X				X		X	
**Echocardiography**	X				X		X	*X*
**Laboratory:**								
**Serology**	X							
**Serum pregnancy test**[c]	X							
**HAMA**[g]	X							
**Hematology, Chemistry, Electrolytes**	X			X[b]	X		X	*X*
**Blood Lipids**	X							
**NT-proBNP**	X				X		X	*X*
**QoL questionnaires** [a]	X					X	X	*X*
**6MWT**	X				X		X	
**Bone marrow aspiration/blood sample**		X						
**Randomization to treatment**		X						
**Cell/placebo preparation**		X						
**Cell/placebo transfer**		X						
**Injections of study treatment/ CABG**			X					
**MACE**							X	*X*
**Concomitant medications**	X	X	X [f]	X	X	X	X	*X*
**Adverse events/Serious Adverse Events**	X	X	X	X	X	X	X	*X*

[a] = SF 36 & MLHF-Q, EQ-5D; [b] = 36 hours after OP; after 0, 6, 12, 24 hours only CK, CK-MB, TNT. [c] = For female patients of childbearing potential only, [d] = 18 months after study finalization , [e] = patients receive dates for follow-ups, [f] = surgery standard medication will not be documented, [g] = only for patients with known hypersensitivity or sensitization against murine products;

Abbreviations: CCS= Canadian Cardiovascular Society, ECG=electrocardiogram, MACE= major adverse cardiovascular events, MRI= magnetic resonance imaging, NYHA=New York Heart Association, 6MWT=6-minute walk test, QoL=Quality of Life, SVES=supraventricular extrasystoles, VES=ventricular extrasystoles;

7.2 Observations by Visit

7.2.1 *Screening (Assessment I; Day -7 to Day -1)*

- Inform patient about clinical trial (record in appropriate documentation)
- Obtain written informed consent, record date and time of informed consent (see Section 9.7)
- Demographic details: height in cm, year of birth, gender
- General medical history (see Section 6.3.2.6)
- Cardiac medical history (see Section 6.3.2.7)
- Risk factors (see Section 6.3.2.8)
- In- and exclusion criteria (see Sections 4.1& 4.2)
- Vital signs (see Section 6.3.2.2)
- General physical examination (see Section 6.3.2.3)
- Blood sampling for serology (see Section 6.3.2.1)
- Blood sampling for laboratory tests (see Section 6.3.2.1)
- Blood sampling for NT-proBNP (pg/mL; to be sent to Core Lab) and HAMA in case of known hypersensitivity (see Section 6.3.2.1)
- Serum pregnancy test (female patients of childbearing potential only, see Section 6.3.2.1)
- Cardiac MRI scan and echocardiography to be sent to Core Lab (see Sections 6.1.1& 6.1.2, 6.1.5 & 6.1.6), value for LVEF within 12 hours from MRI Core Lab
- Classification of heart failure with NYHA and angina with CCS (see Section 6.1.3)
- Heart catheterization (see Section 6.1.4)
- Holter and LOWN classification (historic data may be used, see Section 6.3.2.4)
- 12-lead ECG (see Section 6.3.2.5)
- Completion of QoL questionnaires (MLHF and SF-36; see Section 6.2)
- 6-Minute Walk Test (6MWT, see Section 6.1.7)
- Documentation of AEs/SAEs during screening procedure (see Section 6.3.1)
- Concomitant medication, platelet aggregation inhibitors during the last 2 weeks (see Section 5.7)
- Determine patient’s eligibility for enrollment (PI and study physician or at least two study physicians). Give reason in case patient is not eligible.

7.2.2 *Bone Marrow Transfer /Preparation of Cell Product/Placebo (Assessment Ia; Day -2 to 0)*

- Bone marrow aspiration and 20 mL blood sample collection for preparation of autologous serum (see Section 5.8). Transferring data in form "Transfer Protocol":
  - Serologic data (seronegative/seropositive)
  - Date and time of bone marrow harvest
  - Bone marrow volume (mL)
  - Date and time of blood sample taking
  - Blood sample volume
  - Date, time of start (handover to carrier) and end of transport from OP to stem cell laboratory

- Transfer of cells to the stem cell laboratory for processing
- Randomization to study treatment group performed by the cell processing laboratory
- Cell/placebo preparation according to validated SOPs
- Transfer of investigational drug from stem cell laboratory to OP with "Transfer Protocol"
- Documentation of data in "Transfer Protocol"
    - Date, time of start and end of transport from stem cell laboratory to OP at study site
    - Date and time of receiving investigational drug at OP, department, names of study nurse and physician
    - Confirmation of receipt of sealed randomization code envelope
    - Copy of "Transfer Protocol" into CRF
- Documentation of any change in concomitant medication
- Documentation of any AE/SAE since last assessment
- Data transfer into ”Cell Preparation Form” to be performed after last patient out only:
    - Allocation of randomization No. to $CD133^+$ cell preparation group or placebo group
    - Volume of bone marrow aspirate after filtration (200 µm) in mL
    - Volume of “final product” after resuspension in saline/autologous serum in mL
    - Recovery of CD 133^+ cells in “final product” compared to bone marrow
    - Log depletion of non-target cells
    - Total viable CD 133^+ cells in 5 1mL syringes (“Final product ready for injection”)
    - Bacterial contamination: results of quick test and sterility test

7.2.3 *Intra OP: Surgical Operation (Assessment II; Day 0)*

<u>During the CABG Operation</u>

- Date of surgery, time start and end of OP
- Number and distribution (RIVA, RCX, RCA) of distal anastomoses
- Number of myocardial injections of study treatment (15 injections planned; see Section 5.2 and Figure 5)
- Quantity of 5 mL completely injected, documentation of actual quantity (if applicable)
- Localization of injections according to Figure 5
- Bypass time (min.)
- Cross clamp time (min.)
- Documentation of low cardiac output (if yes: cardiac output < 2 (L/min/m^2) body surface? no/yes
- Documentation of mechanic assist device if applicable
- Documentation of concomitant medication for treatment of an AE/SAE; standard medication (narcotics etc.) will not be documented,
- Documentation of any AE/SAE since last assessment

7.2.4 *Post OP/Intesive Care Unit (Assessment II a; Day 0 to Day 4 approx.)*

Post-operation/ICU

Blood sampling for laboratory tests at 0, 6, 12, 24 hours post-operation (only three values)

- Clinical Chemistry:
  - Creatine Kinase (CK) (U/l)
  - Creatine Kinase MB (CK-MB) (U/l)
  - Troponin T (TNT) (µg/l)
- Blood sampling for laboratory tests at 36 hours post-operation
  - Hematology (see Table 1)
  - Clinical Chemistry (all, see Table 1)
  - Electrolytes (see Table 1)
- Performance of 12-lead ECG (see Section 6.3.2.5)
- Documentation of any perioperative infarction
  - If applicable: Localization of myocardial infarct (LV/RV; if LV: septal, anterior, posterior, lateral, anterolateral)

End of Intensive Care Unit

- Length of stay (days)
- Ventilation (hours)
- Catecholamines and Inotropics (adrenaline, noradrenaline, dopamine, dobutamine, PDE-inhibitor, Ca-sensitizer; maximum in µg/kg/min)
- Documentation of concomitant medication for treatment of an AE/SAE
- Documentation of any AE/SAE since last assessment

7.2.5 *Hospital Discharge: (Assessment III; within 72 hrs of discharge)*

- Vital signs (see Section 6.3.2.2)
- General physical examination (see Section 6.3.2.3)
- Blood sampling for laboratory tests (Hematology, Clinical Chemistry and Electrolytes; see Section 6.3.2.1)
- Blood sampling for NT-proBNP (pg/mL; to be sent to Core Lab; see Section 6.3.2.1)
- Cardiac MRI scan and echocardiography to be sent to Core Lab (see Sections 6.1.1& 6.1.2, 6.1.5 & 6.1.6)
- Classification of heart failure with NYHA and angina with CCS (see Section 6.1.3)
- Holter and LOWN classification (see Section 6.3.2.4)
- 12-lead ECG (see Section 6.3.2.5)
- 6-Minute Walk Test if reasonable (6MWT, see Section 6.1.7)
- Date of discharge, where to (home, other hospital, rehabilitation)
- Documentation of any change in concomitant medication
- Documentation of any AE/SAE since last assessment
- Provide patient with dates for telephone call after 3 months post-OP (study pass)
- Provide patient with dates for cardiac MRI, echocardiography, and Holter appointments after 6 months post-OP (study pass)

7.2.6 *Follow-Up Period*

Prior to Assessment IV QoL questionnaires and addressed envelope should be sent to patients so that they can complete the questionnaires at home.

7.2.6.1 3-Month Follow-Up post OP (± 2 weeks, Assessment IV, per telephone)

- Date of telephone call
- Patient able to perform assessment? If not, give reasons: hospitalization or unknown (in case patient has deceased, do not fill out 3-Month Follow-Up but proceed to 6-Month Follow-Up/Earl Termination)
- Retrieve QoL questionnaires; if not returned to hospital at time of Assessment IV remind patient to complete form (see Section 6.2)
- Confirmation of dates for next follow-up
- Documentation of any change in concomitant medication
- Documentation of any AE/SAE since last assessment

7.2.6.2 6-Month Follow-Up post OP (± 2 weeks, Assessment V)/Early Termination

- Study termination
    - has the study been terminated according to protocol? If yes, proceed with Physical examination/vital signs
    - If study was early terminated:
        - Date of withdrawal/early termination
        - Reason for withdrawal/early termination (AE, at the discretion of the investigator, violation of eligibility criteria, deviation from the treatment plan specified in the protocol, pregnancy, patient withdraws consent, death, other (describe)
- Date of patient contact
- Vital signs (see Section 6.3.2.2)
- General physical examination (see Section 6.3.2.3)
- Blood sampling for laboratory tests (Hematology, Clinical Chemistry and Electrolytes; see Section 6.3.2.1)
- Blood sampling for NT-proBNP (pg/mL; to be sent to Core Lab; see Section 6.3.2.1)
- Cardiac MRI scan and echocardiography to be sent to Core Lab (see Sections 6.1.1& 6.1.2, 6.1.5 & 6.1.6)
- Classification of heart failure with NYHA and angina with CCS (see Section 6.1.3)
- Holter and LOWN classification (historic data may be used, see Section 6.3.2.4)
- 12-lead ECG (see Section 6.3.2.5)
- Determination of MACE (see Section 6.3.1.6)
- Completion of QoL questionnaires (MLHF and SF-36; see Section 6.2.)
- 6-Minute Walk Test (6MWT, see Section 6.1.7)
- Documentation of any change in concomitant medication
- Documentation of any AE/SAE since last assessment

7.2.6.3 24-Month Follow-Up post OP (± 2 weeks, Assessment VI after data closure for safety reason)

- Date of patient contact
- Vital signs (see Section 6.3.2.2)
- General physical examination (see Section 6.3.2.3)
- Echocardiography from practice based cardiologist (see Sections 6.1.1, 6.1.2, & 6.1.6)
- Holter from practicing cardiologist and LOWN classification (see Section 6.3.2.4)
- Blood sampling for laboratory tests (Hematology, Clinical Chemistry and Electrolytes; see Section 6.3.2.1)
- Blood sampling for NT-proBNP (pg/mL; to be sent to Core Lab; see Section 6.3.2.1)
- Completion of QoL questionnaires (MLHF and SF-36; see Section 6.2.)
- Performance of 12-lead ECG (see Section 6.3.2.5)
- Determination of MACE (see Section 6.3.1.6)
- Documentation of concomitant medication related to treatment of AE/SAE
- Documentation of any AE/SAE and reporting of any SAE that is related to the investigational product (relatedness 3 to 5) since last assessment; documentation of any MACE and any Event of Specific Interest since last assessment (reporting only in case of relatedness 3 to 5); see Section 6.3.1, 6.3.1.5, and 6.3.1.6

8 STATISTICAL METHODS

Before unblinding, a separate statistical analysis plan (SAP) will be finalized, providing detailed methods for the analyses outlined below. Analyses will be performed after database lock.
Any deviations from the planned analyses will be described and justified in the final integrated study report.

8.1 Study Patients

8.1.1 *Disposition of Patients*

The number and percentage of patients entering and completing each phase of the study will be presented, stratified by treatment. Reasons for withdrawal pre- and post-randomization will also be summarized.

8.1.2 *Protocol Deviations*

Every deviation from the trial protocol must be specified and documented in writing separately for each patient. The Investigator has to report the protocol deviation to the sponsor. The Investigator must consult with the monitor and discuss the type and extent of deviation as well as the possible consequences for further participation of the patient in the study. If the evaluability of a patient is questionable the national coordinating Investigator, the trial site principal Investigator and the biometrician will be consulted. Major violations leading to exclusion from the PPS (Per Protocol Set) will be defined during the blind review meeting and in agreement with the Sponsor after data cleaning but before database closure and before breaking the randomization code.
One of the following surgical complications will lead to post-hoc exclusion from the study:

- Perioperative cerebral ischemic insult with persisting relevant neurologic deficit
- Deep sternal wound infection
- Rare surgical complications (i.e. respiratory failure with need for prolonged mechanical ventilation, intestinal infarction, several acute pancreatitits, chronic chylothorax, aortic dissection, peripherical ischemia requiring surgery)

Patients with one or more major protocol violations will be excluded from PPS group leading to the exclusion of all efficacy data from the PPS analysis, if one of the following criteria is fulfilled:

- Patients without measurement after 6 months for LVEF
- Violation of inclusion or exclusion criteria.

Number of patients with protocol deviations during the study and listings describing the deviations will be provided. Patients that are not attendant at study visits or refuse to undergo certain assessments are non-compliant with the study protocol. Listings and frequency tables of those patients will be provided.
The insufficiency of $CD133^{+}$ cell counts (less then $0.5x10^{6}$ but $\geq 0.1\ x10^{6}$) will lead to post-hoc exclusion from the study but patients will be followed-up similar to the intend-to-treat population.

8.1.3 *Analysis Populations*

Two different sets of patients will be defined to perform analyses. A “Full Analysis Set” (FAS) following the principle of intent-to-treat (ITT) will include every patient as randomized and compare the patients according to the group to which they were randomly allocated, regardless of patients' compliance, or withdrawal from the study. It is the most cautious approach and so minimizes type 1 error, helps preserve prognostic balance in the study arms and allows for the greatest generalizability.

Secondly, an analysis of all per protocol treated patients will be performed. The “Per Protocol Set” (PPS) is a subset of the FAS patients who are compliant with the study protocol. The PPS sample will consist of all subjects from the FAS group without any major protocol violation.

First and foremost, confirmatory analyses on primary efficacy variable will be performed on the FAS patients but a secondary analysis will also be performed based upon the Per Protocol Set, to assess the sensitivity of the analysis to the choice of analysis population. The ITT analysis will be considered as the primary one.

Patients who received the cellular product or Placebo but dropped-out post-hoc because of cell count insufficiency will be evaluated separately.

The safety population will comprise all patients randomized into the study, in which each patient’s treatment is as taken on the study. Safety evaluations will be performed on the safety population.

8.2 General Considerations

In statistical analyses no missing values will be replaced.

All statistical analyses are performed by the Institute of Medical Informatics and Biometry of the University of Rostock. Statistical analyses will be carried out using the SPSS statistical analysis system, version 14.0.

All statistical tests will be conducted using a two-sided test with $\alpha=0.05$, unless otherwise stated.

For all data collected during the trial and reported in the CRF describing the sample, listings of the individual raw data as well as tables of sample characteristics and/or frequencies will first be given. Continuous data will be summarized first by treatment group, secondly by certain study time points, thirdly by each study center using descriptive statistics (mean, median, standard deviation [SD], minimum and maximum, number of available observations and number of missing observations). It will be done for absolute and percent changes and if appropriate from baseline screening too.

Categorical data will be summarized first by treatment group, secondly by certain study time points, thirdly by each study centers. The numbers and percentages of each of categories, the number of available observations and the number of missing observations will be presented in frequency tables.

Any outliers that are detected during the blind review of the data will be investigated. Methods for dealing with outliers will be defined in the SAP or in an addendum to the SAP, prior to unblinding.

The statistical analysis of study results will be performed according to the CPMP guidelines for “Biostatistical methodology in clinical trials in applications for marketing authorizations for medicinal products” and the ICH guideline “Statistical principles for clinical trials”.

8.3 Demographics, Baseline Characteristics and Concomitant Medications

Demographic data, medical history, concomitant disease and concomitant medication will be documented and analyzed. All data will be summarized by means of descriptive statistics (mean, SD, median, minimum, maximum, number of available observations and number of missing observations) or frequency tables, stratified by treatment.
For all data collected during the procedure, listings of the individual raw data as well as tables of sample characteristics and/or frequencies will be given. Graphic presentations will be produced.

8.4 Treatment and Study Compliance

Treatment will be administered by study personnel hence patient compliance with study treatment will not be monitored (comp. Section 5.6) and analyzed.
Patients that are not attendant at study visits or refuse to undergo certain assessments are non-compliant with the study protocol. Listings and frequency tables of those patients are provided.

8.5 Efficacy Analyses

The trial will be conducted in seven study sites. Therefore centers will be considered as possible prognostic factor influencing the outcome.
With regard to possible baseline and study site effects, the two-sided hypothesis for the continuous primary efficacy variable LVEF at 6 months postoperatively (comp. section 6.1.1) will be assessed using analysis of covariance (ANCOVA) adjusting for baseline LVEF and study sites.
Secondary efficacy variables (comp. section 6.1.1) will be analyzed in a strictly explorative way. The distance walked pre-op and post-op will be compared. If p-values are computed, no adjustment for multiple testing will be made and they will be interpreted in the exploratory sense. Similarly, confidence intervals computed will be interpreted as interval estimates for presence or absence of effects in the study data.
In order to check differences between the treatment groups for the fifth secondary endpoint (death, myocardial infarction, need of reintervention) an unadjusted survival analysis with Kaplan-Meier estimations will be performed using the logrank test.
Details of the statistical analyses will be documented in SAP (Statistical Analysis Plan) that is to finalize before unblinding.

8.6 Safety Analyses

Adverse events (AEs) and serious adverse events (SAE) (comp. Sections 6.3), will be listed summed by occurence, severity (comp. Section 6.3.1.2.1), outcome, and causal relationship to treatment (comp. Section 6.3.1.2.2) and will be descriptively compared between the two treatment groups using Fishers exact test. Associated AE tables present the total number of patients reporting at least one specific event and the maximum severity grade (patients reporting more than one episode of the same event are counted only once by the maximum severity and highest causality). Special tables will be displayed for MACE (comp. Section 6.3.1.6) and for AEs leading to withdrawal.
In addition, summary tables with patient identification will be presented. The tables provide the numbers and percents of patients with AEs, and also include the subject identification in the table.

Separate summarizations of AEs by worst severity, and of SAEs and AEs that are likely related to treatment and AEs that are unlikely related to treatment (comp. Section 6.3.1.2.2) will also be provided. All AEs will be listed.
Descriptive statistics for laboratory parameters (comp. Table 1, Section 6.3.2.1) will be presented by treatment group and time point. For continuous laboratory parameters, changes from baseline to the other time points will be presented by treatment group and descriptive statistics will be calculated.
Values will also be presented according to the CTC categories. Values outside corresponding normal ranges will be displayed and tabulated.
A new abnormality is defined as an out-of-range value that was previously normal or a value that is initially out of range in one direction (either low or high) and becomes out-of-range in the opposite direction at the end of the period. A worsened abnormality is a value that was out of range at the start and became more abnormal during the period. Should the rate of new or worsened abnormalities be deemed excessive, further examination of the nature of the abnormalities will be performed.
Changes in vital signs (comp. Section 6.3.2.2) and electrocardiograms will also be examined for treatment group differences.

8.7 Determination of Sample Size

Considerations for determining the sample size of the primary efficacy parameter LVEF at 6 months postoperatively, measured by MRI at rest, were carried out on the basis of the results of a previous efficacy pilot trial with n=20 patients per group conducted in Rostock by Steinhoff and co-workers (Stamm et al, 2007). For LVEF at 6 months postoperatively the results were 47.1±7 for patients of the CABG & cell injection group and 41.3±8 for patients of the CABG only group.
The stratification of the primary analysis by center is neglected in the sample size calculation. Instead of the analysis of covariance (ANCOVA) to be used in the primary analysis, the two-sample *t* test scenario with equal variances is considered.
Sample size determination is done under the assumption of a two-sided type I error (α) at 5% and a type II error (β) at 10% (i.e. a power at 90%). Since there is some uncertainty regarding the expected means and standard deviations Table 3 presents how many patients per group are necessary for several scenarios, e.g. to show a difference of 3.5 to 5.5 between the two groups.
The scenario of a difference of about 4 to 5 is considered as a clinical relevant difference. With a difference of 4.5 and a standard deviation of 7.5, at least n=60 patients per group are necessary and with an additional 15% drop-out rate a total of at least 142 patients will be randomized.
For n=60 patients per group, Table 4 presents the power for some other scenarios of mean and standard deviation.

Sample size calculation was done using the commercial program nQuery Advisor 5.0, section 8, table MTT0-1 [Elashoff, 2002]. Computation was realized using central and non-central t-distribution where the non-centrality parameter is $\sqrt{n}\,\delta/\sqrt{2}$ and δ is defined as effect size $|\mu_1-\mu_2| / \sigma$ [O´Brien RG, Muller KE, 1993].

Table 3: Number of Patients Necessary to Show Differences of the Primary Efficacy Parameter LVEF at 6 Months Postoperatively (Two-Sided, α = 0.05, 1 – β = 0.90)

LVEF 6 months postoperatively<br>Difference of means between CABG & cell injection group and CABG only group (standard deviation within both groups)	Number of patients per group
3.5 (7)	86
3.5 (7.5)	98
3.5 (8)	111
4 (7)	66
4 (7.5)	75
4 (8)	86
4.5 (7)	52
**4.5 (7.5)**	**60**
4.5 (8)	68
5 (7)	43
5 (7.5)	49
5 (8)	55
5.5 (7)	36
5.5 (7.5)	41
5.5 (8)	46

Table 4: Power with N=60 Patients per Treatment Group for Several Differences of Means and Standard Deviations

LVEF 6 months postoperatively<br>Difference of means between CABG & cell injection group and CABG only group (standard deviation within both groups)	Power
3.5 (7)	77
3.5 (7.5)	71
3.5 (8)	66
4 (7)	87
4 (7.5)	82
4 (8)	77
4.5 (7.)	93
**4.5 (7.5)**	**90**
4.5 (8)	86
5 (7)	97
5 (7.5)	95
5 (8)	92
5.5 (7.)	98
5.5 (7.5)	97
5.5 (8)	96

8.8 Interim Analysis

An interim analysis will be performed on the first 70 patients randomized and followed-up for at least 6 month using the adaptive two-stage approach described by Bauer and Köhne, 1994.

At the first interim analysis the null hypothesis H_{01} can be rejected if

$$p_1 \leq \alpha_1,$$

where p_1 is the (one-sided) p-value from the t-test and α_1 the critical value.

The trial will be stopped for futility if

$$p_1 \geq \alpha_0,$$

where α_0 is the stopping boundary for futility.

If the trial will be continued reassessment of the sample size will be performed.

At the second stage the null hypothesis H_{02} can be rejected if

$$p_2 \leq \alpha_2,$$

where p_2 is the (one-sided) p-value from the t-test and $\alpha_2 = c_\alpha / p_1$.

The sample size n_2 for the second stage will be assessed by considering observed variability and effect of the first stage.

At the end of the trial the null hypothesis H_0 will be rejected if

$$p_1 \bullet p_2 \leq c_\alpha$$

where c_α is the critical value for the combination test.

The effect size of the primary efficacy parameter will be calculated in a semi-blinded manner. The sample size calculation for the second step will be performed using ADDPLAN (Wassmer & Eisebitt, 2001).

Overall α-level = 0.025 (one-sided), $\alpha_0 = 0.5$, $\alpha_1 = 0.0102$, $c_\alpha = 0.0038$

The following secondary endpoints will be evaluated descriptively at the first stage:

- Change in LVEF at 6 months post-OP compared with preoperatively (screening) assessed by echocardiography
- Change in physical exercise capacity determined by 6 minute walk test at 6 months compared with preoperatively
- Change in NYHA and CCS class at 6 months post-OP compared with preoperatively

Interim analysis for safety

- MACE (cardiac death, myocardial infarction, secondary intervention/reoperation, ventricular arrhythmia).

Conduct of interim analysis

All staff involved in the interim analysis or with access to the unblinded data should not participate in the conduct of the study or in the blind review of the data at the end of the study. Therefore the unblinded interim analysis will be performed by an independent statistician outside the clinIT AG. Dr. Tibor Schuster who is associated member of the SMB will be appointed as independent statistician. The interim analysis will be performed by Dr. Schuster in a semi-blind manner. The randomization code of the subjects who will be analyzed in the interim analysis will be provided to the independent statistician by Miltenyi Biotec. The results of the interim analysis will be provided to the steering committee for decision purpose

Data Flow Display

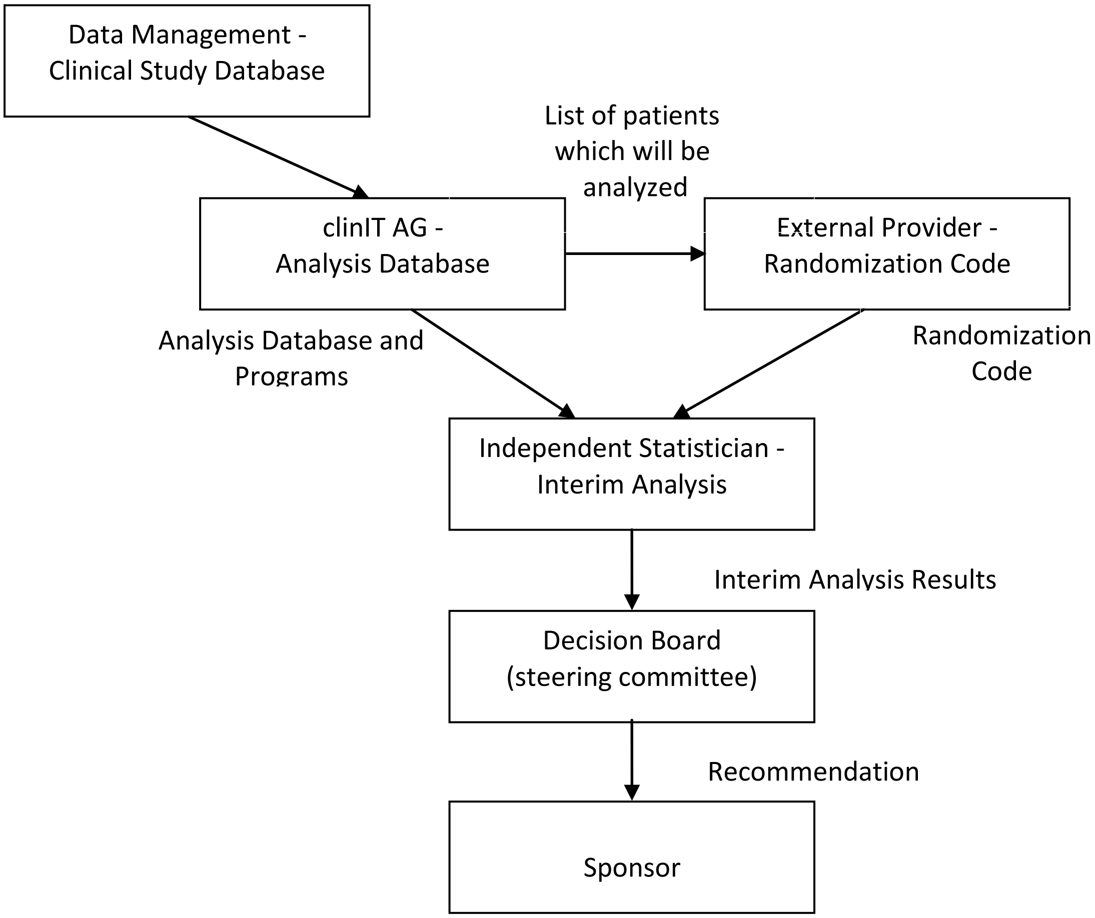

9 ETHICAL, LEGAL AND ADMINISTRATIVE ASPECTS

9.1 Data Quality Assurance

The Sponsor will conduct a site visit to verify the qualifications of each Investigator, inspect the site facilities, and inform the Investigators of responsibilities and the procedures for ensuring adequate and correct documentation.

The Trial Site Principal Investigator of each trial site will conduct a site visit to verify the qualifications of each Investigator. Before beginning the study there will be an Investigator meeting for training on the protocol, GCP, etc.

Each study center will be attended by a clinical monitor. The clinical monitor is qualified by his/her training and experience to oversee the conduct of the study. The principle Investigators and the Investigators agree with and support the monitoring procedure as described in the protocol.

The Investigator is required to prepare and maintain adequate and accurate case histories designed to record all observations and other data pertinent to the study for each study participant. All information recorded in the CRF for this study must be consistent with the patients' source documentation (i.e., medical records).

To ensure data quality and completeness the following will be adhered to:

- Fully documented Individual Case Safety Reports (ICSRs) in the data base
- Diligent follow-up of each case
- Retain reporter's verbatim AE terms (documenting any Sponsor differences)
- Consistent and accurate codification of reported terms.

The responsible person in the central stem cell laboratory will be primarily responsible for the quality and safety of the cell product. The quality control and safety data will be subject to secondary post-hoc monitoring by the Clinical Monitor. Should the Clinical Monitor detect a violation of the quality control and product release standards, he/she will exclude the patient from the study and immediately inform the local Investigators so that appropriate medical measures can be taken.

The general coordination of the pooling for the study will be performed by the Coordinating Investigator Prof. Dr. med. G. Steinhoff from the University of Rostock.

9.2 Case Report Forms and Source Documentation

All relevant data collected during the study for all of the patients enrolled in the study will be recorded in CRF. The data will be entered by the responsible Investigator or someone authorized by him in a timely manner.

The physician will confirm the completeness, correctness and plausibility of the data by his signature. All source documents from which CRF entries are derived should be placed in the patient's medical records. Measurements for which source documents are usually available include laboratory assessments, 12-lead ECG recordings, cardiac MRI scans, echocardiography and Holter.

The original data in the CRF for each patient will be checked against source documents at the study site by the clinical monitor. Additions and corrections will be dated and signed by the responsible physician or an authorized person. Reasons must be given for corrections that are not self-explanatory.

All data are stored in a central database. Instances of uninterpretable data will be discussed with the Investigator for resolution.

The original data of MRI and ECHO will be recorded directly with standardized methods and materials and stored digitally. Data will be transferred electronically to core laboratories for analyses. Analyzed data will be entered by the study personnel into the CRF.
If corrections and/or additions are needed after checking the CRF, a corresponding query must be formulated and forwarded to the physician for his response.

9.3 Access to Source Data

During the course of the study, a monitor will make site visits to review protocol compliance, compare CRF and individual patient's medical records, assess drug accountability, and ensure that the study is being conducted according to pertinent regulatory requirements. CRF entries will be verified with source documentation. The review of medical records will be performed in a manner to ensure that patient confidentiality is maintained.
Checking of the CRF for completeness and clarity, and cross-checking with source documents, will be required to monitor the progress of the study. Regulatory authorities, IECs, and other authorized persons may wish to carry out source data checks and/or on-site audit inspections. Direct access to source data will be required for these inspections and audits. They will be carried out giving due consideration to data protection and medical confidentiality. The Investigator assures the Sponsor of the necessary support at all times.

9.4 Data Processing

All data will be entered in a central data base.
The data-review and data-handling document, to be developed during the initiation phase of the study, will include specifications for consistency and plausibility checks on data and will also include data-handling rules for obvious data errors. Query/data correction forms for unresolved queries will be cleared for resolution with the Investigator. The database will be updated on the basis of signed corrections.

9.5 Archiving Study Records

According to ICH guidelines, essential documents should be retained for a minimum of 2 years after the last approval of a marketing application in an ICH region and until there are no pending or contemplated marketing applications in an ICH region or at least 2 years have elapsed since the formal discontinuation of clinical development of the investigational product. The essential documents of this study will be retained for a longer period if required by the applicable legal requirements.

9.6 Good Clinical Practice

The procedures set out in this study protocol are designed to ensure that the Sponsor and Investigators abide by the principles of the Good Clinical Practice guidelines of the ICH, and of the Declaration of Helsinki (2013). The study also will be carried out in keeping with local legal requirements.

9.7 Informed Consent

Patients who are eligible for enrolment into the study will be informed by their surgeon in detail about the trial. Patients will be allowed adequate time for consideration and making an informed decision, ideally 24 hours. During this period patients will have the opportunity to discuss questions and concerns with their surgeon. In patients willing to participate in the trial, informed consent will be obtained from the patient (or his/her legally authorized representative) according to the regulatory and legal requirements of Germany. This consent form must be dated and retained by the Investigator as part of the study records. The Investigator will not undertake any investigation specifically required only for the clinical study until valid consent has been obtained. The date and time when consent was obtained will also be documented in the CRF.
According to the German Medicines Act, under-age patients must personally provide informed consent (in addition to their legal representatives) provided they are able to understand the information given to him/her in this study. This is not relevant for this study, as under age persons are excluded.
The explicit wish of a minor, or mentally incapacitated adult, who is capable of forming an opinion and assessing the study information, to refuse participation in or to be withdrawn from the study at any time will be considered by the Investigator.
Patients can withdraw their consent at any time point during the study period.
If a protocol amendment is required, the informed consent form may need to be revised to reflect the changes to the protocol. If the consent form is revised, it must be reviewed and approved by the appropriate IEC/PEI, and signed by all patients subsequently enrolled in the study as well as those currently enrolled in the study.

9.8 Protocol Approval and Amendment

Before the start of the study, the study protocol and/or other relevant documents will be approved by the IEC and Competent Authorities (PEI), in accordance with German legal requirements. The Sponsor must ensure that all ethical and legal requirements have been met before the first patient is enrolled in the study.
This protocol is to be followed exactly. To alter the protocol, amendments must be written, receive approval from the appropriate personnel, and receive IEC and Competent Authority (PEI) approval prior to implementation (if appropriate).
Administrative changes (not affecting the patient benefit/risk ratio) may be made without the need for a formal amendment. All amendments will be distributed to all protocol recipients, with appropriate instructions.

9.9 Safety Monitoring Board

A Safety Monitoring Board (SMB) will be convened for this study. Its duty will be regularly to review the progress of the study and assess the accumulating safety data from the study. It will, after meeting, advise the Sponsor on the continuing safety of current participants in the study and on the continuing validity and scientific merit of the study. All decisions about the conduct of the study will rest solely with the Sponsor.

The SMB will also be involved by the Statistician, if the drop-out rate exceeds the calculated limit.

SMB procedures will be described in the SMB Charter, which will be approved by the Sponsor and by each Board member.

The SMB will consist of three medical experts in the field:

- **Giulio Pompilio**, **M.D. Ph.D.**., Foundation Heart Center Monzino, University of Milano, Italy. - Department of Cardiovascular Surgery – Centro Cardiologico Monzino IRCCS Via Parea 4, Milano 20138, Italy. Phone: +39 (02)58002562 – Fax: +39 (02)58011194 - e-mail: giulio.pompilio@ccfm.it
- **Warren Sherman**, **M.D., F.A.C.C., F.S.C.A.I.** Cardiologist, Columbia University, Medical Center, N.Y., – College of Physicians & Surgeons of Columbia University
  177 Fort Washington Avenue – MHB 7GN-435 – New York, NY 10032, USA
  Phone: +1 (212) 305-0423 – e-mail: Warren.Sherman@msnyuhealth.org
- **Francesco Siclari**, **M.D. Ph.D.**, Heart Center, Lugano, Switzerland.
  Cardiocentro Ticino – Via Tesserete 48 – CH-6900 Lugano
  Phone: +41 (0) 91 805 31 44 – Fax: +41 (0) 91 805 31 48
  e-mail: francesco.siclari@cardiocentro.org

The documentation to SMB contains detailed clinical information about the SAEs and the clinical course in written reports. Events with class III, class IV and V severity will be reported to the SMB. No member of the SMB will belong to Team A or B and all will be blinded to the randomization of the study treatment. If the SMB notes that the frequency of class III, IV and/or V events in the entire study or a given center exceeds that which is generally expected during CABG surgery, it will request unblinding of the affected patients. If SMB detects that the frequency of AEs exceeds the expected frequency irrespective of the study group allocation, it will review all cases with the local Investigators and determine adequate conduct of the study procedure. If the SMB notes the frequency of AEs exceeds the expected frequency only in the cell treatment group, it will unblind and review the data of the other study centers. Should the overall frequency of class III, class IV or class V AEs in the cell treated group be significantly ($p<0.05$ by chi-square test) higher than in the control group, the SMB will terminate the trial.

9.10 Duration of the Study

For an individual patient, the maximum duration of the study for each patient will be approximately 6 months (including up to 7 days for screening, 1 day of treatment and up to 6 months follow-up).
The study will close when all patients have completed the 6-Month Follow-up Assessment (Assessment V).
A safety follow-up will be performed 24 months after OP (Assessment VI).

9.11 Premature Termination of the Study

If the Investigator, the Sponsor, or the Monitor becomes aware of conditions or events that suggest a possible hazard to subjects if the study continues, the study may be terminated after appropriate consultation between the relevant parties. The study may also be terminated early at the Sponsor’s discretion in the absence of such a finding.
Conditions that may warrant termination include, but are not limited to:

- The discovery of an unexpected, significant, or unacceptable risk to the patients enrolled in the study;
- Failure to enroll patients at an acceptable rate;
- A decision on the part of the Sponsor to suspend or discontinue development of the drug.

9.12 Confidentiality

All study findings and documents will be regarded as confidential. The Investigator and members of his research team must not disclose such information without prior written approval from the Sponsor.

The anonymity of participating patients must be maintained. Patients will be identified on CRF submitted to the Data Base by their patient number, not by name. Documents not to be submitted to the Data Base that identify the patient (e.g., the signed informed consent) must be maintained in confidence by the Investigator.

9.13 Other Ethical and Regulatory Issues (Optional)

During the course of the study, the Investigator is obligated to submit to the IEC the following: amendments to the protocol, serious and unexpected AEs and their outcomes, specific site updates as agreed to by the Investigator and respective IEC, and any additional information (e.g., unexpected SAEs reported by other investigative sites, amendments to the Investigator Brochure, and administrative changes to the protocol) requested by the Sponsor to be provided to the IEC.

If a significant safety issue is identified, either from an ICSR or review of aggregate data, then the Sponsor will issue prompt notification to all parties – regulatory authorities, Investigators and IECs and PEI.

A significant safety issue is one that has a significant impact on the course of the clinical trial or program (including the potential for suspension of the trial program or amendments to protocols) or warrants immediate update of informed consent.

9.14 Liability and Insurance

The Insurance for study-related claims will be covered by Gothaer, Germany. The Sponsor will take out reasonable third-party liability insurance cover in accordance with all local legal requirements. The civil liability of the Investigator, the persons instructed by him and the hospital, practice or institute in which they are employed and the liability of the Sponsor with respect to financial loss due to personal injury and other damage that may arise as a result of the carrying out of this study are governed by the applicable law.

The Sponsor will arrange for patients participating in this study to be insured against financial loss due to personal injury caused by the pharmaceutical products being tested or by medical steps taken in the course of the study.

9.15 Publication Policy

The results of the clinical trial will be published after complete data collection and evaluation. Prof. G. Steinhoff, as the national coordinating Investigator and LKP of the trial, will be the senior author of the main publication. Trial site principal Investigators will be represented as authors of the publication according to the number of patients recruited and followed.

Partial or preliminary results may only be published beforehand with the approval of national coordinating Investigator (LKP) and the Sponsor . The publication is to be initiated by the national coordinating Investigator (LKP). The author is to consider the following persons as co-author:

- Trial Site Principal Investigators and Investigators (Prof. G. Steinhoff – last position, the other Investigators according to the number of patients recruited at trial site)
- The Statistician.

Any publication in the form of a lecture, poster of publication of data must basically be approved by the national coordinating Investigator (Prof. G. Steinhoff) and the Sponsor.

10 REFERENCE LIST

Asahara T, Murohara T, Sullivan A et al. Isolation of putative progenitor endothelial cells for angiogenesis. *Science*. 1997;275:964-967.

Asahara T, Masuda H, Takahashi T et al. Bone marrow origin of endothelial progenitor cells responsible for postnatal vasculogenesis in physiological and pathological neovascularization. Circ Res. 1999; 85(3):221-8.

Assmus B, Schachinger V, Teupe C, et al. Transplantation of Progenitor Cells and Regeneration Enhancement in Acute Myocardial Infarction (TOPCARE-AMI). Circulation. Dec 10 2002;106(24):3009-3017.

Assmus B, Honold J, Schächinger V, et al. Transcoronary transplantation of progenitor cells after myocardial infarction. NEJM 2006;355:1222-1232.

Balsam LB, Wagers AJ, Christensen JL, et al. Haematopoietic stem cells adopt mature haematopoietic fates in ischaemic myocardium. Nature. Apr 8 2004;428(6983):668-673.

Bauer P. and Köhne K.(1994). Evaluation of Experiments with Adaptive Interim Analyses. Biometrics 50, 1029-1041.

Beeres SL, Bax JJ, Kaandorp TA et al. Usefulness of intramyocardial injection of autologous bone marrow-derived mononuclear cells in patients with severe angina pectors and stress-induced myocardial ischemia. Am J Cardiol 2006;97(9):1326-31.

Beltrami AP, Urbanek K, Kajstura J et al. Evidence that human cardiac myocytes divide after myocardial infarction. *N Engl J Med*. 2001;344:1750-1757.

Bianchi DW, Johnson KL, Salem D. Chimerism of the transplanted heart. *N Engl J Med*. 2002;346:1410-1412.

Bittira B, Kuang JQ, Al Khaldi A et al. In vitro preprogramming of marrow stromal cells for myocardial regeneration. *Ann Thorac Surg*. 2002;74:1154-1159.

Camargo FD, Chambers SM, Goodell MA. Stem cell plasticity: from transdifferentiation to macrophage fusion. *Cell Proliferation*. 2004;37:55-65.

Chedrawy EG, Wang JS, Nguyen DM et al. Incorporation and integration of implanted myogenic and stem cells into native myocardial fibers: anatomic basis for functional improvements. *J Thorac Cardiovasc Surg*. 2002;124:584-590.

Chien KR. Stem cells: Lost in translation. *Nature*. 2004;428:607-608.

Couzin J, Vogel G. Cell therapy - Renovating the heart. *Science*. 2004;304:192-194.

Elashoff JD, nQuery Advisor®Release 5.0 User´s Guide. Los Angelos, CA; 2002

Epstein SE, Kornowski R, Fuchs S et al. Angiogenesis therapy - Amidst the hype, the neglected potential for serious side effects. *Circulation*. 2001;104:115-119.

Fernandez-Aviles F, San Roman JA, Garcia-Frade J, et al. Experimental and clinical regenerative capability of human bone marrow cells after myocardial infarction. Circ Res. Oct 1 2004;95(7):742-748.

Fuchs S, Satler LF, Kornowski R et al. Catheter-based autologous bone marrow myocardial injection in vooption patients with advanced cronary artery disease: a feasibility study. J Am Coll Cardiol 2003;41(10):1721-4.

Fuchs S, Kornowski R, Weisz G et al. Transendocardial autologous bone marrow cell transplantation in patients with advanced ischemic heart disease: Final results from a multi-center feasibility study. *Journal of the American College of Cardiology*. 2004;43:99A.

Galinanes M, Loubani M, Davies J et al. Autotransplantation of unmanipulated bone marrow into scarred myocardium is safe and enhances cardiac function in humans. *Cell Transplant*. 2004;13:7-13.

Gheorghiade M, Bonow RO. Chronic heart failure in the United States: a manifestation of coronary artery disease. Circulation. Jan 27 1998;97(3):282-289

Ghodsizad A, Klein HM, Borowski A, et al. Intraoperative isolation and processing of BM-derived stem Cells.Cytotherapy. 2004;6(5):523-6

Goodell MA, Rosenzweig M, Kim H et al. Dye efflux studies suggest that hematopoietic stem cells expressing low or undetectable levels of CD34 antigen exist in multiple species. *Nat Med*. 1997;3:1337-1345.

Goodell MA. Stem-cell "plasticity": befuddled by the muddle. *Current Opinion in Hematology*. 2003;10:208-213.

Hamano K, Nishida M, Hirata K et al. Local implantation of autologous bone marrow cells for therapeutic angiogenesis in patients with ischemic heart disease: clinical trial and preliminary results. *Jpn Circ J*. 2001;65:845-847.

Hamano K, Li TS, Kobayashi T, et al. Therapeutic angiogenesis induced by local autologous bone marrow cell implantation. Ann Thorac Surg. Apr 2002;73(4):1210-1215.

Honold J, Assmus B, Lehman R et al. Stem cell therapy of cardiac disease: an update. *Nephrology Dialysis Transplantation*. 2004;19:1673-1677.

Jackson KA, Majka SM, Wang H et al. Regeneration of ischemic cardiac muscle and vascular endothelium by adult stem cells. *J Clin Invest*. 2001;107:1395-1402.

Jiang Y, Vaessen B, Lenvik T, et al. Multipotent progenitor cells can be isolated from postnatal murine bone marrow, muscle, and brain. Exp Hematol 2002;30(8):896-904.

Kajstura J, Leri A, Finato N et al. Myocyte proliferation in end-stage cardiac failure in humans. *Proc Natl Acad Sci U S A*. 1998;95:8801-8805.

Kamihata H, Matsubara h, Nishiue T. et al. Impalntation of bone marrow mononuclear cells into ischemic myocardium enhances collateral perfusion and regional function via side supply of angioblasts, angiogenic ligands, and cytokines. Circulation 2001:28:104(9):1046-52.

Kamihata H, Matsubara H, Nishiue T et al. Improvement of collateral perfusion and regional function by implantation of peripheral blood mononuclear cells into ischemic hibernating myocardium. *Arterioscler Thromb Vasc Biol*. 2002;22:1804-1810.

Kinnaird T, Stabile E, Burnett MS et al. Bone marrow-derived cells for enhancing collateral development - Mechanisms, animal data, and initial clinical experiences. *Circulation Research*. 2004;95:354-363.

Kobayashi T, Hamano K, Li TS et al. Enhancement of angiogenesis by the implantation of self bone marrow cells in a rat ischemic heart model. *J Surg Res*. 2000;89:189-195.

Kocher AA, Schuster MD, Szabolcs MJ, et al. Neovascularization of ischemic myocardium by human bone-marrow-derived angioblasts prevents cardiomyocyte apoptosis, reduces remodeling and improves cardiac function. Nat Med. Apr 2001;7(4):430-436.

Korbling M, Estrov Z. Adult stem cells for tissue repair - A new therapeutic concept? *New England Journal of Medicine*. 2003;349:570-582.

Krause DS, Theise ND, Collector MI et al. Multi-organ, multi-lineage engraftment by a single bone marrow-derived stem cell. Cell 2001;105(3):369-77.

Laflamme MA, Myerson D, Saffitz JE et al. Evidence for cardiomyocyte repopulation by extracardiac progenitors in transplanted human hearts. *Circ Res*. 2002;90:634-640.

Leri A, Kajstura J, Anversa P. Cardiac stem cells and mechnaisms of myocardial regeneration. Physiol Rev 2005;85:1373-1416.

Lunde K, Solheim S, Aakhus S, et al. Intracoronary injection of mononuclear bone marrow cells in acute myocardial infarction. NEJM 2006;355:1199-1209.

Ma N, Ladilov Y, Moebius JM, On LL, Piechaczek C, David A, Kaminski A, Choi YH, Li W, Egger D, Stamm C, Steinhoff G. Intramyocardial delivery of human CD133^{+} cells in a SCID mouse cryoinjury model. Cardiov Res 2006; 71(1):158-169.

Makino S, Fukuda K, Miyoshi S et al. Cardiomyocytes can be generated from marrow stromal cells in vitro. J Clin Invest 1999;103(5):697-705.

Mathur A, Martin JF. Stem cells and repair of the heart. *Lancet*. 2004;364:183-192.

Menasche P, Hagege AA, Scorsin M et al. Myoblast transplantation for heart failure. Lancet 2001;347:279-280.

Muller P, Pfeiffer P, Koglin J et al. Cardiomyocytes of noncardiac origin in myocardial biopsies of human transplanted hearts. *Circulation*. 2002;106:31-35.

Murry CE, Soonpaa MH, Reinecke H, et al. Haematopoietic stem cells do not transdifferentiate into cardiac myocytes in myocardial infarcts. Nature. Apr 8 2004;428(6983):664-668.

Nasseri BA, Kukucka M, Dandek M, Ebell W, Hetzer R & Stamm C: Autologous CD133^{+} bone marrow cells and bypass grafting for regeneration of ischemic myocardium: results of the Cardio133 trial. J. Am. Coll. Cardiol. 2012; 59:E864

O´Brien RG, Muller KE. Unified Power Analysis for t-tests through Multivariate Hypotheses. In: Edwards LK (Ed.). Applied Analysis of Variance in Behavioral Science, Marcel Dekker, New York, 1993, Chapter 8 (pp 297-344).

Oakley RE, Msherqzi ZA, Lim SK et al. Transplantation of autologous bone marrow-derived cells into the myocardium of patients undergoing coronary bypass. Heart Surg Forum 2005;8(5):348-50.

Orlic D, Kajstura J, Chimenti S, et al. Bone marrow cells regenerate infarcted myocardium. Nature. Apr 5 2001;410(6829):701-705.

Orlic D, Kajstura J, Chimenti S et al. Transplanted adult bone marrow cells repair myocardial infarcts in mice. *Ann N Y Acad Sci*. 2001;938:221-229.

Orlic D, Kajstura J, Chimenti S et al. Mobilized bone marrow cells repair the infarcted heart, improving function and survival. *Proc Natl Acad Sci U S A*. 2001;98:10344-10349.

Parker JP, Danielsen B, Marcin J, Dai J, Mahendra G, Steimle AE. The California Report on Corornary Artery Bypass Graft Surgery 2003 Hospital Data www.oshpd.ca.gov/HQAD/Outcomes/Studies/cabg/2003Report/2003Report.pdf (November 2006).

Patel AN, Geffner L, Vina RF, Saslavsky J, Urschel HC, Kormos R, Benetti F. Surgical treatment for congestive heart failure with autologous adult stem cell transplantation: A prospective randomized study. J Thor Cardiov Surg 2005;130(6):1631-1638.

Peichev M, Naiyer AJ, Pereira D et al. Expression of VEGFR-2 and AC133 by circulating human CD34(+) cells identifies a population of functional endothelial precursors. *Blood*. 2000;95:952-958

Perin EC, Dohmann HF, Borojevic R et al. Transendocardial, autologous bone marrow cell transplantation for severe, chronic ischemic heart failure. *Circulation*. 2003;107:2294-2302.

Quaini F, Urbanek K, Beltrami AP et al. Chimerism of the transplanted heart. *N Engl J Med*. 2002;346:5-15.

Rafii S, Lyden D. Therapeutic stem and progenitor cell transplantation for organ vascularization and regeneration. *Nature Medicine*. 2003;9:702-712.

Risau W. Mechanisms of angiogenesis. *Nature*. 1997;386:671-674.

Rosenberger WF, Lachin JM. Randomization in Clinical Trials. New York: Wiley; 2002.

Schaechinger V, Assmus B, Britten MB, et al. Transplantation of progenitor cells and regeneration enhancement in acute myocardial infarction: final one-year results of the TOPCARE-AMI Trial. *J Am Coll Cardiol* 2004;**44**(8)**:**1690-9.

Schächinger V, Erbs S, Elsässer A, et al. Intracoronary bone marrow-derived progenitor cells in acute myocardial infarction. NEJM 2006;355:1201-1221.

Serruys PW, Morice MC, Kappetein AP, et al. Percutaneous coronary intervention versus coronary-artery bypass grafting for severe coronary artery disease. *N Engl J Med.* 2009 Mar 5;360(10):961-72.

Shake JG, Gruber PJ, Baumgartner WA et al. Mesenchymal stem cell implantation in a swine myocardial infarct model: engraftment and functional effects. Ann Thorac Surg 2002;73(6):1919-25.

Spangrude GJ, Torok-Storb B, Little MT. Chimerism of the transplanted heart. *N Engl J Med*. 2002;346:1410-1412.

Stamm C, Westphal B, Kleine HD, et al. Autologous bone-marrow stem-cell transplantation for myocardial regeneration. Lancet. Jan 4 2003;361(9351):45-46.

Stamm C, Kleine HD, Westphal B, et al. CABG and bone marrow stem cell transplantation after myocardial infarction. J Thorac Cardiovasc Surg 2004;52:152-158.

Stamm C, Kleine HD, Choi YH, et al. Intramyocardial delivery of $CD133^+$ bone marrow cells and coronary artery bypass grafting for chronic ischemic heart disease: Safety and efficacy studies. J Thorac Cardiovasc Surg 2007;133:717-25.

Strauer BE, Brehm M, Zeus T, et al. Repair of infarcted myocardium by autologous intracoronary mono-nuclear bone marrow cell transplantation in humans. Circulation. Oct 8 2002;106(15):1913-1918.

Steinhoff G:.Stammzelltherapie zur Regeneration von Herzmuskelgewebe. Internist 2006;47:479-488.

Takakura N, Watanabe T, Suenobu S, et al. A role for hematopoietic stem cells in promoting angiogenesis. Cell 2000;21(102(2):199-209.

Toma C, Pittenger Mf, Cahill KS et al. Human mesenchymal stem cells differentiate to a cardiomyocyte phenotype in the adult murine heart. Circulation 2002; 105(1):93-8.

Tomita S, Li RK, Weisel RD et al. Autologous transplantation of bone marrow cells improves damaged heart function. *Circulation*. 1999;100:II247-II256.

Tomita S, Mickle DA, Weisel RD et al. Improved heart function with myogenesis and angiogenesis after autologous porcine bone marrow stromal cell transplantation. J Thorac Cardiovasc Surg 2002;123(6):1132-40.

Tse HG, Kwong YL, Chan JK et al. Angiogenesis in ischaemic myocardium by intramyocardial autologous bone marrow mononuclear cell implnatation. Lancet 2003; 361(9351):47-9.

Tse HF, Thambar S, Kwong YL et al. Safety of catheter-based intramyocardial aulogous bone marrow cells implantation for therapeutic angiogenesis. Am J Cardiol. 2006; 98(1):60-2.

Urbich C, Dimmeler S. Endothelial progenitor cells: characterization and role in vascular biology. *Circ Res*. 2004;95:343-353.

Vaccarino V, Abramson JL, Veledar E, Weintraub WS. Sex Differences in Hospital Mortality After Coronary Artery Bypass Surgery: Evidence for a Higher Mortality in Younger Women. Circulation 2002; 105; 1176-1181.

Wassmer G, Eisebitt R. /ADDPLAN 2001: Adaptive Designs—Plans and Analyses/. Cologne, Germany: University

Wakitani S, Saito T, Caplan AI. Myogenic cells derived from rat bone marrow mesenchymal stem cells exposed to 5-azacytidine. *Muscle Nerve*. 1995;18:1417-1426.

Wang JS, Shum-Tim D, Galipeau J et al. Marrow stromal cells for cellular cardiomyoplasty: feasibility and potential clinical advantages. *J Thorac Cardiovasc Surg*. 2000;120:999-1005.

Wollert KC, Meyer GP, Lotz J, et al. Intracoronary autologous bone-marrow cell transfer after myocardial infarction: the BOOST randomised controlled clinical trial. Lancet. Jul 10-16 2004;364(9429):141-148.

Wollert KC, Drexler H. Clinical applications of stem cells for the heart. Circ Res. Feb 4 2005;96(2):151-163.

Wollert KC, Drexler H. Mesenchymal stem cells for myocardial infarction: promises and pitfalls. *Circulation* 2005;**112**(2)**:**151-3.

World Medical Association Declaration of Helsinki. Recommendations guiding physicians in biomedical research involving human subjects. Adopted by the 18th WMA General Assembly, Helsinki, Finland, June 1964, and amended by the 29th WMA General Assembly, Tokyo, Japan, October 1975 35th WMA General Assembly, Venice, Italy, October 1983 41st WMA General Assembly, Hong Kong, September 1989 48th WMA General Assembly, Somerset West, Republic of South Africa, October 1996.

11 APPENDICES

Appendix 1: Trial Flow Chart

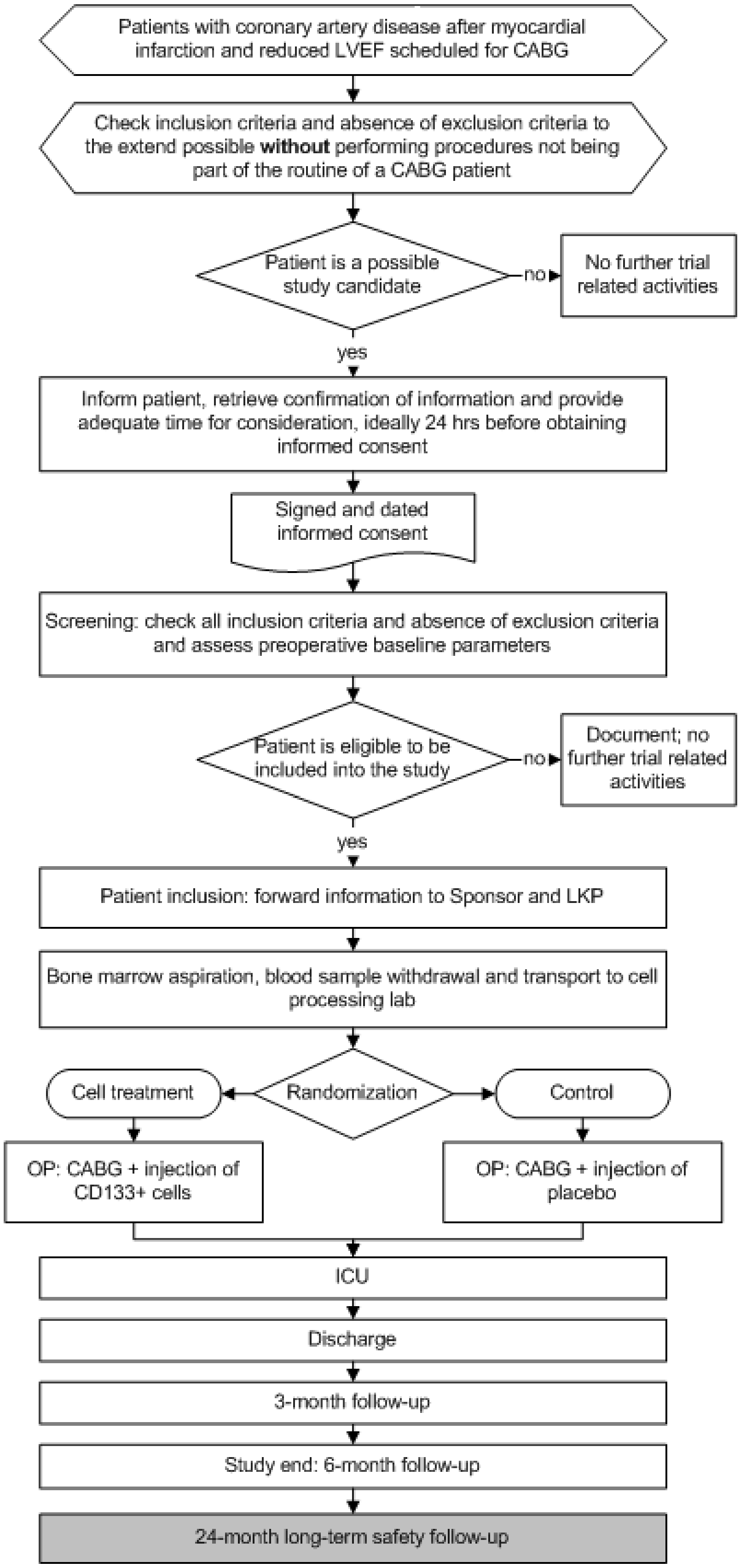

Appendix 2: Adverse Events of Specific Interest

Adverse event reporting						
	**0**	**I**	**II**	**III**	**IV**	**V**
	no event	asympto-matic	symptomatic, no specific treatment	symptomatic, treatment necessary	life-threate-ning	death
AV block<br>☐ I ☐ II ☐ III	☐	☐	☐	☐	☐	☐
Prolonged QT-Interval	☐	☐	☐	☐	☐	☐
Sinus bradycardia	☐	☐	☐	☐	☐	☐
Supraventricular arrhythmia	☐	☐	☐	☐	☐	☐
Ventricular arrhythmia	☐	☐	☐	☐	☐	☐
Vasovagal syncope	☐	☐	☐	☐	☐	☐
Left ventricular failure	☐	☐	☐	☐	☐	☐
Myocardial ischemia	☐	☐	☐	☐	☐	☐
Cerebral ischemia	☐	☐	☐	☐	☐	☐
Myocarditis	☐	☐	☐	☐	☐	☐
Pericardial Effusion, Pericarditis	☐	☐	☐	☐	☐	☐
Deep sternal wound infection	☐	☐	☐	☐	☐	☐

Appendix 3: Short Form Questionnaire (SF36)

Sehr geehrte Patientin, sehr geehrter Patient,

Sie haben sich für die Teilnahme an der Therapiestudie zum Einsatz körpereigener Stammzellen an unserer Klinik entschieden. Im Rahmen dieser Studie bitten wir Sie, die folgenden Fragen zu beantworten.

Die unten aufgelisteten Punkte beschreiben verschiedene Aspekte Ihres Alltags. Die Fragen dazu sollen darüber Aufschluss geben, wie sich Ihr derzeitiger Gesundheitszustand auf Ihre Lebensweise auswirkt.

1.	Wie würden Sie Ihren Gesundheitszustand im Allgemeinen beschreiben?
○	**Ausgezeichnet**
○	**Sehr gut**
○	**Gut**
○	**Weniger gut**
○	**Schlecht**

2.	Im Vergleich zum vergangenen Jahr, wie würden Sie Ihren derzeitigen Gesundheitszustand beschreiben?
○	**Derzeit viel besser**
○	**Derzeit etwas besser**
○	**Etwa wie vor einem Jahr**
○	**Derzeit etwas schlechter**
○	**Derzeit viel schlechter**

Im Folgenden sind einige Tätigkeiten beschrieben, die Sie vielleicht an einem normalen Tag ausüben.

3.	Sind Sie durch Ihren derzeitigen Gesundheitszustand bei diesen Tätigkeiten eingeschränkt? Wenn ja, wie stark?			
		**Ja, stark eingeschränkt**	**Ja, etwas eingeschränkt**	**Nein, überhaupt nicht eingeschränkt**
**3a.**	Anstrengende Tätigkeiten, z. B. schnell laufen, schwere Gegenstände heben, anstrengenden Sport treiben	○	○	○
**3b.**	Mittelschwere Tätigkeiten, z. B. einen Tisch verschieben, Staub saugen, kegeln, Golf spielen	○	○	○
**3c.**	Einkaufstaschen heben und tragen	○	○	○
**3d.**	**Mehrere** Treppenabsätze steigen	○	○	○

**3e.**	**Einen** Treppenabsatz steigen	○	○	○
**3f.**	Sich beugen, knien, bücken	○	○	○
**3g.**	**Mehr als 1 Kilometer** zu Fuß gehen	○	○	○
**3h.**	**Mehrere** Straßenkreuzungen weit zu Fuß gehen	○	○	○
**3i.**	**Eine** Straßenkreuzung weit zu Fuß gehen	○	○	○
**3j.**	Sich baden oder anziehen	○	○	○

4. Hatten Sie in den vergangenen 4 Wochen aufgrund Ihrer körperlichen Gesundheit irgendwelche Schwierigkeiten bei der Arbeit oder anderen alltäglichen Tätigkeiten im Beruf bzw. zu Hause?

		Ja	Nein
**4a.**	Ich konnte nicht **so lange** wie üblich tätig sein.	○	○
**4b.**	Ich habe **weniger geschafft** als ich wollte.	○	○
**4c.**	Ich konnte **nur bestimmte** Dinge tun.	○	○
**4d.**	Ich hatte **Schwierigkeiten** bei der Ausführung.	○	○

5. Hatten Sie in den vergangenen 4 Wochen aufgrund seelischer Probleme irgendwelche Schwierigkeiten bei der Arbeit oder anderen alltäglichen Tätigkeiten im Beruf bzw. zu Hause (z. B. weil Sie sich niedergeschlagen oder ängstlich fühlten)?

		Ja	Nein
**5a.**	Ich konnte nicht **so lange** wie üblich tätig sein.	○	○
**5b.**	Ich habe **weniger geschafft** als ich wollte.	○	○
**5c.**	Ich konnte nicht so **sorgfältig** wie üblich arbeiten.	○	○

6. Wie sehr haben Ihre körperliche Gesundheit oder seelische Probleme in den vergangenen 4 Wochen Ihre normalen Kontakte zu Familienangehörigen, Freunden, Nachbarn oder zum Bekanntenkreis beeinträchtigt?

○ **Überhaupt nicht**

○ **Etwas**

○ **Mäßig**

○ **Ziemlich**

○ **Sehr**

7. Wie stark waren Ihre Schmerzen in den vergangenen 4 Wochen?

○ **Keine Schmerzen**

○	**Sehr leicht**
○	**Leicht**
○	**Mäßig**
○	**Stark**
○	**Sehr stark**

**8.**	**Inwieweit haben die Schmerzen Sie in den vergangenen 4 Wochen bei der Ausübung Ihrer Alltagstätigkeiten zu Hause und im Beruf behindert?**
○	**Überhaupt nicht**
○	**Ein Bisschen**
○	**Mäßig**
○	**Ziemlich**
○	**Sehr**

In diesen Fragen geht es darum, wie Sie sich fühlen und wie es Ihnen in den vergangenen 4 Wochen gegangen ist. (Bitte kreuzen Sie in jeder Zeile das Kästchen an, das Ihrem Befinden am ehesten entspricht).

**9.**	**Wie oft waren Sie in den vergangenen 4 Wochen**	Immer	Meistens	Ziemlich oft	Manch-mal	Selten	Nie
**9a.**	**… voller Schwung?**	○	○	○	○	○	○
**9b.**	**… sehr nervös?**	○	○	○	○	○	○
**9c.**	**… so niedergeschlagen, dass Sie nichts aufheitern konnte?**	○	○	○	○	○	○
**9d.**	**… ruhig und gelassen?**	○	○	○	○	○	○
**9e.**	**… voller Energie?**	○	○	○	○	○	○
**9f.**	**… entmutigt und traurig?**	○	○	○	○	○	○
**9g.**	**… erschöpft?**	○	○	○	○	○	○
**9h.**	**… glücklich?**	○	○	○	○	○	○
**9i.**	**… müde?**	○	○	○	○	○	○

**10.**	**Wie häufig haben Ihre körperliche Gesundheit oder seelische Probleme in den vergangenen 4 Wochen Ihre Kontakte zu anderen Menschen (Besuche bei Freunden, Verwandten usw.) beeinträchtigt?**
○	**Immer**
○	**Meistens**

○	**Manchmal**
○	**Selten**
○	**Nie**

11. Inwieweit trifft jede der folgenden Aussagen auf Sie zu?

		Trifft ganz zu	Trifft weit-gehend zu	Weiß nicht	Trifft weit-gehend nicht zu	Trifft überhaupt nicht zu
**11a.**	**Ich scheine etwas leichter als andere krank zu werden.**	○	○	○	○	○
**11b.**	**Ich bin genauso gesund wie alle anderen, die ich kenne.**	○	○	○	○	○
**11c.**	**Ich erwarte, dass meine Gesundheit nachlässt.**	○	○	○	○	○
**11d.**	**Ich erfreue mich ausgezeichneter Gesundheit.**	○	○	○	○	○

Appendix 4: Minnesota Living with Heart Failure Questionnaire (MLHF)

Sehr geehrte Patientin, sehr geehrter Patient,

Sie haben sich für die Teilnahme an der Therapiestudie zum Einsatz körpereigener Stammzellen an unserer Klinik entschieden. Im Rahmen dieser Studie bitten wir Sie, die folgenden Fragen zu beantworten.

Diese Fragen sollen darüber Aufschluss geben, wie Ihre Herzinsuffizienz Sie im vergangenen Monat an der von Ihnen gewünschten Lebensweise gehindert hat. Die unten aufgelisteten Punkte beschreiben verschiedene Arten von Beeinträchtigungen. Wenn Sie sicher sind, dass ein Punkt nicht auf Sie zutrifft oder in keinem Zusammenhang mit ihrer Herzinsuffizienz steht, kreuzen Sie '0' ("Nein") an und beantworten Sie dann die nächste Frage. Wenn ein Punkt Sie betrifft, kreuzen Sie die Zahl an, die widerspiegelt, wie stark Sie an der von Ihnen gewünschten Lebensweise gehindert wurden.
Hat Ihre Herzinsuffizienz Sie im vergangenen Monat an der von Ihnen gewünschten Lebensweise gehindert, dadurch dass . . . . .

	Nein	Sehr wenig		Mittel		Sehr stark
1. Schwellungen Ihrer Knöchel, Beine etc. auftraten?	0	1	2	3	4	5
2. Sie sich tagsüber hinlegen oder hinsetzen mussten, um sich auszuruhen?	0	1	2	3	4	5
3. Sie beim Gehen oder Treppensteigen Schwierigkeiten hatten?	0	1	2	3	4	5
4. Sie bei der Haus- oder Gartenarbeit Schwierigkeiten hatten?	0	1	2	3	4	5
5. Sie Schwierigkeiten hatten, außer Haus zu gehen?	0	1	2	3	4	5
6. Sie Schwierigkeiten hatten, nachts gut zu schlafen?	0	1	2	3	4	5
7. Sie Schwierigkeiten hatten, mit Familie oder Freunden Kontakte zu pflegen oder gemeinsame Unternehmungen durchzuführen?	0	1	2	3	4	5
8. Sie Schwierigkeiten hatten, Ihren Lebensunterhalt zu verdienen?	0	1	2	3	4	5
9. Sie bei Freizeitbeschäftigungen, Sport oder Hobbys Schwierigkeiten hatten?	0	1	2	3	4	5
10. Sie in Ihrem Sexualleben beeinträchtigt waren?	0	1	2	3	4	5
11. Sie weniger von dem essen konnten, was Sie mögen?	0	1	2	3	4	5
12. Sie unter Kurzatmigkeit litten?	0	1	2	3	4	5
13. Sie müde, erschöpft oder energielos waren?	0	1	2	3	4	5
14. Sie im Krankenhaus bleiben mussten?	0	1	2	3	4	5
15. Sie Geld für Ihre medizinische Versorgung bezahlen mussten?	0	1	2	3	4	5
16. Sie unter Nebenwirkungen Ihrer Medikamente litten?	0	1	2	3	4	5
17. Sie sich als Belastung für Ihre Familie oder Freunde empfanden?	0	1	2	3	4	5
18. Sie das Gefühl hatten, weniger Kontrolle über Ihr Leben zu haben?	0	1	2	3	4	5
19. Sie sich Sorgen machten?	0	1	2	3	4	5

20. Sie Schwierigkeiten hatten, sich zu konzentrieren oder sich an etwas zu erinnern?	0	1	2	3	4	5
21. Sie sich deprimiert fühlten?	0	1	2	3	4	5

Copyright University of Minnesota 1986

Appendix 5: Questionnaire of the EuroQoL group: EQ-5D™

Bitte geben Sie an, welche Aussagen Ihren heutigen Gesundheitszustand am besten beschreiben, indem Sie ein Kreuz in ein Kästchen jeder Gruppe machen.

Beweglichkeit/Mobilität

Ich habe keine Probleme herumzugehen ☐

Ich habe einige Probleme herumzugehen ☐

Ich bin ans Bett gebunden ☐

Für sich selbst sorgen

Ich habe keine Probleme, für mich selbst zu sorgen ☐

Ich habe einige Probleme, mich selbst zu waschen oder mich anzuziehen ☐

Ich bin nicht in der Lage, mich selbst zu waschen oder anzuziehen ☐

Allgemeine Tätigkeiten (z.B. Arbeit, Studium, Hausarbeit, Familien- oder Freizeitaktivitäten)

Ich habe keine Probleme, meinen alltäglichen Tätigkeiten nachzukommen ☐

Ich habe einige Probleme meinen alltäglichen Tätigkeiten nachzukommen ☐

Ich bin nicht in der Lage meinen alltäglichen Tätigkeiten nachzukommen ☐

Schmerzen/Körperliche Beschwerden

Ich habe keine Schmerzen oder Beschwerden ☐

Ich habe mäßige Schmerzen oder Beschwerden ☐

Ich habe extreme Schmerzen oder Beschwerden ☐

Angst/Niedergeschlagenheit

Ich bin nicht ängstlich oder deprimiert ☐

Ich bin mäßig ängstlich oder deprimiert ☐

Ich bin extrem ängstlich oder deprimiert ☐

EQ Visual Analog Scale (VAS)

Bester denkbarer Gesundheitszustand

Ihr heutiger Gesundheitszustand?

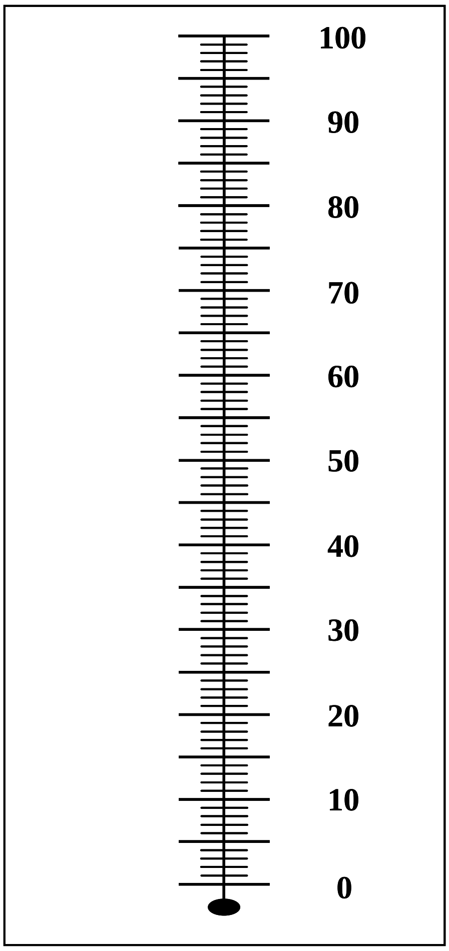

Schlechtest denkbarer Gesundheitszustand
