# Supplementary material for: Hematopoietic stem-cell senescence and myocardial repair - Coronary artery disease genotype/phenotype analysis of post-MI myocardial regeneration response induced by CABG/CD133+ bone marrow hematopoietic stem cell treatment in RCT PERFECT Phase 3
Source: eBioMedicine. 2020 Jul 4;57:102862. doi: 10.1016/j.ebiom.2020.102862 (PMC7339012; doi:10.1016/j.ebiom.2020.102862)
Supplement: Supplementary file 12 [file mmc12.pdf]

CLINICAL STUDY REPORT OF PERFECT 001

Intramyocardial transplantation of bone marrow stem cells for improvement of post-infarct myocardial regeneration in addition to CABG surgery: a controlled, prospective, randomized, double blinded multicentre trial (PERFECT trial)

Development Phase:	III
Name of Investigational Drug:	$CD133^{+}$ autologous bone marrow stem cells
Sponsor:	Miltenyi Biotec GmbH<br>Friedrich-Ebert-Straße 68<br>51429 Bergisch Gladbach<br>Germany
Sponsor Study Number:	PERFECT 001 (M-2006-144)
IND/NDA number:	
EudraCT number:	2006-006404-11 (DRKS, Number: DRKS00000213)
Date of first visit:	20 August 2009
Date of last visit:	10 March 2016
Date of database snapshot:	15 July 2016
Principal Investigator:	Professor Dr. med. Gustav Steinhoff<br>Universität Rostock<br>Direktor Klinik und Poliklinik für Herzchirurgie,<br>Schillingallee 35<br>D-18057 Rostock<br>Phone: +49 (381) 494-6100<br>E-mail: gustav.steinhoff@med.uni-rostock.de
Author(s):	Claudia Frumento PhD, Medical Writer<br>Professor Dr. Günther Kundt, Statistician<br>Uta Mehdorn, Statistician
Version identification:	Final 1.0 - 08 March 2017

The study was conducted in accordance with the Helsinki Declaration and Good Clinical Practice. Independent Ethics Committee approval and written informed consent were obtained before starting the study.

The information contained herein is the property Miltenyi Biotec GmbH and may not be reproduced, published or disclosed to others without written authorisation of Miltenyi Biotec GmbH

SIGNATURE PAGE [SPONSOR]

CLINICAL STUDY

REPORT OF STUDY: PERFECT 001 (M-2006-144)

STUDY TITLE: Intramyocardial transplantation of bone marrow stem cells for improvement of post-infarct myocardial regeneration in addition to CABG surgery: a controlled, prospective, randomized, double blinded multicentre trial (PERFECT trial)

AUTHORS: Claudia Frumento PhD

Professor Dr. Günther Kundt, Statistician

Uta Mehdorn, Statistician

MEDICAL WRITER	STATISTICIAN	MEDICAL DIRECTOR
Claudia Frumento PhD	Prof. Dr. -Ing. Günther Kundt	Dr. med. Liane Preußner
Teltowerstr. 35<br>14109 Berlin, Germany	Universität Rostock<br>Institut für Medizinische Informatik und Biometrie<br>Rembrandtstr. 16/17<br>18057 Rostock, Germany	Miltenyi Biotec GmbH<br>Friedrich-Ebert-Str. 68<br>51429 Bergisch-Gladbach, Germany
Signature:<br><br>Date:	Signature:<br><br>Date:	Signature:<br><br>Date:

APPROVAL PAGE OF THE PRINCIPAL INVESTIGATOR

CLINICAL STUDY

REPORT OF STUDY: PERFECT 001 (M-2006-144)

STUDY TITLE: Intramyocardial transplantation of bone marrow stem cells for improvement of post-infarct myocardial regeneration in addition to CABG surgery: a controlled, prospective, randomized, double blinded multicentre trial (PERFECT trial)

AUTHORS: Claudia Frumento PhD

Professor Dr. Günther Kundt, Statistician

Uta Mehdorn, Statistician

I have read this report and confirm that to the best of my knowledge it accurately describes the conduct and results of the study.

Professor Dr. med. Gustav Steinhoff
Universität Rostock
Direktor Klinik und Poliklinik für Herzchirurgie,
Schillingallee 35
D-18057 Rostock

Signature:

Date:

SYNOPSIS

NAME OF SPONSOR<br>Miltenyi Biotec GmbH	INDIVIDUAL STUDY TABLE<br>REFERRING TO PART<br>OF THE DOSSIER<br>Volume:<br>Page:	(FOR NATIONAL AUTHORITY USE ONLY)
NAME OF FINISHED PRODUCT<br>CD133+ autologous bone marrow derived cells IMPD 1.1.1		
NAME OF ACTIVE INGREDIENT<br>CD133+ autologous bone marrow derived cells		

TITLE OF STUDY

Intramyocardial transplantation of bone marrow stem cells for improvement of post-infarct myocardial regeneration in addition to Coronary Artery Bypass Graft (CABG) surgery: a controlled, prospective, randomized, double blinded multicentre trial (PERFECT trial)

COORDINATING AND PRINCIPAL INVESTIGATOR

Prof. Dr. med. Gustav Steinhoff
Universität Rostock
Direktor Klinik und Poliklinik für Herzchirurgie, Schillingallee 35

PRINCIPAL INVESTIGATORS

Prof. Dr. med. Christof Stamm
Deutsches Herzzentrum Berlin

Prof. Dr. med. Dr. h. c. Axel Haverich
Medizinische Hochschule Hannover

Prof. Dr. med. Gustav Steinhoff
Universität Rostock

Dr. med. Jochen Börgermann
Herz- und Diabeteszentrum Nordrhein Westfalen

Prof. Dr. med. Friedrich-Wilhelm Mohr
Klinik für Herzchirurgie, Herzzentrum Universität Leipzig

PD. Dr. med. Florian Mathias Wagner
Klinik und Poliklinik für Herz- und Gefäßchirurgie
Universitäres Herzzentrum Hamburg

STUDY CENTERS

Deutsches Herzzentrum Berlin
Klinik für Herz-, Thorax- und Gefäßchirurgie
Augustenburger Platz 1
D-13353 Berlin
Phone: +49 (30) 4593- 2109
Fax: +49 (30) 4593-2100
E-mail: stamm@DHZB.de

Medizinische Hochschule Hannover
Klinik für Thorax-, Herz- und Gefäßchirurgie
Carl-Neuberg Strasse 1
D-30625 Hannover
Phone: +49 (511) 532 65 80

**NAME OF SPONSOR**<br>Miltenyi Biotec GmbH	**INDIVIDUAL STUDY TABLE**<br>**REFERRING TO PART**<br>**OF THE DOSSIER**<br>Volume:<br>Page:	**(FOR NATIONAL AUTHORITY USE ONLY)**
**NAME OF FINISHED PRODUCT**<br>CD133+ autologous bone marrow derived cells IMPD 1.1.1		
**NAME OF ACTIVE INGREDIENT**<br>CD133+ autologous bone marrow derived cells		

Fax: +49 (511) 532 5404
E-mail: haverich.axel@mh-hannover.de

Universität Rostock
Klinik und Poliklinik für Herzchirurgie
Schillingallee 35
D-18057 Rostock
Phone: +49 (381) 494-6100
Fax: +49 (381) 494-6102
E-mail: gustav.steinhoff@med.uni-rostock.de

Herz- und Diabeteszentrum Nordrhein Westfalen
Universitätsklinik der Ruhruniversität Bochum Georgstraße 11
32545 Bad Oeynhausen
Phone: +49 (5731) 97 32 47
Fax: +49 (5731) 97 1820
e-mail: jboergermann@hdz-nrw.de

Klinik für Herzchirurgie
Herzzentrum Universität Leipzig
Strümpellstraße 39
04289 Leipzig
Phone: +49(341)865-1421
Fax: +49(3 41)865-1452
e-mail: chir@herzzentrum-leipzig.de

Klinik und Poliklinik für Herz- und Gefäßchirurgie
Universitäres Herzzentrum Hamburg
Martinistr. 52
20246 Hamburg
Phone: +49 (40) 7410-58949
Fax: +49 (40) 7410-57926
e-mail: fl.wagner@uke.de

PUBLICATION (REFERENCE)

Validating intramyocardial bone marrow stem cell therapy in combination with coronary artery bypass grafting, the PERFECT Phase III randomized multicentre trial: study protocol for a randomized controlled trial. Donndorf P, Kaminski A, Tiedemann G, Kundt G, Steinhoff G. 2012 Jul 2;13:99. doi: 10.1186/1745-6215-13-99.

**STUDY PERIOD**	**PHASE OF DEVELOPMENT**
Screening date of first patient in: 20 August 2009<br>Date of last patient completed: 10 March 2016<br>The study was prematurely terminated due to very slow patient recruitment	Phase III

NAME OF SPONSOR<br>Miltenyi Biotec GmbH	INDIVIDUAL STUDY TABLE<br>REFERRING TO PART<br>OF THE DOSSIER<br>Volume:<br>Page:	(FOR NATIONAL AUTHORITY USE ONLY)
NAME OF FINISHED PRODUCT<br>CD133+ autologous bone marrow derived cells IMPD 1.1.1		
NAME OF ACTIVE INGREDIENT<br>CD133+ autologous bone marrow derived cells		

OBJECTIVES

Primary objective

Determine whether injection of autologously derived bone marrow stem cells yielded a functional benefit in addition to the coronary artery bypass graft (CABG) operation as determined by left ventricular heart function (left ventricular ejection fraction [LVEF] determined with magnetic resonance imaging [MRI]).

Secondary Objectives

Determine the effects of an injection of autologously-derived bone marrow stem cells on physical exercise capacity, cardiac function, safety and Quality of Life (QoL).

METHODOLOGY

Controlled, prospective, randomized, double blinded multicentre trial

NUMBER OF PATIENTS (PLANNED AND ANALYSED)

Analysed for safety: The safety population comprised all patients randomized into the study and treated. Safety evaluations were performed on the safety population (SAS). All comparisons were executed per group, to which the patients were randomized.

Analysed for efficacy: The “Full Analysis Set” (FAS) following the principle of intent-to-treat (ITT) included every patient randomized and compare the patients per group to which they were randomly allocated, regardless of patients' compliance, or withdrawal from the study. Confirmatory analyses on primary efficacy end-point was to be performed on the full analysis set (FAS) patients. This intention to treat (ITT) analysis was to be considered as the primary one.

The “Per Protocol Set” (PPS) was defined as a subset of the FAS/ITT patients who were compliant with the study protocol. The PPS consisted of all patients from the FAS/ITT group without any major protocol violation. A secondary efficacy analysis of the primary endpoint was performed based upon the PPS, to assess the sensitivity of the analysis to the choice of analysis population.

Planned number of patients: 142

Screened number of patients: 119

Randomized number of patients: 82

MAIN CRITERIA FOR INCLUSION

Inclusion criteria

- Coronary artery disease after myocardial infarction with indication for CABG surgery
- Currently reduced global LVEF assessed at site by cardiac MRI at rest (25% ≤ LVEF ≤ 50%)
- Presence of a localized akinetic/hypokinetic/hypoperfused area of LV myocardium for defining the target area
- Informed consent of the patient
- 18 years ≤ Age < 80 years
- Not pregnant and not planning to become pregnant during the study. Females with childbearing potential had to provide a negative pregnancy test within 1-7 days before OP and had to be using oral or injectable contraception (non-childbearing potential is defined as post-menopausal for at least 1 year or surgical sterilization or hysterectomy at least 3 months before study start).

NAME OF SPONSOR<br>Miltenyi Biotec GmbH	INDIVIDUAL STUDY TABLE<br>REFERRING TO PART<br>OF THE DOSSIER<br>Volume:<br>Page:	(FOR NATIONAL AUTHORITY USE ONLY)
NAME OF FINISHED PRODUCT<br>CD133+ autologous bone marrow derived cells IMPD 1.1.1		
NAME OF ACTIVE INGREDIENT<br>CD133+ autologous bone marrow derived cells		

Exclusion criteria

- Emergency operation
- Presence of any moderate-severe valve heart disease requiring concomitant valve replacement or reconstruction
- Medical History of recent resuscitation in combination with ventricular arrhythmia classified by LOWN ≥ class II
- Acute myocardial infarction within last 2 weeks
- Debilitating other disease: Degenerative neurologic disorders, psychiatric disease, terminal renal failure requiring dialysis, previous organ transplantation, active malignant neoplasia, or any other serious medical condition that, in the opinion of the Investigator is likely to alter the patient’s course of recovery or the evaluation of the study medication’s safety
- Impaired ability to comprehend the study information
- Absence of written informed consent
- Treatment with any investigational drug within the previous 30 days
- Apparent infection (c-reactive protein [CRP] ≥ 20 mg/L, fever ≥ 38.5° C)
- Contraindication for MRI scan
- Immune compromise including Anti human immunodeficiency virus (HIV) 1/2, HBsAg, Anti-HBc-IgG, Anti hepatitis C virus (HCV), Treponema pallidum
- Pregnant or breast feeding
- Childbearing potential with unreliable birth control methods
- Have previously been enrolled in this study, respectively phase I and phase II
- Known hypersensitivity or sensitization against murine products and human-anti-mouse-antibody-titer ≥ 1:1000
- Contraindication to bone marrow aspiration
- Known hypersensitivity against iron dextran

INVESTIGATIONAL DRUG, DOSE AND MODE OF ADMINISTRATION, BATCH NUMBER

0.5-5x10^6 CD133+ in 5 mL saline and serum suspension, injected intramyocardially during CABG surgery.

The 5 mL suspension were distributed in 15 individual 1 mL syringes (26 Gauge needle) of 0.3 mL aliquots (in total 5 mL, including up to 0.5 mL rest in syringes) and were applied within 3 minutes in the region of interest (infarction border zone) at the end of bypass surgery. No more than one injection per square centimetre could be injected.

REFERENCE DRUG, DOSE AND MODE OF ADMINISTRATION, BATCH NUMBER

5 mL saline plus serum solution, injected intramyocardially during CABG surgery.

The 5 mL suspension were distributed in 15 individual 1 mL syringes (26 Gauge needle) of 0.3 mL aliquots (in total 5 mL, including up to 0.5 mL rest in syringes) ad were applied within 3 minutes in the region of interest (infarction border zone) at the end of bypass surgery. No more than one injection per square centimetre could be injected.

NAME OF SPONSOR<br>Miltenyi Biotec GmbH	INDIVIDUAL STUDY TABLE<br>REFERRING TO PART<br>OF THE DOSSIER<br>Volume:<br>Page:	(FOR NATIONAL AUTHORITY USE ONLY)
NAME OF FINISHED PRODUCT<br>CD133+ autologous bone marrow derived cells IMPD 1.1.1		
NAME OF ACTIVE INGREDIENT<br>CD133+ autologous bone marrow derived cells		

DURATION OF TREATMENT

Not defined

CRITERIA FOR EVALUATION – SAFETY ENDPOINTS

- Major adverse cardiovascular events (MACE - cardiac death, myocardial infarction, secondary intervention/reoperation, ventricular arrhythmia).
- Adverse Events of Specific Interest (AESI): AV-block (I, II or III), prolonged QT interval, sinus bradycardia, supraventricular arrhythmia, ventricular arrhythmia, vasovagal syncope, left ventricular failure, myocardial ischemia, cerebral ischemia, myocarditis, pericardial Effusion, pericarditis, deep sternal wound infection (or wound infection at the site of graft sampling) coded as "deep postoperative wound infection" (Meddra LLT 10074392).
- Serious Adverse Events (SAEs)and Adverse Events (AEs).

CRITERIA FOR EVALUATION – EFFICACY ENDPOINTS

Primary endpoint: LVEF at 6 months postoperatively, measured by MRI at rest and change in LVEF at 6 months post-OP compared with preoperatively (screening) and early postoperatively (discharge) as assessed by MRI. Cardiac MRI was established as the gold standard for determination of LV function (LVEF and LV volumes).

Secondary endpoints:

- Change in LVEF at 6 months post-OP compared with preoperatively (screening) and early postoperatively (discharge) as assessed by echocardiography.
- Change in LV dimensions (left ventricular end systolic dimension [LVESD], [LVEDD]) at 6-month post-OP compared with preoperatively (screening) and early postoperatively (discharge) as assessed by echocardiography.
- Change in physical exercise capacity determined by 6-minute walk test at 6 months post-OP compared with preoperatively (screening) and early postoperatively (discharge).
- Change in New York Heart Association (NYHA) and CCS class at 6 months post-OP compared with preoperatively (screening) and early postoperatively (discharge).
- MACE (cardiac death, myocardial infarction, secondary intervention/reoperation, ventricular arrhythmia).
- QoL-score at 6 months post-OP compared with preoperatively (screening) and 3 months (telephone).

STATISTICAL METHODS

Interim Analysis (IA)

An interim analysis was performed on the first 70 patients randomized, and followed-up for at least 6 months.

Main Analysis (MA)

When all patients included into the trial (planned: 142) had had their 6 Month Follow-up visit the main analysis was to be performed.

In case of stopping for futility patient recruitment was to be stopped. All patients included so far were to be followed up for safety evaluation as foreseen by the protocol. All data analysis as foreseen in MA except the

NAME OF SPONSOR<br>Miltenyi Biotec GmbH	INDIVIDUAL STUDY TABLE<br>REFERRING TO PART<br>OF THE DOSSIER<br>Volume:<br>Page:	(FOR NATIONAL AUTHORITY USE ONLY)
NAME OF FINISHED PRODUCT<br>CD133+ autologous bone marrow derived cells IMPD 1.1.1		
NAME OF ACTIVE INGREDIENT<br>CD133+ autologous bone marrow derived cells		

confirmatory statistical test were to be performed based on the data of those patients enrolled so far at the time point when all patients enrolled so far were followed for at least 6 months.

Safety-Follow-up-Analysis (SFUA)

A Safety-Follow-up-Analysis was to be performed at the time point when the last patient was followed up for additional 18 months and her/his 24-month visit was performed and data are included into the database.

In case of stopping the trial the SFUA was to be performed when all patients enrolled so far had their 24-month safety follow-up visit.

Sample Size

Sample size determination was done under the assumption of a two-sided type I error (α) at 5% and a type II error (β) at 10% (i.e. a power at 90%).The scenario of a difference in LVEF at month 6 post- operatively between the two treatment arms of about 4 to 5 was considered as a clinical relevant difference. With a difference of 4.5 and a standard deviation of 7.5, at least n=60 patients per group were considered necessary and with an additional 15% drop-out rate a total of at least 142 patients were to be randomized.

SAFETY RESULTS

Summary of Adverse Events

- The occurrence rate of MACE was very low when compared with the occurrence reported in the literature for patients undergoing CABG surgery (26.9%). With a p-value of 1.000, there was no difference in the occurrence of MACE between the two groups of patients (1 MACE in the placebo group and 1 MACE in the CD133+ group).
- In the AESIs analysis no statistically significant differences could be observed between both treatment groups indicating that the AESIs were related either with the CABG surgery or the underlying disease (67 AESIs in the placebo group and 68 AESIs in the treatment group);
- During the main trial phase, there were 49 SAEs, 25 (15 patients) in the placebo group and 24 (19 patients) in the CD133+ group. There were no statistical differences observed between the placebo and the CD 133+ group neither overall nor in any of the system organ classes. The most common SAEs were cardiac disorders followed by infections and infestations and respiratory, thoracic and mediastinal disorders. There were no SAEs considered related with the treatment in the CD133+ group.
- In total, there were 619 AEs during the study. Twenty-six AEs during the screening phase and 593 AEs during the main trial phase. There were no statistical differences between the placebo and the CD133+ group, neither overall nor in any of the categories in which the patients or AEs were classified.
- All patients experienced at least one AE during the main trial phase. Overall, there were 19 AEs and two SAEs that were at least possibly related. There were no deaths during the screening nor the main trial phase.

EFFICACY RESULTS

Primary Efficacy

With p-values of 0,8130 and 0,4454 for Visit III and Visit V respectively in the FAS/ITT and p-values of 0,6771 and 0,4261 for Visit III and Visit V respectively in the PPS, the difference between the treatments groups is not statistically significant. The probability of not having detected a possible positive effect of the therapy (injection of CD133+ in the myocardium) on the improvement of LVEF is very small.

Though there were no statistical tests performed with the following endpoints, the following was observed:

- Unadjusted values of LVEF measured by MRI revealed a larger increase in the patients treated with CD133+. In the FS/ITT, baseline LVEF for the placebo group was 35,1 compared to 42.5 at Visit V and for the treatment group it was 32.7 at baseline versus 44.1 at Visit V.
- Mean values of scar tissue in the FAS/ITT measured by MRI were less in the CD133+ group than in the placebo group (27.0 versus 37.3) and in the PPS were also less in the CD133+ group than in the placebo group (27.9 versus 34.8).
- Mean values of non-viable tissue in the FAS/ITT measured by MRI were less in the CD133+ group than in the placebo group (20.0 versus 30.2) and in the PPS were also less in the CD133+ group than in the placebo group (20.7 versus 28.0).

Secondary Efficacy

The secondary efficacy analysis was only a descriptive analysis of the variables and no tests were done regarding possible differences between the placebo and the active treatment group. The following was observed:

- The poor quality of the echocardiographies did not allow any conclusions nor a comparison with the MRI results. Since the two previous studies used the LVEF measured with echocardiography as a primary endpoint, it was decided to use this same method for the secondary endpoints to be able to compare with the previous studies.
- The 6MWT showed the following changes (Visit V-Visit 1) in the mean values: in the FAS/ITT (49.3 for the placebo group and 59.4 in the treatment group. In the PPS, these changes were 50.7 for the placebo group and 56.1 in the treatment group.
- Minimal changes in NYHA class and CCS were observed after surgery plus placebo or active treatment. Mean difference of the NYHA in the placebo and treatment group was -0.7 in the FAS/ITT and in the PPS the mean differences were -0.7 in the placebo group and -0.8 in the treatment group. Mean difference of the CCS in the placebo group was -1.4 in the treatment group was -1.0 in the FAS/ITT and in the PPS the mean differences were -1.4 in the placebo group and -0.9 in the treatment group.
- The occurrence rate of MACE was very low when compared with the occurrence reported in the literature for patients undergoing CABG surgery (26.9%). With a p-value of 1, there was no difference in the occurrence of MACE between the two groups of patients (1 MACE in the placebo group and 1 MACE in the CD133+ group). There were no changes in the EQ-5D - mobility index
- The EQ-5D VAS showed some changes in the mean value: in the FAS/ITT (6.1 for the placebo group and 11.1 in the treatment group. In the PPS, these changes were 4.4 for the placebo group and 16.4 in the treatment group. It should be noted that the increase of the index indicates an improvement of the condition.
- The MLHF-Q total score showed changes in the mean value: in the FAS/ITT (-14.7 for the placebo group and -8.6 in the treatment group. In the PPS, these changes were -16.1 for the placebo group and -10.1 in the treatment group. It should be noted that a negative change in the index indicates an improvement of the condition.

CONCLUSIONS

The procedure was demonstrated to be safe, showing a low incidence of SAEs and MACEs when compared to the SAEs and MACEs in other trials.

Overall, the LVEF increase in the total population (placebo and treatment group) was clinically significant (FAS/ITT: 9,5%; PPS: 9,6%), however the study could not demonstrate a positive effect of the CD133 injection in the LVEF 6 months after surgery.

VERSION IDENTIFICATION FINAL 1.0 – 08 MARCH 2017

TABLE OF CONTENTS

1 INTRODUCTION 18

2 STUDY OBJECTIVES 20
2.1 Primary Objective 20
2.2 Secondary Objectives 20

3 INVESTIGATORS AND STUDY ADMINISTRATIVE STRUCTURE 21

4 ETHICS 26
4.1 Independent Ethics Committee 26
4.2 Ethical conduct of the study 27
4.3 Patient information and consent 27

5 INVESTIGATIONAL PLAN 28
5.1 Overall study design 28
5.2 Discussion of study design 28
5.3 Selection of study population 29
5.3.1 Inclusion criteria 29
5.3.2 Exclusion criteria 29
5.3.3 Removal of patients from therapy or assessment 30
5.4 Treatments 31
5.4.1 Identity of investigational and reference product(s) 31
5.4.2 Treatment administered 31
5.4.3 Treatment compliance and drug accountability 32
5.4.4 Prior and concomitant medication 32
5.4.5 Treatment allocation and randomization 33
5.4.6 Blinding 33
5.4.7 Labelling 35
5.5 Study procedures and study schedule 35
5.5.1 Schedule of assessments 35
5.5.2 Overview of data collection 38
5.5.3 Study flow chart 42
5.6 Changes in the conduct of the study 42

6 DATA QUALITY ASSURANCE 44

7 STATISTICAL METHODS 45
7.1 Primary Objectives 45
7.2 Secondary Objectives 45
7.3 Planned Analyses 45
7.3.1 Interim Analysis (IA) 45
7.3.2 Main Analysis (MA) 45
7.3.3 Safety-Follow-up-Analysis (SFUA) 46
7.4 Sample size justification 46
7.5 Populations of analysis 46
7.5.1 All Patients Consented Set (APCS) 46
7.5.2 Full Analysis Set (FAS/ITT) 46
7.5.3 Safety Analysis Set (SAS) 46
7.5.4 Insufficient CD133+ Analysis Set (I-CD133+-AS) 46
7.5.5 Per Protocol Set (PPS) 47
7.5.6 SAS II and SAS III 47
7.6 Analyses 47

7.6.1 Primary efficacy ... 47
7.6.2 Secondary efficacy ... 48
7.6.3 Safety ... 48
7.6.4 Questionnaires ... 50
7.6.5 Disposition of Patients ... 50
7.7 Changes in planned analyses ... 50

8 INTERIM ANALYSES ... 52

9 STUDY PATIENTS ... 53
9.1 Patient disposition ... 53
9.2 Protocol deviations and violations ... 53
9.3 Study populations analysed ... 54
9.4 Demographic and baseline characteristics ... 55
9.4.1 Medical History ... 55
9.4.2 Previous and Concomitant Medications ... 56

10 EXTENT OF EXPOSURE ... 57
10.1 Dosage and duration ... 57
10.2 Compliance ... 57

11 EFFICACY ANALYSES ... 58
11.1 Primary efficacy analysis ... 58
11.1.1 Results ... 58
11.1.2 Conclusions ... 69
11.2 Secondary efficacy analysis ... 69
11.2.1 Results ... 70
11.2.2 Conclusions ... 89

12 SAFETY ANALYSES ... 91
12.1 Adverse events ... 91
12.1.1 Summary of Adverse Events ... 91
12.1.2 Serious adverse events (including those leading to death) ... 95
12.1.3 Other significant adverse events ... 99
12.2 Clinical laboratory evaluation ... 101
12.2.1 Haematology ... 101
12.2.2 Blood chemistry ... 102
12.2.3 Urinalysis ... 105
12.3 Other safety data ... 105
12.3.1 Vital signs ... 105
12.3.2 Unwanted tissue changes ... 107
12.4 Safety conclusions ... 107

13 DISCUSSION AND CONCLUSIONS ... 109

14 TABLES, FIGURES, AND GRAPHS REFERRED TO BUT NOT INCLUDED IN THE TEXT. 112

15 REFERENCE LIST ... 118

16 APPENDIX A STUDY INFORMATION ... 120
16.1 Appendix A 1 Protocol, protocol amendments and Notes-to-File ... 121
16.2 Appendix A 2 Sample Case Report Form ... 122
16.3 Appendix A 3 Independent Ethics Committee membership list and sample patient information including Informed Consent Form ... 123
16.4 Appendix A 4 Brief résumé of Investigator and other important study personnel ... 124
16.5 Appendix A 5 Certificate of Analysis and Listing of Patients Receiving Investigational Drug(s) from Specific Batches ... 125
16.6 Appendix A 6 Randomisation list ... 126
16.7 Appendix A 7 Audit certificates ... 127

16.8 Appendix A 8 Statistical Analysis Plan ... 128
16.9 Appendix A 9 Laboratory accreditation, inter-laboratory standardization and reference ranges ... 129
16.10 Appendix A 10 Publications based on the study ... 130
16.11 Appendix A 11 Important publications referenced in the report ... 131

17 APPENDIX B STATISTICAL OUTPUT ... 132

18 APPENDIX C INDIVIDUAL DATA LISTINGS ... 133

19 APPENDIX D SELECTED CASE REPORT FORMS AND SUBJECT NARRATIVES ... 134

List of in-text tables

Table 1 Ethics committees that reviewed the study protocol 26

Table 2 Schedule of assessments 36

Table 3 Demographics of the patients included in the study........ 55

Table 4 Concomitant medication of specific interest 56

Table 5 Overall results of the ANCOVA for LVEF at 6 months in the FAS/ITT and PPS populations.. 59

Table 6 Overall results of the ANCOVA for LVEF in the FAS/ITT and PPS - MMRM 60

Table 7 Summary statistics of the change in LVEF assessed by MRI in the FAS/ITT and PPS 62

Table 8 LVEF summary statistics by visit and by treatment group assessed with MRI in the FAS/ITT and the PPS 63

Table 9 LV mass assessed with MRI in the FAS/ITT and the PPS 64

Table 10 LVEDV assessed with MRI in the FAS/ITT and the PPS 65

Table 11 LVESV assessed with MRI in the FAS/ITT and the PPS 66

Table 12 Total scar tissue measured with MRI in the FAS/ITT and the PPS 67

Table 13 Non-viable tissue measured with MRI in the FAS/ITT and the PPS 68

Table 14 Summary statistics of the change in LVEF assessed by echocardiography in the FAS/ITT and the PPS 70

Table 15 Quality of the echocardiography in the FAS/ITT and the PPS 71

Table 16 Difference from month 6 to screening and hospital discharge in LVEDD (echocardiography) in the FAS/ITT and the PPS, by treatment group 73

Table 17 Difference from month 6 to screening and hospital discharge in LVESD (echocardiography) in the FAS/ITT and the PPS, by treatment group 74

Table 18 Difference from month 6 to screening and hospital discharge in total distance (6MWT) in the FAS/ITT and the PPS, by treatment group 75

Table 19 Difference from month 6 to screening and hospital discharge in NYHA classification in the FAS/ITT and the PPS, by treatment group 77

Table 20 Difference from month 6 to screening and hospital discharge in CCS classification in the FAS/ITT and the PPS, by treatment group 78

Table 21 Number of MACEs during the main study phase up to Visit V in the FAS/ITT by treatment group 79

Table 22 EQ-5D - mobility - Difference between level at 6 months post-OP to screening and 3-months post-OP 81

Table 23 EQ-5D – VAS - Difference between level at 6 months post-OP to screening and 3-months post-OP 82

Table 24 MLHF-Q – Emotional score - Difference between level at 6 months post-OP to screening and 3-months post-OP 83

Table 25 MLHF-Q – Physical score - Difference between level at 6 months post-OP to screening and 3-months post-OP 85
Table 26 MLHF-Q – Total score - Difference between level at 6 months post-OP to screening and 3-months post-OP 86
Table 27 SF36 – Mental Health - Difference between level at 6 months post-OP to screening and 3-months post-OP 88
Table 28 SF36 – General health - Difference between level at 6 months post-OP to screening and 3-months post-OP 89
Table 29 Summary of adverse events during screening phase 92
Table 30 Classification of the AEs during screening per severity, relationship to treatment and outcome by treatment group 93
Table 31 Summary of adverse events during the main trial phase 94
Table 32 Classification of the AEs during the main trial phase per severity, relationship to treatment and outcome by treatment group 95
Table 33 All Serious Adverse Events during Main Trial Phase by Treatment Group and System Organ Class 96
Table 34 AESIs as defined in the SAP and how they were coded for the analysis 100
Table 35 Haematology values at Visit I and Visit V by treatment group 101
Table 36 New abnormalities in the haematology values at Visit V by treatment groups 102
Table 37 Blood chemistry values at Visit I and Visit V by treatment group 102
Table 38 Blood lipids at Visit I by treatment group 104
Table 39 NT-pro-BNP- at Visit I and Visit V by treatment group 104
Table 40 New abnormalities in the blood chemistry values at Visit V by treatment groups 105
Table 41 Vital signs at Visit V and the difference vs. Visit I per treatment group. 107

List of in-text figures

Figure 2 Left ventricular segmentation 40
Figure 3 Flow chart of the study 42
Figure 5 Number of patients in each analysis set 54
Figure 6 Evolution of the mean of LVEF for the different treatment groups in the FAS/ITT 61
Figure 7: Rates of sudden death/cardiac arrest according to LVEF (Solomon et al.) 109
Figure 8: 6-month all-cause mortality in five categories of LVEF (Volpi et al, 1993) 110

LIST OF ABBREVIATIONS

6MWT	6-Minute Walk Test
AE	Adverse Event
AESI	Adverse Event of Special Interest
AR	Adverse Reaction
CABG	Coronary Artery Bypass Graft
CCS	Canadian Cardiovascular Society
CRF	Case Report Form
ECG	Electrocardiogram
EQ-5D	EuroQol five dimensions questionnaire
FAS	Full Analysis Set
FAS/ITT	Full Anaysis Set / Intent to treat
GMP	Good Manufacturing Practice
IA	Interim Analysis
ICF	Informed Consent Form
ICH GCP	Tripartite Guidelines Guideline for Good Clinical Practice
ICU	Intensive Care Unit
IMP	Investigational Medicinal Product
ITT	Intent-to-treat
LA	Left Atrium
LMCA	Left Main Coronary Artery
LVEDV	Left Ventricular End Diastolic Volume
LVEF	Left Ventricular Ejection Fraction
LVESD	Left Ventricular End Systolic Dimension
LVOT	Left ventricular Outflow Tract
LVPWD	Left Ventricular Posterior Wall Diameter
MA	Main Analysis
MACE	Major Adverse Cardiovascular Events
MLHF-Q	Minnesota Living with Heart Failure Questionnaire
MMRM	repeat measures approach
MRI	Magnetic Resonance Imaging
NYHA	New York Heart Association

PPS	Per Protocol Set
QoL	Quality of Life
RA	Right Atrium
RCA	Right Coronary Artery
RCX	Ramus Circumflex Artery
RIVA	Ramus Interventricular Anterior
RVEDD	Right Ventricular End Diastolic Dimension
RVOT	Right Ventricular Outflow Tract
SAE	Serious Adverse Event
SAP	Statistical Analysis Plan
SAR	Serious Adverse Reaction
SAS	Safety Analysis Set
SF-36	Short Form Questionnaire
SFUA	Safety-Follow-up-Analysis
SMB	Safety Monitoring Board
SOP	Standard Operating Procedure
SUSAR	Suspected Unexpected Serious Adverse Reaction
VA	Late (diastolic peak flow) Velocity
VAS	Visual Analogue Scale
VE	Early (diastolic peak flow) Velocity
VES	Ventricular Extrasystoles

1 INTRODUCTION

Despite significant improvements in emergency treatment, myocardial infarction leads to a net loss of contractile tissue that can lead to congestive heart failure and life-threatening arrhythmias in many patients with coronary artery disease. Other than heart transplantation, current therapies aim at preventing further episodes of myocardial ischemia and enabling the organism to survive with a heart that is working only at a fraction of its original capacity. This is far from a cure. In this situation, it is understandable that cardiac stem cell therapy attracts considerable attention.

The myocardium consists of terminally differentiated cells without a clinically relevant potential for regeneration and dead cells cannot be replaced by new contractile cells. Instead, remodelling processes lead to interstitial myocardial fibrosis or formation of a transmural fibrotic scar that further impairs systolic and diastolic ventricular function. Surgical or interventional revascularization of ischemic myocardium effectively treats angina, prevents myocardial infarction, improves function of still viable myocardium, and pharmacological therapy appears to have a beneficial impact on remodelling processes. However, viability and function of necrotic myocardium cannot be restored with current therapeutic options. In the last 15 years, the transplantation of cells into infarcted myocardium has evolved into a possible mean to achieve this goal (1, 2).

In previous studies the safety, feasibility and, in part, efficacy of Intramyocardial bone marrow stem cell transplantation were demonstrated in humans (1). In all trials a significant improvement of left ventricular (LV) function and/or improved myocardial perfusion was shown. There were no procedure-related complications, especially no new ventricular arrhythmias or neoplasia. In addition, the intracoronary administration of bone-marrow stem cells after acute myocardial infarction has shown no serious side effects (3-7). The intracoronary application, however, is not efficient in chronic ischemic heart disease and has limited functional benefit in acute myocardial infarction (8-10).

In previous phase I (15 patients; 2001-2003) and phase II (20+20 patients; 2003-2005) studies involving Intramyocardial injection of CD133+ selected bone marrow stem cells there were no adverse events (AEs) related to stem cell injection. Long-term mortality analysis showed that until 2006 two patients died late after operation. One patient died 7 months after cardiac artery bypass graft (CABG) operation (phase I), and one patient 14 months after operation (phase II, study control group) (11). Based on this knowledge a favourable risk-benefit rate was expected for this study.

Based on the existing experience, it seemed justified to conclude that transplantation of purified CD133+ autologous bone marrow cells in the infarct border zone can be safely performed in patients with ischemic heart disease.

This study was designed to demonstrate the therapeutic effect of CD133+ isolated bone marrow stem cell injection into the myocardium in post infarct patients undergoing CABG operation.

Risk-Benefit Assessment

Patients with coronary artery disease have an unsatisfactory quality of life (QoL) and a poor survival prognosis. Conventional treatments including CABG, intracoronary vascular stents and pharmacological support do not cure the coronary artery disease. Conventional treatments do not recruit hibernating myocardium by angiogenesis and do not prevent a sequential loss of heart tissue. Bone marrow stem cells are capable to induce angiogenesis (limited to small vessels) in ischemic tissue and therefore it could be used to treat this affection. Therefore, the combination of macrovascularization by CABG-surgery with microvascularization by bone-marrow stem cells was expected to give a maximal benefit to rescue ischemic heart tissue of the patient. The potential risks of the treatment can be minimized by

several proven measures for the selection, preparation, and application of bone-marrow stem cells of the patient. The available information suggested that the present study had a favourable risk-benefit ratio.

2 STUDY OBJECTIVES

2.1 Primary Objective

Determine whether injection of autologously-derived bone marrow stem cells yielded a functional benefit in addition to the CABG operation as determined by left ventricular heart function (left ventricular ejection fraction [LVEF] determined with magnetic resonance imaging [MRI]).

2.2 Secondary Objectives

Determine the effects of an injection of autologously-derived bone marrow stem cells on physical exercise capacity, cardiac function, safety and QoL.

3 INVESTIGATORS AND STUDY ADMINISTRATIVE STRUCTURE

Sponsor:	Miltenyi Biotec GmbH<br>Friedrich-Ebert-Straße 68<br>51429 Bergisch Gladbach<br>Germany<br>Phone: +49 (2204) – 8306-2823<br>Fax: +49 (2204) – 8306-6699<br>E-mail: janko@miltenyibiotec.de
Principal Investigator:	Prof. Dr. med. Gustav Steinhoff<br>Universität Rostock<br>Direktor Klinik und Poliklinik für Herzchirurgie, Schillingallee 35<br>D-18057 Rostock<br>Phone: +49 (381) 494-6100<br>E-mail: gustav.steinhoff@med.uni-rostock.de
Participating site(s):	Prof. Dr. med. Christof Stamm<br>Deutsches Herzzentrum Berlin<br>Klinik für Herz-, Thorax- und Gefäßchirurgie<br>Augustenburger Platz 1<br>D-13353 Berlin<br>Phone: +49 (30) 4593- 2109<br>Fax: +49 (30) 4593-2100<br>E-mail: stamm@DHZB.de
	Prof. Dr. med. Dr. h. c. Axel Haverich<br>Medizinische Hochschule Hannover<br>Klinik für Thorax-, Herz- und Gefäßchirurgie<br>Carl-Neuberg Strasse 1<br>D-30625 Hannover<br>Phone: +49 (511) 532 65 80<br>Fax: +49 (511) 532 5404<br>E-mail: haverich.axel@mh-hannover.de
	Prof. Dr. med. Gustav Steinhoff<br>Universität Rostock<br>Klinik und Poliklinik für Herzchirurgie<br>Schillingallee 35<br>D-18057 Rostock<br>Phone: +49 (381) 494-6100<br>Fax: +49 (381) 494-6102<br>E-mail: gustav.steinhoff@med.uni-rostock.de
	Dr. med. Jochen Börgermann

	Herz- und Diabeteszentrum Nordrhein Westfalen<br>Universitätsklinik der Ruhruniversität Bochum Georgstraße 11<br>32545 Bad Oeynhausen<br>Phone: +49 (5731) 97 32 47<br>Fax: +49 (5731) 97 1820<br>e-mail: jboergermann@hdz-nrw.de
	Prof. Dr. med. Friedrich-Wilhelm Mohr<br>Klinik für Herzchirurgie<br>Herzzentrum Universität Leipzig<br>Strümpellstraße 39<br>04289 Leipzig<br>Phone: +49(341)865-1421<br>Fax: +49(3 41)865-1452<br>e-mail: chir@herzzentrum-leipzig.de
	PD. Dr. med. Florian Mathias Wagner<br>Klinik und Poliklinik für Herz- und Gefäßchirurgie<br>Universitäres Herzzentrum Hamburg<br>Martinistr. 52<br>20246 Hamburg<br>Phone: +49 (40) 7410-58949<br>Fax: +49 (40) 7410-57926<br>e-mail: fl.wagner@uke.de
Laboratory facilities:	Central laboratory Cell Processing<br>Seracell Stammzelltechnologie GmbH<br>Schillingallee 68<br>18057 Rostock<br>Phone: +49 (381) 4443-1270<br>Fax: +49 (381) 4443-1279<br>E-mail: hans-dieter.kleine@med.uni-rostock.de
	Core Lab MRI Analysis<br>Medizinische Hochschule Hannover<br>Diagnostische Radiologie<br>Carl-Neuberg-Str.1<br>Gebäude K5, Ebene HO<br>D – 30623 Hannover<br>PD Dr. med. Dagmar Hartung<br>Phone: +49 (511) 532-3421<br>Fax: +49 (511) 532- 9421<br>E-mail: hartung.dagmar@mh-hannover.de<br>Prof. Dr. med. Joachim Lotz<br>Phone: +49 (551) 39-89 65<br>Fax: +49 (551) 39-96 06

	E-mail: joachim.lotz@med.uni-goettingen.de
	Core Lab Echocardiography Analysis<br>Dr. med. Julia Gensicke<br>Deutsches Herzzentrum Berlin<br>Klinik für Herz-, Thorax- und Gefäßchirurgie<br>Augustenburger Platz 1<br>D-13353 Berlin<br>Phone: +49 (30) 4593-2319<br>Fax: +49 (30) 4593-2100<br>E-mail: gensicke@dhzb.de
Monitoring:	Freelance CRAs:<br>Tatjana Alberg<br>proclintas - professional clinical trial assistance<br>Gehägestrasse 46a;<br>D - 30655 Hannover<br>Tel.: 0511 69 20 30
	Ulrike Hess<br>Kirchstr. 8a<br>38667 Bad Harzburg<br>Tel.: 05322 559 053 8
	Heike Kurzidim<br>Clinical Research Manager<br>Lottoweg 8<br>04838 Doberschütz<br>Tel. 034244-51 295
Data management:	KOEHLER eClinical GmbH<br>Hornusstrasse 16<br>79108 Freiburg<br>Godehard Höxter<br>Phone: +49-761-50318-627<br>Fax: +49-761-50318-7627
Statistics & reporting:	Prof. Dr. -Ing. Günther Kundt<br>Universität Rostock<br>Institut für Medizinische Informatik und Biometrie<br>Rembrandtstr. 16/17<br>D-18057 Rostock<br>Phone: +49 (381) 494-7311<br>Fax: +49 (381) 494-7203<br>E-mail: guenther.kundt@uni-rostock.de

	Dipl.-Math. H. Lorenz,<br>Im Unterfeld 17,<br>D-63543 Neuberg<br>Phone: +49 (0)6183 73071
SUSAR Reporting<br>DSUR Preparation	SCRATCH Pharmacovigilance GmbH<br>Färbgasse 24<br>35510 Butzbach<br>Dr. Susanne Kienzle-Horn<br>Phone: +49 6033 74535512
Adverse Event Reporting	Miltenyi Biotec GmbH, Bergisch Gladbach<br>Friedrich-Ebert-Straße 68<br>D-51429 Bergisch Gladbach<br>Dr. med. Liane Peußner<br>Phone: +49 (2204) – 8306-3950<br>Fax: +49 (2204) – 8306-6699<br>E-mail: lianep@miltenybiotec.de
Stem Cell Manufacturer	Murat Aktas<br>Miltenyi Biotec GmbH, Bergisch Gladbach<br>Friedrich-Ebert-Straße 68<br>D-51429 Bergisch Gladbach<br>Tel.: +49 15117151803<br>Fax: +49 170 41 06 162<br>E-mail: muratak@miltenyibiotec.de
Central Lab<br>Cell Processing	Seracell Stammzelltechnologie GmbH<br>Schillingallee 68<br>18057 Rostock<br>Phone: +49 (381) 4443-1270<br>Fax: +49 (381) 4443-1279<br>E-mail: hans-dieter.kleine@med.uni-rostock.de
Safety Monitoring Board	Giulio Pompilio, M.D. Ph.D.<br>Department of Cardiovascular Surgery, Centro Cardiologico Monzino IRCCS<br>Via Parea 4<br>20138 Milano, Italy.<br>Phone: +39 (02)58002562<br>Fax: +39 (02)58011194<br>e-mail: giulio.pompilio@ccfm.it<br><br>Prof. Dr. med. Johannes Waltenberger

Albert-Schweitzer-Campus 1, Gebäude A1,
48149 Münster,
Germany
Phone: +49 (251) / 83 - 4 32 01
e-mail: johannes.waltenberger@ukmuenster.de

Francesco Siclari, M.D. Ph.D.
Cardiocentro Ticino
Via Tesserete 48
Lugano 6900
Switzerland
Phone: +41 (0) 91 805 31 44 –
Fax: +41 (0) 91 805 31 48
e-mail: francesco.siclari@cardiocentro.org

4 ETHICS

4.1 Independent Ethics Committee

The protocol and Informed Consent Form (ICF) were approved by the Ethikkommission der Ärztekammer Mecklenburg-Vorpommern der Universität Rostock on 19 September 2007 before the study initiation.

Other Ethics Committees involved in the analysis and evaluation of the protocol were:

Table 1 Ethics committees that reviewed the study protocol

Ethics Committees	Director / Contact person
Ethikkommission der Medizinischen<br>Fakultät der Universität Rostock<br>Institut für Rechtsmedizin<br>St.-Georg-Straße 108<br>18055 Rostock	Herr Prof. Dr. med. A. Büttner
Ethikkommission der Medizinischen<br>Hochschule Hannover<br>Carl-Neuberg-Str. 1<br>30625 Hannover	Herr Prof. Dr. med. H. D. Tröger
Landesamt für Gesundheit und Soziales Berlin<br>Geschäftsstelle der Ethik-Kommission<br>des Landes Berlin<br>Fehrbellliner Platz1<br>10707 Berlin	Herr von Dewitz
Ethikkommission der<br>Ärztekammer Hamburg<br>Weidestr. 122b<br>22083 Hamburg	Frau Dr. Silke Schrum
Ethikkommission der Medizinischen<br>Fakultät der Universität Leipzig<br>Haus: Karl-Sudhoff-Institut<br>Käthe-Kollwitz-Straße 82<br>04109 Leipzig	Herr Prof. Dr. med. R. Preiß
Ethik-Kommission der Med. Fakultät<br>der Ruhr-Uni. Bochum, Sitz Bad Oeynhausen<br>Herz- und Diabeteszentrum NRW<br>Georgstr. 11<br>32545 Bad Oeynhausen	Herr Prof. Dr. med. Wolfgang Burchert

The protocol and all amendments (9) are presented in Appendix A1. All protocol amendments were approved by the corresponding Ethic Committee. All study data were collected on the Case Report Form (CRF). Unique pages of the CRF are presented in Appendix A2.

4.2 Ethical conduct of the study

The study was performed in compliance with the 'Declaration of Helsinki' [Fortaleza, Brasil, 2013[1]], the International Conference of Harmonization Tripartite Guidelines Guideline for Good Clinical Practice (ICH GCP), current international and national regulations, the study protocol and current Standard Operating Procedures (SOPs) of the participating sites, of Miltenyi Biotec GmbH, and KOEHLER eClinical GmbH. These procedures were to ensure the protection of the rights and the integrity of the patients, adequate and correct conduct of all study procedures, adequate data collection, documentation, and data verification. The SOPs relevant to the context of the study are available at the sponsor.

4.3 Patient information and consent

Written informed consent was obtained from each patient or his legal representative before entering the study. The patient information and the Informed Consent Form (ICF) are presented in Appendix A3.

1 http://www.wma.net/en/30publications/10policies/b3/

5 INVESTIGATIONAL PLAN

5.1 Overall study design

The study was planned as a placebo controlled, prospective, randomized, double-blind multicentre (7 centres), phase III, clinical trial investigating the effects of intramyocardial injection of 5 mL CD133+ bone marrow cells or placebo in 142 patients with coronary artery disease scheduled for CABG surgery. Patients were to be randomized to one of the two treatment groups (CD133+ or placebo) in a 1:1 ratio.

The primary efficacy end-point was the LVEF at 6 months postoperatively, measured by MRI at rest and change in LVEF at 6 months post-OP compared with preoperatively (screening) and early postoperatively (discharge) as assessed by MRI.

The secondary efficacy end points were:

- Change in LVEF at 6 months post-OP compared with preoperatively (screening) and early postoperatively (discharge) as assessed by echocardiography.
- Change in LV dimensions ([LVESD], left ventricular end diastolic dimension [LVEDD]) at 6-month post-OP compared with preoperatively (screening) and early postoperatively (discharge) as assessed by echocardiography.
- Change in physical exercise capacity determined by 6-minute walk test at 6 months post-OP compared with preoperatively (screening) and early postoperatively (discharge).
- Change in New York Heart Association (NYHA) and Canadian Cardiovascular Society (CCS) class at 6 months post-OP compared with preoperatively (screening) and early postoperatively (discharge).
- Major adverse cardiovascular events (MACE - cardiac death, myocardial infarction, secondary intervention/reoperation, ventricular arrhythmia).
- QoL-score at 6 months post-OP compared with preoperatively (screening) and 3 months (telephone).

Safety end points were all AEs.

All patient and trial relevant data were collected in the CRFs.

5.2 Discussion of study design

The study design results from the sponsor’s experience with phase I and phase II studies. CD133+ positive bone-marrow stem cells were isolated from the patients’ own bone marrow (iliac crest puncture) and prepared per Good Manufacturing Practice (GMP) standards. Small volume preparations of 5 mL were found safe for intramyocardial application using 0.2–0.3 mL volume per injection. The distribution of injection-sites was found best using one injection per square cm and 26-gauge injection needles. The injections were placed in the borderline tissue around patients’ heart infarction, so that 10-15 injections were needed to circle around the infarction area.

The main limitation of the previous controlled studies was the absence of a placebo-controlled randomized group to exclude influence of intramyocardial injection (saline) on myocardial function. No side-effects were found in the safety and efficacy trial. No rescue medication was necessary.

The sponsor chose the LVEF 6 months after CABG surgery by MRI as a primary endpoint to measure the effect on regeneration of the functionally impaired left ventricle after myocardial infarction. MRI is considered as gold standard for measurement of left ventricular function parameters. To exclude bias the analysis was to be done by a central MRI core-lab.

Additional secondary endpoints, such as physical exercise capacity, echocardiography, safety and QoL would underline the effects of the injection of autologously derived bone marrow stem cells.

5.3 Selection of study population

5.3.1 Inclusion criteria

Patients were included in the study if they met *all* the following criteria:

1. Coronary artery disease after myocardial infarction with indication for CABG surgery
2. Currently reduced global LVEF assessed at site by cardiac MRI at rest (25% ≤ LVEF ≤ 50%)
3. Presence of a localized akinetic/hypokinetic/hypoperfused area of LV myocardium for defining the target area
4. Informed consent of the patient
5. 18 years ≤ Age < 80 years
6. Not pregnant and not planning to become pregnant during the study. Females with childbearing potential had to provide a negative pregnancy test within 1-7 days before OP and had to be using oral or injectable contraception (non-childbearing potential is defined as post-menopausal for at least 1 year or surgical sterilization or hysterectomy at least 3 months before study start).

5.3.2 Exclusion criteria

Patients were excluded from the study if they met *any* of the following criteria:

1. Emergency operation
2. Presence of any moderate-severe valve heart disease requiring concomitant valve replacement or reconstruction
3. Medical History of recent resuscitation in combination with ventricular arrhythmia classified by LOWN ≥ class II
4. Acute myocardial infarction within last 2 weeks
5. Debilitating other disease: Degenerative neurologic disorders, psychiatric disease, terminal renal failure requiring dialysis, previous organ transplantation, active malignant neoplasia, or any other serious medical condition that, in the opinion of the Investigator is likely to alter the patient’s course of recovery or the evaluation of the study medication’s safety
6. Impaired ability to comprehend the study information
7. Absence of written informed consent
8. Treatment with any investigational drug within the previous 30 days
9. Apparent infection (c-reactive protein [CRP] ≥ 20 mg/L, fever ≥ 38.5° C)
10. Contraindication for MRI scan
11. Immune compromise including Anti human immunodeficiency virus (HIV) 1/2, HBsAg, Anti-HBc-IgG, Anti hepatitis C virus, Treponema pallidum
12. Pregnant or breast feeding
13. Childbearing potential with unreliable birth control methods
14. Have previously been enrolled in this study, respectively phase I and phase II
15. Known hypersensitivity or sensitization against murine products and human-anti-mouse-antibody-titer ≥ 1:1000
16. Contraindication to bone marrow aspiration
17. Known hypersensitivity against iron dextran

5.3.3 Removal of patients from therapy or assessment

Patients could withdraw from the study at any time without penalty and for any reason without prejudice to his or her future medical care. All patients were closely monitored by their individual cardiologist and general practitioner and treated as appropriate, per the guidelines issued by the national and international cardiology organizations that relate to patients with coronary artery disease and patients with congestive heart failure. The Investigators reserved the right to conduct additional follow-up examinations.

Patients had to be withdrawn under the following circumstances:

- The patient withdrew consent;
- Pregnancy. If patients were withdrawn due to pregnancy, they were to be followed-up until the outcome for the mother and foetus were known.
- If serious clinical events (e.g., emergency surgery, myocardial infarction, stroke, fever ≥ 38.5 °C) occurred between recruitment and planned surgery.

Patients could be required to withdraw after discussion with the Sponsor and/or Investigator for the following reasons:

- Adverse event(s);
- At the discretion of the Investigator due to medical reasons;
- Violation of eligibility criteria;
- Deviation from the treatment plan specified in the protocol (e.g., incorrect administration of the study drug, failure to attend study visits).

In all cases, the reason(s) for withdrawal, and the primary reason, were to be recorded on the CRF. If a patient was prematurely withdrawn from the study for any reason, the Investigator had to make every effort to perform the evaluations described for the Early Termination Visit (see Section 7.2.6.2 of the Study Protocol).

An excessive withdrawal of patients could have rendered the study uninterpretable. Therefore, unnecessary withdrawal of patients had to be avoided. In case of withdrawal, all efforts had to be made to complete and report the observation and justification of withdrawal in as much detail as possible. Withdrawn patients that dropped-out of the study because of a serious adverse event (SAE) were not to be replaced. However, patients who were not able to reach the primary endpoint because of their body size/weight could be replaced.

Patients from both groups, who either received Placebo or CD133+ cells could be excluded post-hoc from the study because of insufficient CD133+ cell counts (<0.5 Mio but ≥0.1 Mio). In case the drop-out rate exceeded the assumed rate of 15%, the patients could be replaced. Furthermore, patients who were randomized but terminated the study before treatment start could be replaced.

Former studies and experiments have shown that on average, more than $5x10^6$ CD133+ bone marrow stem cells can be isolated from 200 mL bone marrow aspirate (12). In this trial $0.5\text{-}5x10^6$ CD133+ cells isolated from the harvested bone marrow were to be administered. Cell yields lower than the expected $0.5x10^6$ but $\geq 0.1x10^6$ were to be documented and the patients were to be regarded as drop-outs and followed-up like the intend-to-treat population and evaluated separately.

Cell yields lower than $0.1x10^6$ were to be documented and the product was not to be released. The patient would be dropped out of the study and the treating physician and Statistician informed. These

patients were to be followed-up. In case the over-all drop-out rate exceeded the assumed 15% those patients that dropped-out post-hoc because of insufficient cell counts could be replaced.

5.4 Treatments

5.4.1 Identity of investigational and reference product(s)

All patients enrolled in the study underwent bone marrow aspiration (150-200 mL) and withdrawal of 20 mL blood one to two days before CABG surgery. The CD133+ cells were selected from the bone marrow aspirate of each patient and:

- patients in the active group received the CD133+ cells suspended in physiological saline + 10% autologous serum.
- patients of the control group received the placebo preparation. Their CD133+ cells were stored at the manufacturer.

After the selection process using the ClinMACS+, the CD133+ cells were suspended in 5 mL of saline supplemented with 10% autologous serum and were drawn into 5x1 mL syringes. To ensure consistent quality and individual safety of the cell product, the product was released only if the following specifications regarding the validated manufacturing process had been met:

- Minimum number of CD133+ cells = $0.5x10^6$ cells
- Maximum number of CD133+ cells = $5x10^6$ cells
- Minimum depletion of non-target cells = 2.5 log (> 99.6%)
- Minimum percentage of viable cells = 80%
- Manufacturing and Quality Control per GMP

Placebo preparations consisted of 5 mL of saline supplemented with 10% autologous serum and were also drawn in 5x1 mL syringes.

The 5 syringes were packed in an outer package by the manufacturer and were administered within a maximum of 72 hours after aspiration.

Product batches were prepared for each individual patient and per the specifications (See investigational medicinal product [IMP] in Appendix A5)

5.4.2 Treatment administered

Patients randomized to the active treatment group were given 5 mL CD133+ cells, saline and serum suspension intramyocardially during CABG surgery.

Patients randomized to placebo treatment were given 5 mL saline plus serum solution intramyocardially during CABG surgery.

For both treatment groups, the treatments were administered intramyocardially in the infarction border zone (penumbra) during the cardiac surgical procedure. The procedure was performed with extracorporeal circulatory support, aortic cross clamping and cardiac arrest induced by cardioplegia per the centre standards. The treatments were administered before cross clamp release.

The 5 mL suspension were distributed in 15 individual 1 mL syringes (26 Gauge needle) of 0.3 mL aliquots (in total 5 mL, including up to 0.5 mL rest in syringes) ad were applied within 3 minutes in the region of interest (infarction border zone) at the end of bypass surgery. No more than one injection per square centimetre could be injected.

During the whole duration of the study, patients were treated per the standards of the centres and the American Heart Association guidelines for current standard of care.

5.4.3 Treatment compliance and drug accountability

Since the study treatment was administered by the Investigator, patient compliance with study treatment was not monitored.

Patients that are were non-compliant with the study protocol such as non-attendance at study visits or refusal to undergo certain assessments were candidates for patient withdrawal (see Section 5.3.3). The Investigator was responsible for maintaining accurate study drug accountability records throughout the study. The dispensing of the study treatment was documented in the CRF “intra OP” on day 0.

The Investigator was responsible of ensuring that all unused or partially used study treatment was disposed as per the Transfusionsgesetz - § 17 (blood transfusion law, paragraph 17) and the local regulations for biological products.

Study treatment which had not left the manufacturer facilities and that was not to be used for patient treatment or super numerous stem cells after manufacturing were to be used for research or had to be destroyed per the patient information and the informed consent. The manufacturer ensured, that disposal followed the Transfusionsgesetz - § 17 and its regulations for biological products.

5.4.4 Prior and concomitant medication

Any medication for the patients’ treatment per the guidelines and the standards of the centres was permitted.

Any other medication, other than the study drug, including herbal and other non-traditional medicinal products, was considered a concomitant medication. All concomitant medications were recorded in the CRF "Concomitant Medication": generic name, indication, route of administration, dose rate with unit of measurement, date started (before trial or date), date stopped (ongoing or date) and application (continuous or as necessary). Any change in the dosage or regimen of a concomitant medication was recorded in the CRF.

At screening, patients were asked what medications they were currently taking. Additionally, any platelet aggregation inhibitors they had taken during the previous 2 weeks was documented. At each subsequent assessment, any new concomitant medication and any changes in concomitant medications was documented.

The following medications were of special interest:

- ACE-inhibitor
- Aldosteron-Antagonist
- Beta-blocker
- Ca-Antagonist
- Digitalis
- Antiarrhythmic other
- ASS
- ATII Receptor Antagonist
- CSE-inhibitor
- Diuretic
- Marcumar
- Nitrate

Standard medication (narcotics, etc.) used during the CABG surgery was not documented in the CRF. Any medication for support of the cardiovascular system that was given to the patient during the surgery was documented.

Only medication administered for the treatment of Adverse or Serious Adverse Events was to be documented.

During the stay in the Intensive Care Unit (ICU), only the maximum doses of catecholamines, inotropics and any medication for the treatment of adverse or serious adverse events were documented.

5.4.5 Treatment allocation and randomization

Upon enrolment, each patient received a four-digit patient number consisting of (x yza). The 1st digit assigned x, indicated the study site (–see List of trial personnel), the 2nd, 3rd and 4th digit assigned, yza, indicated the individual patient. Enrolled patients who dropped out of the study before randomization retained their patient number.

Randomization to study treatment occurred at Assessment Ia (cell-preparation) after all screening procedures had been performed, eligibility for the study confirmed and after bone-marrow aspiration. The randomization was attributed by Team A (cell processing team).

Randomized patients who terminated their study participation for any reason, regardless of whether study intervention was done or not, retained their randomization number. The randomization code was assigned using sealed envelopes provided to the Team A for each study centre.

Patients were randomized on a 1:1 basis to receive CD133+ cells or placebo.

The randomization procedure was stratified by study site. Six randomization lists (one per centre) were provided to the bone-marrow isolation laboratory (Team A) which prepared the investigational product. The randomization procedure used was the Permuted Block Design within strata (13). The block size within each stratum was randomly selected. The size of the randomization blocks as well as seed numbers were documented but were not disclosed to the study centres to avoid predictability of treatment. The study treatment was randomly assigned to the randomization numbers in advance and per the randomization list held by the Biostatistician.

The randomization envelopes were numbered in an ascending order. If a patient was to be randomized a member of Team A opened the envelope with the lowest number among all unused sealed envelopes available for the centre. Patient was randomized per the information in the envelope, and CD133+ cell or the placebo product was produced for the current patient.

The randomization code was stored by the Statistician. Only the members of Team A had access to the randomization code. All members signed an agreement, stating that the randomization code was to be kept confidential and no person outside the Team A had access to the code.

Sealed emergency envelopes were to be stored at each site in a secure place containing the randomization code. In case of an emergency which required knowledge of treatment the treatment code for an individual patient could be revealed to a member of Team B (treatment team) by opening the sealed envelope.

5.4.6 Blinding

The study was performed in a double-blind manner. The appearance of the final placebo and cellular product was indistinguishable to the Investigators. Cell concentrations of 0.2 to 2.0 x 10^6 were not

detectable by pure vision in the type of syringes used for the application. The patients were blinded to the treatment they received. The surgeons and the Investigators involved in the preoperative and postoperative assessments were blinded regarding the treatment group assignment (Team B). Only the laboratory personnel involved in the cell isolation process was not blinded (Team A).

Team A (cell processing team) was responsible for the preparation of the medicinal product as well as for the preparation of the placebo control and for randomization. This team was not involved in patient recruitment/selection, clinical assessment, data collection or the treatment/sham injection. These unblinded team members could not reveal the identity of the study medication at any time.

Team B (treatment team) was responsible for the patient recruitment/selection, clinical assessment, data collection, bone marrow harvest, and performed the treatment/sham injection. The members of Team B were unaware of the randomization code and blinded to the treatment.

In case of an emergency, and necessity for breaking the code, an emergency envelope was available 24 hours a day, 7 days a week for a member of Team B. When breaking the code, a member of Team B signed and dated the emergency envelope which had been unblinded. The treatment code for an individual patient could be revealed to a member of Team B only in case of an emergency which required knowledge of treatment. At the end of the trial all emergency envelopes were returned to the statistician and were checked for integrity of the seal.

The study blind could not be broken except in a medical emergency (where knowledge of the study drug received would affect the treatment of the emergency) or regulatory requirement (e.g., for SAEs or death).

The investigator had to promptly document and explain to the sponsor any code breaking (e.g. accidental unblinding, unblinding due to a serious adverse event) of the investigational product. If the blind was broken, the date, time and reason had to be documented in the patient's CRF, and any associated AE report.

If an emergency unblinding became necessary, the Investigator had to notify the Sponsor/Medical Monitor, if possible, prior to unblinding. The Investigator was responsible for opening the specified envelope, in the presence of a witness, both of whom had to sign and date the envelope.

All envelopes, whether sealed or opened, were returned to the statistician, and overviewed by the Sponsor at the end of the study.

The Safety Monitoring Board (SMB) had access to the randomization code, and the code could be broken after appropriate discussion with the Sponsor.

If an Investigator, site personnel performing assessments, or patient, was unblinded, the patient had to be withdrawn from the study and procedures accompanying withdrawal were to be performed. In cases where there were ethical reasons for the patient to remain in the study, the Investigator had to obtain specific approval from the corresponding Investigator or the Sponsor for the patient to continue in the study.

Serious unexpected suspected adverse reactions (SUSARs), which were patient to expedited reporting, had to be unblinded before submission to the Regulatory Authorities.

The overall randomization code was to be broken only for reporting purposes. This occurred once all final clinical data had been entered to the database, all data queries had been resolved, and the assignment of patients to the analysis populations had been completed.

5.4.7 Labelling

The cell suspension or placebo product was delivered in 1 mL opaque syringes which were packed for transport. The syringes were labelled in accordance with the applicable regulatory guidelines GCP-O §5 (2) 5 for labelling requirements for containers with no more than ten millilitres volume. The outer package was labelled with the complete requirements in accordance with GCP-O §5 (2) (with name, address and telephone number of the Sponsor, name and strength of the product, with a note, that the medicinal product was intended for use in a clinical trial, with precautions for the disposal of unused investigational medicinal product (IMP), with trial centre identification and patient identification number, and other details. Since the test product was an autologous product and only very limited storage was allowed, additional notes were on the outer package as: Do not irradiate! Pass on without delay! For immediate use!

5.5 Study procedures and study schedule

5.5.1 Schedule of assessments

Fehler! Verweisquelle konnte nicht gefunden werden. summarizes the assessments and their schedule.

Table 2 Schedule of assessments

Assessments	I	Ia	II	IIa	III	IV	V	VI
	Screening	BM transfer/ Cell Prep.	Intra-OP	Post OP /ICU	Hospital Discharge [e]	3-Month FU [e]	6-Month FU/ Early termin.	24-Month FU
Timepoint	Day -7 to -1	Day -2 to 0	Day 0	Day 1	Within 72 hrs of discharge	Month 3[e] (± 2 weeks)	Month 6 (±2 weeks)	Month 24[d] (±2 weeks)
Informed consent	X							
Patient Demographics	X							
Inclusion/exclusion criteria	X							
Medical history: general/cardiac /risk factors	X							
Physical examination: general/vital signs	X				X		X	X
Cardiac examination:								
NYHA and CCS criteria	X				X		X	
Heart catheterization	X							
Holter (ventricular extra systoles [VES], supraventricular extra systoles [SVES]), LOWN	X				X		X	X
12-lead electrocardiogram (ECG)	X			X	X		X	X
Cardiac MRI scan	X				X		X	
Echocardiography	X				X		X	X
Laboratory:								
Serology	X							
Serum pregnancy test[c]	X							
HAMA[g]	X							
Haematology, Chemistry, Electrolytes	X			X[b]	X		X	X
Blood Lipids	X							

Assessments	I	Ia	II	IIa	III	IV	V	VI
	Screening	BM transfer/ Cell Prep.	Intra-OP	Post OP /ICU	Hospital Discharge [e]	3-Month FU [e]	6-Month FU/ Early termin.	24-Month FU
Timepoint	Day -7 to -1	Day -2 to 0	Day 0	Day 1	Within 72 hrs of discharge	Month 3[e] (± 2 weeks)	Month 6 (±2 weeks)	Month 24[d] (±2 weeks)
N-Terminal pro-B-type Natriuretic Peptide	X				X		X	X
QoL questionnaires [a]	X					X	X	X
Six minute walk test (6MWT)	X				X		X	
Bone marrow aspiration/blood sample		X						
Randomization to treatment		X						
Cell/placebo preparation		X						
Cell/placebo transfer		X						
Injections of study treatment/ CABG			X					
MACE							X	X
Concomitant medications	X	X	X [f]	X	X	X	X	X
Adverse events/Serious Adverse Events	X	X	X	X	X	X	X	X

5.5.2 Overview of data collection

5.5.2.1 Primary efficacy end-point(s)

Evaluation of treatment efficacy was based upon assessment of the LVEF at 6 months postoperatively (Assessment V), measured by MRI at rest and was the primary efficacy end-point.

5.5.2.2 Cardiac MRI scan.

Parameters recorded were:

- Date of recording
- LV mass (g/m^2), body surface (m^2), weight (kg), height (cm)
- Left ventricular end diastolic volume (LVEDV), left ventricular end systolic volume (LVESV), LVEF (%), Scar tissue total (g), Non-viable tissue (g)
- Ventricle function and wall motion (quality) for segments 1-17 (Figure 1)
- Wall motion thickening (%)
- Regional muscle mass (g)
- Perfusion at stress and at rest for segments 1-17 (Figure 1)
- Vitality/late enhancement (LE) for segments 1-17 (Figure 1)
- LE volume (% LV mass), transmurality LE (%)
- Total assessment vitality for segments 1-17 (Figure 1)
- Unwanted tissue changes; no/yes, if yes, describe
- Pericardial effusion (no, few, moderate, much)
- Pleural effusion (no, few, moderate, much)
- Thrombus no/yes

The LV parameters recorded with MRI were assessed according to the SOP for Cardiac MRI evaluation of PERFECT-Study, Rev. 2.0 SOP PERFECT Trial 11-04-2013, the SOP Late Enhancement without Gray Zone Version 1.5 UMG Radiologie MRT 2013-04-10 and the SOP Segmentation LV for Volumes and Wall Motion Version 1.2 UMG Radiologie MRT 2013-04-10 (available in the trial master file).

5.5.2.3 Secondary efficacy endpoints

Secondary efficacy end points were:

- Echocardiography: the analysis and interpretation of data from the echocardiography concerning left ventricular (LV) dimensions and functional parameters was done centrally by a reviewer blinded to the treatment the patient had received. Echocardiography parameters recorded were:
  - Date
  - Heart rate (per min)
  - quality of the images (good, middle, bad, orthograde ultrasound not measurable)
  - Left ventricular end diastolic dimension LVEDD (mm), LVEDV (cm^3), left ventricular end systolic dimension LVESD (mm), LVEF (%, four chamber view), inter ventricular septum diameter (IVSD) (mm), left ventricular posterior wall diameter (LVPWD) (mm) and LVOT (mm)
  - Main area of impaired LV infarction will be recorded in the CRF.
  - Quality assessment of wall movement (normal, akinetic, hypokinetic or dyskinetic)
  - Presence of an aneurysm.

- Pericardial effusion - no/yes, if yes: size (mm) and location (anterior, posterior, lateral, circular).
  - Mitral regurgitation (none, mild, moderate)
  - Tricuspid regurgitation (none, mild, moderate; if mild or moderate: Δ Pmax in mm/Hg)
  - Transmitral flow: early (diastolic peak flow) velocity (VE) in cm/sec, late (diastolic peak flow) velocity (VA) in cm/sec, VE/VA < 1 or >1; deceleration time (DT) in msec), velocity time integral (LVOT) in cm
  - Aortic valve regurgitation (none, mild, moderate), right ventricular end diastolic dimension (RVEDD) right ventricular outflow tract (RVOT) in mm, left atrium (LA) in mm, right atrium (RA) in mm
  - Unwanted tissue changes; no/yes; if yes, describe

- Classification of Heart Failure (NYHA)/Angina (Canadian Cardiovascular Society - CSS)
- Heart catheterization parameters: critical stenosed vessels (≥50%) recorded as: left main coronary artery (LMCA), ramus interventricular anterior (RIVA), ramus circumflex artery (RCX), right coronary artery (RCA). The LVEF (%) and area of LV infarction (septal, posterior, anterior, lateral and other) was recorded. Quality was recorded as akinetic, hypokinetic, hypokinetic to akinetic or dyskinetic.
- 6-Minute Walk Test (6MWT) – Parameters recorded were:
  - Medication taken within the last 12 hours before the test will be documented.
  - Blood pressure (mm/Hg), heart rate (beats/min)
  - Saturation of peripheral oxygen (SpO2; %),
  - Dyspnoea and fatigue assessed by Borg scale
  - Occurrence of any heart rhythm disturbances (if yes, slight to moderate or severe?)
  - Reasons for stopping or early termination of the test
  - Total distance walked within 6 minutes

- Quality of Life Questionnaires
  - Short Form Questionnaire (SF-36)
  - Minnesota Living with Heart Failure Questionnaire (MLHF-Q)
  - EQ-5D.

Figure 1 Left ventricular segmentation

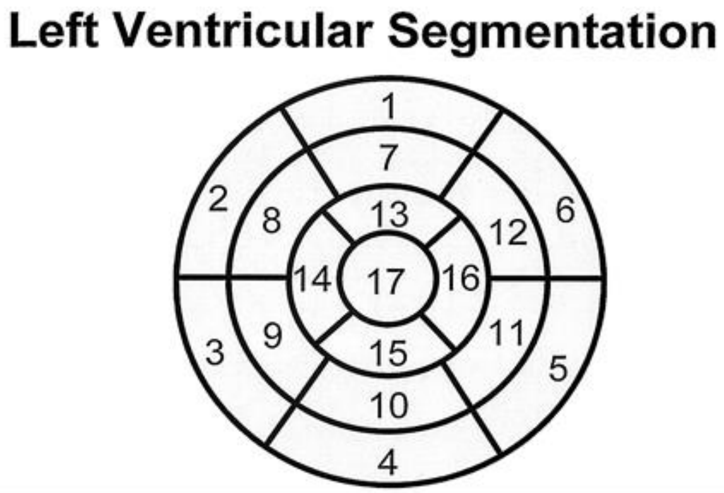

1. basal anterior	7. mid anterior	13. apical anterior
2. basal anteroseptal	8. mid anteroseptal	14. apical septal
3. basal inferoseptal	9. mid inferoseptal	15. apical inferior
4. basal inferior	10. mid inferior	16. apical lateral
5. basal inferolateral	11. mid inferolateral	17. apex
6. basal anterolateral	12. mid anterolateral	

5.5.2.4 Safety endpoints

Safety endpoints were all adverse events (AEs) as defined below:

Adverse Event (AE)

Any untoward medical occurrence in a patient or clinical investigation patient administered a pharmaceutical product and which does not necessarily have a causal relationship with this treatment is defined as an AE. An AE can therefore be any unfavourable and unintended sign (including an abnormal laboratory finding), symptom, or disease temporally associated with the use of an investigational medicinal product (IMP), whether or not related to the IMP.

Adverse Reactions (ARs)

Adverse reactions (ARs) include all untoward and unintended responses to an investigational medicinal product (IMP) related to any dose administered. All AEs judged by either the reporting Investigator or the Sponsor as having a causal relationship of possibly, probably or definitely related to the IMP qualify as ARs. An AR is defined as unexpected when its nature, severity or outcome is not consistent with the information that has been obtained from previous observations and investigational trials.

Serious Adverse Events (SAEs)

A SAE is any untoward medical occurrence that at any dose:

- results in death,
- is life-threatening (Life-threatening refers to an event in which the patient was at risk of death at the time of the event; it does not refer to an event, which, hypothetically, might have caused death if it were more serious.),
- requires inpatient hospitalization or prolongation of existing hospitalization,
- results in persistent or significant disability/incapacity,
- is associated with congenital abnormality/birth defect, or
- is another important medical event that may not be immediately life threatening or result in death or hospitalization but, based upon appropriate medical judgment are thought to jeopardize the patient or patient and/or require medical or surgical intervention to prevent one of the outcomes defining a SAE.

Serious Adverse Reactions (SARs) and Suspected Unexpected Serious Adverse Reactions (SUSARs)

An adverse reaction (AR) that meets seriousness criteria is defined as a serious adverse reaction (SAR). A suspected unexpected (unlisted) serious adverse reaction (SUSAR) is a SAR, the nature or severity of which is not consistent with the applicable product information (Investigator's brochure [IB]).

Adverse Events of Specific Interest

AV block, prolonged QT-Interval, sinus bradycardia, supraventricular arrhythmia, ventricular arrhythmia, vasovagal syncope, left ventricular failure, myocardial ischemia, cerebral ischemia, myocarditis, pericardial effusion, pericarditis or deep sternal wound infection were considered to be of specific interest for the purpose of this study and were carefully monitored.

After termination of the study, AEs not listed as AEs of specific interest were not documented unless they were possibly, probably or definitely related to the investigational medicinal product or resulted in death. In case of seriousness all possibly, probably or definitely related AEs were reported.

Major Adverse Cardiovascular Events – MACE

Major adverse cardiovascular events (patient alive no/yes, reoperation no/yes, secondary ICU admission no/yes, infarction post-OP no/yes, readmission no/yes, reintervention no/yes, new ventricular arrhythmia no/yes) were to be separately assessed.

5.5.3 Study flow chart

Figure 2 Flow chart of the study

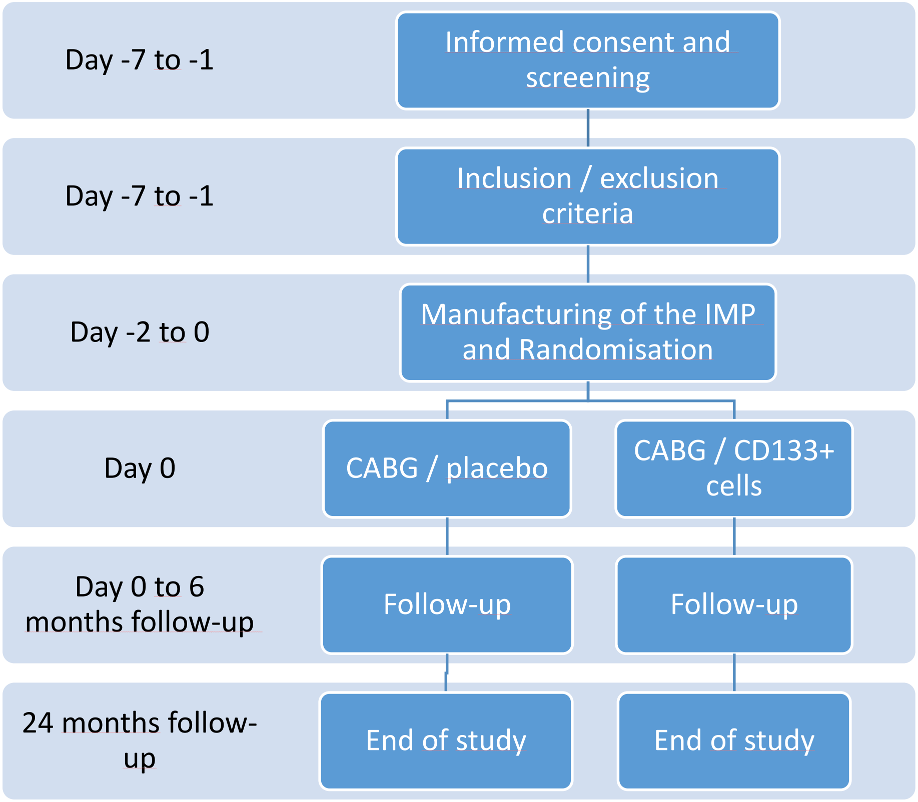

5.6 Changes in the conduct of the study

There were six amendments of the protocol that included substantial changes in the protocol during the study:

Amendment 1 dated 19 May 2009 - Change in the necessary number of cells in the product to be released. Original cell count: 5 -10 x 10^6 New cell count: 1- 10 x 10^6 (see Section 5.4.1). ATP GmbH handed over the sponsor responsibilities to the Miltenyi Biotec GmbH. The protocol was updated with respect to sponsor contact details, responsibilities and received an internal identification number (M-2006-144).

Amendment 2 dated 11 November 2009 - The inclusion criteria 25% < LVEF < 35% was changed to 25% < LVEF < 40% (see Section 5.3.1)

Amendment 3 dated 15 November 2010 – Change in the necessary number of cells in the product to be released. Previous cell count: 1 -10 x 10^6 New cell count: 0.5 - 5 x 10^6. It was decided that those patients who received the cellular product or placebo but dropped-out post-hoc because of cell count

insufficiency (less than $0.5x10^6$ but $\geq 0.1 x10^6$) were to be evaluated separately and followed-up like the intend-to-treat population.

Amendment 6 dated 17 October 2011 - The inclusion criteria 25% < LVEF < 40% was changed to 25% < LVEF < 50% (see Section 5.3.1)

Amendment 7 dated 2 July 2012 – It was decided to conduct an interim analysis based on the first 70 patients included (see Section 7.3.1)

Amendment 8 dated 16 October 2013 – The overall duration of the study was prolonged to 5.5 years and those patients that had dropped out without any treatment or due to body size/weight that prevented to reach the endpoint could be replaced.

There were two amendments (Amendment 4 - 21 December 2010, Amendment 5 - 27 April 2011) to include more study centres.

6 DATA QUALITY ASSURANCE

To ensure that study procedures conformed across all investigator sites, the protocol, case report form and safety reporting were reviewed with the investigator and his personnel responsible for the conduct of the study by the sponsor's representative(s) at the investigation site. A multi-investigator meeting was held on June 16th 2008 at the BMFZ, Biomedizinisches Forschungszentrum, Schillingallee 68, 18057 Rostock.

Adherence to the protocol requirements and verification of data generation accuracy was achieved through monitoring visits to each investigator site. All procedures were performed according to methodologies detailed in Miltenyi Biotec GmbH SOPs.

KOEHLER eClinical GmbH, Hornusstrasse 16, 79108 Freiburg a Contract Research Organisation (CRO), was employed to perform the data management according to an agreed contract. The Contract Research Organization responsibilities were conducted according to their own SOPs (available at their site).

Monitoring was provided by freelance CRAs (see Section 3)

SUSAR Reporting and Development Safety Update Report was provided by SCRATCH Pharmacovigilance GmbH and Adverse Event Reporting by Miltenyi Biotec GmbH, Dr. med. Liane Preußner.

Independent Audit statement:

An independent inspection by the Bezirksregierung Köln (Frau Mainz-Kuhlmann) took place on the 4 November 2013.

The scope of the inspection was: quality management, manufacturing of the IMP, personnel, charge release, quality control, complaint handling, and logistics.

The outcome of the inspection as stated by the Bezirksregierung Köln was: Miltenyi Biotec fulfills the criteria for the manufacturing of the IMP according to GMP. Six major findings were solved within six weeks after inspection.

7 STATISTICAL METHODS

7.1 Primary Objectives

The primary objective was to determine whether injection of autologously-derived bone marrow stem cells yields a functional benefit in addition to the CABG operation as determined by left ventricular heart function (LVEF-MRI).

Primary endpoint: LVEF at 6 months postoperatively, measured by MRI at rest and change in LVEF at 6 months post-OP compared with preoperatively (screening) and early postoperatively (discharge) as assessed by MRI. Cardiac MRI was to be established as the gold standard for determination of LV function (LVEF and LV volumes).

7.2 Secondary Objectives

The secondary objective was to determine the effects of an injection of autologously-derived bone marrow stem cells on physical exercise capacity, cardiac function, safety and QoL.

Secondary endpoints:

- Change in LVEF at 6 months post-OP compared with preoperatively (screening) and early postoperatively (discharge) as assessed by echocardiography.
- Change in LV dimensions ([LVESD], [LVEDD]) at 6-month post-OP compared with preoperatively (screening) and early postoperatively (discharge) as assessed by echocardiography.
- Change in physical exercise capacity determined by 6-minute walk test at 6 months post-OP compared with preoperatively (screening) and early postoperatively (discharge).
- Change in New York Heart Association (NYHA) and Canadian Cardiovascular Society (CCS) class at 6 months post-OP compared with preoperatively (screening) and early postoperatively (discharge).
- MACE (cardiac death, myocardial infarction, secondary intervention/reoperation, ventricular arrhythmia).
- QoL-score at 6 months post-OP compared with preoperatively (screening) and 3 months (telephone).

Secondary efficacy endpoints were to be assessed when performing the main analysis (MA).

7.3 Planned Analyses

7.3.1 Interim Analysis (IA)

An interim analysis was to be performed on the first 70 patients randomized, and followed-up for at least 6 months.

7.3.2 Main Analysis (MA)

When all patients included into the trial (planned: 142) had had their 6 Month Follow-up visit the main analysis was to be performed.

In case of stopping for futility patient recruitment was to be stopped. All patients included so far were to be followed up for safety evaluation as foreseen by the protocol. All data analysis as foreseen in the MA except the confirmatory statistical test were to be performed based on the data of those patients enrolled so far at the time point when all patients enrolled so far were followed for at least 6 months.

7.3.3 Safety-Follow-up-Analysis (SFUA)

A Safety-Follow-up-Analysis (SFUA) was to be performed at the time point when the last patient was followed up for additional 18 months and her/his 24-month visit was performed and data are included in the database.

In case of stopping the trial the SFUA was to be performed when all patients enrolled so far had their 24-month safety follow-up visit.

7.4 Sample size justification

The stratification of the primary analysis by centre was neglected in the sample size calculation. Instead of the analysis of covariance (ANCOVA) to be used in the primary analysis, the two-sample t-test scenario with equal variances was considered.

Sample size determination was done under the assumption of a two-sided type I error (α) at 5% and a type II error (β) at 10% (i.e. a power at 90%).The scenario of a difference in LVEF at month 6 post-operatively between the two treatment arms of about 4 to 5 was considered as a clinical relevant difference. With a difference of 4.5 and a standard deviation of 7.5, at least n=60 patients per group were considered necessary and with an additional 15% drop-out rate a total of at least 142 patients were to be randomized.

Sample size calculation was done using the commercial program nQuery Advisor 5.0, section 8, table MTT0-1 (14). Computation was realized using central and non-central t-distribution where the non-centrality parameter is $\sqrt{n}\ \delta/\sqrt{2}$ and δ is defined as effect size $|\mu 1-\mu 2| / \sigma$ (15).

A reassessment of the sample size was to be performed using ADDPLAN, if the trial was to be continued after the planned interim analysis on the first 70 patients randomized and followed-up for at least 6 months using the adaptive two-stage approach described by Bauer 1994 (16). The trial was to be stopped for futility or continued after reassessment of the sample size.

7.5 Populations of analysis

7.5.1 All Patients Consented Set (APCS)

All patients who consented to participate to the study were to be included into the All Patients Consented Set.

7.5.2 Full Analysis Set (FAS/ITT)

A full analysis set (FAS) following the principle of intent-to-treat (ITT) had to include every patient randomized and compare the patients per group to which they were randomly allocated, regardless of patients' compliance, or withdrawal from the study. Confirmatory analyses on primary efficacy end-point was to be performed on the FAS patients. This ITT analysis was to be considered as the primary one.

7.5.3 Safety Analysis Set (SAS)

The safety population had to comprise all patients randomized into the study and treated. Safety evaluations were to be performed on the safety population (SAS). All comparisons were to be executed per the group, to which the patients were randomized.

7.5.4 Insufficient CD133+ Analysis Set (I-CD133+-AS)

Patients who received the cellular product or Placebo but were excluded from per protocol analysis set post-hoc because of cell count insufficiency were to be evaluated separately. This “Insufficient CD133+ Analysis Set”-Population (silent drop-outs) had to include every patient with a randomization number

and a CD133+ cell count from 0.5x106> CD133+ cell count ≥ 0.1x106. All comparisons in the I-CD133+- AS Population were to be executed per the group, to which the patients were randomized.

7.5.5 Per Protocol Set (PPS)

The per protocol set (PPS) was defined as a subset of the FAS/ITT patients who were compliant with the study protocol. The PPS sample had to consist of all patients from the FAS/ITT group without any major protocol violation. A secondary efficacy analysis of the primary endpoint had to be performed based upon the PPS, to assess the sensitivity of the analysis to the choice of analysis population.

7.5.6 SAS II and SAS III

In case treatment application violations were regarded as major violations during the blinded review meeting, and it was decided to analyse safety issues separately, two additional safety analysis sets SASII and SASIII which were not foreseen in the protocol were to be created. SASII had to consist of all patients who were treated correctly with 15 injections. SASIII had to consist of all patients who were treated with more than 15 injections.

7.6 Analyses

7.6.1 Primary efficacy

The primary endpoint was LVEF at 6 months post-OP, measured by MRI at rest and was to be assessed based on the FAS/ ITT population (IA and MA). To show the sensitivity of the results the primary endpoint was to be assessed additionally based on the PPS (MA).

Regarding the inclusion criterion LVEF in screening phase, the range of LVEF was enlarged several times to provide the potential benefit to a larger patient group. Nevertheless, baseline LVEF, measured by MRI at rest, was to be considered as possible prognostic factor influencing the outcome and so the change of the inclusion criterion should not influence the analysis.

The hypotheses were:

H0: μ1<=μ2   H1: μ1>μ2,

with

μ1: The mean of LVEF at 6 months post-OP measured by MRI in the active treatment group

μ2: The mean of LVEF at 6 months post-OP measured by MRI in the placebo group

In the interim analysis including the data of the first 70 patients randomized and followed-up for at least 6 months the null hypothesis H01

H01: μ1IA<=μ2IA H11: μ1IA>μ2IA

had to be rejected if

p1 ≤ α1,

where p1 is the (one-sided) p-value from the t-test and α1 the critical value with α1=0.0102.

The trial had to stopped for futility if

p1 ≥ α0, where α0 is the stopping boundary for futility with α0=0.5.

If the trial was to be continued, reassessment of the sample size had to be performed. The sample size n2 for the second stage was to be assessed by considering observed variability and effect of the first stage.

The effect size of the primary efficacy parameter in the interim analysis will be calculated in a semi-blinded manner.

At the second stage the statistical test was to be performed based on all data of all patients after last patient's last visit, but not included into the interim analysis. The null hypothesis H02 was

H02: $\mu1 <= \mu2$ H12: $\mu1 > \mu2$,

and was to be rejected if

$p2 \leq \alpha2$,

where p2 is the (one-sided) p-value from the second t-test and $\alpha2 = c\alpha/p1$ with $c\alpha = 0.00380$.

At the end of the trial the null overall hypothesis H0 was to be rejected if

$p1 \cdot p2 \leq c\alpha$

where $c\alpha = 0.00380$ is the critical value for the combination test. (16) [Primary Efficacy Parameter]

7.6.2 Secondary efficacy

All secondary endpoint parameters were to be analysed in a strictly explorative way and performed in all respective analysis groups: FAS/ITT, PPS, I-CD133+AS during the MA. All data had to be summarized by means of descriptive statistics (mean, SD, median, minimum, maximum, number of available observations and number of missing observations) or frequency tables, stratified by treatment. To check differences between the treatment groups for the fifth secondary endpoint (MACE) an unadjusted survival analysis with Kaplan-Meier estimations had to be performed for each single kind of event using the logrank test.

7.6.3 Safety

Safety analysis was to be performed based on the data of patients in the SAS, SASII and SASIII, if applicable.

7.6.3.1 Adverse events

A general summary table of adverse events were to be provided in the MA and the SFUA:

- Number of adverse events and number and frequency of patients who experienced at least one adverse event during screening phase,
- Number of adverse events and number and frequency of patients who experienced at least one adverse event during main phase
- Number of adverse events and number and frequency of patients who experienced at least one adverse event during follow up phase (SFUA only)
- Number of adverse events and number and frequency of patients who experienced at least one adverse event during whole trial phase (SFUA only)
- Number of adverse events and number and frequency of patients who experienced at least one serious adverse event during screening phase,
- Number of adverse events and number and frequency of patients who experienced at least one serious adverse event during main phase
- Number of adverse events and number and frequency of patients who experienced at least one serious adverse event during follow up phase (SFUA only)

- Number of adverse events and number and frequency of patients who experienced at least one serious adverse event during whole trial phase (SFUA only)
- Number of adverse events and number and frequency of patients who experienced an adverse event leading to death during screening phase,
- Number of adverse events and number and frequency of patients who experienced an adverse event leading to death during main phase
- Number of adverse events and number and frequency of patients who experienced an adverse event leading to death during follow up phase (SFUA only)
- Number of adverse events and number and frequency of patients who experienced an adverse event leading to death during whole trial phase (SFUA only)

AEs and SAEs were to be summarized overall (frequency tables) by occurrence, worst severity, outcome and causal relationship to treatment and were to be descriptively compared between the two treatment groups using Fisher's Exact Test. AE tables present the total number of patients reporting at least one specific event and the maximum severity grade (in case patients reported more than one episode of the same event, they were to be counted only once considering the maximum severity and strongest causality).

Special tables were to be displayed for MACE, AEs of specific interest and for AEs leading to death or leading to withdrawal due to any reason in MA and SFUA.

Separate summarizations of SAEs and AEs that were likely, probable and definitely related to treatment (ARs and SARs, respectively) and AEs that are unlikely and not related to treatment were to be provided in MA and SFUA.

SAEs and AEs which occurred during screening phase since the MRI and the harvesting of bone marrow is an interventional procedure, were to be analysed based on the data of all patients who gave consent and attended the screening procedure (SAS + FAS/ITT together).

All AEs were to be listed (MA and SFUA).

7.6.3.2 Adverse Events of Specific Interest (AESI)

Separate tables were to be provided for AEs of specific interest in MA and SFUA.

For each kind of AESI the rate of events and the rate of patients who experienced the event at least once by treatment group were to be provided for events occurred

- during the first 6 months of follow up (until (including) visit V (to be presented in MA),
- during the following 18 months (long term follow up beginning after visit V until visit month 24, (to be presented in the SFUA) and
- during the time since surgery until visit month 24 (to be presented in the SFUA).

7.6.3.3 MACE

For each kind of MACE, the rate of events and the rate of patients who experienced the adverse event at least one time had to be provided in MA and SFUA for events occurred

- during the first 6 Months of follow up (until (including) visit V, in MA),
- during the following 18 months (long term follow up beginning after visit V until visit month 24, in SFUA and
- during the time since surgery until visit month 24 (SFUA).

7.6.3.4 Laboratory Values

In MA and SFUA descriptive statistics for laboratory parameters were to be presented by treatment group and time point. For continuous laboratory parameters, changes from baseline to the other time points were to be presented by treatment group and descriptive statistics were to be calculated. Values clinically significant outside corresponding normal ranges and therefore reported as adverse event were to be displayed and tabulated together with adverse events.

The rate of new or worsened abnormalities were to be presented as well as the rate of abnormal values. A new abnormality is defined as an out-of-range value that was previously normal or a value that is initially out of range in one direction (either low or high) and becomes out of-range in the opposite direction at the end of the period. A worsened abnormality is a value that was out of range at the start and became more abnormal during the period. More abnormal means a higher Common Terminology Criteria grade recorded in the AE section of the CRF.

7.6.3.5 Unwanted Tissue Changes

In MA and SFUA the rate of patients by treatment group were to be provided,

- who developed a tumour during the first 6 Months of follow up (until (including) visit V (to be presented in MA),
- who developed a tumour during the following 18 months (long term follow up beginning after visit V until visit month 24, in SFUA only) and
- who developed a tumour during the time since surgery until visit month 24 (SFUA only)

7.6.3.6 Physical Examination, Vital Signs and 12-lead electrocardiogram (ECG)

In MA and SFUA changes in vital signs, general physical examination and 12-lead ECG parameters as well as the occurrence of unwanted tissue changes were to be analysed for treatment group differences.

7.6.4 Questionnaires

All QoL questions from the questionnaires EuroQol five dimensions questionnaire (EQ-5D), MLHF-Q and SF-36 were to be analysed in a strictly explorative way. All data was to be summarized by means of descriptive statistics (mean, SD, median, minimum, maximum, number of available observations and number of missing observations) or frequency tables, stratified by treatment.

7.6.5 Disposition of Patients

The following data were to be listed and presented according to the Statistical Analysis Plan (SAP):

- Demographics and baseline characteristics
- Concomitant medication and medical history
- Heart catheterization results

7.7 Changes in planned analyses

Patient recruitment was stopped on 12th November 2015 when 82 (40 in the test product group, 40 in the placebo group, and 2 randomized but no treated) instead of the planned 142 evaluable patients were included in the trial. Due to this lower number of patients several changes to the planned analysis were considered.

The most relevant change was:

Efficacy analysis

The trial was conducted at six study sites. Since there were more than 20 patients recruited in only two centres and the other centres recruited less than 10 patients each, it was decided not do a per centre analysis.

Other changes in the planned analysis can be found in Appendix A8 - Statistical Analysis Plan.

8 INTERIM ANALYSES

An interim efficacy analysis was performed on the FAS/ITT analysis population. Certain demographic and baseline parameters were presented in summary or frequency tables as specified below as well as reasons for exclusion from SAS. Besides the evaluation of the primary endpoint the following secondary endpoints were evaluated based on the FAS/ITT population:

- Change in LVEF at 6 months post-OP compared with preoperatively (screening) assessed by echocardiography
- Change in physical exercise capacity determined by 6-minute walk test at 6 months compared with preoperatively
- Change in NYHA and CCS class at 6 months post-OP compared with preoperatively
- MACE (cardiac death, myocardial infarction, secondary intervention/reoperation, ventricular arrhythmia).

Results of the interim analysis

In total, 35 patients were treated with the test product and 35 patients were treated with placebo.

The interim primary efficacy analysis (LVEF at Visit V) indicated that there was a statistically significant difference between the treatment group and the placebo group (mean LVEFs 40.44 vs. 44.94; p=0.026).

The interim efficacy analysis of the secondary endpoints LVEF-, 6MWT-, NYHA- and CCS-change did not show remarkable group differences.

It was decided to continue the study until completion of enrolment.

9 STUDY PATIENTS

9.1 Patient disposition

Figure 4 Number of patients included in the study and randomized

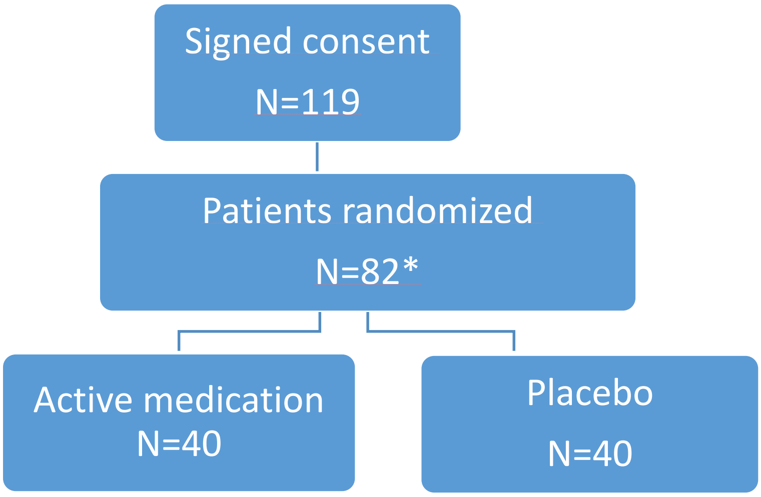

***2 patients were randomized but did not received treatment**

A total of 119 patients were screened in 6 centres. All patients signed the informed consent form and were included in the study. Eighty-two (82) patients were randomized to active treatment or placebo. Forty 40 (48.8%) received an injection of CD133+ cells and 40 (48.8%) received an injection of placebo after the CABG OP. Two (2) patients were randomized but not treated because the CD 133+ preparation did not comply with the release criteria for GMP. The three largest enrolling centres were Centre 2 – MH Hannover (40 Patients, 34%), Centre 3 – Universität Rostock (38 Patients, 32%) and Centre 5 – Herzzentrum Leipzig (15 Patients, 13%).

At the time of database lock for the final analysis 30 (75.0%) patients in the placebo group and 28 (70.0%) patients in the CD133+ treatment group had completed Assessment V (6-months after CABG operation) per protocol. One (2.5%) patients in the placebo arm and 6 (5.0%) patients in the CD133+ treatment group had withdrawn from the study mainly due to adverse events (reported 5 times) and other reasons (reported 7 times).

A summary of the patients' disposition of all patients enrolled in the study is provided in Table DIS03T_FAS and Listing DIS03L_FAS (Appendices B and C).

9.2 Protocol deviations and violations

Listings of patients with inclusion/exclusion and protocol violations is provided in Listing ANA06L_ALL.rtf (Appendix C)

9.3 Study populations analysed

As described in the protocol and the SAP, the main analysis populations were the PPS (N=58), SAS (N=77), the FAS/ITT (N=82) and the APCS (N=119). Additionally, the SASII)(N=42) and SASIII (N=35) were derived from the SAS and included patients who received exactly 15 injection or more than 15 injections of the product (Placebo or CD133+ cells) respectively. The patients from the FAS/ITT treated with an insufficient cell count were included in the Insufficient CD133+ Analysis Set (I-CD133+-AS) (N=3). The detailed definition of the analysis populations can be found in the SAP paragraph 4.4.

The primary efficacy analysis was performed for the FAS/ITT and the PPS, the secondary efficacy analysis for FAS/ITT, PPS and I-CD133+-AS.

After a careful review of the blinded data in a blind data review meeting conducted on the 20 May 2016 a total of 19 patients were excluded from the FAS/ITT due to protocol violations. The reason for the exclusion of these patients is further detailed in Listing 0105_ANA06L_ALL.rtf (Appendix C). A definition of the protocol violations leading to exclusion of analysis populations can be found in the SAP paragraph 4.3.5.

The allocation of patients to the different analysis sets is shown in Figure 3.

Figure 3 Number of patients in each analysis set

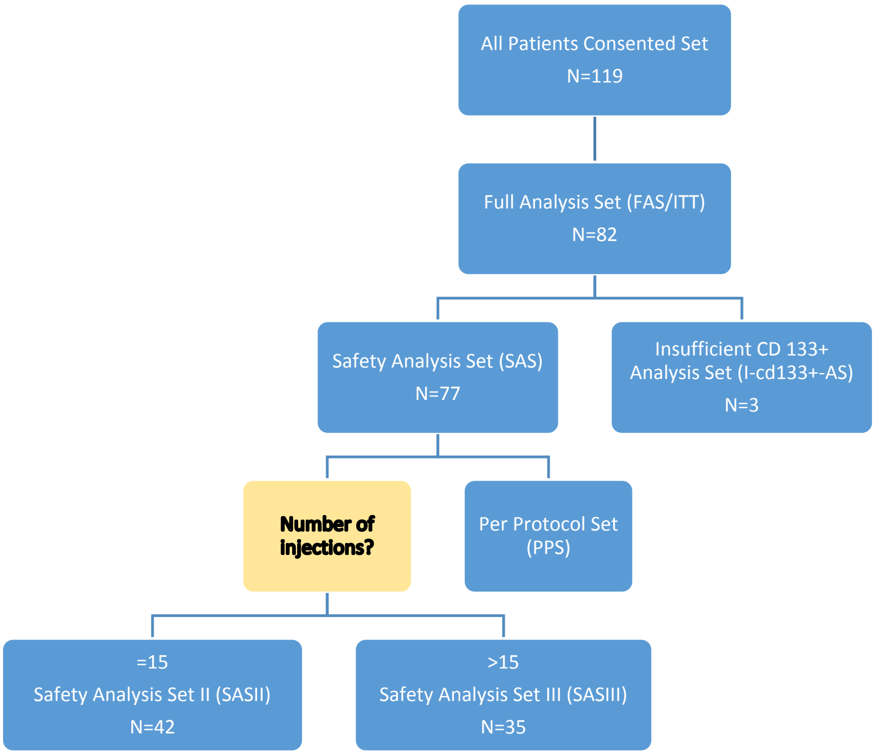

9.4 Demographic and baseline characteristics

Most of the patients were males, with a mean age of 62-63 years in both groups. The demographic and baseline characteristics of the patients show that both groups were comparable. Table 3 shows age, sex and body weight of the patients included in the study.

Table 3 Demographics of the patients included in the study.

	Placebo	CD133+	Not treated	Total
Age [years]				
Mean	62.9	63.0	56.0	62.8
Std	8.49	8.72	15.56	8.69
Median	61.5	64.5	56.0	62.5
Q1 - Q3	58 - 69	59 - 70	45 - 67	58 - 69
Min - Max	35 - 79	34 - 78	45 - 67	34 - 79
N	40	40	2	82
Sex				
Female	6 (5%)	5 (13%)	-	11 (13%)
Male	34 (85%)	35 (88%)	2 (100%)	71 (87%)
Body Weight [kg]				
Mean	90.4	87.1	-	88.8
Std	14.55	16.36	-	15.43
Median	91.5	87.0	-	90.0
Q1 - Q3	82 - 102	76 - 98	-	77 - 100
Min - Max	59 - 117	50 - 123	-	50 - 123

Note: Denominator for percentages is column N.

9.4.1 Medical History

Medical history findings were registered in three overall categories: general medical history, cardiac medical history and risk factors.

General medical history was collected in 9 categories. The most frequently reported categories were “other diseases” (95% of the total population, 93% vs 98% of patients who received CD133+ and placebo, respectively), “abdomen” (29% of the total population, 28% vs 33% of the patients, respectively) and “blood and hematopoietic system” (28% of the total population, 25% vs 30% of the patients, respectively). For more details, see Table MEDHIS01T_FAS and Listing MEDHIS02L_FAS (Appendices B and C).

Cardiac medical history was collected in several categories. The most frequently reported categories were “myocardial ischemia” (90% of the total population, 90% vs 92% of the patients who received CD133+ or placebo respectively) and “left ventricular failure” (57% of the total population, 52% vs 60% of the patients, respectively). For more details, see Tables MEDHIS03T_FAS (Appendix B).

Risk factors were also collected in several categories. The most frequently reported categories were “family disposition: arterial hypertension” (31.7% of the total population, 27.5% vs 37.5% of the patients respectively), “family disposition: diabetes mellitus” (31.7% of the total population, 30.0% vs 35.0% of the patients respectively) and “prior PCI and/or stent” (28.0% of the total population, 22.5% vs 35.0% of the patients respectively). Additionally, 70.7% of the total population (72.5% vs. 70.0% of the patients

respectively) had smoked previously or was smoking at the time of the study. For more details, see Table MEDHIS05T_FAS (Appendix B)

9.4.2 Previous and Concomitant Medications

Concomitant medication was coded using the WHO Drug Dictionary version from March 2014. Twelve categories of concomitant medications of special interest were defined based on the Anatomical Therapeutic Chemical class. The most frequently used concomitant medications of special interest were Beta blockers (99% of the total population, 100% vs 98% of the patients who received CD133+ and placebo respectively), ASS (99% of the population, 97% vs 100% of the patients respectively) and diuretics (95% of the total population, 95% vs 95% of the patients respectively).

Table 4 Concomitant medication of specific interest

	**Placebo**	**CD133+**	**Total**
ASS	40 (100%)	36 (97%)	76 (99%)
Beta blocker	39 (98%)	37 (100%)	76 (99%)
CSE inhibitor	38 (95%)	37 (100%)	75 (97%)
Diuretic	38 (95%)	35 (95%)	73 (95%)
ACE inhibitor	33 (83%)	31 (84%)	64 (83%)
Aldosteron antagonist	23 (58%)	21 (57%)	44 (57%)
Ca antagonist	13 (33%)	16 (43%)	29 (38%)
Nitrate	12 (30%)	10 (27%)	22 (29%)
Antiarrhythmic other	12 (30%)	8 (22%)	20 (26%)
ATII receptor antagonist	9 (23%)	8 (22%)	17 (22%)
Marcumar	5 (13%)	7 (19%)	12 (16%)
Digitalis	4 (10%)	6 (16%)	10 (13%)

Note: Denominator for percentages is column N.

Source: P132_perfect - CONCMED01T.sas [SVN:28622] Data Extract: 15JUL2016 Generation Date : 09AUG2016 11:33

10 EXTENT OF EXPOSURE

In the context of this study it is not possible to define an extent of exposure since the time and mount of CD133+ autologous bone marrow stem cells that are active/alive is unknown.

10.1 Dosage and duration

In this study, patients were treated with injections (15 or more) of CD133+ cells preparation or placebo during CABG surgery. All patients underwent bone marrow aspiration (150-200 mL) and a withdrawal of 20 mL of blood prior to CABG surgery.

The CD133+ cell preparation consisted in 5 mL of CD133+ cells (0,5-5x106 cells) suspended in physiological saline and 10% of autologous serum.

The placebo consisted in 5 mL of physiological saline and 10% of autologous serum.

The product (CD133+ cells or placebo) was injected intramyocardially (divided in 15 injections or more) during CABG surgery.

10.2 Compliance

Not applicable.

11 EFFICACY ANALYSES

Efficacy analysis was performed per SAP V1.0 and to its amendment dated 02.05.2016 (See Appendix A 8 – Statistical Analysis Plan).

11.1 Primary efficacy analysis

The primary outcome measure for this study was the LVEF at 6 months post-OP, measured by MRI at rest. Including:

- Change in LVEF at 6 months post-OP compared with preoperatively (screening) and early postoperatively (discharge) assessed by cardiac MRI scans. The data are summarized in the following tables in Appendix B: 0309_MRI08T_FAS.rtf and 0318_ECHO09T_FAS.rtf
- Change in LV dimensions ((LVESD), left ventricular diastolic dimension (LVEDD)) at 6 months post-OP compared with preoperatively (screening) and early postoperatively (discharge) as assessed by echocardiography. The data are summarized in Appendix B: 0319_ECHO10T_FAS

The analysis populations for the primary endpoint were the FAS/ITT containing all the randomized patients and the PPS.

Sixty-four (64) patients (34 in the placebo and 30 in the test product group) of the FAS/ITT had a LVEF value measured with MRI at Visit V and were included in the primary efficacy analysis.

Fifty-eight (58) patients (30 in the placebo and 28 in the test product group) of the PPS had a LVEF value measured with MRI at Visit V and were included in the primary efficacy analysis.

11.1.1 Results

The LVEF data are summarized by treatment and visit descriptively (Appendix B: Table 0302_MRI01T_FAS.rtf). The continuous primary efficacy variable LVEF at 6 months post-OP was analysed using analysis of covariance (ANCOVA) adjusting for the covariates treatment, study site and baseline LVEF with regards to possible baseline and study side effects.

The overall results of the ANCOVA for the FAS/ITT and PPS are presented in Table 5. The only statistically significant covariate was the LVEF at baseline with a p-value of 0.0205 for the FAS/ITT and 0.0308 for the PPS.

Table 5 Overall results of the ANCOVA for LVEF at 6 months in the FAS/ITT and PPS populations

**FAS/ITT**					
**Parameter**	**DF**	**Type III SS**	**Mean Square**	**F value**	**p-value**
Treatment	1	4.0861461	4.0861461	0.03	0.8581
Center	3	493.5374425	164.5124808	1.30	0.2855
LVEF at Baseline	1	728.4601006	728.4601006	5.76	0.0205
Treatment * Center	2	77.7836378	38.8918189	0.31	0.7366
Treatment * LVEF at Baseline	1	6.7541545	6.7541545	0.05	0.8182
Center * LVEF at Baseline	3	504.7373428	168.2457809	1.33	0.2760
Treatment * Center * LVEF at Baseline	2	103.6547991	51.8273996	0.41	0.6660
Note: 18 patients (22%) excluded from analysis due to missing values.					
**PPS**					
**Parameter**	**DF**	**Type III SS**	**Mean Square**	**F value**	**p-value**
Treatment	1	1.7327535	1.7327535	0.01	0.9108
Center	3	480.4722983	160.1574328	1.17	0.3318
LVEF at Baseline	1	685.1261451	685.1261451	5.02	0.0308
Treatment * Center	2	95.7113171	47.8556585	0.35	0.7062
Treatment * LVEF at Baseline	1	2.6900177	2.6900177	0.02	0.8890
Center * LVEF at Baseline	3	474.0604457	158.0201486	1.16	0.3377
Treatment * Center * LVEF at Baseline	2	123.1749751	61.5874875	0.45	0.6398
Note: 0 patients (0%) excluded from analysis due to missing values.					

Source: P132_perfect - EFF01T.sas [SVN:23809] Data Extract: 15JUL2016 Generation Date : 09AUG2016 11:16 Appendix 3: 0301_EFF01T_FAS.rtf and P132_perfect - EFF01T.sas [SVN:23809] Data Extract: 15JUL2016 Generation Date : 09AUG2016 11:16 Appendix 3: 03011_EFF01T_PPROT.rtf

Due to the large number of missing values for LVEF assessed by MRI scan at 6 months post-OP (18 patients, 22% of the FAS/ITT population) and at discharge (visit III, 49 patients, 60% of the FAS/ITT population), an additional analysis was made using a mixed model analysis for repeat measures approach (MMRM) in order to compensate possible artefacts due to incomplete data groups. The change from baseline at Visit III (post-OP) and at Visit V (6 months post-OP) for the FAS/ITT and PPS are presented in Table 6. Additionally, a plot of the evolution of the mean of the LVEF is presented on Figure 4.

This was the approach used for the interim analysis as well.

Table 6 Overall results of the ANCOVA for LVEF in the FAS/ITT and PPS - MMRM

FAS/ITT	Estimated BL LVEF	DF	Estimate	Standard-error	95% CI	p-Value
**Visit III**						
Average change from Baseline						
Placebo	33.93	35	37.0086	2.1182	[32.7; 41.3]	
CD133+	33.93	35.2	37.7163	2.1220	[33.4; 42.0]	
Difference in Treatment Groups						
CD133+ - Placebo		35.8	0.7077	2.9691	[-5.3; 6.7]	0.8130
**Visit V**						
Average change from Baseline						
Placebo	33.93	58.6	42.4145	1.9638	[38.5; 46.3]	
CD133+	33.93	57.7	44.6347	2.1589	[40.3; 49.0]	
Difference in Treatment Groups						
CD133+ - Placebo		57.5	2.2202	2.8895	[-3.6; 8.0]	0.4454
**PPS**	**Estimated BL LVEF**	**DF**	**Estimate**	**Standard-error**	**95% CI**	**p-Value**
**Visit III**						
Average change from Baseline						
Placebo	33.69	31.1	36.4312	2.2885	[31.8 ; 41.1]	
CD133+	33.69	31.1	37.7676	2.2771	[33.1 ; 42.4]	
Difference in Treatment Groups						
CD133+ - Placebo		31.1	1.3364	3.1786	[-5.1 ; 7.8]	0.6771
**Visit V**						
Average change from Baseline						
Placebo	33.69	50	41.9530	2.1684	[37.6 ; 46.3]	
CD133+	33.69	50	44.4923	2.2577	[40.0 ; 49.0]	
Difference in Treatment Groups						
CD133+ - Placebo		50	2.5393	3.1643	[-3.8 ; 8.9]	0.4261

DF=Degree of Freedom, CI=Confidence Interval

Source: P132_perfect - EFF02T.sas [SVN:LOCAL PROGRAM] Data Extract: 15JUL2016 Generation Date : 10AUG2016 21:02

With p-values of 0,8130 and 0,4454 for Visit III and Visit V respectively in the FAS/ITT and p-values of 0,6771 and 0,4261 for Visit III and Visit V respectively in the PPS, the difference between the treatments groups is not statistically significant.

Figure 4 Evolution of the mean of LVEF for the different treatment groups in the FAS/ITT

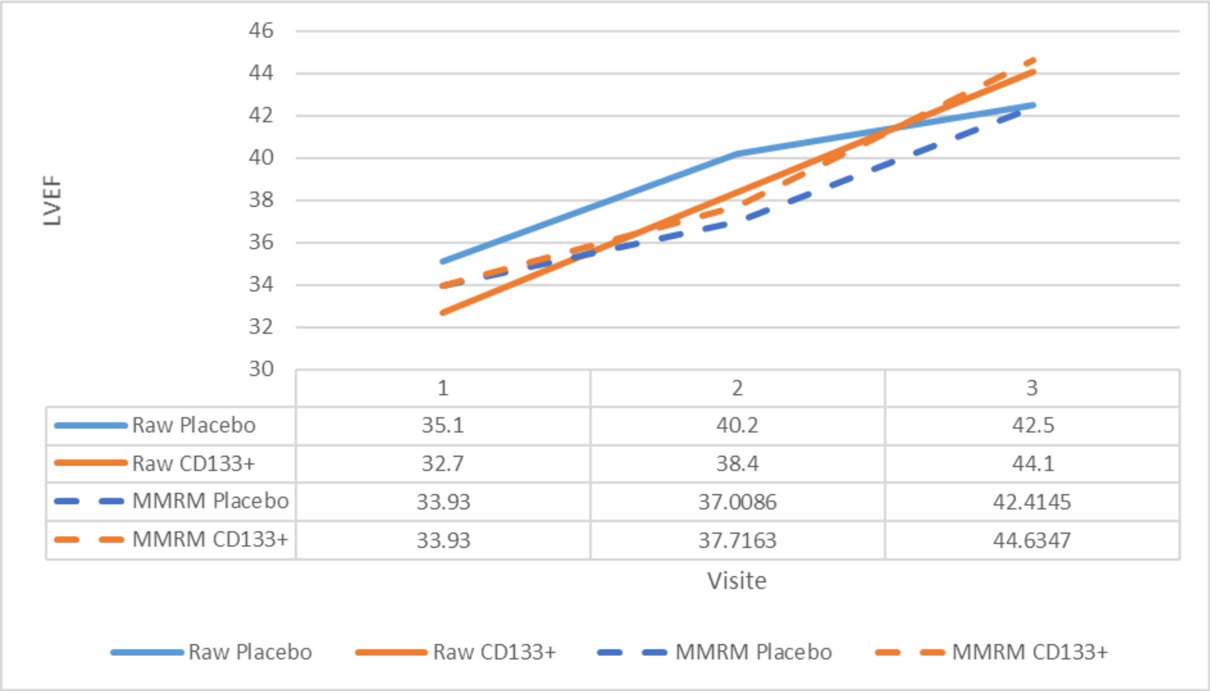

Summary statistics of the change in LVEF assessed by MRI scan at 6 months post-OP compared with preoperatively (Visit I) and early postoperatively (Visit III) in the FAS/ITT and the PPS are presented in Table 7.

Table 7 Summary statistics of the change in LVEF assessed by MRI in the FAS/ITT and PPS

**FAS/ITT**	**Placebo**	**CD133+**	**Not treated**	**Total**
Difference between Visit V (6-Month Follow-Up) and Visit I (Screening)				
N[a]	34	30	0	64
Mean[b]	8.0	11.4	-	9.6
Std	8.71	13.52	-	11.26
Median	7.0	10.5	-	8.0
Q1 - Q3	2 - 12	1 - 20	-	2 - 17
Min - Max	-6 - 32	-13 - 42	-	-13 - 42
Difference between Visit V (6-Month Follow-Up) and Visit III (Hospital Discharge)				
N[a]	16	14	0	30
Mean[c]	4.1	8.8	-	6.3
Std	8.57	6.38	-	7.87
Median	2.0	8.0	-	7.0
Q1 - Q3	-1 - 10	4 - 10	-	1 - 10
Min - Max	-11 - 23	1 - 21	-	-11 - 23

**PPS**	**Placebo**	**CD133+**	**Total**
Difference between Visit V (6-Month Follow-Up) and Visit I (Screening)			
N[a]	30	28	58
Mean[b]	7.9	11.4	9.6
Std	9.05	13.75	11.59
Median	7.0	10.5	8.0
Q1 - Q3	2 - 12	2 - 20	2 - 16
Min - Max	-6 - 32	-13 - 42	-13 - 42
Difference between Visit V (6-Month Follow-Up) and Visit III (Hospital Discharge)			
N[a]	15	14	29
Mean[c]	4.3	8.8	6.5
Std	8.80	6.38	7.92
Median	3.0	8.0	8.0
Q1 - Q3	-1 - 10	4 - 10	1 - 10
Min - Max	-11 - 23	1 - 21	-11 - 23

[a]Number of measurements
[b]The difference was calculated as $Value_{6months}$ – $Value_{VI}$, therefore a positive mean value represents an increase of the LVEF
[c]The difference was calculated as $Value_{6months}$ – $Value_{VIII}$, therefore a positive mean value represents an increase of the LVEF
Source: P132_perfect - MRI08T.sas [SVN:27200] Data Extract: 15JUL2016 Generation Date : 09AUG2016 11:17 Appendix 3: 0309_MRI08T_FAS.rtf and P132_perfect - MRI08T.sas [SVN:27200] Data Extract: 15JUL2016 Generation Date : 09AUG2016 11:20

**FAS/ITT**	**Placebo**	**CD133+**	**Not treated**	**Total**
Visit I (Screening)				
N	39	40	2	81
Mean	35.1	32.7	30.0	33.7
Std	6.33	5.25	5.66	5.89

FAS/ITT	Placebo	CD133+	Not treated	Total
Median	35.0	32.0	30.0	34.0
Q1 - Q3	29 - 39	28 - 36	26 - 34	29 - 37
Min - Max	25 - 49	25 - 48	26 - 34	25 - 49
Visit V (6-Month Follow-Up)				
N	34	30	0	64
Mean	42.5	44.1	-	43.2
Std	9.65	13.78	-	11.70
Median	45.5	42.5	-	44.0
Q1 - Q3	33 - 50	36 - 53	-	34 - 51
Min - Max	25 - 61	19 - 74	-	19 - 74

PPS	Placebo	CD133+	Total
Visit I (Screening)			
N	30	28	58
Mean	34.4	32.5	33.5
Std	6.46	5.98	6.26
Median	35.0	32.0	32.5
Q1 - Q3	29 - 38	28 - 37	28 - 37
Min - Max	25 - 49	25 - 48	25 - 49
Visit V (6-Month Follow-Up)			
N	30	28	58
Mean	42.3	43.9	43.1
Std	9.95	13.89	11.93
Median	44.0	42.5	42.5
Q1 - Q3	33 - 50	37 - 52	34 - 51
Min - Max	25 - 61	19 - 74	19 - 74

Source: P132_perfect - MRI01T.sas [SVN:28096] Data Extract: 15JUL2016 Generation Date : 09AUG2016 11:19

Table 9 to Table 13 show the LV parameters measured with MRI for the FAS/IIT and the PPS.

Table 8 LVEF summary statistics by visit and by treatment group assessed with MRI in the FAS/ITT and the PPS

FAS/ITT	Placebo	CD133+	Not treated	Total
Visit I (Screening)				
N	39	40	2	81
Mean	35.1	32.7	30.0	33.7
Std	6.33	5.25	5.66	5.89
Median	35.0	32.0	30.0	34.0
Q1 - Q3	29 - 39	28 - 36	26 - 34	29 - 37
Min - Max	25 - 49	25 - 48	26 - 34	25 - 49
Visit V (6-Month Follow-Up)				
N	34	30	0	64
Mean	42.5	44.1	-	43.2
Std	9.65	13.78	-	11.70

FAS/ITT	Placebo	CD133+	Not treated	Total
Median	45.5	42.5	-	44.0
Q1 - Q3	33 - 50	36 - 53	-	34 - 51
Min - Max	25 - 61	19 - 74	-	19 - 74

PPS	Placebo	CD133+	Total
Visit I (Screening)			
N	30	28	58
Mean	34.4	32.5	33.5
Std	6.46	5.98	6.26
Median	35.0	32.0	32.5
Q1 - Q3	29 - 38	28 - 37	28 - 37
Min - Max	25 - 49	25 - 48	25 - 49
Visit V (6-Month Follow-Up)			
N	30	28	58
Mean	42.3	43.9	43.1
Std	9.95	13.89	11.93
Median	44.0	42.5	42.5
Q1 - Q3	33 - 50	37 - 52	34 - 51
Min - Max	25 - 61	19 - 74	19 - 74

Source: P132_perfect - MRI01T.sas [SVN:28096] Data Extract: 15JUL2016 Generation Date : 09AUG2016 11:19

Table 9 LV mass assessed with MRI in the FAS/ITT and the PPS

FAS/ITT		Placebo	CD133+	Not treated	Total
Visit I (Screening)	N	39	39	2	80
	Mean	179.4	182.6	273.0	183.3
	Std	42.53	43.14	43.84	44.74
	Median	178.0	186.0	273.0	179.5
	Q1 - Q3	146 - 198	142 - 208	242 - 304	146 - 209
	Min - Max	104 - 287	1 - 274	242 - 304	101 - 304
Visit III (Hospital Discharge)	N	17	16	0	33
	Mean	178.4	185.6	-	181.9
	Std	54.08	46.79	-	50.02
	Median	178.0	191.5	-	189.0
	Q1 - Q3	139 - 195	155 - 200	-	141 - 200
	Min - Max	110 - 322	93 - 277	-	93 - 322
Visit V (6-Month Follow-Up)	N	34	30	0	64
	Mean	173.8	170.1	-	172.1
	Std	44.54	32.74	-	39.20
	Median	174.0	173.5	-	174.0
	Q1 - Q3	152 - 188	145 - 192	-	149 - 190
	Min - Max	104 - 331	109 - 229	-	104 - 331

PPS		Placebo	CD133+	Total
Visit I (Screening)	N	30	28	58
	Mean	184.4	183.5	183.9
	Std	44.39	36.65	40.49
	Median	182.5	186.0	185.5
	Q1 - Q3	146 - 210	161 - 200	147 - 202
	Min - Max	104 - 287	122 - 270	104 - 287
Visit III (Hospital Discharge)	N	15	14	29
	Mean	178.6	178.9	178.7
	Std	57.81	42.82	50.22
	Median	186.0	191.5	190.0
	Q1 - Q3	129 - 221	150 - 200	139 - 200
	Min - Max	110 - 322	93 - 250	93 - 322
Visit V (6-Month Follow-Up)	N	30	28	58
	Mean	173.9	171.0	172.5
	Std	47.11	33.57	40.81
	Median	174.0	178.5	176.5
	Q1 - Q3	143 - 188	147 - 194	143 - 192
	Min - Max	104 - 331	109 - 229	104 - 331

Source: P132_perfect - MRI02T.sas [SVN:28096] Data Extract: 15JUL2016 Generation Date : 09AUG2016 11:19

Table 10 LVEDV assessed with MRI in the FAS/ITT and the PPS

FAS/ITT		Placebo	CD133+	Not treated	Total
Visit I (Screening)	N	39	39	2	80
	Mean	105.7	112.4	145.5	110.0
	Std	26.32	35.65	47.38	31.88
	Median	107.0	109.0	145.5	108.5
	Q1 - Q3	88 - 121	93 - 127	112 - 179	92 - 123
	Min - Max	45 - 176	41 - 198	112 - 179	41 - 198
Visit III (Hospital Discharge)	N	17	16	0	33
	Mean	104.9	119.9	-	112.2
	Std	21.46	50.00	-	38.20
	Median	108.0	106.5	-	108.0
	Q1 - Q3	87 - 122	86 - 150	-	87 - 127
	Min - Max	66 - 139	52 - 247	-	52 - 247
Visit V (6-Month Follow-Up)	N	34	30	0	64
	Mean	102.6	104.7	-	103.6
	Std	24.13	44.07	-	34.64
	Median	99.5	101.5	-	100.5
	Q1 - Q3	87 - 123	72 - 114	-	83 - 117
	Min - Max	60 - 157	47 - 249	-	47 - 249

PPS		Placebo	CD133+	Total)
Visit I (Screening)	N	30	28	58
	Mean	107.4	106.8	107.1
	Std	26.44	32.61	29.32
	Median	108.5	103.5	107.5
	Q1 - Q3	92 - 121	92 - 125	92 - 122
	Min - Max	45 - 176	41 - 194	41 - 194
Visit III (Hospital Discharge)	N	15	14	29
	Mean	103.7	115.6	109.4
	Std	21.38	52.13	39.08
	Median	108.0	103.0	105.0
	Q1 - Q3	85 - 122	83 - 127	85 - 122
	Min - Max	66 - 139	52 - 247	52 - 247
Visit V (6-Month Follow-Up)	N	30	28	58
	Mean	101.0	105.9	103.3
	Std	23.23	44.99	35.21
	Median	99.0	101.5	100.5
	Q1 - Q3	83 - 123	77 - 115	82 - 115
	Min - Max	60 - 151	47 - 249	47 - 249

Source: P132_perfect - MRI02T.sas [SVN:28096] Data Extract: 15JUL2016 Generation Date : 09AUG2016 11:19

Table 11 LVESV assessed with MRI in the FAS/ITT and the PPS

FAS/ITT		Placebo	CD133+	Not treated	Total)
Visit I (Screening)	N	39	39	2	80
	Mean	69.1	75.5	102.0	73.0
	Std	19.34	25.59	41.01	23.41
	Median	69.0	75.0	102.0	72.0
	Q1 - Q3	53 - 85	60 - 85	73 - 131	58 - 85
	Min - Max	31 - 110	21 - 141	73 - 131	21 - 141
Visit III (Hospital Discharge)	N	17	16	0	33
	Mean	62.9	76.6	-	69.5
	Std	20.60	40.01	-	31.79
	Median	59.0	63.5	-	60.0
	Q1 - Q3	49 - 79	53 - 107	-	51 - 87
	Min - Max	31 - 96	14 - 160	-	14 - 160
Visit V (6-Month Follow-Up)	N	34	30	0	64
	Mean	59.9	60.8	-	60.3
	Std	21.98	33.76	-	27.89
	Median	54.0	59.0	-	55.5
	Q1 - Q3	43 - 72	33 - 77	-	43 - 75
	Min - Max	28 - 112	13 - 144	-	13 - 144

PPS		Placebo	CD133+	Total
Visit I (Screening)	N	30	28	58
	Mean	71.2	71.9	71.5
	Std	19.80	23.76	21.61
	Median	71.0	73.0	71.5
	Q1 - Q3	61 - 85	58 - 81	58 - 84
	Min - Max	31 - 110	21 - 141	21 - 141
Visit III (Hospital Discharge)	N	15	14	29
	Mean	63.7	71.9	67.6
	Std	21.55	40.59	31.85
	Median	59.0	59.5	59.0
	Q1 - Q3	49 - 87	51 - 83	51 - 83
	Min - Max	31 - 96	14 - 160	14 - 160
Visit V (6-Month Follow-Up)	N	30	28	58
	Mean	58.9	61.5	60.2
	Std	22.18	34.28	28.44
	Median	52.5	59.0	54.0
	Q1 - Q3	43 - 72	36 - 77	42 - 77
	Min - Max	28 - 112	13 - 144	13 - 144

Source: P132_perfect - MRI02T.sas [SVN:28096] Data Extract: 15JUL2016 Generation Date : 09AUG2016 11:19

Table 12 Total scar tissue measured with MRI in the FAS/ITT and the PPS

FAS/ITT		Placebo	CD133+	Not treated	Total
Visit I (Screening)	N	37	34	2	73
	Mean	32.3	29.9	42.5	31.5
	Std	12.60	18.59	4.95	15.59
	Median	32.0	28.0	42.5	30.0
	Q1 - Q3	23 - 41	18 - 34	39 - 46	20 - 41
	Min - Max	2 - 56	4 - 89	39 - 46	2 - 89
Visit III (Hospital Discharge)	N	17	14	0	31
	Mean	34.6	31.2	-	33.1
	Std	14.21	19.08	-	16.38
	Median	31.0	30.5	-	31.0
	Q1 - Q3	26 - 39	20 - 46	-	20 - 44
	Min - Max	18 - 73	0 - 72	-	0 - 73
Visit V (6-Month Follow-Up)	N	33	27	0	60
	Mean	37.3	27.0	-	32.7
	Std	14.01	13.19	-	14.48
	Median	36.0	28.0	-	31.0
	Q1 - Q3	29 - 44	22 - 34	-	25 - 41
	Min - Max	16 - 69	0 - 53	-	0 - 69

PPS		Placebo	CD133+	Total
Visit I (Screening)	N	28	25	53
	Mean	30.4	31.9	31.1
	Std	12.28	20.78	16.68
	Median	31.5	27.0	29.0
	Q1 - Q3	23 - 40	19 - 41	19 - 41
	Min - Max	2 - 49	4 - 89	2 - 89
Visit III (Hospital Discharge)	N	15	12	27
	Mean	32.1	33.5	32.7
	Std	10.89	18.29	14.35
	Median	30.0	30.5	30.0
	Q1 - Q3	20 - 39	21 - 48	20 - 44
	Min - Max	18 - 51	9 - 72	9 - 72
Visit V (6-Month Follow-Up)	N	29	25	54
	Mean	34.8	27.9	31.6
	Std	12.02	12.50	12.61
	Median	32.0	28.0	30.0
	Q1 - Q3	29 - 42	22 - 34	25 - 37
	Min - Max	16 - 64	1 - 53	1 - 64

Source: P132_perfect - MRI02T.sas [SVN:28096] Data Extract: 15JUL2016 Generation Date : 09AUG2016 11:19

Table 13 Non-viable tissue measured with MRI in the FAS/ITT and the PPS

FAS/ITT		Placebo	CD133+	Not treated	Total
Visit I (Screening)	N	37	34	2	73
	Mean	25.8	24.3	31.5	25.3
	Std	14.31	18.42	16.26	16.22
	Median	27.0	19.5	31.5	22.0
	Q1 - Q3	14 - 37	12 - 32	20 - 43	13 - 36
	Min - Max	0 - 56	0 - 70	20 - 43	0 - 70
Visit III (Hospital Discharge)	N	17	14	0	31
	Mean	27.6	25.2	-	26.5
	Std	14.58	15.53	-	14.81
	Median	27.0	25.5	-	27.0
	Q1 - Q3	16 - 33	15 - 34	-	15 - 34
	Min - Max	7 - 55	0 - 57	-	0 - 57
Visit V (6-Month Follow-Up)	N	33	27	0	60
	Mean	30.2	20.0	-	25.6
	Std	14.11	12.85	-	14.38
	Median	28.0	20.0	-	24.5
	Q1 - Q3	19 - 39	9 - 30	-	17 - 35
	Min - Max	6 - 65	0 - 50	-	0 - 65

PPS		Placebo	CD133+	Total
Visit I (Screening)	N	28	25	53
	Mean	24.1	26.0	25.0
	Std	14.22	20.56	17.35
	Median	25.0	19.0	21.0
	Q1 - Q3	11 - 37	12 - 36	12 - 36
	Min - Max	0 - 50	0 - 70	0 - 70
Visit III (Hospital Discharge)	N	15	12	27
	Mean	25.7	26.6	26.1
	Std	13.94	14.77	14.04
	Median	27.0	25.5	27.0
	Q1 - Q3	15 - 33	16 - 34	15 - 33
	Min - Max	7 - 55	6 - 57	6 - 57
Visit V (6-Month Follow-Up)	N	29	25	54
	Mean	28.0	20.7	24.6
	Std	12.63	12.70	13.07
	Median	26.0	20.0	23.5
	Q1 - Q3	19 - 39	11 - 30	17 - 32
	Min - Max	6 - 59	0 - 50	0 - 59

Source: P132_perfect - MRI02T.sas [SVN:28096]   Data Extract: 15JUL2016   Generation Date : 09AUG2016 11:19

11.1.2 Conclusions

With p-values of 0.8130 and 0.4454 for Visit III and Visit V respectively in the FAS/ITT and of 0.6771 and 0.4261 for Visit III and Visit V respectively in the PPS, the difference between the treatments groups is not statistically significant. This means that the H0: $\mu1<=\mu2$, with H1: $\mu1>\mu2$, with $\mu1$: mean LVEF at 6 months post-OP measured by MRI in the active treatment group and $\mu2$: mean LVEF at 6 months post-OP measured by MRI in the placebo group, could not be rejected. The probability of not having detected a possible positive effect of the therapy (injection of CD133+ in the myocardium) on the improvement of LVEF is very small.

Though there were no statistical tests performed with the following endpoints (this would enable a statement on the effect of the treatment), the following was observed:

- Unadjusted values of LVEF measured by MRI revealed a larger increase in the patients treated with CD133+. In the FS/ITT, baseline LVEF for the placebo group was 35,1 compared to 42.5 at Visit V and for the treatment group it was 32.7 at baseline versus 44.1 at Visit V.
- Mean values of scar tissue in the FAS/ITT measured by MRI were less in the CD133+ group than in the placebo group (27.0 versus 37.3) and in the PPS were also less in the CD133+ group than in the placebo group (27.9 versus 34.8).
- Mean values of non-viable tissue in the FAS/ITT measured by MRI were less in the CD133+ group than in the placebo group (20.0 versus 30.2) and in the PPS were also less in the CD133+ group than in the placebo group (20.7 versus 28.0).

11.2 Secondary efficacy analysis

The secondary endpoints were the following:

- Change in LVEF at 6 months post-OP compared with preoperatively (screening) and early postoperatively (discharge) as assessed by echocardiography.
- Change in LV dimensions ([LVESD], [LVEDD]) at 6-month post-OP compared with preoperatively (screening) and early postoperatively (discharge) as assessed by echocardiography.
- Change in physical exercise capacity at 6 months post-OP compared with preoperatively (screening) and early postoperatively (discharge) determined by 6-minute walk test.
- Change in NYHA and CCS class at 6 months post-OP compared with preoperatively (screening) and early postoperatively (discharge).
- Occurrence of MACE (cardiac death, myocardial infarction, secondary intervention/reoperation, ventricular arrhythmia).
- EQ-5D
- MLHF-Q
- SF36

All secondary endpoint parameters were summarized by means of descriptive statistics (mean, SD, median, minimum, maximum, number of available observation and number of missing observations) or frequency tables and by treatment. All summaries were performed in all analysis groups: FAS/ITT, PPS, I-CD133+-AS. Results of the I-CD133+-AS set are not presented because there were only three patients included in this set.

11.2.1 Results

11.2.1.1 Changes in LVEF

Summary statistics of the change in LVEF assessed by echocardiography at 6 months post-OP compared with preoperatively (Visit I) and early postoperatively (Visit III) in the FAS/ITT and the PPS are listed in Table 14.

Table 14 Summary statistics of the change in LVEF assessed by echocardiography in the FAS/ITT and the PPS

FAS/ITT	Placebo	CD133+	Not treated	Total
Difference between Visit V (6-Month Follow-Up) and Visit I (Screening)				
N[a]	32	31	0	63
Mean[b]	5.1	6.0	-	5.6
Std	10.73	7.48	-	9.21
Median	3.5	5.0	-	5.0
Q1 - Q3	0 - 10	0 - 10	-	0 - 10
Min - Max	-13 - 47	-10 - 23	-	-13 - 47
Difference between Visit V (6-Month Follow-Up) and Visit III (Hospital Discharge)				
N[a]	28	30	0	58
Mean[c]	4.5	4.3	-	4.4
Std	9.70	5.82	-	7.86
Median	5.0	4.0	-	5.0
Q1 - Q3	0 - 12	0 - 8	-	0 - 10
Min - Max	-22 - 25	-7 - 15	-	-22 - 25

PPS	Placebo (N=30)	CD133+ (N=28)	Total (N=58)
Difference between Visit V (6-Month Follow-Up) and Visit I (Screening)			
N	25	26	51
Mean	3.6	6.0	4.8
Std	7.58	7.87	7.75
Median	5.0	6.0	5.0
Q1 - Q3	0 - 9	0 - 10	0 - 10
Min - Max	-13 - 18	-10 - 23	-13 - 23
Difference between Visit V (6-Month Follow-Up) and Visit III (Hospital Discharge)			
N	22	26	48
Mean	4.2	4.7	4.5
Std	9.28	5.69	7.47
Median	5.0	4.5	5.0
Q1 - Q3	0 - 13	0 - 8	0 - 10
Min - Max	-22 - 18	-5 - 15	-22 - 18

[a]Number of measurements
[b]The difference was calculated as $Value_{6months}$ – $Value_{VI}$, therefore a positive mean value represents an increase of the LVEF
[c]The difference was calculated as $Value_{6months}$ – $Value_{VIII}$, therefore a positive mean value represents an increase of the LVEF
Source: P132_perfect - MRI08T.sas [SVN:27200] Data Extract: 15JUL2016 Generation Date : 09AUG2016 11:17

Overall there was an improvement of the LVEF in both the treatment and placebo groups.

Since the results of the LVEF analysis depend very much on the quality of the echocardiography, this was analysed in detail (Table 15). In the FAS/ITT, at Visit I, there were only 5% echocardiographies considered to be of good quality and at Visit V only 2.5 %; in the PPS, at Visit 1, only 3.4% echocardiographies were considered to be of good quality and at Visit V only 1.7%.

Table 15 Quality of the echocardiography in the FAS/ITT and the PPS

**FAS/ITT**		**Placebo**	**CD133+**	**Total**
**Quality of echocardiography**				
Visit I (Screening)	Good	-	4 (10.5%)	4 (5.0%)
	Middle	22 (55.0%)	13 (34.2%)	36 (45.0%)
	Bad	14 (35.0%)	16 (42.1%)	30 (37.5%)
	MISSING	4 (10.0%)	5 (13.2%)	10 (12.5%)
	N	40	38	80
	orthograde ultrasound not measurable	8 (20.0%)	8 (21.1%)	16 (20.0%)
	MISSING	32 (80.0%)	30 (78.9%)	64 (80.0%)
	N	40	38	80
Visit V (6-Month Follow-Up)	Good	2 (5.1%)	-	2 (2.5%)
	Middle	18 (46.2%)	19 (47.5%)	37 (45.7%)
	Bad	9 (23.1%)	11 (27.5%)	20 (24.7%)
	MISSING	10 (25.6%)	10 (25.0%)	22 (27.2%)
	orthograde ultrasound not measurable	13 (33.3%)	6 (15.0%)	19 (23.5%)
	MISSING	26 (66.7%)	34 (85.0%)	62 (76.5%)
	N	39	40	81

(continued)

Table 14 (cont.) Quality of the echocardiography in the FAS/ITT and the PPS

PPS		Placebo (N=30)	CD133+ (N=28)	Total (N=58)
Visit I (Screening)	good	-	2 (7.1%)	2 (3.4%)
	middle	15 (50.0%)	11 (39.3%)	26 (44.8%)
	bad	13 (43.3%)	11 (39.3%)	24 (41.4%)
	MISSING	2 (6.7%)	4 (14.3%)	6 (10.3%)
	N	30	28	58
	orthograde ultrasound not measurable	5 (16.7%)	5 (17.9%)	10 (17.2%)
	MISSING	25 (83.3%)	23 (82.1%)	48 (82.8%)
	N	30	28	58
Visit V (6-Month Follow-Up)	good	1 (3.3%)	-	1 (1.7%)
	middle	14 (46.7%)	15 (53.6%)	29 (50.0%)
	bad	8 (26.7%)	10 (35.7%)	18 (31.0%)
	MISSING	7 (23.3%)	3 (10.7%)	10 (17.2%)

Note: Denominator for percentages is column N.

Source: P132_perfect - MRI02T.sas [SVN:28096] Data Extract: 15JUL2016 Generation Date : 09AUG2016 11:17

11.2.1.2 Change in left ventricular dimensions

Summary statistics of the change of LVEDD and LVESD assessed by echocardiography in the FAS/ITT and the PPS at 6 months post-OP compared with preoperatively (Visit I) and early postoperatively are presented in Appendix B 0311_ECHO02T_FAS.rtf.

Differences between measurements taken at 6 month and screening or hospital discharge are presented in Table 16 and Table 17 respectively.

Table 16 Difference from month 6 to screening and hospital discharge in LVEDD (echocardiography) in the FAS/ITT and the PPS, by treatment group

FAS/ITT	Placebo	CD133+	Not treated	Total
Difference between Visit V (6-Month Follow-Up) and Visit I (Screening)				
N[a]	33	29	0	62
Mean[b]	-3.9	-0.6	-	-2.3
Std	12.46	6.74	-	10.25
Median	-3.0	1.0	-	0.0
Q1 - Q3	-7 - 2	-1 - 3	-	-6 - 3
Min - Max	-62 - 19	-29 - 8	-	-62 - 19
Difference between Visit V (6-Month Follow-Up) and Visit III (Hospital Discharge)				
N[a]	23	26	0	49
Mean[c]	-1.0	1.3	-	0.2
Std	4.94	5.57	-	5.35
Median	0.0	0.5	-	0.0
Q1 - Q3	-5 - 2	-2 - 4	-	-3 - 3
Min - Max	-9 - 13	-11 - 13	-	-11 - 13

PPS	Placebo (N=30)	CD133+ (N=28)	Total (N=58)
Difference between Visit V (6-Month Follow-Up) and Visit I (Screening)			
N[a]	26	25	51
Mean[b]	-2.2	-0.9	-1.6
Std	7.42	7.05	7.20
Median	-2.5	1.0	0.0
Q1 - Q3	-8 - 2	-1 - 2	-6 - 2
Min - Max	-14 - 19	-29 - 8	-29 - 19
Difference between Visit V (6-Month Follow-Up) and Visit III (Hospital Discharge)			
N[a]	18	22	40
Mean[c]	-0.6	1.0	0.3
Std	5.10	5.13	5.11
Median	0.0	0.0	0.0
Q1 - Q3	-4 - 2	-2 - 4	-3 - 3
N[a]	-9 - 13	-11 - 12	-11 - 13

[a]Number of measurements
[b]The difference was calculated as $Value_{6months}$ – $Value_{VI}$, therefore a negative mean value represents a decrease of the LVEDD
[c]The difference was calculated as $Value_{6months}$ – $Value_{VIII}$, therefore a negative mean value represents a decrease of the LVEDD
Source: P132_perfect - ECHO10T.sas [SVN:28562] Data Extract: 15JUL2016 Generation Date : 09AUG2016 11:17

Table 17 Difference from month 6 to screening and hospital discharge in LVESD (echocardiography) in the FAS/ITT and the PPS, by treatment group

FAS/ITT	Placebo	CD133+	Not treated	Total
Difference between Visit V (6-Month Follow-Up) and Visit I (Screening)				
N[a]	23	21	0	44
Mean[b]	0.2	0.5	-	0.4
Std	11.20	6.03	-	9.00
Median	1.0	1.0	-	1.0
Q1 - Q3	-7 - 7	-5 - 6	-	-6 - 7
Min - Max	-22 - 28	-10 - 9	-	-22 - 28
Difference between Visit V (6-Month Follow-Up) and Visit III (Hospital Discharge)				
N[a]	15	16	0	31
Mean[c]	3.5	1.4	-	2.4
Std	22.55	7.78	-	16.39
Median	-3.0	0.5	-	0.0
Q1 - Q3	-5 - 5	-4 - 7	-	-5 - 5
Min - Max	-19 - 80	-10 - 14	-	-19 - 80

PPS	Placebo	CD133+	Total
Difference between Visit V (6-Month Follow-Up) and Visit I (Screening)			
N[a]	18	18	36
Mean[b]	-1.6	0.1	-0.8
Std	10.19	6.18	8.35
Median	-2.5	0.5	-1.0
Q1 - Q3	-7 - 5	-5 - 6	-6 - 6
Min - Max	-22 - 13	-10 - 8	-22 - 13
Difference between Visit V (6-Month Follow-Up) and Visit III (Hospital Discharge)			
N[a]	11	15	26
Mean[c]	-2.5	1.5	-0.2
Std	8.94	8.04	8.49
Median	-4.0	1.0	-2.5
Q1 - Q3	-7 - 5	-4 - 9	-5 - 5
Min - Max	-19 - 13	-10 - 14	-19 - 14

[a]Number of measurements
[b]The difference was calculated as $Value_{6months}$ – $Value_{VI}$, therefore a positive mean value represents an increase of the LVESD
[c]The difference was calculated as $Value_{6months}$ – $Value_{VIII}$, therefore a positive mean value represents an increase of the LVESD
Source: P132_perfect - ECHO10T.sas [SVN:28562]   Data Extract: 15JUL2016   Generation Date : 09AUG2016 11:17

11.2.1.3 Change in physical exercise capacity

Summary statistics of the change in physical exercise capacity at 6 months post-OP compared with preoperatively (Visit I) and early postoperatively (discharge) in the FAS/ITT and the PPS determined by a 6-minute walk test are presented in Table 18.

Table 18 Difference from month 6 to screening and hospital discharge in total distance (6MWT) in the FAS/ITT and the PPS, by treatment group

FAS/ITT	Placebo	CD133+	Not treated	Total
Difference between Visit V (6-Month Follow-Up) and Visit I (Screening)				
N[a]	33	21	0	54
Mean[b]	49.3	59.4	-	53.3
Std	107.03	106.60	-	105.96
Median	50.0	30.0	-	46.5
Q1 - Q3	-5 - 117	-9 - 141	-	-9 - 117
Min – Max	-170 - 310	-93 - 369	-	-170 - 369
Difference between Visit V (6-Month Follow-Up) and Visit III (Hospital Discharge)				
N[a]	24	16	0	40
Mean[c]	48.0	100.8[d]	-	69.1
Std	104.31	69.69[d]	-	94.72
Median	49.5	94.3	-	75.0
Q1 - Q3	-20 - 100	59 - 159	-	1 - 149
Min - Max	-156 - 275	-42 - 227	-	-156 - 275

PPS	Placebo	CD133+	Total
Difference between Visit V (6-Month Follow-Up) and Visit I (Screening)			
N[a]	25	17	42
Mean[b]	50.7	56.1	52.9
Std	116.17	113.13	113.58
Median	47.0	30.0	39.5
Q1 - Q3	-5 - 117	-30 - 100	-28 - 117
Min – Max	-170 - 310	-93 - 369	-170 - 369
Difference between Visit V (6-Month Follow-Up) and Visit III (Hospital Discharge)			
N[a]	19	14	33
Mean[c]	44.7	90.0	63.9
Std	111.16	64.80	95.77
Median	45.0	85.5	73.0
Q1 - Q3	-34 - 112	45 - 158	2 - 147
N[a]	-156 - 275	-42 - 163	-156 - 275

[a]Number of measurements
[b]The difference was calculated as $Value_{6months}$ – $Value_{VI}$, therefore a positive mean value represents an increase of the exercise capacity
[c]The difference was calculated as $Value_{6months}$ – $Value_{VIII}$, therefore a positive mean value represents an increase of the exercise capacity
[d]These two values that are not within the expected ranges are due to the non-standardized methods used to assess this endpoint.
Source: P132_perfect - 6MWT06T.sas [SVN:27200] Data Extract: 15JUL2016 Generation Date : 09AUG2016 11:18

11.2.1.4 Change in NYHA and CCS class

Patients were graded per the NYHA classification of heart failure in classes I to IV where higher scores reflect poorer quality of life. The roman numerals were converted in arabic numerals to make the analysis described below. In case more than one NYHA grading was reported per patient and visit the

average value was used. For more details on the description of symptoms leading to the score see Table 5 in the SAP (Appendix 1).

Additionally, the CCS classification was used to classify angina pectoris in classes 0 to IV. Higher scores reflect also here poorer quality of life. The roman numerals have been converted to arabic numerals for the analysis. In case more than one CCS grading had been reported per patient and visit, the average value was used. For more details on the description of symptoms leading to the score see Table 6 in the SAP (Appendix 1).

The differences between screening/hospital discharge and 6-month follow-up are displayed in Table 19 and Table 20 respectively.

Table 19 Difference from month 6 to screening and hospital discharge in NYHA classification in the FAS/ITT and the PPS, by treatment group

**FAS/ITT**	**Placebo**	**CD133+**	**Not treated**	**Total**
Difference between Visit V (6-Month Follow-Up) and Visit I (Screening)				
N[a]	37	32	0	69
Mean[b]	-0.7	-0.7	-	-0.7
Std	0.85	1.00	-	0.92
Median	-1.0	-1.0	-	-1.0
Q1 - Q3	-1 - 0	-1 - 0	-	-1 - 0
Min - Max	-2 - 2	-2 - 2	-	-2 - 2
Difference between Visit V (6-Month Follow-Up) and Visit III (Hospital Discharge)				
N[a]	30	19	0	49
Mean[c]	-0.1	-0.4	-	-0.2
Std	0.71	0.90	-	0.80
Median	0.0	-1.0	-	0.0
Q1 - Q3	0 - 0	-1 - 0	-	-1 - 0
Min - Max	-2 - 2	-2 - 2	-	-2 - 2

**PPS**	**Placebo**	**CD133+**	**Total**
Difference between Visit V (6-Month Follow-Up) and Visit I (Screening)			
N[a]	29	27	56
Mean[b]	-0.7	-0.8	-0.7
Std	0.71	0.97	0.84
Median	-1.0	-1.0	-1.0
Q1 - Q3	-1 - 0	-1 - 0	-1 - 0
Min - Max	-2 - 1	-2 - 2	-2 - 2
Difference between Visit V (6-Month Follow-Up) and Visit III (Hospital Discharge)			
N[a]	22	15	37
Mean[c]	-0.1	-0.6	-0.3
Std	0.53	0.74	0.66
Median	0.0	-1.0	0.0
Q1 - Q3	0 - 0	-1 - 0	-1 - 0
Min - Max	-1 - 1	-2 - 1	-2 - 1

[a]Number of measurements
[b]The difference was calculated as $Value_{6months}$ – $Value_{VI}$, therefore a negative mean value represents an NYHA improvement
[c]The difference was calculated as $Value_{6months}$ – $Value_{VIII}$, therefore a positive mean value represents an NYHA worsening
Source: P132_perfect - NYHACCS02T.sas [SVN:27384] Data Extract: 15JUL2016 Generation Date : 09AUG2016 11:18

Table 20 Difference from month 6 to screening and hospital discharge in CCS classification in the FAS/ITT and the PPS, by treatment group

FASD/ITT	Placebo	CD133+	Not treated	Total
Difference between Visit V (6-Month Follow-Up) and Visit I (Screening)				
N[a]	36	32	0	68
Mean[b]	-1.4	-1.0	-	-1.2
Std	1.13	1.28	-	1.20
Median	-2.0	0.0	-	-1.0
Q1 - Q3	-2 - 0	-2 - 0	-	-2 - 0
Min - Max	-3 - 0	-3 - 1	-	-3 - 1
Difference between Visit V (6-Month Follow-Up) and Visit III (Hospital Discharge)				
N[a]	29	17	0	46
Mean[c]	-0.2	0.2	-	-0.1
Std	0.94	0.88	-	0.93
Median	0.0	0.0	-	0.0
Q1 - Q3	-1 - 0	0 - 0	-	0 - 0
Min - Max	-2 - 2	-2 - 2	-	-2 - 2

PPS	Placebo	CD133+	Total
Difference between Visit V (6-Month Follow-Up) and Visit I (Screening)			
N[a]	28	27	55
Mean[b]	-1.4	-0.9	-1.1
Std	1.10	1.24	1.18
Median	-2.0	0.0	-1.0
Q1 - Q3	-2 - 0	-2 - 0	-2 - 0
Min - Max	-3 - 0	-3 - 1	-3 - 1
Difference between Visit V (6-Month Follow-Up) and Visit III (Hospital Discharge)			
N[a]	21	13	34
Mean[c]	-0.1	0.1	-0.1
Std	0.85	0.86	0.85
Median	0.0	0.0	0.0
Q1 - Q3	0 - 0	0 - 0	0 - 0
Min - Max	-2 - 2	-2 - 2	-2 - 2

[a]Number of measurements
[b]The difference was calculated as $Value_{6months}$ – $Value_{VI}$, therefore a negative mean value represents an CCS improvement
[c]The difference was calculated as $Value_{6months}$ – $Value_{VIII}$, therefore a positive mean value represents an CCS worsening
Source: P132_perfect - NYHACCS02T.sas [SVN:27384] Data Extract: 15JUL2016 Generation Date : 09AUG2016 11:18

11.2.1.5 Occurrence of MACE

A summary of the MACEs that occurred in the FAS/ITT during the study up to 6 months post-OP is displayed in Table 21.

.

Table 21 Number of MACEs during the main study phase up to Visit V in the FAS/ITT by treatment group

	CD133+	Placebo	Total	p-value [1]
pts with at least one MACE	1 (2.7%)	1 (2.5%)	2 (2.6%)	1.0000
number of MACEs	1	1	2	

Source: P132_perfect - AE01T.sas [SVN:29690] Data Extract: 15JUL2016 Generation Date : 23SEP2016 12:45

To check the differences between the treatment groups, an unadjusted survival analysis with Kaplan-Meier estimations was performed using the logrank test. As the number of MACE occurrence was very low, only the Kaplan-Meier plot computed for all categories of MACE is included in Figure 3. With a p-value of 0,9469, there is no significant difference in the occurrence of MACE between the two groups of patients (Placebo or CD 133+).

Figure 3 Kaplan-Meier plot of the occurrence of MACE in the FAS/ITT

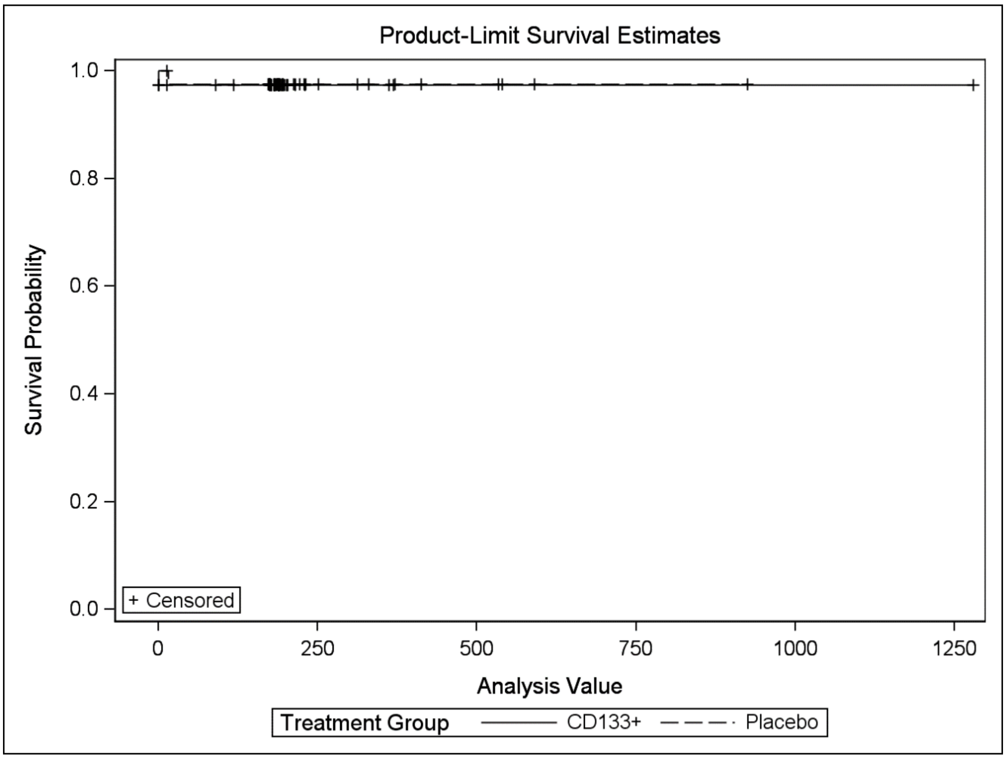

The number of MACE was also low in the PPS population (only 1 CD 133+ patient had a ventricular arrhythmia).

11.2.1.6 QoL-score

The QoL of the patients has been evaluated using 3 different questionnaires:

- EQ-5D
- MLHF-Q
- SF36

The evaluation was performed at baseline (Visit I), per telephone 3 months after the operation (Visit IV) and during the 6-month post-Op visit (Visit V).

11.2.1.6.1 EQ-5D

The EQ-5D consists of 2 parts: the EQ-5D descriptive systems and the EQ visual analogue scale (VAS). The descriptive system comprises the following 5 questions: mobility, self-care, usual activities, pain/discomfort and anxiety/depression. The patient is asked to indicate his/her health state by ticking in the box of the most appropriate statement in each of the 5 questions. Each of the 5 questions is divided into 3 levels of perceived problems:

Level 1: indicates no problem

Level 2: indicates some problems

Level 3: indicates extreme problems

A health state is created by combining the answers to the 5 questions as a 5-digit code. (EuroQolGroup, 2014)

For example, state 11111 indicates no problems on any of the 5 dimensions, while state 11223 indicates no problems with mobility and self-care, some problems with performing usual activities, moderate pain or discomfort and extreme anxiety or depression.

When answering the EQ VAS the patient was asked to indicate his/her health state by marking the scale between 100 (best imaginable health state) and 0 (worst imaginable health state).

The changes in mobility in the FAS/ITT and the PPS at 6 months post-OP compared with preoperatively and 3 months are summarized in Table 22.

Table 22 EQ-5D - mobility - Difference between level at 6 months post-OP to screening and 3-months post-OP

FAS/ITT	Placebo	CD133+	Not treated	Total
Difference between Visit V (6-Month Follow-Up) and Visit I (Screening)				
N[a]	28	24	0	52
Mean[b]	0.0	-0.2	-	-0.1
Std	0.54	0.51	-	0.53
Median	0.0	0.0	-	0.0
Q1 - Q3	0 - 0	-1 - 0	-	0 - 0
Min - Max	-1 - 1	-1 - 1	-	-1 - 1
Difference between Visit V (6-Month Follow-Up) and Visit IV (3-Month Follow-Up)				
N[a]	29	25	0	54
Mean[c]	-0.1	0.0	-	-0.0
Std	0.26	0.20	-	0.24
Median	0.0	0.0	-	0.0
Q1 - Q3	0 - 0	0 - 0	-	0 - 0
Min - Max	-1 - 0	0 - 1	-	-1 - 1

PPS	Placebo	CD133+	Total
Difference between Visit V (6-Month Follow-Up) and Visit I (Screening)			
N	21	19	40
Mean	0.0	-0.3	-0.1
Std	0.38	0.48	0.46
Median	0.0	0.0	0.0
Q1 - Q3	0 - 0	-1 - 0	0 - 0
Min - Max	-1 - 1	-1 - 0	-1 - 1
Difference between Visit V (6-Month Follow-Up) and Visit IV (3-Month Follow-Up)			
N	22	21	43
Mean	-0.0	0.0	-0.0
Std	0.21	0.00	0.15
Median	0.0	0.0	0.0
Q1 - Q3	0 - 0	0 - 0	0 - 0
Min - Max	-1 - 0	0 - 0	-1 - 0

[a]Number of measurements
[b]The difference was calculated as $Value_{6months}$ – $Value_{V\ I}$, therefore a negative mean value represents a mobility improvement
[c]The difference was calculated as $Value_{6months}$ – $Value_{V\ IV}$, therefore a negative mean value represents a mobility improvement
Source: P132_perfect - EQ5D01T.sas [SVN:27384] Data Extract: 15JUL2016 Generation Date : 09AUG2016 11:25

The tables for self-care, usual activities, pain/discomfort and anxiety/depression are available in Section 14. Like the results for mobility, there were minimal changes in these variables.

The changes in EQ-5D VAS are summarized in Table 23 and indicate an overall limited improvement on health state as perceived by the patients.

Table 23 EQ-5D – VAS - Difference between level at 6 months post-OP to screening and 3-months post-OP

FAS/ITT	Placebo	CD133+	Not treated	Total
Difference between Visit V (6-Month Follow-Up) and Visit I (Screening)				
N[a]	28	23	0	51
Mean[b]	6.1	11.1	-	8.4
Std	18.00	23.39	-	20.55
Median	7.5	10.0	-	10.0
Q1 - Q3	-6 - 20	-1 - 30	-	-5 - 25
Min - Max	-35 - 35	-47 - 46	-	-47 - 46
Difference between Visit V (6-Month Follow-Up) and Visit IV (3-Month Follow-Up)				
N[a]	30	25	0	55
Mean[c]	5.0	6.3	-	5.6
Std	14.00	14.30	-	14.02
Median	5.0	5.0	-	5.0
Q1 - Q3	-1 - 15	0 - 20	-	-1 - 16
Min - Max	-30 - 30	-20 - 30	-	-30 - 30

PPS	Placebo	CD133+	Total
Difference between Visit V (6-Month Follow-Up) and Visit I (Screening)			
N	21	18	39
Mean	4.4	16.4	9.9
Std	18.23	22.49	20.92
Median	5.0	20.0	14.0
Q1 - Q3	-5 - 15	5 - 30	-4 - 25
Min - Max	-35 - 30	-47 - 46	-47 - 46
Difference between Visit V (6-Month Follow-Up) and Visit IV (3-Month Follow-Up)			
N	23	21	44
Mean	5.6	6.4	6.0
Std	13.48	14.78	13.95
Median	5.0	5.0	5.0
Q1 - Q3	0 - 15	0 - 20	0 - 16
Min - Max	-30 - 30	-20 - 30	-30 - 30

[a]Number of measurements
[b]The difference was calculated as $Value_{6months}$ – $Value_{V\ I}$, therefore a positive mean value represents a health state improvement
[c]The difference was calculated as $Value_{6months}$ – $Value_{V\ IV}$, therefore a positive mean value represents a health state improvement
Source: P132_perfect - EQ5D01T.sas [SVN:27384] Data Extract: 15JUL2016 Generation Date : 09AUG2016 11:25

11.2.1.6.2 MLHF-Q

The MLHF-Q uses Likert-type response scales ranging from 0 (no effect on QOL), to 5 (highest impact on QOL) where higher scores reflect poorer QOL. The total score of MLHF-Q was calculated per patient

and visit as the sum of the 21 questions. The minimum score for a patient is 0, the maximum score 120 (17).

Additionally, the sum of the physical (questions 2-7, 12-13) and emotional (questions 17-21) components were calculated. The sum score of the physical component ranges from 0 to 40, the sum score of the emotional components from 0 to 25.

The changes in the scores computed based on the answers of MLHF-Q in the FAS/ITT and the PPS at 6 months post-OP compared with preoperatively and 3 months are summarized in Table 24 for the emotional score,

Table 25 for the physical score and

Table 26 for the total score.

Table 24 MLHF-Q – Emotional score - Difference between level at 6 months post-OP to screening and 3-months post-OP

FAS/ITT	Placebo	CD133+	Not treated	Total
Difference between Visit V (6-Month Follow-Up) and Visit I (Screening)				
N[a]	33	29	0	62
Mean[b]	-3.5	-1.8	-	-2.7
Std	5.57	5.74	-	5.67
Median	-3.0	-1.0	-	-1.5
Q1 - Q3	-6 - 0	-6 - 1	-	-6 - 0
Min - Max	-15 - 8	-13 - 17	-	-15 - 17
Difference between Visit V (6-Month Follow-Up) and Visit IV (3-Month Follow-Up)				
N[a]	34	27	0	61
Mean[c]	-1.7	-1.9	-	-1.8
Std	4.87	5.54	-	5.13
Median	-1.0	0.0	-	-1.0
Q1 - Q3	-3 - 0	-2 - 0	-	-2 - 0
Min - Max	-12 - 12	-18 - 7	-	-18 - 12

Continued

Table 24 (cont.) MLHF-Q – Emotional score - Difference between level at 6 months post-OP to screening and 3-months post-OP

PPS	Placebo	CD133+	Total
Difference between Visit V (6-Month Follow-Up) and Visit I (Screening)			
N	26	24	50
Mean	-4.1	-2.3	-3.2
Std	5.59	6.14	5.87
Median	-3.5	-1.5	-3.0
Q1 - Q3	-7 - 0	-7 - 1	-7 - 0
Min - Max	-15 - 8	-13 - 17	-15 - 17
Difference between Visit V (6-Month Follow-Up) and Visit IV (3-Month Follow-Up)			
N	26	23	49
Mean	-2.2	-2.4	-2.3
Std	5.03	5.82	5.36
Median	-1.0	-1.0	-1.0
Q1 - Q3	-4 - 0	-2 - 0	-3 - 0
Min - Max	-12 - 12	-18 - 7	-18 - 12

Summary Scores only calculated if all contributing items are available
[a]Number of measurements
[b]The difference was calculated as $Value_{6months}$ – $Value_{V\ I}$, therefore a negative mean value represents an emotional score improvement
[c]The difference was calculated as $Value_{6months}$ – $Value_{V\ IV}$, therefore a negative mean value represents an emotional score improvement
Source: P132_perfect - MLHFQ01T.sas [SVN:27969] Data Extract: 15JUL2016 Generation Date : 09AUG2016 11:25

Table 25 MLHF-Q – Physical score - Difference between level at 6 months post-OP to screening and 3-months post-OP

FAS/ITT	Placebo	CD133+	Not treated	Total
Difference between Visit V (6-Month Follow-Up) and Visit I (Screening)				
N[a]	34	29	0	63
Mean[b]	-5.6	-5.6	-	-5.6
Std	10.55	10.89	-	10.62
Median	-5.0	-5.0	-	-5.0
Q1 - Q3	-13 - -1	-11 - 0	-	-13 - 0
Min - Max	-29 - 18	-25 - 19	-	-29 - 19
Difference between Visit V (6-Month Follow-Up) and Visit IV (3-Month Follow-Up)				
N[a]	35	28	0	63
Mean[c]	-3.5	-3.7	-	-3.6
Std	7.91	10.25	-	8.95
Median	-2.0	-1.0	-	-2.0
Q1 - Q3	-6 - 1	-8 - 2	-	-6 - 1
Min - Max	-26 - 11	-33 - 15	-	-33 - 15

PPS	Placebo	CD133+	Total
Difference between Visit V (6-Month Follow-Up) and Visit I (Screening)			
N	26	24	50
Mean	-6.1	-6.0	-6.1
Std	9.35	11.52	10.34
Median	-5.0	-6.0	-5.0
Q1 - Q3	-15 - -1	-14 - 2	-15 - 0
Min - Max	-29 - 10	-25 - 19	-29 - 19
Difference between Visit V (6-Month Follow-Up) and Visit IV (3-Month Follow-Up)			
N	27	24	51
Mean	-3.6	-3.5	-3.5
Std	8.49	10.86	9.58
Median	-2.0	-0.5	-1.0
Q1 - Q3	-7 - 1	-7 - 2	-7 - 1
Min - Max	-26 - 11	-33 - 15	-33 - 15

Summary Scores only calculated if all contributing items are available
[a]Number of measurements
[b]The difference was calculated as $Value_{6months}$ – $Value_{V\ I}$, therefore a negative mean value represents a physical score improvement
[c]The difference was calculated as $Value_{6months}$ – $Value_{V\ IV}$, therefore a negative mean value represents a physical score improvement
Source: P132_perfect - MLHFQ01T.sas [SVN:27969] Data Extract: 15JUL2016 Generation Date : 09AUG2016 11:25

Table 26 MLHF-Q – Total score - Difference between level at 6 months post-OP to screening and 3-months post-OP

FAS/ITT	Placebo	CD133+	Not treated	Total
Difference between Visit V (6-Month Follow-Up) and Visit I (Screening)				
N[a]	23	25	0	48
Mean[b]	-14.7	-8.6	-	-11.5
Std	19.29	20.42	-	19.92
Median	-15.0	-8.0	-	-11.0
Q1 - Q3	-30 - 0	-16 - 0	-	-27 - 0
Min - Max	-49 - 27	-52 - 31	-	-52 - 31
Difference between Visit V (6-Month Follow-Up) and Visit IV (3-Month Follow-Up)				
N[a]	23	20	0	43
Mean[c]	-6.3	-3.5	-	-5.0
Std	18.64	20.70	-	19.44
Median	-4.0	-2.0	-	-2.0
Q1 - Q3	-16 - 5	-5 - 4	-	-6 - 4
Min - Max	-48 - 34	-70 - 21	-	-70 - 34

PPS	Placebo	CD133+	Total
Difference between Visit V (6-Month Follow-Up) and Visit I (Screening)			
N	18	21	39
Mean	-16.1	-10.1	-12.9
Std	17.04	21.44	19.52
Median	-16.5	-9.0	-13.0
Q1 - Q3	-30 - -3	-18 - 0	-30 - 0
Min - Max	-49 - 11	-52 - 31	-52 - 31
Difference between Visit V (6-Month Follow-Up) and Visit IV (3-Month Follow-Up)			
N	15	17	32
Mean	-7.7	-3.8	-5.6
Std	21.24	22.47	21.64
Median	-4.0	-2.0	-2.0
Q1 - Q3	-20 - 5	-3 - 4	-8 - 5
Min - Max	-48 - 34	-70 - 21	-70 - 34

Summary Scores only calculated if all contributing items are available
[a]Number of measurements
[b]The difference was calculated as $Value_{6months}$ – $Value_{V\ I}$, therefore a negative mean value represents a total score improvement
[c]The difference was calculated as $Value_{6months}$ – $Value_{V\ IV}$, therefore a negative mean value represents a total score improvement
Source: P132_perfect - MLHFQ01T.sas [SVN:27969] Data Extract: 15JUL2016 Generation Date : 09AUG2016 11:25

11.2.1.6.3 Short Form Questionnaire (SF36)

The SF36 consists of 36 questions. The response for each item is coded according to the original value with a value from 0-100 to make the values comparable across all questions according to the Table 4-3 Coding (see Appendix A – SAP) for SF-36 responses.

An average value per patient and visit is calculated across the items in each of the eight scales:

- Physical Functioning
- Role-Physical
- Bodily Pain
- General Health
- Vitality
- Social Functioning
- Role-Emotional
- Mental Health

as described by Ware et al. 1994 (18) and summarized in Table 4-4 SF-36 Scales and Summary Measures (See Appendix 1 – SAP paragraph 4.7.8).

Afterwards the summary measures for Physical Health and Mental Health were calculated as the average value of corresponding scales.

The changes in the scores computed based on the answers of SF36 in the FAS/ITT and the PPS at 6 months post-OP compared with preoperatively and 3 months are summarized in tables Table 42 to Table 53 in Section 14.

Table 27 and Table 28 show the changes in the overall Mental Health and the general Health.

Table 27 SF36 – Mental Health - Difference between level at 6 months post-OP to screening and 3-months post-OP

FAS/ITT	Placebo	CD133+	Not treated	Total
Difference between Visit V (6-Month Follow-Up) and Visit I (Screening)				
N[a]	36	29	0	65
Mean[b]	8.17	5.24	-	6.86
Std	19.563	18.673	-	19.079
Median	4.00	8.00	-	4.00
Q1 - Q3	-4.0 - 20.0	-8.0 - 20.0	-	-4.0 - 20.0
Min – Max	-44.0 - 60.0	-32.0 - 44.0	-	-44.0 - 60.0
Difference between Visit V (6-Month Follow-Up) and Visit IV (3-Month Follow-Up)				
N[a]	37	29	0	66
Mean[c]	4.32	2.21	-	3.39
Std	14.678	15.805	-	15.102
Median	4.00	0.00	-	0.00
Q1 - Q3	-4.0 - 12.0	-4.0 - 8.0	-	-4.0 - 12.0
Min – Max	-20.0 - 32.0	-36.0 - 40.0	-	-36.0 - 40.0

PPS	Placebo	CD133+	Total
Difference between Visit V (6-Month Follow-Up) and Visit I (Screening)			
N	28	24	52
Mean	9.64	8.33	9.04
Std	17.864	17.729	17.639
Median	6.00	8.00	8.00
Q1 - Q3	-2.0 - 22.0	-6.0 - 20.0	-4.0 - 20.0
Min – Max	-20.0 - 60.0	-32.0 - 44.0	-32.0 - 60.0
Difference between Visit V (6-Month Follow-Up) and Visit IV (3-Month Follow-Up)			
N	29	25	54
Mean	4.97	3.84	4.44
Std	13.466	16.350	14.736
Median	0.00	0.00	0.00
Q1 - Q3	-4.0 - 12.0	-4.0 - 12.0	-4.0 - 12.0
Min – Max	-20.0 - 32.0	-36.0 - 40.0	-36.0 - 40.0

Scales and Summary Measures only calculated if all contribuing items are available
[a]Number of measurements
[b]The difference was calculated as $Value_{6months}$ – $Value_{V\ I}$,
[c]The difference was calculated as $Value_{6months}$ – $Value_{V\ IV}$,
Source: P132_perfect - SF3601T.sas [SVN:27384] Data Extract: 15JUL2016 Generation Date : 09AUG2016 11:26

Table 28 SF36 – General health - Difference between level at 6 months post-OP to screening and 3-months post-OP

FAS/ITT	Placebo	CD133+	Not treated	Total
Difference between Visit V (6-Month Follow-Up) and Visit I (Screening)				
N[a]	36	29	0	65
Mean[b]	6.86	6.51	-	6.71
Std	17.967	21.747	-	19.582
Median	8.75	5.00	-	5.00
Q1 - Q3	-5.0 - 20.0	-5.0 - 20.0	-	-5.0 - 20.0
Min – Max	-35.0 - 50.0	-35.0 - 45.0	-	-35.0 - 50.0
Difference between Visit V (6-Month Follow-Up) and Visit IV (3-Month Follow-Up)				
N[a]	37	29	0	66
Mean[c]	5.03	3.71	-	4.45
Std	15.697	19.760	-	17.467
Median	5.00	0.00	-	5.00
Q1 - Q3	-5.0 - 15.0	-5.0 - 12.5	-	-5.0 - 15.0
Min – Max	-20.0 - 40.0	-30.0 - 40.0	-	-30.0 - 40.0

PPS	Placebo	CD133+	Total
Difference between Visit V (6-Month Follow-Up) and Visit I (Screening)			
N	28	24	52
Mean	10.21	8.28	9.32
Std	16.414	23.195	19.652
Median	10.00	10.00	10.00
Q1 - Q3	-2.5 - 20.0	-7.5 - 26.3	-5.0 - 22.5
Min – Max	-20.0 - 50.0	-35.0 - 45.0	-35.0 - 50.0
Difference between Visit V (6-Month Follow-Up) and Visit IV (3-Month Follow-Up)			
N	29	25	54
Mean	6.08	3.50	4.88
Std	16.076	18.736	17.239
Median	10.00	0.00	5.00
Q1 - Q3	-5.0 - 15.0	-5.0 - 12.5	-5.0 - 15.0
Min – Max	-20.0 - 40.0	-30.0 - 40.0	-30.0 - 40.0

Scales and Summary Measures only calculated if all contribuing items are available
[a]Number of measurements
[b]The difference was calculated as $Value_{6months} – Value_{V\ I}$,[c]The difference was calculated as $Value_{6months} – Value_{V\ IV}$,
Source: P132_perfect - SF3601T.sas [SVN:27384] Data Extract: 15JUL2016 Generation Date : 09AUG2016 11:26

11.2.2 Conclusions

Since the secondary efficacy analysis was only a descriptive analysis of the variables and no tests were done regarding possible differences between the placebo and the active treatment group, no conclusions can be drawn. It is though interesting to point out the following observations:

- The poor quality of the echocardiographies (Table 15) did not allow any conclusions nor a comparison with the MRI results. Since the two previous studies used the LVEF measured with echocardiography as a primary endpoint, it was decided to use this same method for the secondary endpoints to be able to compare with the previous studies.
- The 6MWT showed the following changes (Visit V-Visit 1) in the mean values: in the FAS/ITT (49.3 for the placebo group and 59.4 in the treatment group. In the PPS, these changes were 50.7 for the placebo group and 56.1 in the treatment group.
- Minimal changes in NYHA class and CCS were observed after surgery plus placebo or active treatment. Mean difference of the NYHA in the placebo and treatment group was -0.7 in the FAS/ITT and in the PPS the mean differences were -0.7 in the placebo group and -0.8 in the treatment group. Mean difference of the CCS in the placebo group was -1.4 in the treatment group was -1.0 in the FAS/ITT and in the PPS the mean differences were -1.4 in the placebo group and -0.9 in the treatment group.
- The occurrence rate of MACE was very low when compared with the occurrence reported in the literature for patients undergoing CABG surgery (26.9%) (19). With a p-value of 1.00, there was no difference in the occurrence of MACE between the two groups of patients (1 MACE in the placebo group and 1 MACE in the CD133+ group).
- There were no changes in the EQ-5D - mobility index
- The EQ-5D VAS showed some changes in the mean value: in the FAS/ITT (6.1 for the placebo group and 11.1 in the treatment group. In the PPS, these changes were 4.4 for the placebo group and 16.4 in the treatment group. It should be noted that the increase of the index indicates an improvement of the condition.
- The MLHF-Q total score showed changes in the mean value: in the FAS/ITT (-14.7 for the placebo group and -8.6 in the treatment group. In the PPS, these changes were -16.1 for the placebo group and -10.1 in the treatment group. It should be noted that a negative change in the index indicates an improvement of the condition.

12 SAFETY ANALYSES

As the number of injections varied from patient to patient, 2 additional safety analysis populations were created: SASII contained all patients from SAS who received exactly 15 injections of product (CD133+ or Placebo) and SASIII contained all patients who received more than 15 injections of product.

The results reported in the following sections are those for the SAS. Since there were no differences in the AEs between the placebo and treatment groups, there is no reason to analyse in detail the results of the analyses of the SASII and SASIII. These can be found in Appendix B.

12.1 Adverse events

12.1.1 Summary of Adverse Events

In total, there were 619 AEs during the study. Twenty-six AEs during the screening phase and 593 AEs during the main trial phase. There were no statistical differences between the placebo and the CD133+ group, neither overall nor in any of the categories in which the patients or AEs were classified (see Table 29 to Table 32).

All patients experienced at least one AE during the main trial phase. Overall there were 135 Adverse Events of Special Interest (AESIs) observed during the main trial phase, 19 AEs and two SAEs that were at least possibly related. There were no deaths during the screening nor the main trial phase.

Table 29 Summary of adverse events during screening phase

	CD133+	Placebo	Total	p-value [1]
pts with at least one AE	10 (27.0%)	7 (17.5%)	17 (22.1%)	0.4118
number of AEs	10	16	26	
pts with at least one SAE	-	1 (2.5%)	1 (1.3%)	1.0000
number of SAEs	-	1	1	
number of Deaths	-	-	-	
number of MACE	-	-	-	
pts with at least one AESI	2 (5.4%)	5 (12.5%)	7 (9.1%)	0.4334
number of AESIs	2	11	13	
pts with at least one AE that was at least possibly related	-	1 (2.5%)	1 (1.3%)	1.0000
number of AEs that were at least possibly related	-	1	1	
number of SAEs that were at least possibly related	-	-	-	
pts with at least one AE that was not or unlikely related	10 (27.0%)	6 (15.0%)	16 (20.8%)	0.2631
number of AEs that were not or unlikely related	10	15	25	
pts with at least one SAE that was not or unlikely related	-	1 (2.5%)	1 (1.3%)	1.0000
number of SAEs that were not or unlikely related	-	1	1	
number of AEs leading to withdrawal	-	-	-	

Note: For patients denominator for percentages is column N.
Note: For severity, relation and outcome denominator for percentages is total number of AEs.
[1] Group comparison using Fishers Exact Test.
Multiple occurrences of the same adverse event in one individual counted only once
* For multiple occurrences of the same adverse event maximal intensity is displayed
** For multiple occurrences of the same adverse event 'worst case' relation is displayed
*** For multiple occurrences of the same adverse event outcome of latest AE occurrence is displayed

Source: P132_perfect - AE01T.sas [SVN:29690] Data Extract: 15JUL2016 Generation Date : 23SEP2016 12:45

Table 30 Classification of the AEs during screening per severity, relationship to treatment and outcome by treatment group

	CD133+	Placebo	Total	p-value [1]
Severity*				
no AE	1 (10.0%)	1 (6.3%)	2 (7.7%)	1.0000
Asymptomatic	1 (10.0%)	4 (25.0%)	5 (19.2%)	0.3759
symptomatic, no treatment	1 (10.0%)	2 (12.5%)	3 (11.5%)	1.0000
symptomatic, specific treatment	7 (70.0%)	9 (56.3%)	16 (61.5%)	0.8048
N	10	16	26	0.4233
Relation**				
not related	8 (80.0%)	15 (93.8%)	23 (88.5%)	0.2871
Unlikely	2 (20.0%)	-	2 (7.7%)	0.2257
Possible	-	1 (6.3%)	1 (3.8%)	1.0000
N	10	16	26	0.4233
Outcome***				
recovered without sequelae	8 (80.0%)	14 (87.5%)	22 (84.6%)	0.3849
persisting	2 (20.0%)	1 (6.3%)	3 (11.5%)	0.6069
unknown	-	1 (6.3%)	1 (3.8%)	1.0000
N	10	16	26	0.4233

Note: For patients denominator for percentages is column N.
Note: For severity, relation and outcome denominator for percentages is total number of AEs.
[1] Group comparison using Fishers Exact Test.
Multiple occurrences of the same adverse event in one individual counted only once
* For multiple occurrences of the same adverse event maximal intensity is displayed
** For multiple occurrences of the same adverse event 'worst case' relation is displayed
*** For multiple occurrences of the same adverse event outcome of latest AE occurrence is displayed

Source: P132_perfect - AE01T.sas [SVN:29690] Data Extract: 15JUL2016 Generation Date : 23SEP2016 12:45

Table 31 Summary of adverse events during the main trial phase

	CD133+	Placebo	Total	p-value [1]
pts with at least one AE	37 (100%)	40 (100%)	77 (100%)	
number of AEs	282	311	593	
pts with at least one SAE	19 (51.4%)	15 (37.5%)	34 (44.2%)	0.2563
number of SAEs	25	24	49	
number of Deaths	-	-	-	
pts with at least one MACE	1 (2.7%)	1 (2.5%)	2 (2.6%)	1.0000
number of MACEs	1	1	2	
pts with at least one AESI	32 (86.5%)	31 (77.5%)	63 (81.8%)	0.3822
number of AESIs	67	68	135	
pts with at least one AE that was at least possibly related	2 (5.4%)	7 (17.5%)	9 (11.7%)	0.1564
number of AEs that were at least possibly related	6	13	19	
pts with at least one SAE that was at least possibly related	-	2 (5.0%)	2 (2.6%)	0.4942
number of SAEs that were at least possibly related	-	2	2	
pts with at least one AE that were not or unlikely related	37 (100%)	39 (97.5%)	76 (98.7%)	1.0000
number of AEs that were not or unlikely related	276	298	574	
pts with at least one SAE that was not or unlikely related	19 (51.4%)	14 (35.0%)	33 (42.9%)	0.1720
number of SAEs that were not or unlikely related	25	22	47	
pts with at least one AE leading to withdrawal	1 (2.7%)	-	1 (1.3%)	0.4805
number of AEs leading to withdrawal	1	-	1	

Note: For patients denominator for percentages is column N.
Note: For severity, relation and outcome denominator for percentages is total number of AEs.
[1] Group comparison using Fishers Exact Test.
Multiple occurrences of the same adverse event in one individual counted only once
* For multiple occurrences of the same adverse event maximal intensity is displayed
** For multiple occurrences of the same adverse event 'worst case' relation is displayed
*** For multiple occurrences of the same adverse event outcome of latest AE occurrence is displayed

Source: P132_perfect - AE01T.sas [SVN:29690]   Data Extract: 15JUL2016   Generation Date : 23SEP2016 12:45

Table 32 Classification of the AEs during the main trial phase per severity, relationship to treatment and outcome by treatment group

	CD133+	Placebo	Total	p-value [1]
Severity*				
no AE	-	12 (3.9%)	12 (2.0%)	5.087E-04
asymptomatic	77 (27.3%)	71 (22.8%)	148 (25.0%)	0.2179
symptomatic, no treatment	71 (25.2%)	72 (23.2%)	143 (24.1%)	0.5660
symptomatic, specific treatment	128 (45.4%)	154 (49.5%)	282 (47.6%)	0.3242
life threatening	6 (2.1%)	2 (0.6%)	8 (1.3%)	0.1593
N	282	311	593	
Relation**				
not related	245 (86.9%)	264 (84.9%)	509 (85.8%)	0.5557
unlikely	31 (11.0%)	34 (10.9%)	65 (11.0%)	1.0000
possible	6 (2.1%)	13 (4.2%)	19 (3.2%)	0.1700
N	282	311	593	
Outcome***				
recovered without sequelae	210 (74.5%)	237 (76.2%)	447 (75.4%)	0.6344
recovered with sequelae	10 (3.5%)	10 (3.2%)	20 (3.4%)	0.8247
persisting	38 (13.5%)	47 (15.1%)	85 (14.3%)	0.6391
unknown	24 (8.5%)	17 (5.5%)	41 (6.9%)	0.1490
N	282	311	593	

Note: For patients denominator for percentages is column N.
Note: For severity, relation and outcome denominator for percentages is total number of AEs.
[1] Group comparison using Fishers Exact Test.
Multiple occurrences of the same adverse event in one individual counted only once
* For multiple occurrences of the same adverse event maximal intensity is displayed
** For multiple occurrences of the same adverse event 'worst case' relation is displayed
*** For multiple occurrences of the same adverse event outcome of latest AE occurrence is displayed
Source: P132_perfect - AE01T.sas [SVN:29690]   Data Extract: 15JUL2016   Generation Date : 23SEP2016 12:45

12.1.2 Serious adverse events (including those leading to death)

During the main trial phase, there were 49 SAEs, 25 (15 patients) in the placebo group and 24 (19 patients) in the CD133+ group. There were no statistical differences observed between the placebo and the CD 133+ group neither overall nor in any of the system organ classes. The most common SAEs were cardiac disorders followed by infections and infestations and respiratory, thoracic and mediastinal disorders. Table 33 lists the SAEs by treatment group and system organ class.

Table 33 All Serious Adverse Events during Main Trial Phase by Treatment Group and System Organ Class

	Placebo SAEs / Patients (%)	CD133+ SAEs / Patients (%)	Total SAEs / Patients (%)	p-value [1]
ALL BODY SYSTEMS	24 / 15 (38%)	25 / 19 (51%)	49 / 34 (44%)	0.2563
Cardiac disorders	8 / 7 (18%)	11 / 10 (27%)	19 / 17 (22%)	0.4118
Infections and infestations	7 / 6 (15%)	3 / 3 (8%)	10 / 9 (12%)	0.4837
Respiratory, thoracic and mediastinal disorders	1 / 1 (3%)	3 / 3 (8%)	4 / 4 (5%)	0.3460
General disorders and administration site conditions	-	3 / 3 (8%)	3 / 3 (4%)	0.1062
Injury, poisoning and procedural complications	1 / 1 (3%)	1 / 1 (3%)	2 / 2 (3%)	1.0000
Nervous system disorders	-	2 / 2 (5%)	2 / 2 (3%)	0.2276
Renal and urinary disorders	1 / 1 (3%)	1 / 1 (3%)	2 / 2 (3%)	1.0000
Blood and lymphatic system disorders	1 / 1 (3%)	-	1 / 1 (1%)	1.0000
Eye disorders	1 / 1 (3%)	-	1 / 1 (1%)	1.0000
Gastrointestinal disorders	1 / 1 (3%)	-	1 / 1 (1%)	1.0000
Reproductive system and breast disorders	1 / 1 (3%)	-	1 / 1 (1%)	1.0000
Skin and subcutaneous tissue disorders	1 / 1 (3%)	-	1 / 1 (1%)	1.0000
Surgical and medical procedures	-	1 / 1 (3%)	1 / 1 (1%)	0.4805
Vascular disorders	1 / 1 (3%)	-	1 / 1 (1%)	1.0000

Note: Denominator for percentages is column N. Multiple occurrences of the same adverse event in one individual counted only once
Investigator Term for Adverse Events encoded using MedDRA version 15.0 [1] Group comparison using Fishers Exact Test.

Source: P132_perfect - AE0301T.sas [SVN:29329] Data Extract: 15JUL2016 Generation Date : 09AUG2016 11:30

12.1.2.1 Narratives of the Serious Adverse Events

12.1.2.1.1 Treatment: 5 ml CD133$^+$ cells

There were no SAEs considered related with the treatment in the CD133$^+$ group.

12.1.2.1.2 Treatment: Placebo

There were two SAEs (ventricular tachycardia) considered to be possibly related with the treatment in the placebo group.

Patient [3/30] – SAE (Persistent atrial flutter; ventricular tachycardia); hospitalization

Treatment group: Placebo

Patient details: 55 years, male

Events**:** hospitalization (persistent atrial flutter; ventricular tachycardia)

Patient 3/30 entered the study with coronary artery disease on 08 Nov 2012.

Prior heart catheterization was performed on 20 Sep 2012 and demonstrated critical stenosis or hypoperfused vessels, respectively, of the left anterior descending artery (LAD; ramus interventricularis anterior, RIVA), the RCX and the RCA. Relevant medical (cardiac) history included hypertension, exertional dyspnea, nocturia, dyslipoproteinemia, thrombocytosis and asymptomatic myocardial ischemia. The risk factors comprised, a family disposition of arterial hypertension and a nicotine abuse of 60 pack-years and a smoking Euroscore of 1.33. On 05 Nov 2012, prior to the intervention, the patient was diagnosed with heart failure NYHA class II. The 12 lead electrocardiogram (ECG) performed on 05 Nov 2012 demonstrated sinus rhythm at a heart rate (HR) of 53 beats per minute (bpm), prolonged QT intervals and ST elevations in V2 and V3 and an ST decrease in V5 and V6, respectively, as well as pathologic Q-spikes in II and III. Terminally negative T-waves were seen in II, III and aVF. The Holter ECG performed on 07 Nov 2012 showed 0.3% ventricular extrasystoles (VES) occurring in couplets (LOWN IVa) and no ventricular tachycardia.

Relevant medication at the time of the serious adverse events included metoprolol (hypertension), clopidogrel and acetylsalicylic acid (thrombosis prophylaxis), amiodarone (tachycardia prophylaxis) and simvastatin (hyperlipidemia) at therapeutic dosages.

On 13 Nov 2012, three anastomoses were implanted during the cardiac surgery, bypassing the stenoses of the RIVA, RCX and RCA. During the intervention, 5 ml of the study treatment were administered by means of 25 injections.

Persistent atrial flutter

On 29 Nov 2012, sixteen days post intervention, the patient was hospitalized due to persistent atrial flutter of mild intensity and moderate ventricular tachycardia. An endocardial ablation was performed and the patient had recovered without sequelae by 03 Dec 2012, thereafter continuing study participation as planned. ***The event was evaluated by the investigator as unlikely related to the investigational product.***

Ventricular tachycardia

On 29 Nov 2012, sixteen days post intervention, the patient was hospitalized due to persistent atrial flutter of mild intensity and moderate ventricular tachycardia. The measures taken included an electrophysiological investigation and the patient recovered without sequelae by 03 Dec 2012, thereafter continuing study participation as planned.

The event was evaluated by the investigator as possibly related to the investigational product.

Patient [3/34] – SAE (metastasized colon carcinoma with hepatic metastases; ventricular tachycardia); life threatening (colon carcinoma), prolonged hospitalization (ventricular tachycardia)

Treatment group: CD133$^+$ cells

Patient details: 53 years, male

Events: metastasized colon carcinoma with hepatic metastases (life threatening), prolonged hospitalization (ventricular tachycardia)

Patient 3/34 entered the study with coronary artery disease on 17 Feb 2014.

Prior heart catheterization was performed on 14 Aug 2013 and demonstrated critical stenosis or hypoperfused vessels, respectively, of the left anterior descending artery (LAD; ramus interventricularis

anterior, RIVA) and the RCX. The heart catheterization also revealed hypokinetic and dyskinetic infarction areas of the left ventricle.

Relevant medical (cardiac) history included type 2 diabetes mellitus, arterial hypertension, extrasystolia, hyperlipidemia, adrenal ectomia due to arterial hypertension in 1992, mitral valve regurgitation (grade I) and 60-70% stenosis of the arteria carotis interna. In addition, the patient reported symptomatic myocardial ischemia not requiring specific treatment. Risk factors were limited to previous smoking with 30 pack-years and a smoking Euroscore of 2.59. On 26 Feb 2014. prior to the intervention, the patient was diagnosed with heart failure NYHA class I. The 12-lead electrocardiogram (ECG) performed on 26 Feb 2014 demonstrated sinus rhythm at a HR of 82 bpm, ST elevations in V1, V2 and V3, lack of R-spikes in V2 as well as terminally negative T-waves in V5, V6, I and aVL. The Holter ECG performed on 17 Feb 2014 showed 3.6% VES occurring in couplets (LOWN IVa) and 0.3% supraventricular extrasystoles, respectively, and no ventricular tachycardia.

On 28 Feb 2014, two anastomoses were implanted during the cardiac surgery, bypassing the stenoses of the RIVA and RCA. During the intervention, 5 ml of the study treatment were administered by means of 15 injections.

Ventricular tachycardia

On 06 Mar 2014, six days post-intervention, the patient was diagnosed with asymptomatic ventricular tachycardia of mild intensity with ventricular salves, couples and doublets.

Relevant medication at the time of the serious adverse event included insulin (diabetes mellitus), metamizol (pain at sternal scar post-surgery), torasemide and hydrochlorothiazide (lower leg edema), acetylsalicylic acid (thrombosis prophylaxis) and simvastatin (hyperlipidemia) at therapeutic dosages.

The ventricular tachycardia was treated with carvedilol and kaliumchloride substitution. The control ECG showed only isolated VES, multiple ventricular salves and a mild ventricular tachycardia. To monitor the event and to establish long-term prognosis and treatment, i.e. the need to place an implantable cardioverter defibrillator (ICD), the period of hospitalization was prolonged (starting on 11 Mar 2014). On 13 Mar 2014, the patient was discharged with persisting ventricular tachycardia.

The event was evaluated by the investigator as possibly related to the investigational product.

Metastasized colon carcinoma with hepatic metastases

On 26 Sep 2015, approximately 1.5 years post-intervention, the patient was diagnosed with advanced colon carcinoma and hepatic metastases. The investigator assessed the event as being of moderate severity.

Relevant medication at the time of the serious adverse event included felodipin (arterial hypertension), carvedilol (ventricular tachycardia), acetylsalicylic acid (thrombosis prophylaxis), vildagliptin and metformin hydrochloride (diabetes mellitus) and simvastatin (hyperlipidemia) at therapeutic dosages.

The concomitant stenosis of the colon was treated by placement of a stent into the left colic flexure on 26 Sep 2015. On 28 Sep 2015, a colon perforation occurred at the cecum resulting in fecal peritonitis. On the same day, the colon perforation was treated with an emergency laparotomy and the placement of a terminal ileostoma. On 01 Oct 2015, the patient was re-laparotomized and a peritoneal lavage was performed. Subsequently, chemotherapy was initiated. The colon carcinoma currently persists.

The event was evaluated by the investigator as unlikely related to the investigational product.

12.1.3 Other significant adverse events

There were a set of AEs that were considered Adverse Events of Specific Interest (AESIs), since these could be directly related to the intervention and the active treatment. These were:

- AV-block (I, II or III)
- Prolonged QT interval
- Sinus bradycardia
- Supraventricular arrhythmia
- Ventricular arrhythmia
- Vasovagal syncope
- Left ventricular failure
- Myocardial ischemia
- Cerebral ischemia
- Myocarditis
- Pericardial Effusion
- Pericarditis
- Deep sternal wound infection (or wound infection at the site of graft sampling) coded as “deep postoperative wound infection” (Meddra LLT 10074392).

There were 135 AESIs observed, these were evenly distributed in both the control and the CD 133+ groups. No statistically significant differences could be observed between both treatment groups.

Table 34 AESIs as defined in the SAP and how they were coded for the analysis

**AESI**	**Coded** (MedDRA version 15.0)	**Placebo**	**CD133+**	**Total**	**p-value [1]**
AV block I, II or III		2 (6%)	3 (8%)	5 (7%)	
	Atrioventricular block	-	2 (5%)	2 (3%)	0.2276
	Atrioventricular block first degree	1 (3%)	1 (3%)	2 (3%)	1.0000
	Atrioventricular block complete	1 (3%)	-	1 (1%)	1.0000
Prolonged QT interval		3 (8%)	1 (3%)	4 (5%)	
	Electrocardiogram QT prolonged	3 (8%)	1 (3%)	4 (5%)	0.6161
Sinus bradycardia		4 (10%)	1 (3%)	5 (6%)	
	Bradycardia	4 (10%)	1 (3%)	5 (6%)	0.3602
Supraventricular arrhythmia		16 (41%)	19 (52%)	35 (45%)	
	Atrial fibrillation	9 (23%)	9 (24%)	18 (23%)	1.0000
	Supraventricular tachyarrhythmia	2 (5%)	3 (8%)	5 (6%)	0.6670
	Arrhythmia supraventricular	2 (5%)	1 (3%)	3 (4%)	1.0000
	Supraventricular extrasystoles	2 (5%)	1 (3%)	3 (4%)	1.0000
	Supraventricular tachycardia	-	3 (8%)	3 (4%)	0.1062
	Atrial flutter	1 (3%)	1 (3%)	2 (3%)	1.0000
	Sinus tachycardia	-	1 (3%)	1 (1%)	0.4805
Ventricular arrhythmia		8 (21%)	6 (16%)	14 (17%)	
	Ventricular arrhythmia	2 (5%)	2 (5%)	4 (5%)	1.0000
	Ventricular extrasystoles	2 (5%)	2 (5%)	4 (5%)	1.0000
	Ventricular tachycardia	3 (8%)	-	3 (4%)	0.2413
	Tachyarrhythmia	1 (3%)	-	1 (1%)	1.0000
	Ventricular fibrillation	-	1 (3%)	1 (1%)	0.4805
	Ventricular flutter	-	1 (3%)	1 (1%)	0.4805
Vasovagal syncope		1 (3%)	1 (3%)	2 (3%)	
	Syncope	1 (3%)	1 (3%)	2 (3%)	1.0000
Left ventricular failure		2 (6%)	3 (8%)	5 (7%)	
	Cardiovascular insufficiency	1 (3%)	1 (3%)	2 (3%)	1.0000
	Cardiac failure	1 (3%)	2 (5%)	3 (4%)	0.6055
Myocardial ischemia		1 (3%)	2 (5%)	3 (4%)	
	Acute myocardial infarction	1 (3%)	-	1 (1%)	1.0000
	Angina pectoris	-	2 (5%)	2 (3%)	0.2276
Cerebral ischemia		-	2 (6%)	2 (6%)	
	Cerebral infarction	-	1 (3%)	1 (1%)	0.4805
	Cerebrovascular accident	-	1 (3%)	1 (1%)	0.4805
Myocarditis		-	-	-	-
Pericardial effusion		8 (20%)	6 (16%)	14 (18%)	
	Pericardial effusion	8 (20%)	6 16%)	14 (18%)	0.7715
Pericarditis		-	-	-	-
Deep sternal wound infection		-	-	-	-

AESI	**Coded** (MedDRA version 15.0)	Placebo	CD133+	Total	p-value [1]
Other arrhythmias		4 (11%)	3 (8%)	7 (9%)	
	Cardiac arrest	1 (3%)	-	1 (1%)	1.0000
	Bradyarrhythmia	1 (3%)	-	1 (1%)	1.0000
	Tachycardia	2 (5%)	1 (3%)	3 (4%)	1.0000
	Arrhythmia	-	2 (5%)	2 (3%)	0.2276
Total		67	68	135	

Multiple occurrences of the same adverse event in one individual counted only once. Denominator for percentages is column N.
[1] Group comparison using Fishers Exact Test.
Source: P132_perfect - AE0301T.sas [SVN:29329] Data Extract: 15JUL2016 Generation Date : 09AUG2016 11:29

12.2 Clinical laboratory evaluation

12.2.1 Haematology

Table 35 summarizes the haematology values at Visit I and Visit V per treatment group. No statistical tests were performed to analyse differences between the two treatment groups.

Table 36 summarizes the new abnormalities in the haematology values detected after the intervention and until Visit V per treatment group. No statistical tests were performed to analyse differences between the two treatment groups.

Table 35 Haematology values at Visit I and Visit V by treatment group

		Placebo	CD133+	Total
Haemoglobin				
Visit I	< lower range	15 (37.5%)	11 (29.7%)	26 (33.8%)
	within normal range	25 (62.5%)	26 (70.3%)	51 (66.2%)
Visit V	< lower range	14 (35.0%)	11 (31.4%)	25 (33.3%)
	within normal range	25 (62.5%)	23 (65.7%)	48 (64.0%)
Leukocytes				
Visit I	> upper range	6 (15.0%)	6 (16.2%)	12 (15.6%)
	within normal range	34 (85.0%)	31 (83.8%)	65 (84.4%)
Visit V	> upper range	8 (20.0%)	2 (5.7%)	10 (13.3%)
	within normal range	31 (77.5%)	32 (91.4%)	63 (84.0%)
Thrombocytes				
Visit I	< lower range	1 (2.5%)	3 (8.1%)	4 (5.2%)
	> upper range	2 (5.0%)	-	2 (2.6%)
	within normal range	37 (92.5%)	34 (91.9%)	71 (92.2%)
Visit V	< lower range	-	3 (8.6%)	3 (4.0%)
	> upper range	2 (5.0%)	-	2 (2.7%)
	within normal range	37 (92.5%)	31 (88.6%)	68 (90.7%)

Source: P132_perfect - LAB01T.sas [SVN:27969] Data Extract: 15JUL2016 Generation Date : 09AUG2016 11:31

Table 36 New abnormalities in the haematology values at Visit V by treatment groups

		Placebo	CD133+	Total
Hemoglobin				
Visit V	new abnormality	3 (7.5%)	6 (17.1%)	9 (12.0%)
	no new abnormality	36 (90.0%)	28 (80.0%)	64 (85.3%)
Leukocytes				
Visit V	new abnormality	4 (10.0%)	1 (2.9%)	5 (6.7%)
	no new abnormality	35 (87.5%)	33 (94.3%)	68 (90.7%)
Thrombocytes				
Visit V	no new abnormality	39 (97.5%)	34 (97.1%)	73 (97.3%)

Source: P132_perfect - LAB02T.sas [SVN:28047] Data Extract: 15JUL2016 Generation Date : 09AUG2016 11:32

12.2.2 Blood chemistry

Table 37 to Table 39 summarize the blood chemistry values at Visit I and Visit V per treatment group. No statistical tests were performed to analyse differences between the two treatment groups.

Table 37 Blood chemistry values at Visit I and Visit V by treatment group

		Placebo	CD133+	Total
C-reactive protein				
Visit I	> upper range	10 (25.0%)	10 (27.0%)	20 (26.0%)
	within normal range	30 (75.0%)	26 (70.3%)	56 (72.7%)
Visit V	> upper range	8 (20.0%)	7 (20.0%)	15 (20.0%)
	within normal range	28 (70.0%)	25 (71.4%)	53 (70.7%)
Creatinine				
Visit I	< lower range	1 (2.5%)	1 (2.7%)	2 (2.6%)
	> upper range	7 (17.5%)	11 (29.7%)	18 (23.4%)
	within normal range	32 (80.0%)	25 (67.6%)	57 (74.0%)
Visit V	< lower range	-	1 (2.9%)	1 (1.3%)
	> upper range	8 (20.0%)	12 (34.3%)	20 (26.7%)
	within normal range	31 (77.5%)	19 (54.3%)	50 (66.7%)
Creatine Kinase				
Visit I	> upper range	5 (12.5%)	3 (8.1%)	8 (10.4%)
	within normal range	35 (87.5%)	33 (89.2%)	68 (88.3%)
Visit V	> upper range	5 (12.5%)	4 (11.4%)	9 (12.0%)
	within normal range	34 (85.0%)	30 (85.7%)	64 (85.3%)
Creatine Kinase MB				
Visit I	> upper range	9 (22.5%)	9 (24.3%)	18 (23.4%)
	within normal range	19 (47.5%)	17 (45.9%)	36 (46.8%)
Visit V	> upper range	12 (30.0%)	12 (34.3%)	24 (32.0%)
	within normal range	19 (47.5%)	15 (42.9%)	34 (45.3%)
Troponin T				
Visit I	> upper range	7 (17.5%)	10 (27.0%)	17 (22.1%)
	within normal range	27 (67.5%)	17 (45.9%)	44 (57.1%)

		Placebo	CD133+	Total
Visit V	> upper range	3 (7.5%)	7 (20.0%)	10 (13.3%)
	within normal range	33 (82.5%)	24 (68.6%)	57 (76.0%)
Sodium				
Visit I	< lower range	9 (22.5%)	5 (13.5%)	14 (18.2%)
	> upper range	-	-	-
	within normal range	31 (77.5%)	32 (86.5%)	63 (81.8%)
Visit V	< lower range	6 (15.0%)	1 (2.9%)	7 (9.3%)
	> upper range	-	-	-
	within normal range	33 (82.5%)	33 (94.3%)	66 (88.0%)
Potassium				
Visit I	< lower range	-	1 (2.7%)	1 (1.3%)
	> upper range	2 (5.0%)	-	2 (2.6%)
	within normal range	38 (95.0%)	36 (97.3%)	74 (96.1%)
Visit V	< lower range	2 (5.0%)	1 (2.9%)	3 (4.0%)
	> upper range	4 (10.0%)	-	4 (5.3%)
	within normal range	33 (82.5%)	33 (94.3%)	66 (88.0%)

Source: P132_perfect - LAB01T.sas [SVN:27969]   Data Extract: 15JUL2016   Generation Date : 09AUG2016 11:31

Table 38 Blood lipids at Visit I by treatment group

		Placebo	CD133+	Total
Total cholesterol				
Visit I	< lower range	1 (2.5%)	1 (2.7%)	2 (2.6%)
	> upper range	5 (12.5%)	6 (16.2%)	11 (14.3%)
	within normal range	32 (80.0%)	25 (67.6%)	57 (74.0%)
LDL cholesterol				
Visit I	< lower range	1 (2.5%)	1 (2.7%)	2 (2.6%)
	> upper range	3 (7.5%)	4 (10.8%)	7 (9.1%)
	within normal range	30 (75.0%)	27 (73.0%)	57 (74.0%)
HDL cholesterol				
Visit I	< lower range	9 (22.5%)	15 (40.5%)	24 (31.2%)
	> upper range	1 (2.5%)	-	1 (1.3%)
	within normal range	24 (60.0%)	17 (45.9%)	41 (53.2%)
Triglycerides				
Visit I	> upper range	10 (25.0%)	6 (16.2%)	16 (20.8%)
	within normal range	28 (70.0%)	25 (67.6%)	53 (68.8%)

Source: P132_perfect - LAB01T.sas [SVN:27969]   Data Extract: 15JUL2016   Generation Date : 09AUG2016 11:31

Table 39 NT-pro-BNP- at Visit I and Visit V by treatment group

		Placebo	CD133+	Total
NT-pro-BNP				
Visit I	> upper range	36 (90.0%)	35 (94.6%)	71 (92.2%)
	within normal range	2 (5.0%)	-	2 (2.6%)
Visit V	> upper range	35 (87.5%)	31 (88.6%)	66 (88.0%)
	within normal range	2 (5.0%)	1 (2.9%)	3 (4.0%)

Source: P132_perfect - LAB01T.sas [SVN:27969]   Data Extract: 15JUL2016   Generation Date : 09AUG2016 11:31

Table 40 summarizes the new abnormalities in the blood chemistry values detected after the intervention and until Visit V per treatment group. No statistical tests were performed to analyse differences between the two treatment groups.

Table 40 New abnormalities in the blood chemistry values at Visit V by treatment groups

		Placebo	CD133+	Total
C-reactive protein				
Visit V	new abnormality	6 (15.0%)	2 (5.7%)	8 (10.7%)
	no new abnormality	30 (75.0%)	30 (85.7%)	60 (80.0%)
Creatinine				
Visit V	new abnormality	4 (10.0%)	6 (17.1%)	10 (13.3%)
	no new abnormality	35 (87.5%)	26 (74.3%)	61 (81.3%)
Creatine Kinase				
Visit V	new abnormality	2 (5.0%)	3 (8.6%)	5 (6.7%)
	no new abnormality	37 (92.5%)	30 (85.7%)	67 (89.3%)
Creatine Kinase MB				
Visit V	new abnormality	3 (7.5%)	4 (11.4%)	7 (9.3%)
	no new abnormality	25 (62.5%)	21 (60.0%)	46 (61.3%)
Troponin T				
Visit V	new abnormality	-	2 (5.7%)	2 2.7%)
	no new abnormality	32 (80.0%)	23 (65.7%)	55 (73.3%)
Sodium				
Visit V	new abnormality	2 (5.0%)	1 (2.9%)	3 (4.0%)
	no new abnormality	37 (92.5%)	33 (94.3%)	70 (93.3%)
Potassium				
Visit V	new abnormality	6 (15.0%)	1 (2.9%)	7 (9.3%)
	no new abnormality	33 (82.5%)	33 (94.3%)	66 (88.0%)
Nt-proBNP				
Visit V	new abnormality	1 (2.5%)	-	1 (1.3%)
	no new abnormality	34 (85.0%)	30 (85.7%)	64 (85.3%)
	MISSING	5 (12.5%)	5 (14.3%)	10 (13.3%)
	N	40	35	75

Note: Denominator for percentages is column N.

Source: P132_perfect - LAB02T.sas [SVN:28047] Data Extract: 15JUL2016 Generation Date : 09AUG2016 11:32

12.2.3 Urinalysis

NA

12.3 Other safety data

12.3.1 Vital signs

The following vital signs were assessed in accordance with the Schedule of Assessments (Table 2)

- Supine blood pressure in mm/Hg

- Classification of blood pressure according to the ESC/ESH guidelines 2007[2]
- Resting pulse rate in beats/min
- Body temperature in °C (aural)
- Body weight in kg
- Respiratory rate in breaths/min
- Examination of head, neck, thorax, abdomen, neurological system, musculoskeletal system, skin, others (except heart)

Table 41 summarizes the vital signs values at Visit V and the difference vs. Visit I per treatment group.

There were no statistical tests performed to analyse differences between the treatment groups and there were no vital signs values that were unexpected and should be reported in detail.

The results of the physical examination of head, neck, thorax, abdomen, neurological system, musculoskeletal system, skin, and others are listed in in PHYSEX01T: Physical Examination - Vital Signs and Physical Examination (Appendix B) and there were no unexpected findings that should be reported in detail.

[2] No table was provided for this endpoint. Listing available in: P132_perfect - DL04L.sas [SVN:29185] Data Extract: 15JUL2016 Generation Date : 09AUG2016 11:42

Table 41 Vital signs at Visit V and the difference vs. Visit I per treatment group.

	Values at Visit V						Difference vs. Visit I (Screening)					
**Systolic BP mmHg**	**n**	**Mean**	**Std**	**Median**	**Q1 - Q3**	**Min - Max**	**n**	**Mean**	**Std**	**Median**	**Q1 - Q3**	**Min - Max**
Placebo (N=40)	39	131.5	17.10	135.0	120 - 145	75 - 165	38	1.2	19.89	0.5	-10 - 15	-55 - 38
CD 133 + (N=37)	33	130.4	20.07	130.0	120 - 140	95 - 195	33	4.8	19.65	5.0	-7 - 15	-42 - 53
Total (N=77)	72	131.0	18.39	130.0	120 - 140	75 - 195	71	2.9	19.72	1.0	-10 - 15	-55 - 53
**Diastolic BP mmHg**												
Placebo (N=40)	39	77.1	9.69	80.0	70 - 85	50 - 93	38	2.5	12.65	1.5	-4 - 10	-29 - 29
CD 133 + (N=37)	33	77.8	11.16	80.0	70 - 80	60 - 119	33	2.5	10.84	0.0	-5 - 12	-23 - 21
Total (N=77)	72	77.4	10.32	80.0	70 - 80	50 - 119	71	2.5	11.76	0.0	-5 - 10	-29 - 29
**Resting PR [beats/min]**												
Placebo (N=40)	39	69.7	13.10	68.0	60 - 79	49 - 99	37	-3.1	14.83	-2.0	-10 - 9	-52 - 21
CD 133 + (N=37)	32	72.1	10.28	70.0	64 - 80	56 - 94	31	-2.3	14.00	-2.0	-11 - 7	-33 - 25
Total (N=77)	71	70.8	11.89	69.0	64 - 79	49 - 99	68	-2.7	14.36	-2.0	-11 - 9	-52 - 25
**Body temperature [°C]**												
Placebo (N=40)	32	36.22	0.512	36.20	36.0 - 36.6	35.0 - 37.4	32	-0.12	0.561	0.00	-0.6 - 0.1	-1.2 - 1.4
CD 133 + (N=37)	30	36.28	0.389	36.25	36.1 - 36.4	35.0 - 37.2	30	-0.04	0.575	0.00	-0.6 - 0.4	-1.1 - 1.1
Total (N=77)	62	36.25	0.454	36.20	36.0 - 36.5	35.0 - 37.4	62	-0.08	0.564	0.00	-0.6 - 0.2	-1.2 - 1.4
**Body weight [kg]**												
Placebo (N=40)	39	89.2	15.72	90.0	78 - 99	53 - 125	39	-1.3	5.42	-1.0	-5 - 2	-12 - 10
CD 133 + (N=37)	33	89.9	15.68	88.0	79 - 100	58 - 120	33	1.6	5.63	3.0	-2 - 5	-11 - 10
Total (N=77)	72	89.5	15.60	89.5	79 - 100	53 - 125	72	0.0	5.67	0.5	-3 - 5	-12 - 10
**Respiratory rate [breaths/min]**												
Placebo (N=40)	18	15.7	1.97	16.0	14 - 16	13 - 20	13	-0.4	4.25	0.0	0 - 2	-13 - 4
CD 133 + (N=37)	16	16.5	3.56	15.5	14 - 19	13 - 26	14	1.7	3.97	1.0	-1 - 3	-3 - 12
Total (N=77)	34	16.1	2.82	16.0	14 - 16	13 - 26	27	0.7	4.17	0.0	-1 - 2	-13 - 12

Source: P132_perfect - PHYSEX01T.sas [SVN:29178]   Data Extract: 15JUL2016   Generation Date : 09AUG2016 11:32

12.3.2 Unwanted tissue changes

No unwanted tissue changes were detected following the IMP/placebo injections. There was one patient (Patient ID 02-035) in whom a small nodule was detected with echocardiography in the right apical lung at Visit I. This finding was confirmed at Visit III and Visit V in the MRI scans. The patient was randomized to the CD 133+ treatment group.

12.4 Safety conclusions

Since:

- in the AEs analysis, there were no statistical differences between the placebo (311 AEs) and the CD133+ group (282 AEs), neither overall nor in any of the categories in which the patients or AEs were classified;

- in the SAEs analysis, there were no statistical differences observed between the placebo (24 (SAEs) and the CD 133+ group (25 SAEs) neither overall nor in any of the system organ classes;
- the two SAEs classified as possibly related with the treatment occurred in the placebo treatment group;
- in the AESIs analysis no statistically significant differences could be observed between both treatment groups indicating that the AESIs were related either with the CABG surgery or the underlying disease (67 AESIs in the placebo group and 68 AESIs in the treatment group); and
- there were no unexpected or relevant findings in the laboratory values and vital signs;

it can be concluded that the active treatment with CD 133+ did not have a different safety profile when compared to the treatment with placebo.

13 DISCUSSION AND CONCLUSIONS

Phase I and II pilot studies reported in the literature using CD133^{+} cells isolated using the CliniMACS system showed that transepicardial injections have beneficial effects because of the cell transplantation. This was demonstrated by improvements in global LV function or perfusion. Thus, the primary objective of this trial was to show whether injection of autologously derived bone marrow stem cells yielded a functional benefit in addition to the CABG surgery as determined by LVEF measured by MRI.

Prior to the final analysis on all 82 enrolled patients, an interim analysis was performed with 70 patients completing the 6 month follow-up.

In this interim analysis, the statistical analysis (ANCOVA) of the primary endpoint LVEF at 6 months showed a significant group difference of 4.5% between placebo and CD133+ with a greater mean value for the CD133+ group.

A difference of 4.5% in LVEF is of clinical significance for patients who have previously suffered a myocardial infarction. Trials on large patient populations indicate a clear correlation between LVEF and survival as reported by Solomon et al. 2005 (20) on 14,609 patients following a myocardial infarction. In the early post-infarction period, a decrease of 5% in the LVEF was associated with a 21% increase in the risk of sudden death. The absolute risk of sudden death was highest within the first year of myocardial infarction.

Figure 5: Rates of sudden death/cardiac arrest according to LVEF (Solomon et al.)

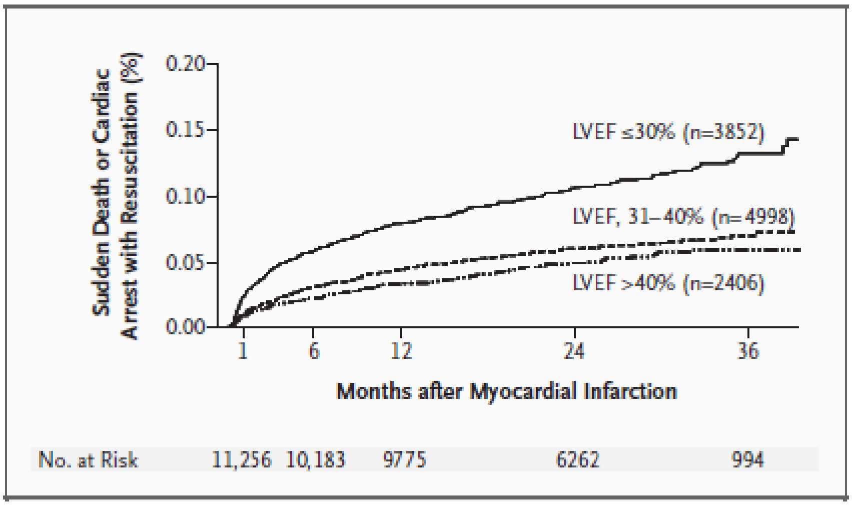

In an older publication (21) on 10,219 survivors of myocardial infarction a link between the LVEF and survival could also be shown. The LVEF- mortality curve exhibited a hyperbolic trend with an upturn in mortality occuring at values of less than 40% (Figure 6).

Figure 6: 6-month all-cause mortality in five categories of LVEF (Volpi et al, 1993)

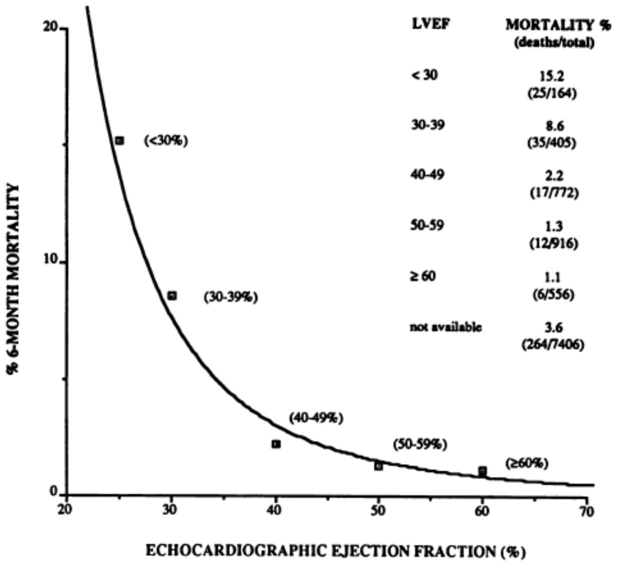

However, in the final analysis of the primary endpoint the value was lower than the one obtained in the interim analysis, showing a difference in LVEF between visit I and III of 0.7077 (p=0.8130) and between visit I and V of 2.2202 (p=0.4454) according to ANCOVA. The results of the echocardiography showed a mean difference of 2.4 between the groups after 6 months following the administration of the CD133+ cells or placebo.

Similar results were found by Nasseri et al., 2014 (22) in a clinical trial using transepicardial injection of isolated CD133$^+$ cells in 60 patients with chronic ischaemic heart disease. The study could not show a clinical benefit in contrast to placebo. It should be mentioned though, that the patient populations, trial design as well as IMP composition and method of application were different.

Other secondary endpoints that did not undergo statistical tests to demonstrate efficacy showed some positive trends. These included the unadjusted values of LVEF measured by MRI, which revealed a larger increase in the patients treated with CD133+ (3.4%) and the mean values of scar tissue and non-viable tissue in the PPS measured by MRI, which were less in the CD133+ group than in the placebo group.

In order to evaluate the study results it should be mentioned that the PERFECT study was designed to include a larger number of patients (142), but it had to be stopped due to slow recruitment. This not only limited the study population but also affected the power of the results.

In the PERFECT trial, the two MACEs occurred in 2 (2.4%) patients, affecting one patient in the placebo group and one in the CD133+ group. Both MACEs were classified as ventricular arrhythmias. Actual data published in the SYNTAX-trial show a MACCE-rate in CABG-surgery of 9.9% (85 events in 860 patients) in a 6 month period after surgery (23). Thus, the safety data in the PERFECT trial is well in line with published data in large cohorts. Furthermore, the PERFECT trial was supervised by an independent SMB, which came to the same conclusion.

Similar results were observed in the phase I and II pilot studies mentioned above: transepicardial injections have a high safety profile. There were no procedure-related complications reported for up to 5 years postoperatively, in particular no increased ventricular arrhythmia or neoplasia.

In conclusion, the procedure was demonstrated to be safe, showing a low incidence of SAEs and MACEs when compared to the SAEs and MACEs in other trials.

Overall, the LVEF increase was clinically significant (~9%) in both the placebo and treatment group, however the study could not demonstrate a positive effect of the CD133 injection in the LVEF 6 months after surgery.

14 TABLES, FIGURES, AND GRAPHS REFERRED TO BUT NOT INCLUDED IN THE TEXT

Table 42 EQ-5D - anxiety/depression - Difference between level at 6 months post-OP to screening and 3-months post-OP

	Placebo	CD133+	Not treated	Total
Difference between Visit V (6-Month Follow-Up) and Visit I (Screening)				
N	28	25	0	53
Mean	0.0	-0.1	-	-0.1
Std	0.47	0.44	-	0.46
Median	0.0	0.0	-	0.0
Q1 - Q3	0 - 0	0 - 0	-	0 - 0
Min - Max	-1 - 1	-1 - 1	-	-1 - 1
Difference between Visit V (6-Month Follow-Up) and Visit IV (3-Month Follow-Up)				
N	29	25	0	54
Mean	-0.1	0.0	-	-0.0
Std	0.46	0.50	-	0.47
Median	0.0	0.0	-	0.0
Q1 - Q3	0 - 0	0 - 0	-	0 - 0
Min - Max	-1 - 1	-1 - 1	-	-1 - 1

Source: P132_perfect - EQ5D01T.sas [SVN:27384] Data Extract: 15JUL2016 Generation Date : 09AUG2016 11:25

Table 43 EQ-5D – pain/discomfort - Difference between level at 6 months post-OP to screening and 3-months post-OP

	Placebo	CD133+	Not treated	Total
Difference between Visit V (6-Month Follow-Up) and Visit I (Screening)				
N	28	25	0	53
Mean	-0.0	0.1	-	0.0
Std	0.64	0.81	-	0.72
Median	0.0	0.0	-	0.0
Q1 - Q3	0 - 0	0 - 1	-	0 - 0
Min - Max	-1 - 1	-2 - 1	-	-2 - 1
Difference between Visit V (6-Month Follow-Up) and Visit IV (3-Month Follow-Up)				
N	30	25	0	55
Mean	-0.0	-0.2	-	-0.1
Std	0.61	0.37	-	0.52
Median	0.0	0.0	-	0.0
Q1 - Q3	0 - 0	0 - 0	-	0 - 0
Min - Max	-1 - 1	-1 - 0	-	-1 - 1

Source: P132_perfect - EQ5D01T.sas [SVN:27384] Data Extract: 15JUL2016 Generation Date : 09AUG2016 11:25

Table 44 EQ-5D – self-care - Difference between level at 6 months post-OP to screening and 3-months post-OP

	Placebo	CD133+	Not treated	Total
Difference between Visit V (6-Month Follow-Up) and Visit I (Screening)				
N	28	25	0	53
Mean	0.0	-0.0	-	-0.0
Std	0.00	0.35	-	0.24
Median	0.0	0.0	-	0.0
Q1 - Q3	0 - 0	0 - 0	-	0 - 0
Min - Max	0 - 0	-1 - 1	-	-1 - 1
Difference between Visit V (6-Month Follow-Up) and Visit IV (3-Month Follow-Up)				
N	30	25	0	55
Mean	-0.0	0.0	-	-0.0
Std	0.18	0.29	-	0.23
Median	0.0	0.0	-	0.0
Q1 - Q3	0 - 0	0 - 0	-	0 - 0
Min - Max	-1 - 0	-1 - 1	-	-1 - 1

Source: P132_perfect - EQ5D01T.sas [SVN:27384] Data Extract: 15JUL2016 Generation Date : 09AUG2016 11:25

Table 45 EQ-5D – usual activities - Difference between level at 6 months post-OP to screening and 3-months post-OP

	Placebo	CD133+	Not treated	Total
Difference between Visit V (6-Month Follow-Up) and Visit I (Screening)				
N	28	25	0	53
Mean	0.0	-0.2	-	-0.1
Std	0.51	0.58	-	0.55
Median	0.0	0.0	-	0.0
Q1 - Q3	0 - 0	-1 - 0	-	0 - 0
Min - Max	-1 - 1	-1 - 1	-	-1 - 1
Difference between Visit V (6-Month Follow-Up) and Visit IV (3-Month Follow-Up)				
N	29	25	0	54
Mean	0.1	-0.2	-	-0.1
Std	0.46	0.52	-	0.51
Median	0.0	0.0	-	0.0
Q1 - Q3	0 - 0	-1 - 0	-	0 - 0
Min - Max	-1 - 1	-1 - 1	-	-1 - 1

Source: P132_perfect - EQ5D01T.sas [SVN:27384] Data Extract: 15JUL2016 Generation Date : 09AUG2016 11:25

Table 46 SF36 – Bodily Pain (BP) - Difference between level at 6 months post-OP to screening and 3-months post-OP

	Placebo	CD133+	Not treated	Total
Difference between Visit V (6-Month Follow-Up) and Visit I (Screening)				
N	36	29	0	65
Mean	6.18	-4.14	-	1.58
Std	28.040	39.110	-	33.554
Median	0.00	0.00	-	0.00
Q1 - Q3	-12.5 - 22.5	-25.0 - 22.5	-	-20.0 - 22.5
Min - Max	-35.0 - 90.0	-100.0 - 77.5	-	-100.0 - 90.0
Difference between Visit V (6-Month Follow-Up) and Visit IV (3-Month Follow-Up)				
N	37	29	0	66
Mean	9.09	8.62	-	8.88
Std	27.292	26.037	-	26.545
Median	0.00	0.00	-	0.00
Q1 - Q3	0.0 - 22.5	0.0 - 22.5	-	0.0 - 22.5
Min - Max	-90.0 - 67.5	-47.5 - 67.5	-	-90.0 - 67.5

Scales and Summary Measures only calculated if all contributing items are available

Source: P132_perfect - SF3601T.sas [SVN:27384] Data Extract: 15JUL2016 Generation Date : 09AUG2016 11:26

Table 47 SF36 – General Health (GH) - Difference between level at 6 months post-OP to screening and 3-months post-OP

	Placebo	CD133+	Not treated	Total
Difference between Visit V (6-Month Follow-Up) and Visit I (Screening)				
N	36	29	0	65
Mean	6.86	6.51	-	6.71
Std	17.967	21.747	-	19.582
Median	8.75	5.00	-	5.00
Q1 - Q3	-5.0 - 20.0	-5.0 - 20.0	-	-5.0 - 20.0
Min - Max	-35.0 - 50.0	-35.0 - 45.0	-	-35.0 - 50.0
Difference between Visit V (6-Month Follow-Up) and Visit IV (3-Month Follow-Up)				
N	37	29	0	66
Mean	5.03	3.71	-	4.45
Std	15.697	19.760	-	17.467
Median	5.00	0.00	-	5.00
Q1 - Q3	-5.0 - 15.0	-5.0 - 12.5	-	-5.0 - 15.0
Min - Max	-20.0 - 40.0	-30.0 - 40.0	-	-30.0 - 40.0

Scales and Summary Measures only calculated if all contributing items are available

Source: P132_perfect - SF3601T.sas [SVN:27384] Data Extract: 15JUL2016 Generation Date : 09AUG2016 11:26

Table 48 SF36 – Mental Health (MH) - Difference between level at 6 months post-OP to screening and 3-months post-OP

	Placebo	CD133+	Not treated	Total
Difference between Visit V (6-Month Follow-Up) and Visit I (Screening)				
N	36	29	0	65
Mean	8.17	5.24	-	6.86
Std	19.563	18.673	-	19.079
Median	4.00	8.00	-	4.00
Q1 - Q3	-4.0 - 20.0	-8.0 - 20.0	-	-4.0 - 20.0
Min - Max	-44.0 - 60.0	-32.0 - 44.0	-	-44.0 - 60.0
Difference between Visit V (6-Month Follow-Up) and Visit IV (3-Month Follow-Up)				
N	37	29	0	66
Mean	4.32	2.21	-	3.39
Std	14.678	15.805	-	15.102
Median	4.00	0.00	-	0.00
Q1 - Q3	-4.0 - 12.0	-4.0 - 8.0	-	-4.0 - 12.0
Min - Max	-20.0 - 32.0	-36.0 - 40.0	-	-36.0 - 40.0

Scales and Summary Measures only calculated if all contributing items are available

Source: P132_perfect - SF3601T.sas [SVN:27384] Data Extract: 15JUL2016 Generation Date : 09AUG2016 11:26

Table 49 SF36 – Physical Functioning (PH) - Difference between level at 6 months post-OP to screening and 3-months post-OP

	Placebo	CD133+	Not treated	Total
Difference between Visit V (6-Month Follow-Up) and Visit I (Screening)				
N	36	28	0	64
Mean	12.70	8.77	-	10.98
Std	30.990	28.335	-	29.690
Median	10.00	7.78	-	10.00
Q1 - Q3	-0.6 - 25.0	-12.5 - 30.0	-	-10.0 - 30.0
Min - Max	-51.4 - 75.0	-50.0 - 70.0	-	-51.4 - 75.0
Difference between Visit V (6-Month Follow-Up) and Visit IV (3-Month Follow-Up)				
N	37	28	0	65
Mean	9.14	1.43	-	5.82
Std	16.659	20.178	-	18.512
Median	5.00	-2.50	-	5.00
Q1 - Q3	0.0 - 18.3	-10.0 - 12.5	-	-5.0 - 15.0
Min - Max	-35.0 - 55.0	-40.0 - 45.0	-	-40.0 - 55.0

Scales and Summary Measures only calculated if all contributing items are available

Source: P132_perfect - SF3601T.sas [SVN:27384] Data Extract: 15JUL2016 Generation Date : 09AUG2016 11:26

Table 50 SF36 – Role Emotional (RE) - Difference between level at 6 months post-OP to screening and 3-months post-OP

	Placebo	CD133+	Not treated	Total
Difference between Visit V (6-Month Follow-Up) and Visit I (Screening)				
N	36	29	0	65
Mean	-10.65	-17.24	-	-13.59
Std	44.925	53.170	-	48.492
Median	0.00	0.00	-	0.00
Q1 - Q3	-33.3 - 0.0	-33.3 - 0.0	-	-33.3 - 0.0
Min - Max	-100.0 - 66.7	-100.0 - 66.7	-	-100.0 - 66.7
Difference between Visit V (6-Month Follow-Up) and Visit IV (3-Month Follow-Up)				
N	37	29	0	66
Mean	-18.02	-2.30	-	-11.11
Std	49.438	52.653	-	51.085
Median	0.00	0.00	-	0.00
Q1 - Q3	-33.3 - 0.0	-33.3 - 0.0	-	-33.3 - 0.0
Min - Max	-100.0 - 100.0	-100.0 - 100.0	-	-100.0 - 100.0

Scales and Summary Measures only calculated if all contributing items are available

Source: P132_perfect - SF3601T.sas [SVN:27384] Data Extract: 15JUL2016 Generation Date : 09AUG2016 11:26

Table 51 SF36 – Role Physical (RP) - Difference between level at 6 months post-OP to screening and 3-months post-OP

	Placebo	CD133+	Not treated	Total
Difference between Visit V (6-Month Follow-Up) and Visit I (Screening)				
N	36	29	0	65
Mean	-20.14	-18.10	-	-19.23
Std	49.577	45.265	-	47.345
Median	-12.50	0.00	-	0.00
Q1 - Q3	-50.0 - 0.0	-50.0 - 0.0	-	-50.0 - 0.0
Min - Max	-100.0 - 100.0	-100.0 - 100.0	-	-100.0 - 100.0
Difference between Visit V (6-Month Follow-Up) and Visit IV (3-Month Follow-Up)				
N	37	29	0	66
Mean	-17.57	-12.93	-	-15.53
Std	37.669	41.523	-	39.166
Median	0.00	0.00	-	0.00
Q1 - Q3	-25.0 - 0.0	-50.0 - 0.0	-	-50.0 - 0.0
Min - Max	-100.0 - 100.0	-100.0 - 100.0	-	-100.0 - 100.0

Scales and Summary Measures only calculated if all contributing items are available

Source: P132_perfect - SF3601T.sas [SVN:27384] Data Extract: 15JUL2016 Generation Date : 09AUG2016 11:26

Table 52 SF36 – Social Functioning (SF) - Difference between level at 6 months post-OP to screening and 3-months post-OP

	Placebo	CD133+	Not treated	Total
Difference between Visit V (6-Month Follow-Up) and Visit I (Screening)				
N	36	29	0	65
Mean	12.85	4.31	-	9.04
Std	25.264	27.402	-	26.379
Median	12.50	0.00	-	0.00
Q1 - Q3	0.0 - 25.0	-12.5 - 12.5	-	0.0 - 25.0
Min - Max	-37.5 - 75.0	-37.5 - 62.5	-	-37.5 - 75.0
Difference between Visit V (6-Month Follow-Up) and Visit IV (3-Month Follow-Up)				
N	37	29	0	66
Mean	11.82	2.16	-	7.58
Std	21.839	20.613	-	21.693
Median	0.00	0.00	-	0.00
Q1 - Q3	0.0 - 25.0	0.0 - 0.0	-	0.0 - 12.5
Min - Max	-37.5 - 75.0	-37.5 - 62.5	-	-37.5 - 75.0

Scales and Summary Measures only calculated if all contributing items are available

Source: P132_perfect - SF3601T.sas [SVN:27384] Data Extract: 15JUL2016 Generation Date : 09AUG2016 11:26

Table 53 SF36 – Vitality (VT) - Difference between level at 6 months post-OP to screening and 3-months post-OP

	Placebo	CD133+	Not treated	Total
Difference between Visit V (6-Month Follow-Up) and Visit I (Screening)				
N	36	29	0	65
Mean	13.52	8.28	-	11.18
Std	21.156	20.714	-	20.962
Median	12.50	10.00	-	10.00
Q1 - Q3	0.0 - 25.0	-5.0 - 25.0	-	-5.0 - 25.0
Min - Max	-30.0 - 60.0	-35.0 - 55.0	-	-35.0 - 60.0
Difference between Visit V (6-Month Follow-Up) and Visit IV (3-Month Follow-Up)				
N	37	29	0	66
Mean	12.43	6.21	-	9.70
Std	17.741	18.740	-	18.311
Median	10.00	5.00	-	5.00
Q1 - Q3	5.0 - 25.0	-10.0 - 20.0	-	0.0 - 25.0
Min - Max	-20.0 - 75.0	-35.0 - 40.0	-	-35.0 - 75.0

Scales and Summary Measures only calculated if all contributing items are available

Source: P132_perfect - SF3601T.sas [SVN:27384] Data Extract: 15JUL2016 Generation Date : 09AUG2016 11:26

15 REFERENCE LIST

1. Leri A, Kajstura J, Anversa P. Cardiac stem cells and mechanisms of myocardial regeneration. Physiological reviews. 2005;85(4):1373-416.
2. Steinhoff. Stammzelltherapie zur Regeneration von Herzmuskelgewebe. Internist 2006(47):479-88.
3. Assmus B, Schachinger V, Teupe C, Britten M, Lehmann R, Dobert N, et al. Transplantation of Progenitor Cells and Regeneration Enhancement in Acute Myocardial Infarction (TOPCARE-AMI). Circulation. 2002;106(24):3009-17.
4. Fernandez-Aviles F, San Roman JA, Garcia-Frade J, Fernandez ME, Penarrubia MJ, de la Fuente L, et al. Experimental and clinical regenerative capability of human bone marrow cells after myocardial infarction. Circulation research. 2004;95(7):742-8.
5. Strauer BE, Brehm M, Zeus T, Kostering M, Hernandez A, Sorg RV, et al. Repair of infarcted myocardium by autologous intracoronary mononuclear bone marrow cell transplantation in humans. Circulation. 2002;106(15):1913-8.
6. Wollert KC, Meyer GP, Lotz J, Ringes-Lichtenberg S, Lippolt P, Breidenbach C, et al. Intracoronary autologous bone-marrow cell transfer after myocardial infarction: the BOOST randomised controlled clinical trial. Lancet (London, England). 2004;364(9429):141-8.
7. Wollert KC, Drexler H. Clinical applications of stem cells for the heart. Circulation research. 2005;96(2):151-63.
8. Schachinger V, Erbs S, Elsasser A, Haberbosch W, Hambrecht R, Holschermann H, et al. Intracoronary bone marrow-derived progenitor cells in acute myocardial infarction. The New England journal of medicine. 2006;355(12):1210-21.
9. Lunde K, Solheim S, Aakhus S, Arnesen H, Abdelnoor M, Egeland T, et al. Intracoronary injection of mononuclear bone marrow cells in acute myocardial infarction. The New England journal of medicine. 2006;355(12):1199-209.
10. Assmus B, Honold J, Schachinger V, Britten MB, Fischer-Rasokat U, Lehmann R, et al. Transcoronary transplantation of progenitor cells after myocardial infarction. The New England journal of medicine. 2006;355(12):1222-32.
11. Stamm C, Kleine HD, Choi YH, Dunkelmann S, Lauffs JA, Lorenzen B, et al. Intramyocardial delivery of CD133+ bone marrow cells and coronary artery bypass grafting for chronic ischemic heart disease: safety and efficacy studies. The Journal of thoracic and cardiovascular surgery. 2007;133(3):717-25.
12. Ghodsizad A, Klein HM, Borowski A, Stoldt V, Feifel N, Voelkel T, et al. Intraoperative isolation and processing of BM-derived stem cells. Cytotherapy. 2004;6(5):523-6.
13. Rosenberger WF LJ. Randomization in Clinical Trials. . New York: : Wiley; 2002.
14. JD E. nQuery Advisor®Release 5.0 User´s Guide. Los Angelos, CA;2002.
15. O´Brien RG MK. Unified Power Analysis for t-tests through Multivariate Hypotheses. Dekker M, editor. New York,: Edwards LK (Ed.); 1993.
16. Bauer PK. Evaluation of Experiments with Adaptive Interim Analyses. Biometrics. 1994:1029-41.
17. Rector TS, Carson PE, Anand IS, McMurray JJ, Zile MR, McKelvie RS, et al. Assessment of long-term effects of irbesartan on heart failure with preserved ejection fraction as measured by the minnesota living with heart failure questionnaire in the irbesartan in heart failure with preserved systolic function (I-PRESERVE) trial. Circulation Heart failure. 2012;5(2):217-25.
18. McHorney CA, Ware JE, Jr., Lu JF, Sherbourne CD. The MOS 36-item Short-Form Health Survey (SF-36): III. Tests of data quality, scaling assumptions, and reliability across diverse patient groups. Medical care. 1994;32(1):40-66.
19. Mohr FW, Morice MC, Kappetein AP, Feldman TE, Stahle E, Colombo A, et al. Coronary artery bypass graft surgery versus percutaneous coronary intervention in patients with three-vessel disease and left main coronary disease: 5-year follow-up of the randomised, clinical SYNTAX trial. Lancet (London, England). 2013;381(9867):629-38.
20. Solomon SD, Zelenkofske S, McMurray JJ, Finn PV, Velazquez E, Ertl G, et al. Sudden death in patients with myocardial infarction and left ventricular dysfunction, heart failure, or both. The New England journal of medicine. 2005;352(25):2581-8.

21. Volpi A, De Vita C, Franzosi MG, Geraci E, Maggioni AP, Mauri F, et al. Determinants of 6-month mortality in survivors of myocardial infarction after thrombolysis. Results of the GISSI-2 data base. The Ad hoc Working Group of the Gruppo Italiano per lo Studio della Sopravvivenza nell'Infarto Miocardico (GISSI)-2 Data Base. Circulation. 1993;88(2):416-29.
22. Nasseri BA, Ebell W, Dandel M, Kukucka M, Gebker R, Doltra A, et al. Autologous CD133+ bone marrow cells and bypass grafting for regeneration of ischaemic myocardium: the Cardio133 trial. European heart journal. 2014;35(19):1263-74.
23. Serruys PW, Morice MC, Kappetein AP, Colombo A, Holmes DR, Mack MJ, et al. Percutaneous coronary intervention versus coronary-artery bypass grafting for severe coronary artery disease. The New England journal of medicine. 2009;360(10):961-72.

16 APPENDIX A STUDY INFORMATION

16.1 Appendix A 1 Protocol, protocol amendments and Notes-to-File

16.2 Appendix A 2 Sample Case Report Form

16.3 Appendix A 3 Independent Ethics Committee membership list and sample patient information including Informed Consent Form

16.4 Appendix A 4 Brief résumé of Investigator and other important study personnel

16.5 Appendix A 5 Certificate of Analysis and Listing of Patients Receiving Investigational Drug(s) from Specific Batches

16.6 Appendix A 6 Randomisation list

16.7 Appendix A 7 Audit certificates

16.8 Appendix A 8 Statistical Analysis Plan

16.9 Appendix A 9 Laboratory accreditation, inter-laboratory standardization and reference ranges

16.10 Appendix A 10 Publications based on the study

16.11 Appendix A 11 Important publications referenced in the report

17 APPENDIX B STATISTICAL OUTPUT

18 APPENDIX C INDIVIDUAL DATA LISTINGS

19 APPENDIX D SELECTED CASE REPORT FORMS AND SUBJECT NARRATIVES
