# Supplementary material for: Hematopoietic stem-cell senescence and myocardial repair - Coronary artery disease genotype/phenotype analysis of post-MI myocardial regeneration response induced by CABG/CD133+ bone marrow hematopoietic stem cell treatment in RCT PERFECT Phase 3
Source: eBioMedicine. 2020 Jul 4;57:102862. doi: 10.1016/j.ebiom.2020.102862 (PMC7339012; doi:10.1016/j.ebiom.2020.102862)
Supplement: Supplementary file 13 [file mmc13.docx]

Supplemental Figures:

**
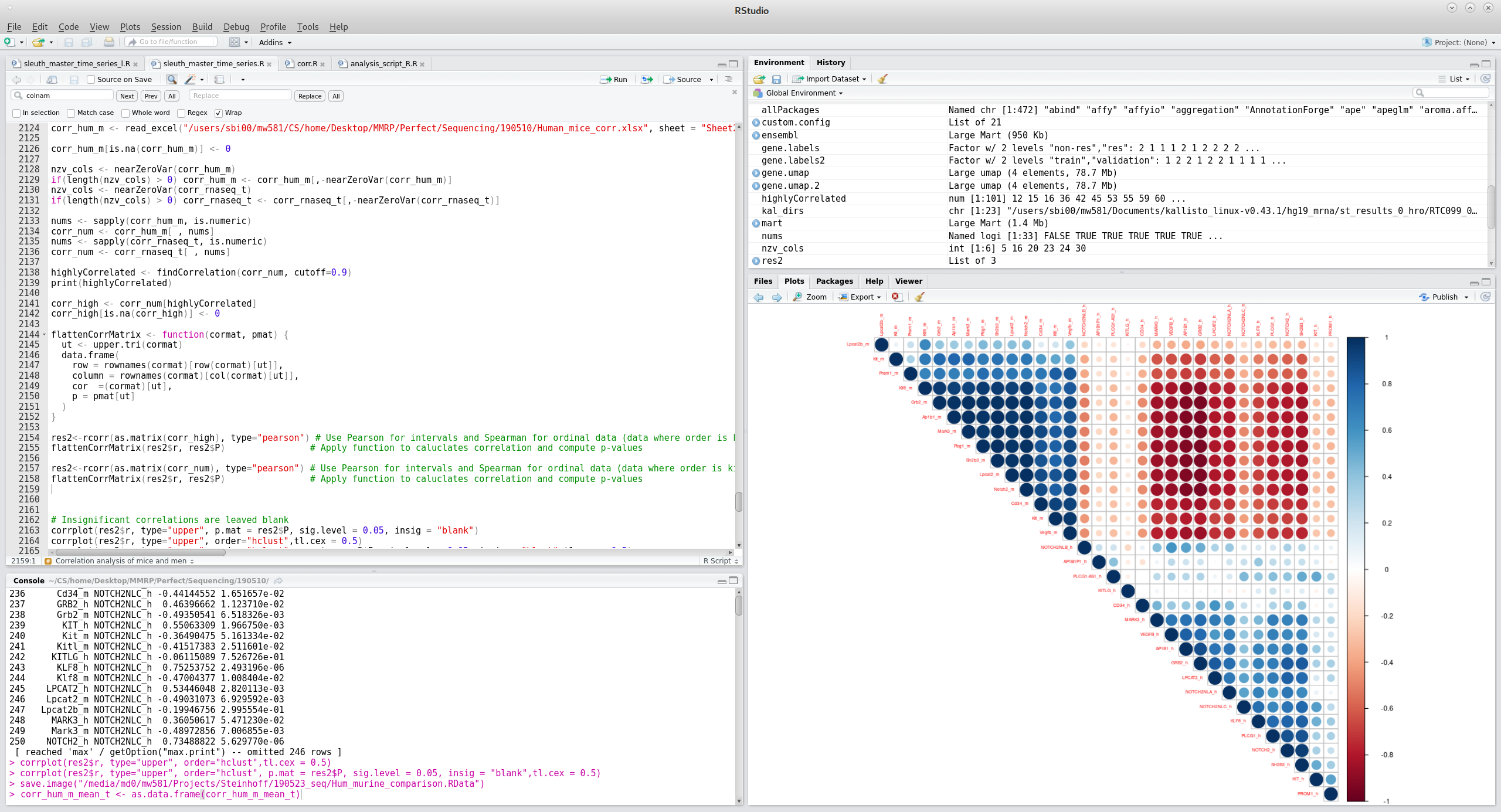
**

**
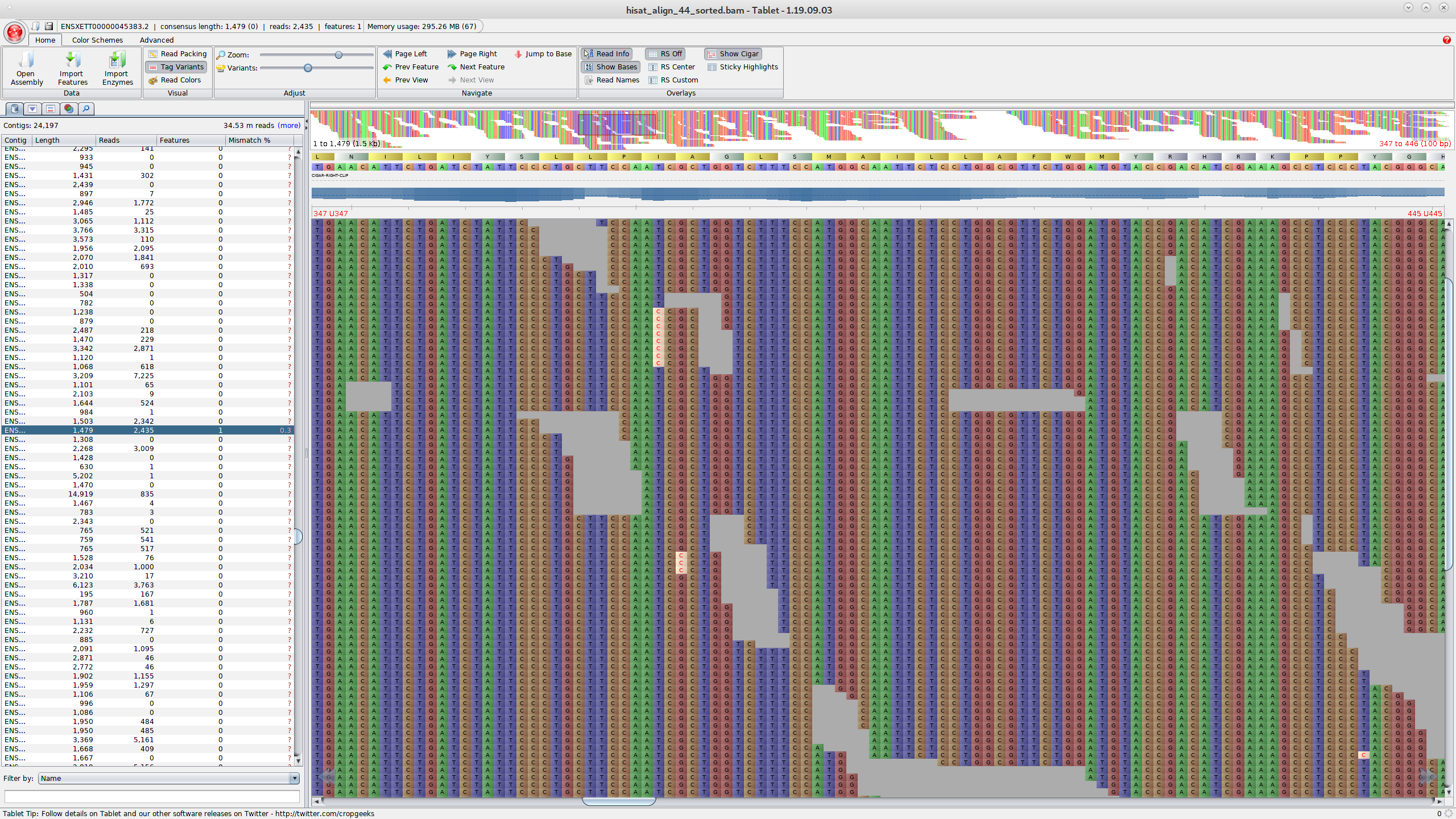
**

**Supplementary Figure S1a:** Screenshot from analysis


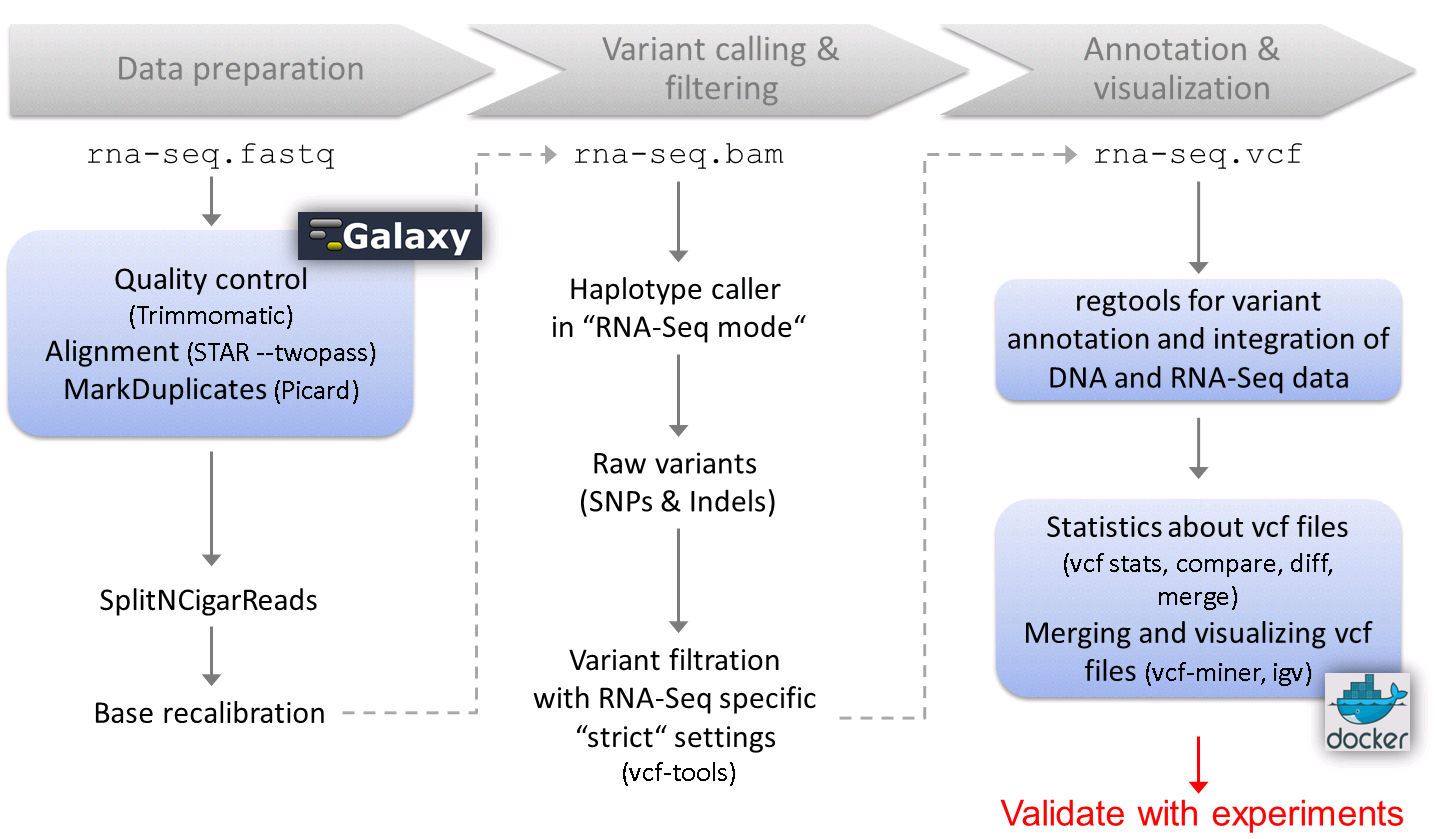


**Supplementary Figure S1b: Variant calling workflow used for RNA-Seq datasets**. The fastq quality control and preprocessing was done by using Trimmomatic (1) Afterwards the reads have been aligned with the 2-pass mode of STAR (2) and gene duplicates have been marked and corrected by Picard (https://github.com/broadinstitute/picard - Accessed at 06.09.2019https://github.com/broadinstitute/picard - Accessed at 06.06.2018). The following data preparation and variant calling procedures have been done as indicated by programs embedded in the Gatk-toolkit (3). The subsequent variant filtering was done with vcf-tools (4) by considering only variants as called, if there are more than five independent reads for a single variant. Annotation and visualization have been achieved by using RegTools (5) and vcf-miner (6) as well as igv (7) respectively.


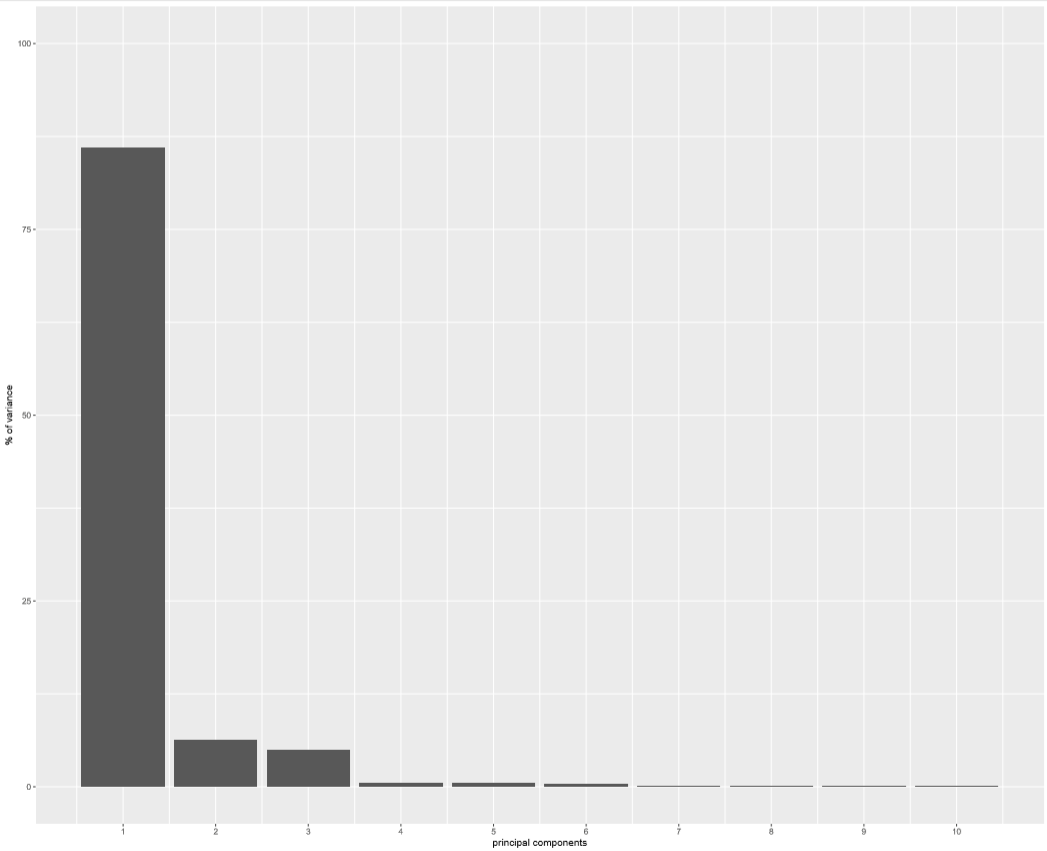


**Supplementary Figure S1c: Variation of the first ten Principal Components (in percent) for gene expression clustering of all 23 patients.**

**Supplemental References**

1. Bolger AM, Lohse M, Usadel B. Trimmomatic: a flexible trimmer for Illumina sequence data. Bioinformatics [Internet]. 2014 Aug 1 [cited 2018 Mar 26];30(15):2114–20. Available from: http://www.ncbi.nlm.nih.gov/pubmed/24695404

2. Dobin A, Gingeras TR. Mapping RNA-seq Reads with STAR. Curr Protoc Bioinforma [Internet]. 2015 Sep 3 [cited 2018 Nov 29];51:11.14.1-19. Available from: http://www.ncbi.nlm.nih.gov/pubmed/26334920

3. McKenna A, Hanna M, Banks E, Sivachenko A, Cibulskis K, Kernytsky A, et al. The Genome Analysis Toolkit: a MapReduce framework for analyzing next-generation DNA sequencing data. Genome Res [Internet]. 2010 Sep [cited 2018 Jan 12];20(9):1297–303. Available from: http://www.ncbi.nlm.nih.gov/pubmed/20644199

4. Danecek P, Auton A, Abecasis G, Albers CA, Banks E, DePristo MA, et al. The variant call format and VCFtools. Bioinformatics [Internet]. 2011 Aug 1 [cited 2018 Nov 29];27(15):2156–8. Available from: https://academic.oup.com/bioinformatics/article-lookup/doi/10.1093/bioinformatics/btr330

5. Feng Y-Y, Ramu A, Cotto KC, Skidmore ZL, Kunisaki J, Conrad DF, et al. RegTools: Integrated analysis of genomic and transcriptomic data for discovery of splicing variants in cancer. bioRxiv [Internet]. 2018 Oct 5 [cited 2018 Nov 29];436634. Available from: https://www.biorxiv.org/content/early/2018/10/05/436634

6. Hart SN, Duffy P, Quest DJ, Hossain A, Meiners MA, Kocher J-P. VCF-Miner: GUI-based application for mining variants and annotations stored in VCF files. Brief Bioinform [Internet]. 2016 Mar [cited 2018 Nov 29];17(2):346–51. Available from: http://www.ncbi.nlm.nih.gov/pubmed/26210358

7. Thorvaldsdottir H, Robinson JT, Mesirov JP. Integrative Genomics Viewer (IGV): high-performance genomics data visualization and exploration. Brief Bioinform [Internet]. 2013 Mar 1 [cited 2018 Nov 29];14(2):178–92. Available from: https://academic.oup.com/bib/article-lookup/doi/10.1093/bib/bbs017


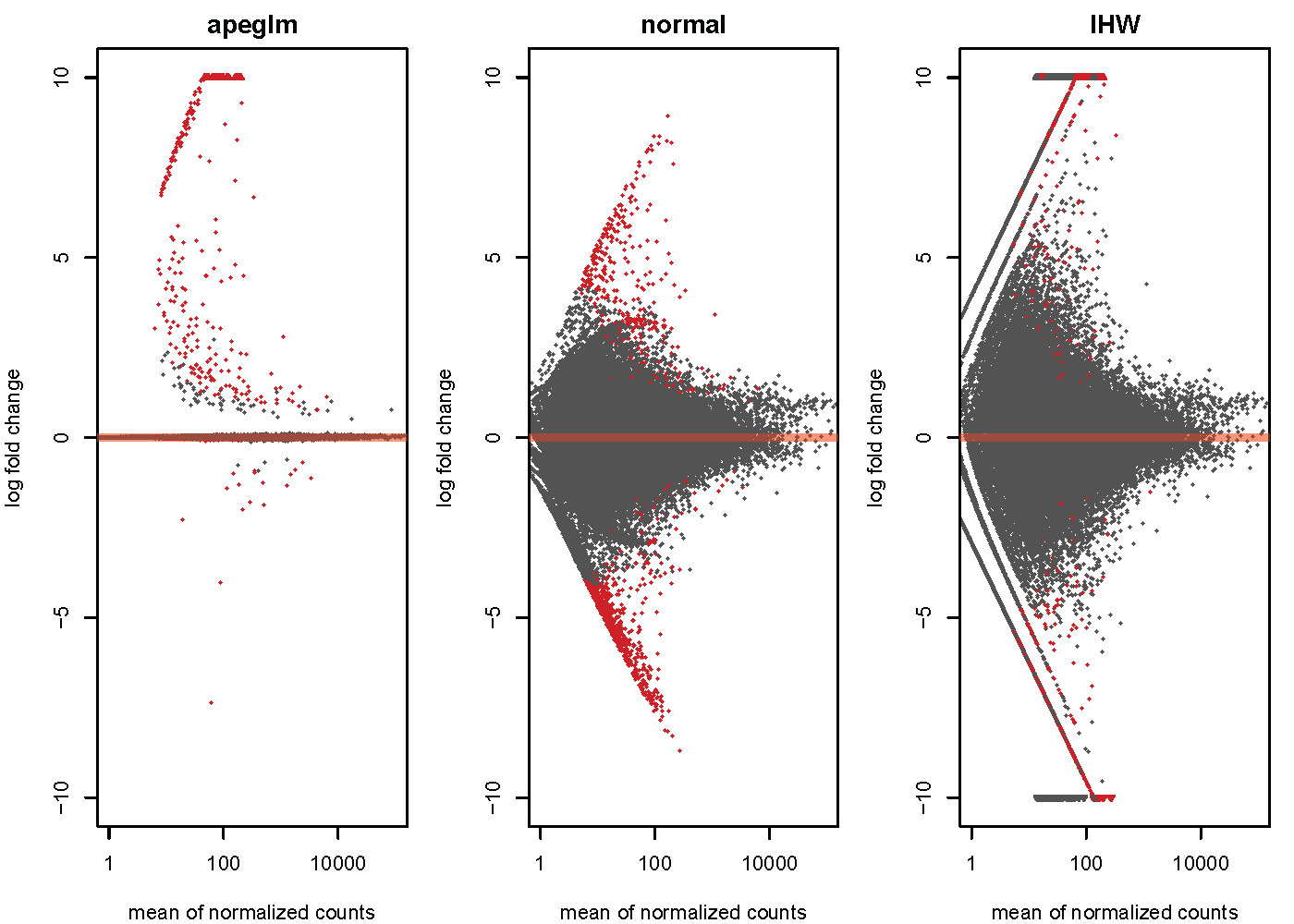


**Supplementary Figure S1c: Evaluation R*vs*NR differential gene expression analysis**

**

**

**Supplementary Figure S2: Pearson correlation analysis of different gene transcripts of PROM1/CD133 and NOTCH2 isoforms.**


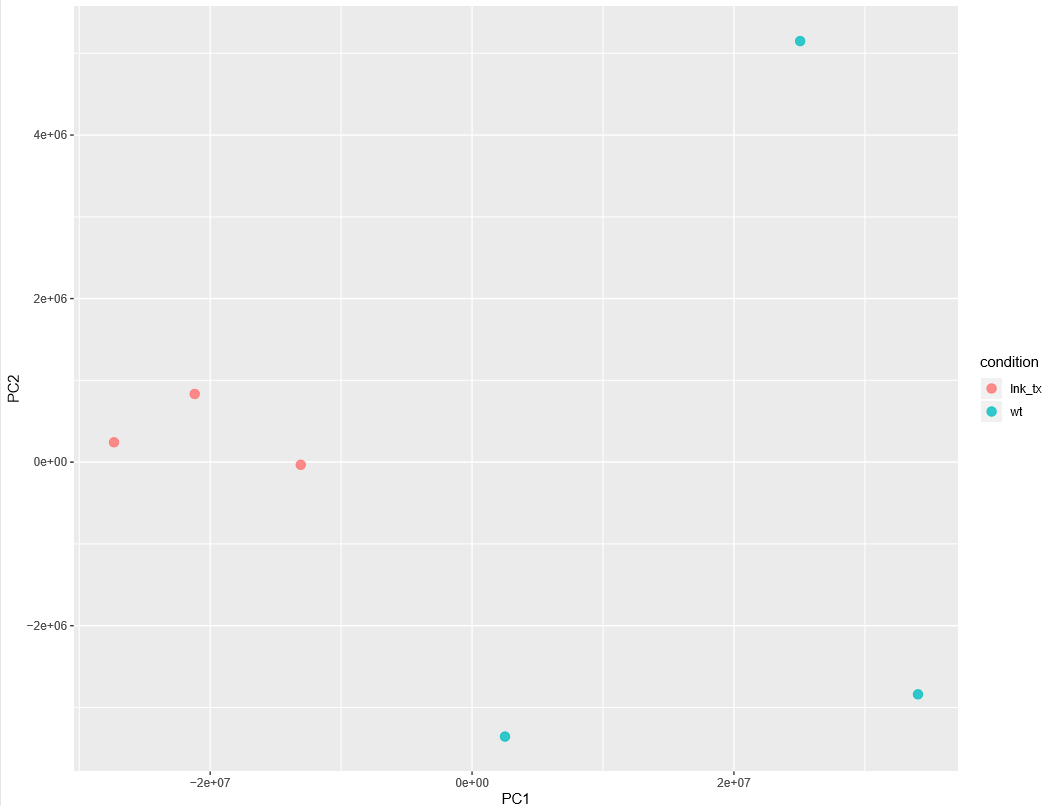


**Supplementary Figure S3:** **PCA plot for mice RNA-Seq datasets**


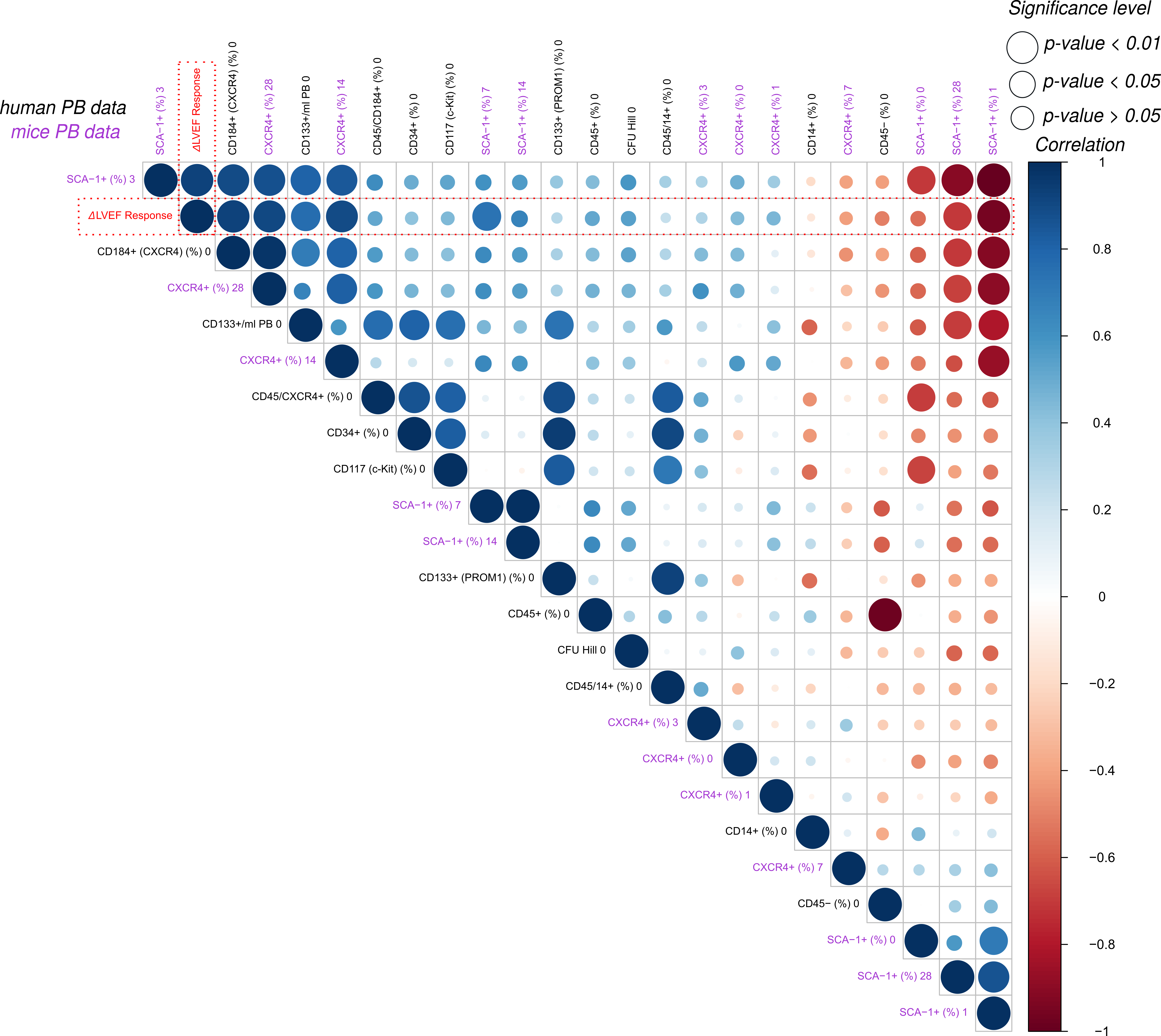


**Supplementary Figure S4:** Pearson correlation analysis between the mouse infarction model (SH2B3/LNK ^-/-^ *vs.* WT) and human phase 3 PERFECT trial (ΔLVEF responder *vs.* non-responder). The human ΔLVEF response is highlighted for an improved visual analysis of important correlations. The color scale, ranging from *1* to *-1* in the upper panel (blue to red), represents the correlation between the different factors. The size of the dots represents the significance (p<0^.^01, p<0^.^05, and p>0^.^05, Pearson correlation) of the respective correlation. a) Comparison of peripheral blood (PB) circulating cells and biomarkers between mice (purple) and human (serum) (black).


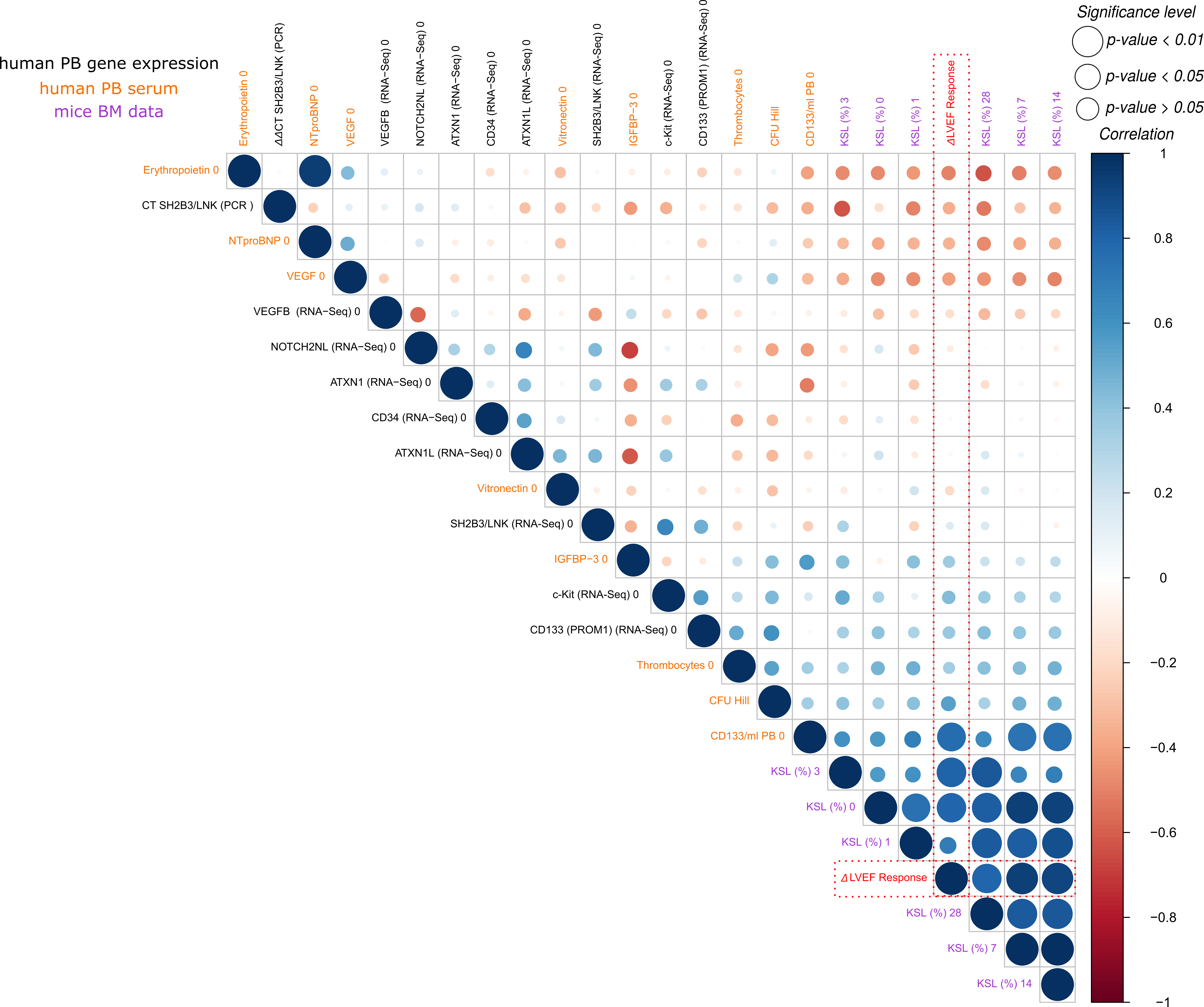


**Supplementary Figure S5:** Pearson correlation analysis between the mouse infarction model (SH2B3/LNK ^-/-^ vs. WT) and human phase 3 PERFECT trial (ΔLVEF responder vs. non-responder). The human ΔLVEF response is highlighted for an improved visual analysis of important correlations. The color scale, ranging from 1 to -1 in the upper panel (blue to red), represents the correlation between the different factors. The size of the dots represents the significance (p<0^.^01, p<0^.^05, and p>0^.^05, Pearson correlation) of the respective correlation. Comparison of peripheral blood (PB) circulating cells and biomarkers between mice (purple) and human (serum) (orange).
